# Development of First-in-Class Dual Sirt2/HDAC6 Inhibitors as Molecular Tools for Dual Inhibition of Tubulin Deacetylation

**DOI:** 10.1021/acs.jmedchem.3c01385

**Published:** 2023-10-30

**Authors:** Laura Sinatra, Anja Vogelmann, Florian Friedrich, Margarita A. Tararina, Emilia Neuwirt, Arianna Colcerasa, Philipp König, Lara Toy, Talha Z. Yesiloglu, Sebastian Hilscher, Lena Gaitzsch, Niklas Papenkordt, Shiyang Zhai, Lin Zhang, Christophe Romier, Oliver Einsle, Wolfgang Sippl, Mike Schutkowski, Olaf Gross, Gerd Bendas, David W. Christianson, Finn K. Hansen, Manfred Jung, Matthias Schiedel

**Affiliations:** † Institute for Drug Discovery, Medical Faculty, Leipzig University, Brüderstraße 34, 04103 Leipzig, Germany; ‡ Institute of Pharmaceutical Sciences, University of Freiburg, Albertstraße 25, 79104 Freiburg, Germany; § Roy and Diana Vagelos Laboratories, Department of Chemistry, University of Pennsylvania, 231 South 34th Street, Philadelphia, Pennsylvania 19104-6323, United States; ∥ Institute of Neuropathology, Medical Center−University of Freiburg, Faculty of Medicine, University of Freiburg, Breisacherstraße 64, 79106 Freiburg, Germany; ⊥ CIBSS−Centre for Integrative Biological Signalling Studies, University of Freiburg, Schänzlestraße 18, 79104 Freiburg, Germany; # Department of Pharmaceutical & Cell Biological Chemistry, Pharmaceutical Institute, University of Bonn, An der Immenburg 4, 53121 Bonn, Germany; ∇ Department of Chemistry and Pharmacy, Medicinal Chemistry, Friedrich-Alexander-University Erlangen-Nürnberg, Nikolaus-Fiebiger-Straße 10, 91058 Erlangen, Germany; ○ Department of Medicinal Chemistry, Institute of Pharmacy, Martin-Luther University of Halle-Wittenberg, Wolfgang-Langenbeck-Straße 2-4, 06120 Halle (Saale), Germany; ◆ Institute of Biochemistry, University of Freiburg, Albertstraße 21, 79104 Freiburg, Germany; ¶ Institut de Génétique et de Biologie Moléculaire et Cellulaire (IGBMC), Université de Strasbourg, CNRS UMR 7104, Inserm UMR-S 1258, 1 rue Laurent Fries, F-67400 Illkirch, France; □ Department of Enzymology, Charles Tanford Protein Center, Institute of Biochemistry and Biotechnology, Martin-Luther-University Halle-Wittenberg, 06120 Halle, Germany; ● Center for Basics in NeuroModulation (NeuroModulBasics), Faculty of Medicine, University of Freiburg, Breisacherstraße 64, 79106 Freiburg, Germany; ◇ Institute of Medicinal and Pharmaceutical Chemistry, Technische Universität Braunschweig, Beethovenstraße 55, 38106 Braunschweig, Germany

## Abstract

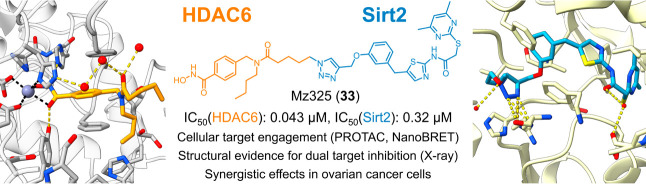

Dysregulation of both tubulin deacetylases sirtuin 2 (Sirt2) and the histone deacetylase 6 (HDAC6) has been associated with the pathogenesis of cancer and neurodegeneration, thus making these two enzymes promising targets for pharmaceutical intervention. Herein, we report the design, synthesis, and biological characterization of the first-in-class dual Sirt2/HDAC6 inhibitors as molecular tools for dual inhibition of tubulin deacetylation. Using biochemical *in vitro* assays and cell-based methods for target engagement, we identified Mz325 (**33**) as a potent and selective inhibitor of both target enzymes. Inhibition of both targets was further confirmed by X-ray crystal structures of Sirt2 and HDAC6 in complex with building blocks of **33**. In ovarian cancer cells, **33** evoked enhanced effects on cell viability compared to single or combination treatment with the unconjugated Sirt2 and HDAC6 inhibitors. Thus, our dual Sirt2/HDAC6 inhibitors are important new tools to study the consequences and the therapeutic potential of dual inhibition of tubulin deacetylation.

## Introduction

**Figure 1:**
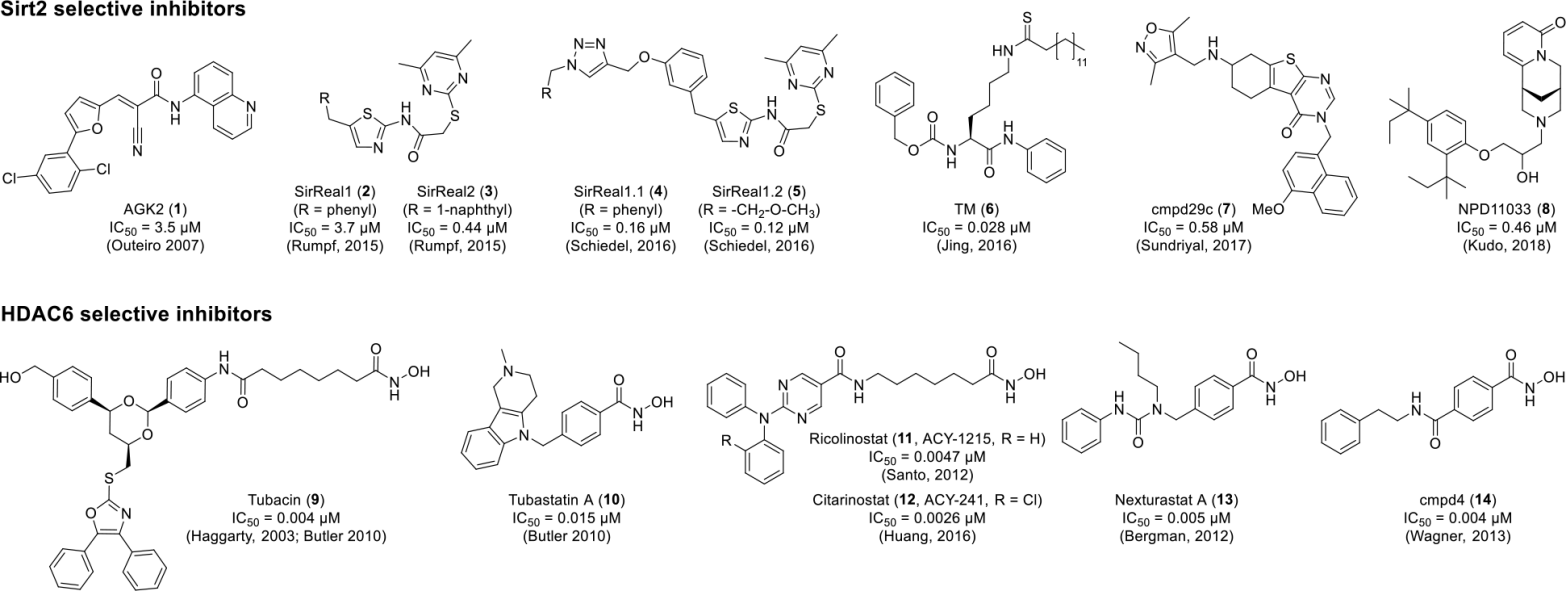
Chemical structures and reported IC_50_ values of highly potent and selective inhibitors of Sirt2 and HDAC6, respectively. References: AGK2 (**1**),^15^ SirReal1 (**2**), SirReal2 (**3**),^18^ SirReal1.1 (**4**), SirReal1.2 (**5**),^19^ TM (**6**),^10^ cmpd29c (**7**),^20^ NDP11033 (**8**),^21^ Tubacin (**9**),^22,23^ Tubastatin A (**10**),^23^ Ricolinostat (ACY-1215, **11**),^24^ Citarinostat (ACY-241, **12**),^25^ Nexturastat A (**13**),^26^ and cmpd4 (**14**).^27^

Both Sirt2 and HDAC6 are protein deacylases that cleave off acetyl as well as other acyl groups from the ε-amino group of lysines in their substrate proteins. While Sirt2 features an NAD^+^-dependent catalytic mechanism and belongs to class III HDACs, also referred to as sirtuins, HDAC6 is a Zn^2+^-dependent lysine deacylase and has been classified as a class IIb HDAC. Despite having different catalytic mechanisms of lysine deacylation, Sirt2 and HDAC6 share several common features, including their substrate spectrum, subcellular localization, and potential as a therapeutic target. Although both deacylases are classified as histone deacetylases, they share acetylated α-tubulin (α-tubulin K40ac) as their major substrate and are hence frequently referred to as tubulin deacetylases.^1,2^ Beside α-tubulin, the cortical actin binding protein (cortactin) and the oncogene K-RAS have been identified as common substrates of Sirt2- and HDAC6-dependent deacetylation,^3,4^ thereby also indicating a certain degree of functional redundancy of both enzymes. In the case of Sirt2- and HDAC6-mediated tubulin deacetylation, the degree of functional redundancy is dependent on the architecture of hyperacetylated tubulin. Whereas most microtubule structures can be deacetylated by both enzymes, deacetylation of perinuclear microtubules can only be achieved by Sirt2.^5^ A further common feature of Sirt2 and HDAC6 is their subcellular localization. Both enzymes are primarily found in the cytosol, colocalized with microtubules, and were even shown to interact with each other by means of coimmunoprecipitation.^1,2^ A dysregulation of both Sirt2 and HDAC6 activity has been associated with the pathogenesis of cancer^4,6−11^ and neurodegeneration,^12−15^ thus making these two enzymes promising targets for pharmaceutical intervention. This has prompted intense efforts in the development of small molecule inhibitors of Sirt2 and HDAC6, which are extensively reviewed elsewhere.^16,17^ A selection of highly potent and selective inhibitors of Sirt2 (**1**–**8**) and HDAC6 (**9**–**14**), respectively, is shown in Figure 1. In the case of HDAC6, the development of isotype preferential small molecule inhibitors already culminated in first drug candidates that progressed to clinical trials. For example, the orally bioavailable ricolinostat (ACY-1215, **11**) and its second generation analogue citarinostat (ACY-241, **12**), entered several clinical trials, primarily for the treatment of multiple myeloma, but also for other diseases, including malignant melanoma, advanced solid tumors, nonsmall cell lung cancer, lymphoid malignancies, and painful diabetic peripheral neuropathy.^17^ In most of these clinical trials, the HDAC6 preferential inhibitor was combined with a second drug.^17^

**Figure 2:**
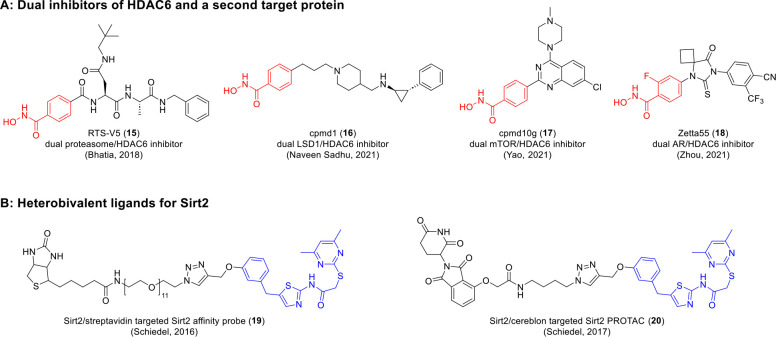
(A) Chemical structures of reported dual inhibitors of HDAC6 and a second target protein.^32−35^ The Zn^2+^-binding core structure of the HDAC6-targeted subunit, a *N*-hydroxybenzamide, is highlighted in red. (B) Chemical structures of heterobifunctional ligands for Sirt2.^19,37^ The core structure of the Sirt2-targeted inhibitor, a SirReal, is highlighted in blue.

In general, a combinatorial inhibition of two different targets involved in disease progression often causes synergistic or additive effects and can reduce the potential for developing drug resistance,^28,29^ which is especially relevant for cancer-targeted therapies. Additionally, dual-target therapies generally yield a high efficacy,^30^ thereby enabling a reduction in therapeutic doses and hence in side effects compared to single-target drug regimens.^28,31^ The common practice of combinatorial application of HDAC6 inhibitors also triggered the development of dual inhibitors for HDAC6 and a second target protein. Dual-targeting inhibitors for mTOR/HDAC6,^32^ LSD1/HDAC6,^33^ AR/HDAC6,^34^ and proteasome/HDAC6^35^ were recently reported as novel anticancer drugs (Figure 2A, **15**–**18**). One thing that has facilitated drug discovery in this regard is the simple design of HDAC6 inhibitors. As can be seen in Figure 1, HDAC6 inhibitors usually comprise a hydroxamic acid as zinc binding group, which is crucial for the chelation of the zinc ion inside the active site tunnel, a linker region, and a rather bulky cap group. Fortunately, HDAC6 tolerates various structural modifications at the cap group of its inhibitors, thereby providing sufficient scope for hybridization approaches toward HDAC6 inhibitor-based multitarget drugs. Generally, the approach of combining two or more independent modes of action into one single molecule has several potential advantages over standard combination therapies, including a reduced risk of drug–drug interactions, more predictable pharmacokinetics, improved patient compliance, and simultaneous presence of all active principles in the tissues where the molecule is intended to work.^36^ Compared to HDAC6, Sirt2 has been considered less frequently for dual-targeting inhibitor approaches. However, the successful development of a Sirt2 affinity probe (**19**) and a Sirt2-targeted PROTAC (**20**, Figure 2B) shows that Sirt2 inhibitors, such as the Sirtuin rearranging ligands (SirReals), can successfully be used for the design of heterobivalent ligands.^19,37^

Herein, we report the development of the first-in-class dual Sirt2/HDAC6 inhibitors as molecular tools for a dual inhibition of tubulin deacetylation. In contrast to sole inhibition of one of these two tubulin deacetylases, which can at least partially be counteracted by the other noninhibited enzyme, a dual Sirt2/HDAC6 inhibition leads to a more comprehensive blockade of tubulin deacetylation. Several of our dual Sirt2/HDAC6 inhibitors showed potent *in vitro* inhibition of both target enzymes. Cellular activity of our dual Sirt2/HDAC6 inhibitors was verified by immunofluorescence microscopy as well as via a cell-based NanoBRET assay. Three new crystal structures of Sirt2 and HDAC6, respectively, in complex with Sirt2- or HDAC6-targeted building blocks, provided structural evidence for the interaction of our dual Sirt2/HDAC6 inhibitors with both targets. In W1 ovarian cancer cells, our lead structure for dual Sirt2/HDAC6 inhibition evoked enhanced effects on cell viability compared to single or combination treatment with the unconjugated Sirt2 and HDAC6 inhibitors. Hence, dual Sirt2/HDAC6 inhibitors are valuable new molecular tools to study the consequences and the therapeutic potential of a dual inhibition of tubulin deacetylation.

## Results and Discussion

### Design Concept

**Figure 3:**
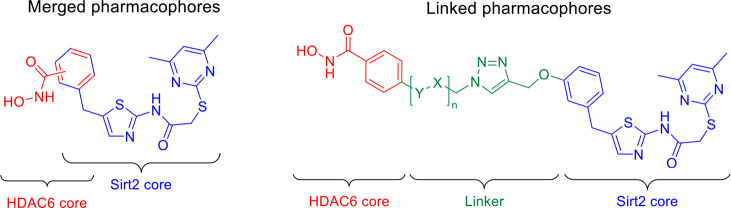
Design of dual Sirt2/HDAC6 inhibitors with merged or linked pharmacophores.

For the design of our dual Sirt2/HDAC6 inhibitors, we referred to the SirReals as Sirt2 ligands and *N*-hydroxybenzamides as HDAC6 inhibitors. We selected these two pharmacophores for the following reasons: (i) both are highly selective for their respective target protein,^18,19,26,27^ (ii) their binding modes were previously elucidated via X-ray cocrystallography,^18,19,35^ and (iii) they were already successfully utilized for the development of heterobivalent ligands or dual-targeted inhibitors, respectively.^19,32−35,37^ The chemical structures of the reported heterobivalent Sirt2 ligands and the dual HDAC6 inhibitors (Figure 2) provided robust evidence on how the SirReal-based pharmacophore and the *N*-hydroxybenzamide can be combined in one single molecule. Based on these insights, we designed our dual Sirt2/HDAC6 inhibitors by either merging or linking the Sirt2- and HDAC6-targeted pharmacophores, as shown in Figure 3. For dual Sirt2/HDAC6 inhibitors with merged Sirt2- and HDAC6-targeted pharmacophores, we directly installed a hydroxamic acid in *meta*- or *para*-position of the benzyl moiety of SirReal1 (**2**). For dual Sirt2/HDAC6 inhibitors with linked Sirt2- and HDAC6-targeted pharmacophores, we incorporated a triazole-based linker unit into the design of these ligands, thereby enabling a conjugation via Cu(I)-catalyzed Huisgen cycloaddition.^38−40^ As linker lengths and composition are critical parameters when linking two pharmacophores, we mainly focused on linker variations for structure–activity relationship studies. In order to also provide a nonselective HDAC/Sirt2 inhibitor, we linked suberoylanilide hydroxamic acid (SAHA, also known as vorinostat), a nonselective inhibitor of Zn^2+^-dependent HDACs,^41^ to our SirReal-based pharmacophore (see below).

### Chemical Synthesis

#### Synthesis of Dual Sirt2/HDAC6 Inhibitors with Merged Pharmacophores

**Scheme 1:**
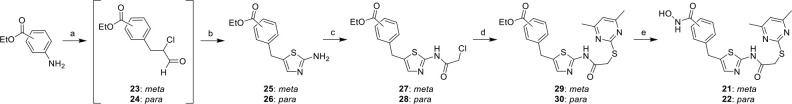
Synthesis of Dual Sirt2/HDAC6 Inhibitors with Merged Pharmacophores (**21**, **22**) Reagents and conditions: (a) NaNO_2_, HCl, water, −5 to 0 °C, 30 min, then acrolein, CuCl_2_·2H_2_O, CaO, acetone, 20 °C, 2 h; (b) thiourea, ethanol, reflux, 2–24 h, 3–17% yield over three steps; (c) chloroacetyl chloride, DIPEA, acetonitrile, 0–20 °C, 2 h, 57–62% yield; (d) 4,6-dimethyl-2-methylsulfanylpyrimidine, Na_2_CO_3_, KI, DMSO, 20 °C, 1.5–2 h, 47–91% yield; (e) H_2_NOH, CH_2_Cl_2_, MeOH, 0 °C, 10 min, then NaOH, 0–20 °C, 2.5 h, 28–33% yield.

The dual Sirt2/HDAC6 inhibitors with merged pharmacophores **21** and **22** were synthesized by referring to previously published protocols for the preparation of SirReals (Scheme 1).^18,42^ In brief, the α-chloropropanal intermediates **23** and **24**, which were synthesized via diazotization of the respective aniline and subsequent Meerwein reaction, were converted to the aminothiazoles **25** and **26** by means of a condensation with thiourea. The aminothiazoles were then chloroacetylated to obtain the amides **27** and **28**. A subsequent nucleophilic substitution of the chloroalkyl species with dimethylmercaptopyrimidine generated compounds **29** and **30**. Finally, the *N*-hydroxybenzamides **21** and **22** were obtained by a reaction of the ethyl benzoates with hydroxylamine.

#### Synthesis of Dual Sirt2/HDAC6 Inhibitors with Linked Pharmacophores

**Scheme 2:**
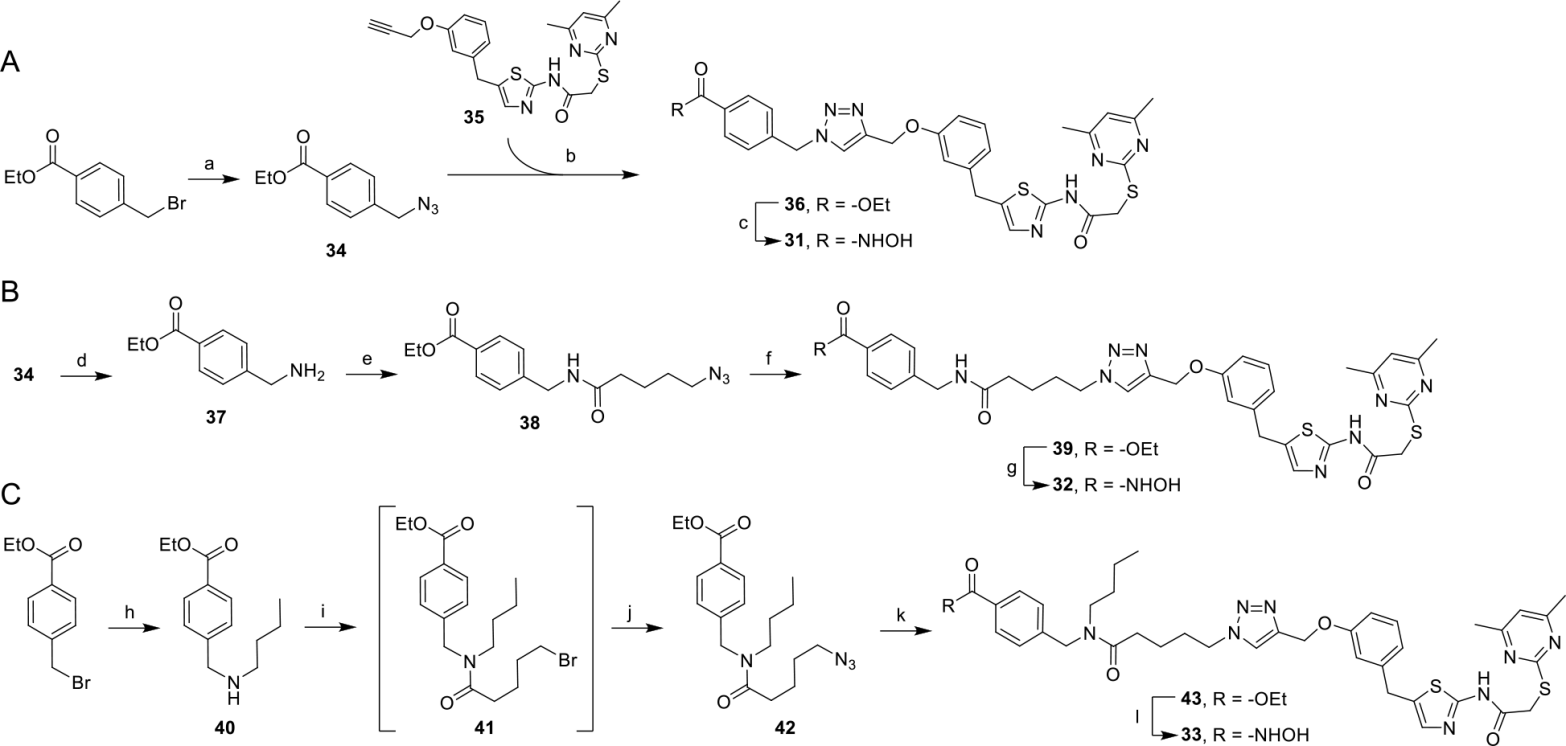
Solution-Phase Synthesis of Dual Sirt2/HDAC6 Inhibitors with Linked Pharmacophores (A) Synthesis of **31**, reagents and conditions: (a) NaN_3_, DMF, water, 20 °C, 16 h, 93% yield; (b) **35**, sodium ascorbate, CuSO_4_·5H_2_O, TBTA, water/*t*BuOH/DMF (1:1:1), 20 °C, 12 h, 83% yield; (c) H_2_NOH, CH_2_Cl_2_, MeOH, 0 °C, 10 min, then NaOH, 0–20 °C, 3.5 h, 70% yield. (B) Synthesis of **32**, reagents and conditions: (d) PPh_3_, THF, water, 20–75 °C, 2 h, 97% yield; (e) 5-azidopentanoic acid, HATU, DIPEA, DMF, 0–20 °C, 16 h, 98% yield; (f) **35**, sodium ascorbate, CuSO_4_·5H_2_O, TBTA, water/*t*BuOH/DMF (1:1:1), 20 °C, 12 h, 89% yield; (g) H_2_NOH, CH_2_Cl_2_, MeOH, 0 °C, 10 min, then NaOH, 0–20 °C, 3.5 h, 47% yield. (C) Synthesis of **33**, reagents and conditions: (h) *n*-butylamine, THF, 20 °C, 3 h, 98% yield; (i) 5-bromopentanoic acid, TBTU, DIPEA, CH_2_Cl_2_, DMF, 0 °C, 15 min, then add **40**, 20 °C, 2 h; (j) NaN_3_, DMSO, 45 °C, 16 h, 56% yield over two steps; (k) **35**, sodium ascorbate, CuSO_4_·5H_2_O, TBTA, water/*t*BuOH/DMF (1:1:1), 20 °C, 16 h, 88% yield; (l) H_2_NOH, CH_2_Cl_2_, MeOH, 0 °C, 10 min, then NaOH, 0–20 °C, 3.5 h, 52% yield.

For the synthesis of dual Sirt2/HDAC6 inhibitors with linked pharmacophores, we utilized both solution-phase and solid-phase chemistry. Compounds with short alkyl linkers between the Sirt2- and HDAC6-inhibiting pharmacophores (**31**–**33**) were conjugated via standard solution-phase Cu(I)-catalyzed Huisgen cycloaddition.^38,39^ To this end, we used the previously reported alkynylated SirReal analogue **35** (Scheme 2A), which has already successfully been used to furnish other heterobivalent Sirt2 ligands, including the Sirt2 PROTAC and the Sirt2-targeted affinity probe (Figure 2B).^19,37^ The clickable, azido-functionalized subunits needed for targeting HDAC6 were synthesized as shown in Scheme 2. In brief, the synthesis of the dual Sirt2/HDAC6 inhibitor **31** (Scheme 2A) was initiated by a nucleophilic substitution of ethyl 4-(bromomethyl)benzoate with sodium azide to furnish **34**. A subsequent Cu(I)-catalyzed Huisgen cycloaddition of **34** and **35** gave the triazole **36**. Similar to the aforementioned syntheses of the dual Sirt2/HDAC6 inhibitors with merged pharmacophores (**21**, **22**), the final *N*-hydroxybenzamide of **31** was furnished by hydroxylaminolysis of the ester precursor **36**. For the synthesis of the dual Sirt2/HDAC6 inhibitor **32** (Scheme 2B), we started with a Staudinger reduction of the azide **34** to the amine **37**, which was subsequently acylated with 5-azidopentanoic acid to obtain the azido-functionalized amide **38**. Following a click reaction between **38** and **35** to give **39**, the *N*-hydroxybenzamide **32** was furnished by a reaction of the ethyl benzoate moiety of **39** with hydroxylamine. The design of the dual Sirt2/HDAC6 inhibitor **33** (Scheme 2C) with its ternary *N*-butyl amide substructure was inspired by nexturastat A (**13**, Figure 1) that also features a *N*-butyl substituent at the ternary nitrogen atom of its urea subunit. **33** was synthesized starting with a nucleophilic substitution of ethyl 4-(bromomethyl)benzoate with butyl amine to furnish the secondary amine **40**. An amide coupling between **40** and 5-bromopentanoic acid gave the amide **41**. Subsequent conversion of the obtained alkyl bromide **41** with sodium azide resulted in the azido-functionalized **42**. The triazole **43** was afforded by a conjugation of **42** with the alkynylated SirReal **35** via click reaction. Again, the final *N*-hydroxybenzamide, in this case **33**, was obtained by an hydroxylaminolysis of the ethyl benzoate precursor **43**.

**Scheme 3:**
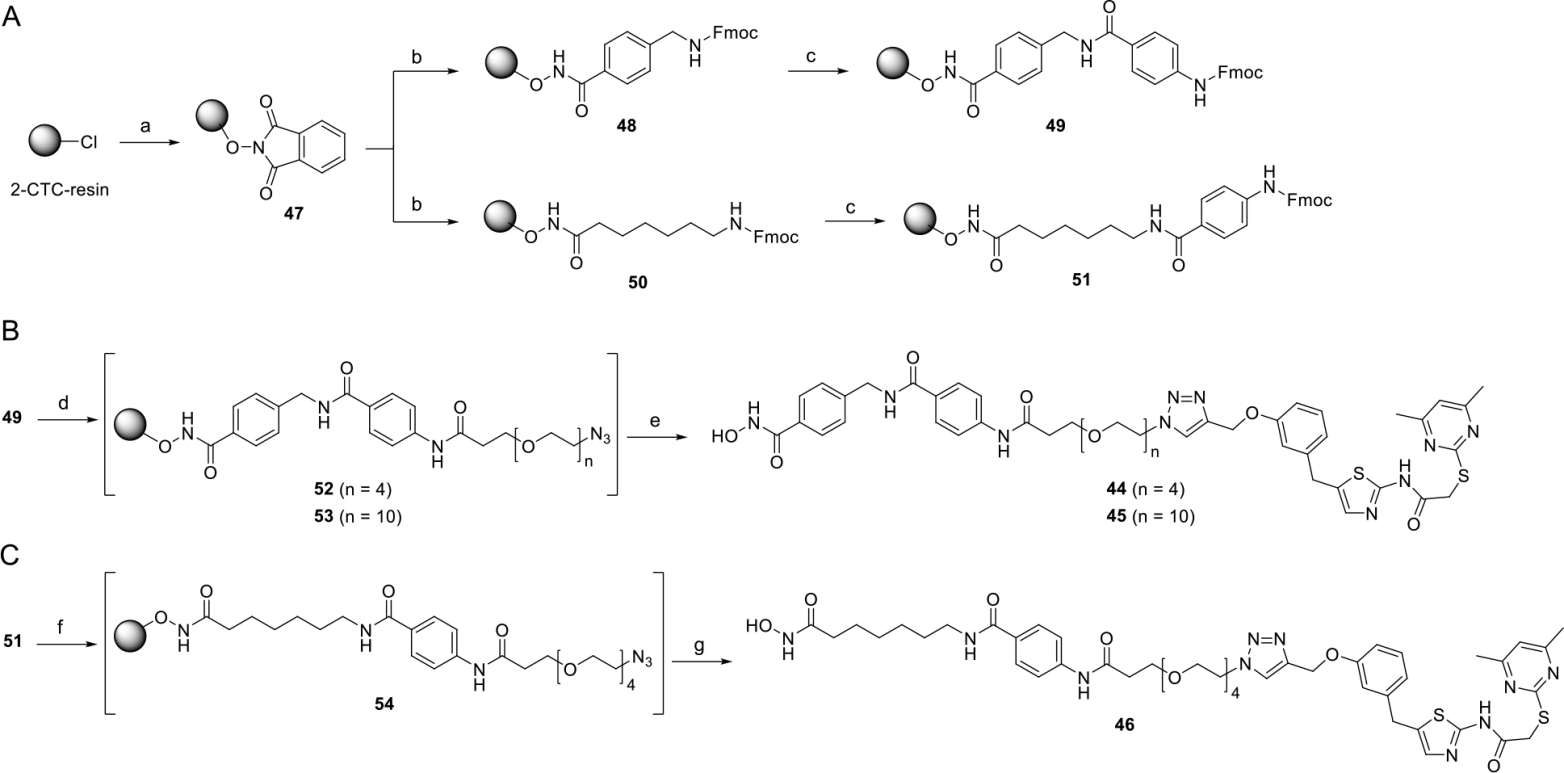
Solid-Phase-Supported Synthesis of Dual Sirt2/HDAC6 Inhibitors with Linked Pharmacophores (A) Solid-phase synthesis of the HDAC6 selective building block **49** as well the non-selective HDAC inhibiting subunit **51**, reagents and conditions: (a) PhthN-OH, Et_3_N, DMF, rt, 48 h; (b) 5% N_2_H_4_·H_2_O in MeOH, rt, 2 × 15 min, then carboxylic acid, HATU, HOBt·H_2_O, DIPEA, DMF, rt, 20 h, loading determined: 0.69 mmol/g for **48**, 0.77–0.97 mmol/g for **50**; (c) 20% piperidine in DMF, 2 × 5 min, then Fmoc-4-aminobenzoic acid, HATU, DIPEA, DMF, rt, 20 h. (B) Solid-phase synthesis of dual Sirt2/HDAC6 inhibitors **44** and **45**, reagents and conditions: (d) 20% piperidine in DMF, 2 × 5 min, then N_3_-PEG_*n*_-COOH, HATU, DIPEA, DMF, rt, 4 h; (e) **35**, TBTA, CuSO_4_·5H_2_O, ascorbic acid, DMF, *t*BuOH, rt, 18 h, then 5% TFA, CH_2_Cl_2_, rt, 1 h; overall yields 43% for **44**, 27% for **45**. (C) Solid-phase synthesis of the SAHA-derived multi-target Sirt2/HDAC inhibitor **46**, reagents and conditions: (f) 20% piperidine in DMF, 2 × 5 min, then N_3_-PEG_4_-COOH, HATU, DIPEA, DMF, rt, 4 h; (g) **33**, TBTA, CuSO_4_·5H_2_O, ascorbic acid, DMF, *t*BuOH, rt, 18 h, then 5% TFA, CH_2_Cl_2_, rt, 1 h; overall yield 43%.

For the synthesis of the dual Sirt2/HDAC6 inhibitors **44**–**46**, that feature longer polyethylene glycol (PEG)-based linkers between the two Sirt2- and HDAC6-targeted pharmacophores, we took advantage of previously published solid-phase-supported protocols for the synthesis of hydroxamic acids.^43,44^ Pioneering work with this respect was reported in 2006 by the group of David Fairlie.^43^ First, we immobilized hydroxylamine on commercially available 2-chlorotrityl chloride (2-CTC) resin. To this end, we treated the resin with *N*-hydroxyphthalimide and triethylamine. After deprotection of the phthaloyl (Phth) group of **47** using hydrazine hydrate, *N*-Fmoc-protected 4-(aminomethyl)benzoic acid for preferential HDAC6 inhibition or *N*-Fmoc-protected 7-aminoheptanoic acid aiming at nonselective HDAC inhibition was coupled to the functionalized resin, to obtain **48** and **50**, respectively. Another coupling cycle with *N*-Fmoc protected *para*-aminobenzoic acid provided the respective HDAC inhibitor precursors **49** and **51** (Scheme 3A). For the synthesis of the dual Sirt2/HDAC6 inhibitors **44** and **45** (Scheme 3B), we introduced PEG-linkers carrying an azide functionality at the aromatic amino group of **49**, which was released after Fmoc deprotection. The conjugation of the resin-bound and azido-functionalized HDAC6 inhibitors **52** or **53** with the alkynylated SirReal **35** was again enabled by Cu(I)-catalyzed Huisgen cycloaddition,^38,39^ thus showing that the solid-phase-supported synthesis of hydroxamic acids can be combined with click chemistry-based approaches. Finally, the dual Sirt2/HDAC6 inhibitors **44** and **45** were cleaved from the resin by treatment with trifluoroacetic acid (TFA). A highly similar procedure was applied for the synthesis of the SAHA-derived multitarget Sirt2/HDAC inhibitor **46** (Scheme 3C). Here, we started with the Fmoc deprotection of **51** followed by the installation of the PEG linker *via* amide coupling. A conjugation of the azido-functionalized amide **54** with the alkynylated SirReal **35** using click chemistry and subsequent cleavage with TFA resulted in the release of **46**.

#### Synthesis of HDAC6 Inhibitors As Control Compounds

**Scheme 4:**
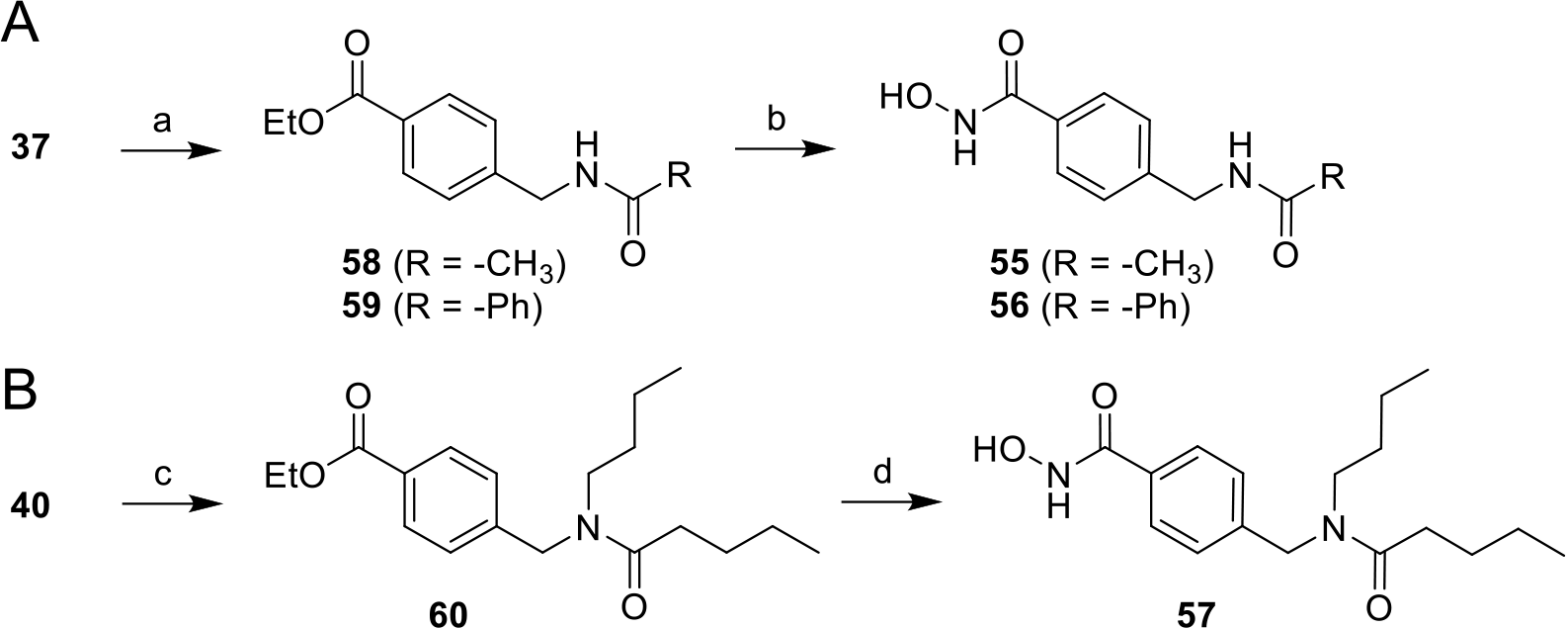
Synthesis of *N*-Hydroxybenzamide-Based HDAC6 Inhibitors as Control Compounds for the Biological Evaluation of Dual Sirt2/HDAC6 Inhibitors (A) Synthesis of **55** and **56**, reagents and conditions: (a) acyl chloride, DIPEA, CH_2_Cl_2_, 0–20 °C, 1 h, 72–86% yield; (b) H_2_NOH, CH_2_Cl_2_, MeOH, 0 °C, 10 min, then NaOH, 0–20 °C, 2.5 h, 35–40% yield. B) Synthesis of **57**, reagents and conditions: (c) pentanoyl chloride, pyridine, CH_2_Cl_2_, 0–20 °C, 4 h, 92% yield; (d) H_2_NOH, CH_2_Cl_2_, MeOH, 0 °C, 10 min, then NaOH, 0–20 °C, 3.5 h, 56% yield.

For the biological evaluation of the dual-targeted inhibitors, control compounds that selectively inhibit one of the two targeted proteins are highly important. The literature known SirReals **4** and **5** (Figure 1) were already available in our lab and were used as control compounds for potent and selective Sirt2 inhibition. The HDAC6 inhibitors **55**–**57** that reflect different HDAC6-targeting units of our dual Sirt2/HDAC6 inhibitors were synthesized according to Scheme 4. In the first step, the primary amine **37** (Scheme 4A) or secondary amine **40** (Scheme 4B) was acylated by a conversion with the respective acyl chloride to obtain the amides **58**–**60**. Then, a hydroxylaminolysis of the ethyl ester groups of **58**–**60** resulted in the hydroxamic acid-based HDAC6 inhibitors **55**–**57**.

### Biology

#### Biochemical *in Vitro* Assays

Our set of potential dual Sirt2/HDAC6 inhibitors were first tested on their potency and selectivity of target protein inhibition by using previously reported biochemical fluorescence-based deacetylation assays (Table 1).^44−46^ Whereas **21** and **22**, which feature a merged Sirt2/HDAC6-targeted pharmacophore, showed only weak Sirt2 inhibition, all dual Sirt2/HDAC6 inhibitors with linked pharmacophores (**31**–**33**, **44**–**45**) evoked both potent Sirt2 and HDAC6 inhibition. The fact that the dual Sirt2/HDAC6 inhibitors with linked pharmacophores exerted similar inhibition of Sirt2 and HDAC6 as compared to the control compounds for sole Sirt2 inhibition (**4** and **5**) or HDAC6 inhibition (**55**–**57**) corroborated the suitability of our design approach for these dual inhibitors. For **32** and **33**, we observed a selective inhibition of the targeted enzymes Sirt2 and HDAC6 compared to the off-target deacetylases Sirt1, Sirt3, and HDAC1. The Sirt2/HDAC6 on-target selectivity of **32** and **33** was further confirmed by inhibition tests with HDAC2 and HDAC3. Consistent with the data for HDAC1, **33** also showed weak off-target inhibition of HDAC2 and HDAC3, which might be a consequence of its bulky tertiary amide-based cap group of the HDAC6-targeted pharmacophore. This assumption is supported by the high HDAC6 selectivity of **57**, which is the HDAC6-targeted building block of **33**. As expected, the SAHA-derived multitarget Sirt2/HDAC inhibitor **46** showed low selectivity for Sirt2 and HDAC6. For this compound, we detected a more potent inhibition of HDAC1 (IC_50_ = 0.21 μM), HDAC2 (IC_50_ = 0.34 μM), and HDAC3 (IC_50_ = 0.14 μM) compared to Sirt2 inhibition (IC_50_ = 0.48 μM). To provide more comprehensive data regarding the sirtuin and HDAC selectivity of our lead structure **33**, we tested **33** for inhibition of HDAC4–5, HDAC7–10, and Sirt5–6 (see Supporting Information, Table S1). While for HDAC8 a weak off-target inhibition with an IC_50_ value of 2.94 ± 0.45 μM was detected, we observed even weaker activities for the other tested sirtuin and HDAC isotypes, thus further confirming the Sirt2/HDAC6 on-target selectivity of **33**.

**Table 1:** Dual Sirt2/HDAC6 Inhibitors Tested by Means of Previously Reported Biochemical *in Vitro* Deacetylation Assays^44−46^^a^

compd	Sirt2	HDAC6	Sirt1	Sirt3	HDAC1	HDAC2	HDAC3
**21**	11.5 ± 0.04	0.15 ± 0.01	ni^b^	ni^b^	2.1 ± 0.2	nt^e^	nt^e^
**22**	15% @ 10 μM	0.011 ± 0.001	ni^b^	ni^b^	0.21 ± 0.03	nt^e^	nt^e^
**31**	0.56 ± 0.27	0.025 ± 0.001	ni^b^	ni^b^	0.62 ± 0.06	nt^e^	nt^e^
**32**	0.15 ± 0.01	0.042 ± 0.007	ni^b^	17% @ 20 μM	1.2 ± 0.2	1.8 ± 0.1	0.71 ± 0.03
**33**	0.32 ± 0.11	0.043 ± 0.003	ni^b^	ni^b^	2.2 ± 0.2	6.0 ± 0.2	2.5 ± 0.2
**44**	0.48 ± 0.04	0.0096 ± 0.0003	ni^b^	ni^b^	0.60 ± 0.03	nt^e^	nt^e^
**45**	0.54 ± 0.01	0.017 ± 0.001	ni^b^	ni^b^	0.46 ± 0.05	nt^e^	nt^e^
**46**	0.48 ± 0.07	0.0050 ± 0.0005	ni^b^	ni^b^	0.21 ± 0.03	0.34 ± 0.02	0.14 ± 0.01
**4**	0.22 ± 0.01	18% @ 10 μM	ni^b^	ni^b^	10% @ 10 μM	nt^e^	nt^e^
**5**	0.12 ± 0.01^c^	ni^c^,^d^	ni^c^,^d^	ni^c^,^d^	ni^c^,^d^	nt^e^	nt^e^
**55**	nt^e^	0.65 ± 0.04	nt^e^	nt^e^	17 ± 1	nt^e^	nt^e^
**56**	nt^e^	0.018 ± 0.001	nt^e^	nt^e^	0.81 ± 0.05	nt^e^	nt^e^
**57**	ni^e^	0.032 ± 0.004	ni^b^	ni^b^	3.2 ± 0.2	4.7 ± 0.1	4.8 ± 0.4
SAHA	nt^e^	0.030 ± 0.008	nt^e^	nt^e^	0.12 ± 0.01	0.16 ± 0.01	0.11 ± 0.01

a IC_50_ values [μM, mean ± SD] or percentual inhibition at a given concentration of the dual Sirt2/HDAC6 inhibitors, as well as reference compounds for selective Sirt2 inhibition (**4**,**5**), HDAC6 inhibition (**55**–**57**), and SAHA as an nonselective inhibitor of Zn^2+^-dependent HDACs.

b ni = no inhibition (inhibition <15% @ 20 μM).

c Values for **5** taken from Vogelmann et al.^47^

d ni = no inhibition (IC_50_ > 100 μM).

e nt = not tested.

In addition to Sirt2-mediated deacetylation, several triazole-based SirReals, including **5**, were recently reported to inhibit Sirt2-catalyzed demyristoylation.^47^ Thus, we were interested to see whether our dual Sirt2/HDAC6 inhibitors also prevent Sirt2-catalyzed defatty acylation. Using a previously reported biochemical *in vitro* demyristoylation assay that is based on the small molecule myristoylated substrate ZMML,^47^ several of our triazole-based dual Sirt2/HDAC6 inhibitors were identified as low micromolar inhibitors of Sirt2-catalyzed demyristoylation (Table 2). Consistent with the data for Sirt2-mediated deacetylation (Table 1), the inhibitors with a merged Sirt2/HDAC6 pharmacophore (**21**,**22**) showed the weakest inhibition of Sirt2-mediated demyristoylation. To confirm the inhibition of Sirt2-mediated demyristoylation for our lead structure **33**, we also used a recently published biochemical activity assay that relies on the conversion of a peptide-based myristoylated substrate.^48^ With this assay, which is known to be very sensitive,^48^ we detected an IC_50_ value of 0.88 ± 0.09 μM (SI, Figure S1), thus corroborating the inhibition of Sirt2-mediated demyristoylation for our dual Sirt2/HDAC6 inhibitor **33**.

**Table 2:** Dual Sirt2/HDAC6 Inhibitors Tested by Means of a Biochemical *in Vitro* Demyristoylation Assay.^47^^a^

compd	Sirt2 (demyristoylation)
**21**	ni^b^
**22**	ni^b^
**31**	29 ± 1
**32**	1.7 ± 0.2
**33**	9.7 ± 1.3
**44**	34 ± 2
**45**	2.8 ± 0.6
**46**	13.2 ± 1.9
**4**	1.4 ± 0.3
**5**	2.5 ± 0.2^c^
**55**	ni^b^
**56**	ni^b^
**57**	<15% @ 5 μM
SAHA	18% @ 10 μM

a IC_50_ values [μΜ, mean ± SD] or percentual inhibition at a given concentration of the dual Sirt2/HDAC6 inhibitors, as well as reference compounds for selective Sirt2 inhibition (**4**,**5**), HDAC6 inhibition (**55**–**57**), and SAHA as an nonselective inhibitor of Zn^2+^-dependent HDACs.

b ni = no inhibition (inhibition <15% @ 20 μM).

c Values for **5** taken from Vogelmann et al.^47^

#### Co-crystal Structures of Sirt2 and HDAC6, Respectively, in Complex with Sirt2- or HDAC6-Targeted Subunits of Dual Sirt2/HDAC6 Inhibitors

**Figure 4:**
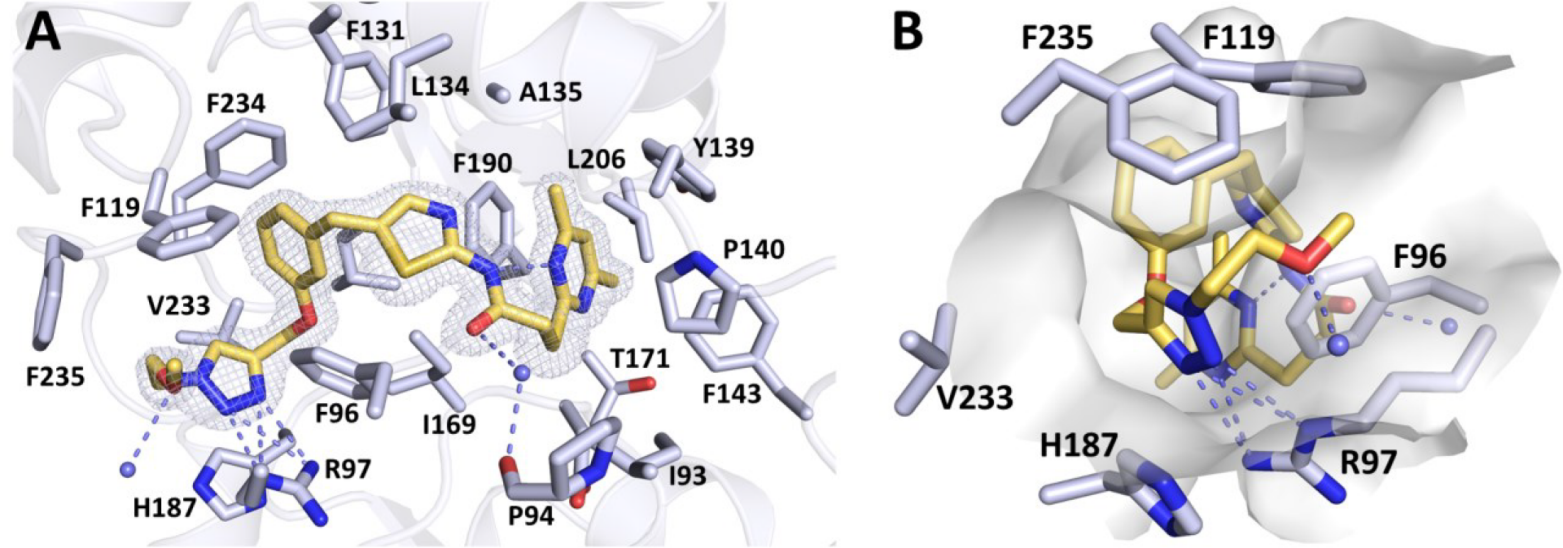
Crystal structure of the Sirt2–**5** complex. (A) **5** is shown as yellow-orange sticks. The 2*F*_o_ – *F*_c_ map is depicted as gray mesh and contoured at 1.0σ. Hydrogen bond interactions are shown as dashed slate lines and water molecules are depicted as small slate spheres. (B) Visualization of **5** binding in the acyl-lysine channel of Sirt2. The methoxyethyl moiety is exposed to the protein surface.

Encouraged by the *in vitro* activities and selectivity profiles of the dual Sirt2/HDAC6 inhibitors **32** as well as **33**, we set out to elucidate their binding modes to their vastly differing targets. As cocrystallization of **32** or **33** with both target proteins as well as the single target proteins did not yield suitable crystals for X-ray analyses, we were aiming for cocrystallizing Sirt2 and HDAC6, respectively, with the subunits of our dual inhibitors that target the binding site of the respective enzyme. We were able to cocrystallize Sirt2 in complex with the triazole-based SirReal1.2 (**5**, see Figure 4). The obtained cocrystal structure was solved at 1.65 Å resolution (*R*_free_ = 0.197, PDB 8OWZ; SI, Table S2) and provides an excellent basis to rationalize the Sirt2 binding mode of our dual Sirt2/HDAC6 inhibitors, as **5** is highly similar to the Sirt2-targeted subunit of **32** and **33**. The complex crystallized in space group *P*2_1_ with one monomer in the asymmetric unit. Compared to the published structure of Sirt2 in complex with a triazole-based SirReal1.1 (**4**, PDB 5DY5),^19^ no distinct conformational changes were observed for the main chain of the protein (root-mean-square deviation (RMSD) of 0.401 Å). The observed binding mode of **5** is highly consistent with the reported binding mode of **4**.^19^ The dimethyl pyrimidine is tightly anchored in the selectivity pocket by hydrophobic amino acids Tyr139, Phe143, Phe190, and Leu206. A conserved water in the active site forms a hydrogen bond network with C=O of **5** and the main chain of Pro94. The typical triazole-based SirReal hydrogen bonds are formed with Arg97 contributing to the affinity of **5**. Additionally, the well resolved electron density for the normally flexible methoxyethyl moiety protruding from the acyl-lysine channel indicates that the water mediated hydrogen bond of 3.0 Å distance stabilizes the conformation in the crystal structure. This water mediated hydrogen bond also provides a possible explanation for the slightly improved potency of **5** (IC_50_ = 0.12 μM) compared to the benzyl analogue **4** (IC_50_ = 0.16 μM).^19^

**Figure 5:**
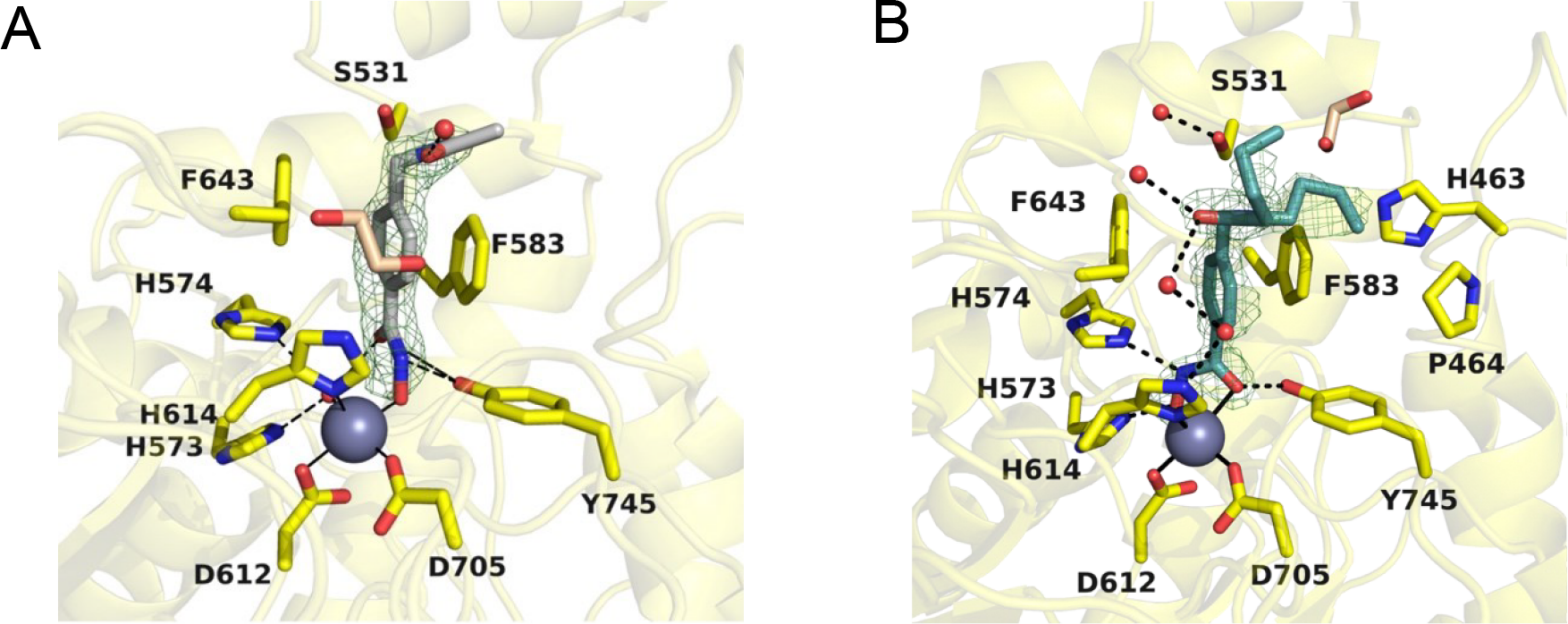
Polder omit map (contoured at 4.0σ) showing (A) the monodendate Zn^2+^-binding mode of **55** (light-gray sticks) in the active site of HDAC6 (yellow, PDB 8G1Z) and (B) the bidendate binding mode of **57** (teal sticks) in the active site of HDAC6 (yellow, chain A, PDB 8G20). The catalytic Zn^2+^ ion is shown as a gray sphere with metal coordination shown as solid black lines. Water molecules are shown as small red spheres and ethylene glycol molecules are shown as tan sticks. Hydrogen bonds are depicted as dashed black lines.

To obtain structural insights into the HDAC6 binding modes of the dual Sirt2/HDAC6 inhibitors **32** and **33**, we submitted **55** and **57**, which represent the HDAC6-targeted subunits of **32** and **33**, respectively, for crystallization trials with HDAC6. Crystal structures of catalytic domain 2 (CD2) of *Danio rerio* (zebrafish) HDAC6 complexed with **55** or **57** were determined at resolutions of 1.87 Å (*R*_free_ = 0.254, PDB 8G1Z; Figure 5A, SI, Table S2) and 1.77 Å (*R*_free_ = 0.229, PDB 8G20; Figure 5B, SI, Table S2), respectively. As the crystal structures of human and zebrafish CD2 enzymes are essentially identical,^49^ the zebrafish HDAC6 CD2 (henceforth simply “HDAC6”) serves as a more readily studied surrogate of the human enzyme. The HDAC6–**55** complex crystallized in space group *P*2_1_ with one monomer in the asymmetric unit. No significant conformational changes are observed between the structures of the inhibitor-bound and unliganded (PDB 5EEM) HDAC6 structures, and the RMSD is 0.15 Å for the 328 Cα atoms. The hydroxamate N–O^–^ group coordinates to the catalytic Zn^2+^ ion with a Zn^2+^–O separation of 2.0 Å. The hydroxamate C=O group accepts a hydrogen bond from the Zn^2+^-bound water molecule, which also forms hydrogen bonds with the imidazole side chains of H573 and H574. Additionally, the phenolic hydroxyl group of Y745 forms poorly oriented hydrogen bonds with the hydroxamate C=O and NH groups. The phenyl subunit of **55** is sandwiched between F583 and F643, making staggered π-stacking interactions. The carbonyl of the inhibitor capping group accepts a hydrogen bond from a nearby water molecule and is positioned 3.6 Å away from a molecule of ethylene glycol. The acetamide NH group of the inhibitor capping group is 3.7 Å from the side chain of S531 and the acetamide methyl group is oriented toward solvent, although electron density coverage is poor in this region of the electron density map (Figure 5A).

The HDAC6–**57** complex crystallized in space group *P*1 with two monomers in the asymmetric unit. The inhibitor binding mode is essentially identical in monomers A and B. Inhibitor binding does not trigger any significant structural rearrangements, as reflected by an RMSD of 0.15 Å (315 Cα atoms) between the structures of inhibitor-bound and unliganded HDAC6 (PDB 5EEM). The hydroxamate moiety of **57** coordinates to the catalytic Zn^2+^ ion with bidendate geometry; the hydroxamate N–O^–^ and C=O groups exhibit Zn^2+^–O separations of 2.0 and 2.4 Å, respectively. The Zn^2+^-bound N–O^–^ group accepts a hydrogen bond from H573 and the Zn^2+^-bound C=O group accepts a hydrogen bond from Y745; the hydroxamate NH group donates a hydrogen bond to H574. The phenyl subunit of **57** is positioned in the aromatic crevice formed by F583 and F643, where it makes staggered π-stacking interactions similar to those observed for **55**. The peptoid carbonyl group participates in a water-mediated hydrogen bond network with H614.

#### Modeling of the Sirt2 and HDAC6 Binding Modes of Dual Sirt2/HDAC6 Inhibitors

**Figure 6:**
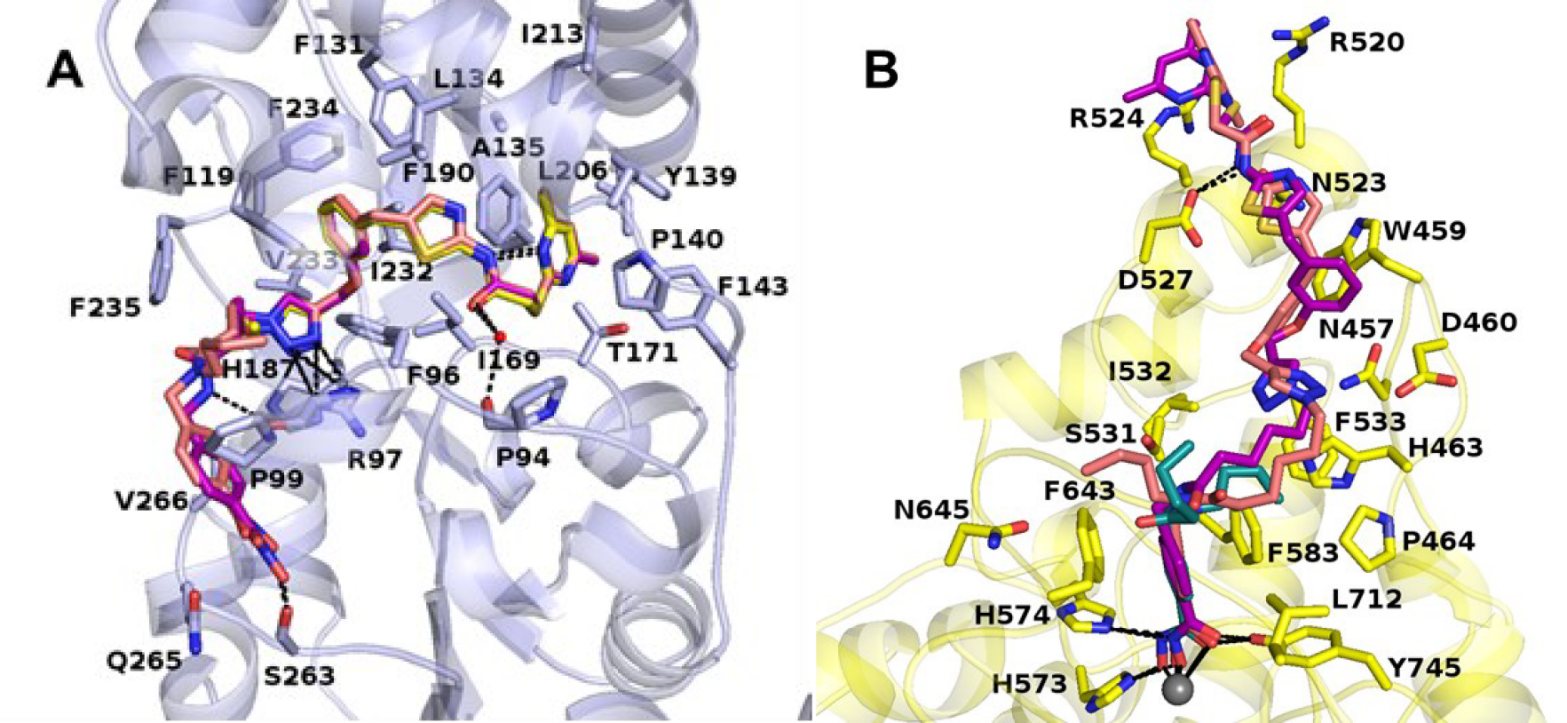
Dual Sirt2/HDAC6 inhibitors **32** and **33** are predicted to bind to their target proteins in a similar manner as observed for their cocrystallized unconjugated building blocks. (A) Predicted Sirt2 binding modes of **32** (purple sticks) and **33** (salmon sticks) overlaid with the observed binding mode of the Sirt2-targeted **5** (yellow sticks, Sirt2 in light blue, PDB 8OWZ). (B) Predicted HDAC6 binding modes of **32** (purple sticks) and **33** (salmon sticks) overlaid with the observed binding mode of the HDAC6-targeted **57** (teal sticks, HDAC6 in yellow, PDB 8G20). The catalytic Zn^2+^ ion is shown as a gray sphere with metal coordination shown as orange dashed lines. Water molecules are shown as small red spheres. Hydrogen bonds are depicted as black dashed lines.

To rationalize the determined *in vitro* results, we performed docking studies using the solved crystal structures of HDAC6-**57** (PDB 8G20) and Sirt2-**5** (PDB 8OWZ). Results from docking studies using the monodentate binding mode as observed in the HDAC6-**55** cocrystal structure (PDB 8G1Z) are not shown because our docking studies strongly supported a bidentate Zn^2+^-binding for our *N*-hydroxybenzamide-based dual Sirt2/HDAC6 inhibitors. The goodness of the docking setup was first tested on the solved crystal structures and showed very good agreement between docking solutions and crystal structures (details in the Experimental Section). Docking of the dual-targeting inhibitors **32** and **33** showed that both the HDAC6 inhibitor part and the Sirt2 inhibitor part bind to the corresponding target in an analogous way as observed for the parent compounds in the crystal structures (Figure 6). In the case of Sirt2, **32** and **33** show the same H-bridges as **5** (conserved water at Pro94 and Arg97). The *N*-hydroxybenzamide residue shows van der Waals interactions with Pro99 and His187, while the hydroxamate is involved in an H-bridge with Ser263 (Figure 6A). **32** shows a further hydrogen bond between the unsubstituted amide group and the backbone of Arg97, which might contribute to the stronger Sirt2 inhibition compared to **33**. For HDAC6, which has a smaller binding pocket, a large portion of the inhibitor moiety lies at the surface of the protein. Only the HDAC6 inhibitor part that is analogue to the **57** structure is completely buried in the binding pocket (Figure 6B). The terminal dimethylpyrimidine ring interacts with the polar residues Arg520, Arg524, and Asp527 by π-aromatic interaction and the inhibitor amide is involved in a H-bridge with Asp527.

To analyze the stability of the obtained docking solutions of **32** and **33** for HDAC6 and Sirt2, molecular dynamics (MD) simulations were performed. First, we tested the MD protocol on the solved crystal structures of Sirt2 and HDAC6. In both cases, stable complexes with low RMSD values were observed in two independent 100 ns MD runs (SI, Figure S2–S3). Both protein conformation and ligand interaction exhibited little variation during MD simulations. In the case of the **32** and **33**–Sirt2 docking complex, MD simulations showed stable protein conformations as well as only minor fluctuations of the Sirt2 inhibitor moiety within the binding pocket (SI, Figure S4–S5). The intramolecular H-bridge as well as the H-bridges between the carbonyl group of the inhibitor and the conserved water molecule were preserved. The HDAC6 targeting group (i.e., *N*-hydroxybenzamide) located at the entrance of the binding pocket showed a higher fluctuation (SI, Figure S6). In the case of the simulated HDAC6–**32** and **33** complexes, the protein and zinc coordination of the inhibitor remained stable. The Sirt2 inhibitor part as well as the interacting amino acid residues at the surface of the protein showed slight rearrangement but resulted in a stabilized complex after 10 ns in both simulations (SI, Figure S7–S9). In summary the docking and MD simulations showed that the dual-targeting inhibitors **32** and **33** interact with Sirt2 and HDAC6 similarly to the original inhibitors. For both targets, interactions of the additional inhibitor moiety have also been found but showed higher flexibility in the MD simulations due to their partial solvent exposure.

#### Cell-Based Studies

**Figure 7:**
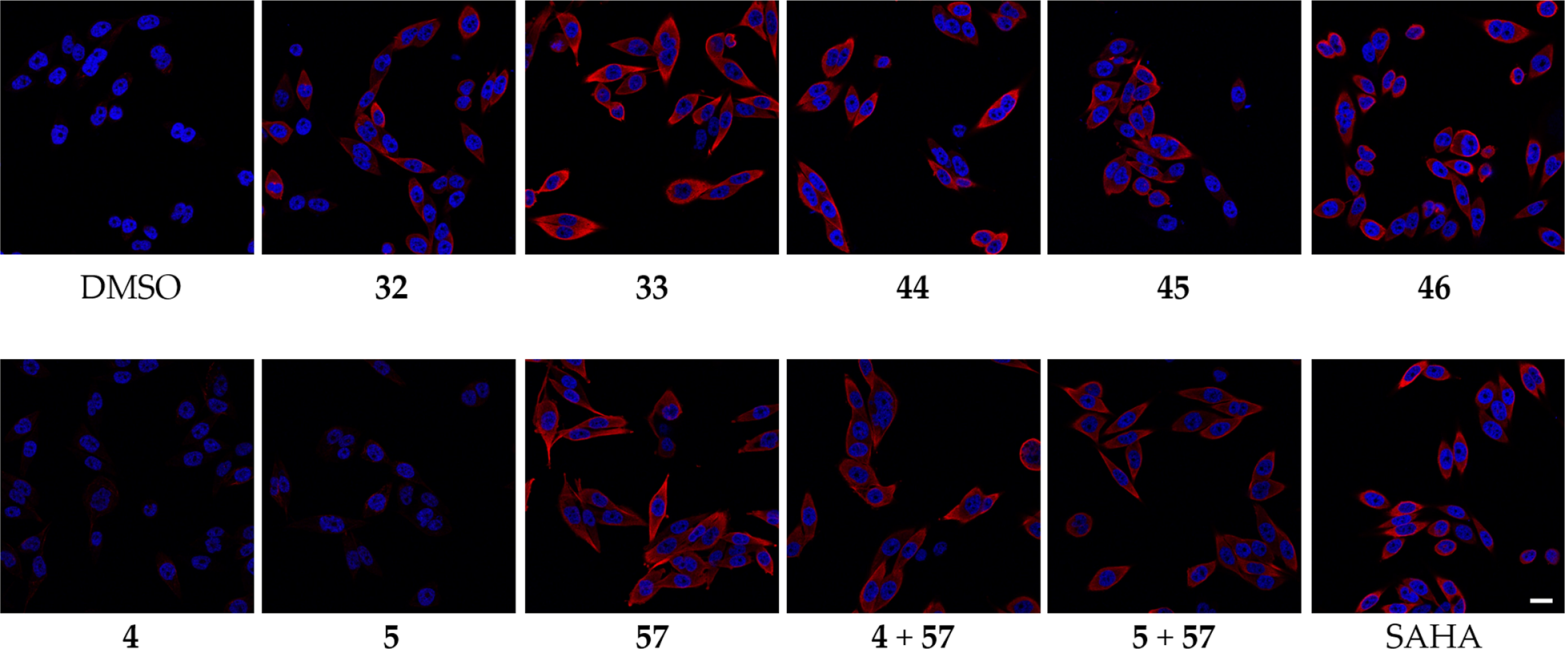
Dual Sirt2/HDAC6 inhibitors increase α-tubulin acetylation levels in PC-3M-luc prostate cancer cells. Cells were treated with 20 μM of Sirt2 inhibitor or DMSO (vehicle) for 5 h before imaging. Representative images (*n* = 4) show acetylation levels of α-tubulin in red and the DAPI-stained nuclei in blue. Scale bar represents 24 μm.

After having shown that several of our dual Sirt2/HDAC6 inhibitors potently block Sirt2- as well as HDAC6-catalyzed deacetylation under cell-free conditions, we now were curious whether these compounds also inhibit the targeted enzymes in a cellular environment. In general, the transition from a cell-free to a cellular environment is a critical step in preclinical drug discovery, as cellular on-target activity of small molecules can be changed significantly due to various factors, including off-target binding, changes in target protein structure/accessibility, and limited cell permeability. The latter is especially relevant for dual inhibitors with linked pharmacophores, because they are not typical drug-like molecules, as a consequence of their high molecular weight (>500 Da), potentially resulting in impaired membrane permeability. Because our dual Sirt2/HDAC6 inhibitors were developed as tools for dual inhibition of tubulin deacetylation, and investigating tubulin acetylation is one of the most commonly used methods to prove cellular activity for both Sirt2 and HDAC6 inhibitors,^50^ we first used this method to evaluate the cellular effects of our synthesized compounds. Based on the previously obtained *in vitro* results (Table 1), we focused on **32** and **33** and **44**–**46** for further cellular characterization. In PC-3M-luc prostate cancer cells, an established cell line for visualizing the cellular effects of tubulin deacetylase inhibitors via immunofluorescence microscopy,^47^ we were able to show that all tested dual Sirt2/HDAC6 inhibitors induced a hyperacetylation of the tubulin network (Figure 7).

**Figure 8:**
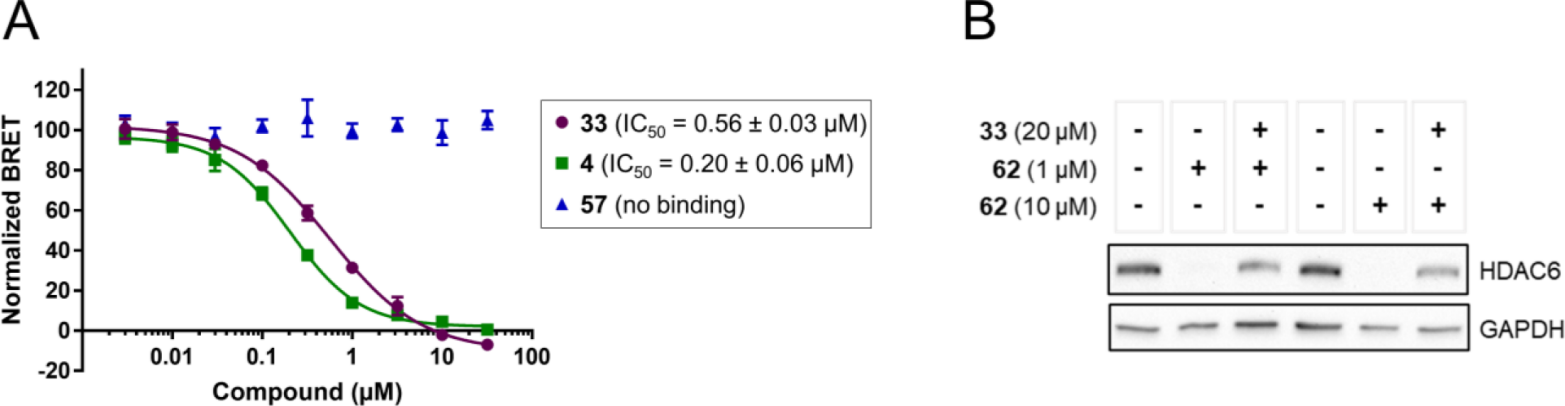
Cellular target engagement studies for the dual Sirt2/HDAC6 inhibitor **33**. (A) Cellular Sirt2 target engagement studies. Representative NanoBRET assay curves displaying the relative Sirt2 affinity of our dual Sirt2/HDAC6 inhibitor **33** in HEK293T cells. **4** and **57** were used as reference compounds for selective Sirt2 and HDAC6 inhibition, respectively. (B) Cellular HDAC6 target engagement studies. MCF-7 cells were treated with vehicle (DMSO) or the selective HDAC6 degrader B4 (**62**)^52^ (1 or 10 μM) for 6 h. For the cotreatment groups, MCF-7 cells were pretreated with **33** (20 μM) for 30 min and followed by treatment with **62** (1 μM) or **62** (10 μM) for additional 6 h. GAPDH was used as loading control. Representative images of *n* = 3 replicates.

This indicates that, despite their high molecular weight, our dual Sirt2/HDAC6 inhibitors are in fact able to pass the cell membranes of PC-3M-luc cells and bind to and inhibit intracellular tubulin deacetylases. In the case of **33**, the increase in tubulin hyperacetylation was especially pronounced. Control experiments with the selective Sirt2 inhibitors **4** or **5**, as well as the HDAC6 selective **57**, suggest that dual Sirt2/HDAC6 inhibition by means of **33** might have a stronger effect on tubulin acetylation compared to single or combination treatment with the unconjugated Sirt2- and HDAC6-targeted inhibitors. As the observed effects of selective Sirt2 inhibition by single treatment with **4** or **5** were very minor and much less pronounced compared to selective HDAC6 inhibition by **57**, we utilized a recently published NanoBRET assay,^47^ in order to verify that the dual Sirt2/HDAC6 inhibitor **33** really binds to Sirt2 on a cellular level. Using this setup for cellular Sirt2 target engagement that is based on the fluorescent probe SirReal-TAMRA (**61**, for chemical structure see SI, Figure S10A),^51^ we detected an IC_50_ value of 0.56 μM for **33**, which is very similar to the IC_50_ value of 0.32 μM determined under cell-free conditions (Figure 8A). In control experiments, the selective Sirt2 inhibitor **4** showed a potent interaction with Sirt2 (IC_50_ = 0.20 μM), whereas the selective HDAC6 inhibitor **57**, which was used as a negative control, showed no Sirt2 binding. To show cellular HDAC6 target engagement for **33**, we performed a degradation rescue experiment, by applying the recently reported selective HDAC6-targeted PROTAC B4 (**62**, for chemical structure, see SI, Figure S10B).^52^ In MCF-7 breast cancer cells that show an upregulated HDAC6 expression,^53^**33** is able to rescue HDAC6 from proteasomal degradation induced by **62**, thus clearly indicating cellular HDAC6 target engagement for **33** (Figure 8B). In summary, our results from cellular target engagement studies suggest that **33** can induce dual inhibition of the tubulin deacetylases Sirt2 and HDAC6 on a cellular level.

#### Cell Viability Assays

As both Sirt2 and HDAC6 are involved in the pathogenesis of cancer,^4,6−11^ but single treatment with either selective Sirt2 or HDAC6 inhibitors evokes only low to moderate effects on cell viability,^9,47,54^ we were interested to see whether a dual Sirt2/HDAC6 inhibition might result in improved effects compared to single or combination treatment with Sirt2 and HDAC6 inhibitors. Thus, we tested our dual Sirt2/HDAC6 inhibitors against a panel of solid cancer cell lines of different chemosensitivity and tissue origin, including HGC27 gastric carcinoma cells, W1 ovarian cancer cells, MCF-7 breast cancer cells, and PC-3M-luc prostate cancer cells (Table 3). All selected cell lines were isolated from tumor types that were already investigated regarding the effects of Sirt2 and HDAC6 inhibition, respectively.^9,47,55−58^ Whereas most of the tested dual Sirt2/HDAC6 inhibitors showed no effects on cell viability, compound **33** reduced the viability of all tested cancer cell lines. These results are consistent with the data from our cell-based studies investigating α-tubulin acetylation (Figure 7), where we also detected the strongest effects for this dual Sirt2/HDAC6 inhibitor. The fact that the closely related dual agents **32** and **33** show different cellular effects (cancer cell viability, tubulin acetylation), despite evoking similar *in vitro* activities, might be a consequence of conformational effects caused by the additional *N*-butyl group in **33**. The ^13^C spectra of **33** clearly supports that this compound can exist both as a *cis* and *trans* amide bond rotamer, whereas the ^13^C spectra of **32** indicates the existence of only one rotamer (i.e., *trans*-amide). By influencing intramolecular interactions, conformational effects can have a massive impact on physicochemical properties, cell permeability, and eventually cellular effects. This is especially relevant for bifunctional molecules such as dual inhibitors or PROTACs, which lie within a chemical space where ADME properties are often complicated.^59^ Western blot experiments investigating cellular histone H3 acetylation (a marker of reduced HDAC1–3 activity) supported that the effects of **33** on cancer cell viability are not caused by an off-target inhibition of class I HDACs (SI, Figure S11). Interestingly, for two of the four tested cancer cell lines (i.e., HGC27, W1), we observed an improved activity of the dual Sirt2/HDAC6 inhibitor **33** compared to single or combination treatment with Sirt2 and HDAC6 inhibitors. For the cancer cell line W1, the improvement in activity of the dual Sirt2/HDAC6 inhibitor **33** was statistically significant (*p* = 0.007), compared to a combination treatment with the selective Sirt2 inhibitor **4** and the selective HDAC6 inhibitor **57**. However, for the interpretation of these data it should be noted that cell viability is primarily impacted by HDAC6 inhibition, the additional effects of Sirt2 inhibition are comparably minor, and an off-target inhibition of other proteins outside the family of lysine deacylases cannot be ruled out.^60^

**Table 3:** Results from Cell Viability Assays with Dual Sirt2/HDAC6 Inhibitors^a^

compd	HGC27	W1	MCF-7	PC-3M-luc
**32**	ni^b^	ni^b^	ni^b^	ni^b^
**33**	12.9 ± 0.9 (ns)	19.2 ± 1.3**(*p* = 0.007)	21.1 ± 6.7 (ns)	30.1 ± 3.8 (ns)
**44**	ni^b^	ni^b^	ni^b^	ni^b^
**45**	ni^b^	ni^b^	ni^b^	ni^b^
**46**	17.2 ± 1.0	ni^b^	ni^b^	ni^b^
**4**	39% @ 50 μM	3% @ 0.1 mM, 22% @ 1 mM, 57% @ 3.2 mM	3% @ 0.1 mM, 13% @ 1 mM, 43% @ 3.2 mM	ni^b^
**57**	21.1 ± 1.6	33.6 ± 4.1	16.3 ± 5.9	30.1 ± 2.3
**4** + **57**	15.1 ± 1.3	34.1 ± 4.6	18.0 ± 3.8	28.3 ± 2.4

a The effects on cell viability are presented as EC_50_ (mean ± SD) or percentual inhibition at a given concentration. EC_50_ values were obtained from at least two independent experiments performed in triplicate. **4** and **57** were used as reference compounds for selective Sirt2 inhibition and HDAC6 inhibition, respectively. When combined, **4** and **57** were applied at identical concentrations. Statistics (t-test): **p ≤ 0.01, *p ≤ 0.05, ns > 0.05, values for **33** compared to combination treatment with **4** and **57**.

b ni = no inhibition (cellular effect <20% @ 50 μM).

## Conclusions

In order to provide a molecular tool for a dual inhibition of the two tubulin deacetylases Sirt2 and HDAC6, we either merged or linked the pharmacophores of selective Sirt2 and HDAC6 inhibitors. For the synthesis of these dual Sirt2/HDAC6 inhibitors, we used both solution-phase and solid-phase chemistry. Our PEG-linker-based dual Sirt2/HDAC6 inhibitors were synthesized by means of solid-phase support. In the course of these syntheses, we showed that hydroxamic acids immobilized on resin can be functionalized with click chemistry-based approaches. In biochemical *in vitro* assays, we identified **33** as a potent and selective inhibitor of both target enzymes. In addition to deacetylation, several of our dual Sirt2/HDAC6 inhibitors, including **33**, are also able to inhibit Sirt2-catalyzed lysine defatty acylation. Applying a diverse set of cell-based methods, including NanoBRET, PROTAC rescue, and immunofluorescence microscopy, we demonstrated that **33** is indeed able to bind to and inhibit the two tubulin deacetylases Sirt2 and HDAC6 in a cellular environment. New cocrystal structures of Sirt2 and HDAC6, respectively, in complex with the Sirt2- or HDAC6-targeted subunits of **33** enabled the modeling of the binary Sirt2–**33** and HDAC6–**33** complexes, thereby indicating that **33** can bind to both target proteins in a similar manner as observed for the unconjugated Sirt2 and HDAC6 inhibitors. In W1 ovarian cancer cells, **33** evoked enhanced effects on cell viability compared to single or combination treatment with the unconjugated Sirt2 and HDAC6 inhibitors. Thus, our dual Sirt2/HDAC6 inhibitors are highly interesting new tools to investigate the consequences and the therapeutic potential of dual inhibition of the two tubulin deacetylases Sirt2 and HDAC6.

## Experimental Section

### Materials and Methods

Starting materials (chemicals) were purchased from commercial suppliers (Abcr, Acros Organics, Alfa Aesar, BLDpharm, Sigma-Aldrich, TCI) and used without any further purification. The alkynylated **35**,^19^ the triazole-based SirReals **4** and ,^19^ the Sirt2-targeted fluorescent probe SirReal-TAMRA (**61**),^51^ and the HDAC6-targeted PROTAC B4 (**62**) were synthesized as previously reported.^52^ Solvents were used in pa quality and dried according to common procedures, if necessary. Thin-layer chromatography (TLC) for reaction monitoring was performed with alumina plates coated with Merck silica gel 60 F254 (layer thickness: 0.2 mm) or Merck silica gel 60 RP-18 F254 (layer thickness: 0.2 mm) and analyzed under UV-light (254 nm). As an alternative method for reaction monitoring, we used high performance liquid chromatography mass spectrometry (HPLC-MS). HPLC-MS analyses were performed using a Thermo Scientific Dionex UltiMate 3000 HPLC system in combination with a DAD detector (220/230/254 nm) and an Agilent ZORBAX ECLIPSE, and XDB-C8 column (3.0 mm × 100 mm, 3.5 μm). Elution was performed at room temperature under gradient conditions. Eluent A was water containing 0.1% (v/v) formic acid; eluent B was methanol. Linear gradient conditions were as follows: 0–0.2 min: A = 75%, B = 25%; 0.2–6.0 min: linear increase to B = 100%; 6.0–8.5 min: B = 100%; 8.5–9.0 min: linear decrease to A = 75%, B = 25%; 9.0–12.0 min: A = 75%, B = 25%. A flow rate of 0.4 mL·min^–1^ was maintained during the entire elution. Mass detection was performed with a BRUKER amaZon SL mass spectrometer using ESI as ionization source. Flash column chromatography was performed with hand packed Silica Columns 60 M (0.040–0.063 μm, 230–400 mesh) as a stationary phase on a Biotage SP-1 or Selekt automated flash purification system with UV–vis detector. Yields were not optimized. NMR spectra were recorded using either a Bruker Avance 400 (^1^H: 400 MHz; ^13^C: 101 MHz), Bruker Avance 600 (^1^H: 600 MHz; ^13^C: 151 MHz), Bruker Avance III HD 400 (^1^H: 400 MHz; ^13^C: 101 MHz), Varian/Agilent Mecury-plus-400 (^1^H: 400 MHz; ^13^C: 101 MHz), or Varian/Agilent Mecury-plus-300 (^1^H: 300 MHz; ^13^C: 75 MHz) instrument. The spectra are referenced against the NMR solvent and are reported as follows: ^1^H, chemical shift δ (ppm), multiplicity (s = singlet, d = doublet, dd = doublet of doublets, t = triplet, m = multiplet, b = broad), integration, coupling constant (*J* in Hz). ^13^C: chemical shift δ (ppm), abbreviations: carbons that could not be found in ^13^C spectra (DEPTQ) but in HMBC or HSQC are additionally marked with a hashtag (#). Signals that are partially overlaid by a solvent signal are marked with an asterisk (*). The assignment resulted from HMBC and HSQC experiments. High resolution mass spectra were either measured with a timsTOF Pro Mass Spectrometer from Bruker Daltonics using ESI as ionization source, a Bruker Daltonics micrOTOF coupled to a LC Packings Ultimate HPLC system and controlled by micrOTOFControl3.4 and HyStar 3.2-LC/MS, or a Bruker Daltonics ESI-qTOF Impact II coupled to a Dionex UltiMateTM 3000 UHPLC system and controlled by micrOTOFControl 4.0 and HyStar 3.2-LC/MS. Purity was determined for all tested compounds by HPLC and UV detection and was >95%. HPLC analyses for compounds **21** and **22**, **31**–**33**, and **55**–**57** were performed using an Agilent 1200 series HPLC system employing a diode array detector (DAD, detection at 200, 220, 254, or 560 nm). If not stated otherwise, the indicated purity was determined at a wavelength of 254 nm. For method 1 (M1), we used a ZORBAX ECLIPSE, XDB-C8 column (4.6 mm × 150 mm, 5 μm) with a flow rate of 0.5 mL·min^–1^. Elution was performed at room temperature under gradient conditions. Eluent A was water containing 0.1% (v/v) TFA; eluent B was acetonitrile. Linear gradient conditions were as follows: 0–3.0 min: A = 90%, B = 10%; 3.0–18.0 min: linear increase to A = 5%, B = 95%; 18.0–24.0 min: A = 5%, B = 95%; 24.0–27.0 min: linear decrease to A = 90%, B = 10%; 27.0–30.0 min: A = 90%, B = 10%. For method 2 (M2), we used a NUCLEODUR C18 Pyramid column (4.6 mm × 250 mm, 5 μm) with a flow rate of 0.5 mL·min^–1^: Elution was performed at room temperature under gradient conditions. Eluent A was water containing 0.3% (v/v) formic acid; eluent B was acetonitrile. Linear gradient conditions were as follows: 0–3.0 min: A = 90%, B = 10%; 3.0–18.0 min: linear increase to B = 100%; 18.0–24.0 min: B = 100%; 24.0–27.0 min: linear decrease to A = 90%, B = 10%; 27.0–30.0 min: A = 90%, B = 10%. HPLC analyses for compounds **44**–**46** were performed using method 3 (M3). Here, either a Thermo Fisher Scientific UltiMateTM 3000 UHPLC system or a Gynkotek Gina 50 HPLC system (Detector: Gynkotek UVD340U, Pump: Dionex P680 HPLC pump, column oven: Dionex STH 585) with a Nucleodur 5 μm, C18 100 Å (250 mm × 4.6 mm, Macherey Nagel) column were used. In the process a flow rate of 1 mL·min^–1^ and a temperature of 25 °C were set. Detection was implemented by UV absorption measurement at a wavelength of λ = 254 nm. Elution was performed under gradient conditions. Eluent A was water containing 0.1% (v/v) TFA; eluent B was acetonitrile containing 0.1% (v/v) TFA. Linear gradient conditions were as follows: 0–5.0 min: A = 95%, B = 5%; 5.0–20.0 min: linear increase to A = 5%, B = 95%; 20.0–25.0 min: A = 5%, B = 95%.

### General Synthetic Methods for Solid-Phase Chemistry

For all syntheses carried out on solid-phase, 2-chlorotrityl chloride resin (200–400 mesh, 1.1–1.8 mmol/g, Iris Biotech) was used. The manual solid-phase synthesis was carried out in PP-reactors with PE frit (sizes: 2/10/20 mL, pore size 25 μm, MultiSynTech GmbH). Syntheses carried out after the modification of the resin and determination of the loading followed the standard Fmoc solid-phase method. In brief, after resin swelling for 30 min in DMF, the standard procedure was carried out by repeating the Fmoc-deprotection and amide coupling. Completion of each coupling step was monitored TNBS test using a TNBS test kit supplied by TCI. After the last reaction cycle was performed, the final compounds were cleaved from the resin using the standard cleavage cocktail (5% TFA/95% CH_2_Cl_2_ (v/v), treatment for 1 h at ambient temperature) and the compounds were purified by preparative RP-HPLC. Fractions containing the desired final compounds were collected and lyophilized yielding the dual- or multitarget inhibitors 41–43 with >95% purity in all cases.

#### TNBS Test

A little amount of resin-beads was placed in a 0.5 mL microcentrifuge tube. One drop of picrylsulfonic acid (∼1% in DMF) and two drops of DIPEA (10% in DMF) were added to the resin beads. The test was evaluated after 5 min reaction time at room temperature.

#### Test Cleavage from the Resin

For a test cleavage, about 2 mg of the dried resin was placed into a tube and treated with the standard cleavage solution for 1 h. After the reaction period was finished, the filtrate was collected and the solvent was removed *in vacuo* giving the crude products, which were dissolved in Milli-Q H_2_O/acetonitrile for analytical purposes via HPLC. A cleavage on a larger scale was carried out in the same way. For each 40 mg of resin 1.0 mL of cleavage cocktail was used.

#### Determination of the Resin Loading

A small part of the different preloaded resin (∼5 mg) was treated with 500 μL of the deprotection solution (20% piperidine in DMF) for 5 min. The filtrate was collected, and the procedure was repeated once. The absorbance of the combined filtrates was measured at a wavelength of 300 nm and the concentration was determined photometrically (ε_300 nm_(dibenzofulvene) = 7800 M^–1^ cm^–1^). With the concentration of the cleaved dibenzofulvene calculated by using Lambert–Beer law and the mass of the resin, the loading could be determined. The full synthetic procedures are provided along with the procedures for the synthesis of the individual compounds (see below).

### Individual Synthetic Methods

#### 3-((2-(2-((4,6-Dimethylpyrimidin-2-yl)thio)acetamido)thiazol-5-yl)methyl)-*N*-hydroxybenzamide (**21**)

Ethyl 3-((2-(2-((4,6-dimethylpyrimidin-2-yl)thio)acetamido)thiazol-5-yl)methyl)benzoate (**29**, 52.4 mg, 118 μmol, 1 equiv) was dissolved in a mixture of 0.35 mL of dichloromethane and 0.71 mL methanol and cooled on an ice bath to 0 °C. Then 220 μL (234 mg, 50%: 117 mg, 3.55 mmol, 30 equiv) of an aqueous 50 wt % hydroxylamine solution were added and the mixture was stirred at 0 °C for 10 min before 47.3 mg (1.18 mmol, 10 equiv) sodium hydroxide were added. After 30 min at 0 °C, the ice bath was removed and stirring of the colorless clear solution was continued at ambient temperature for 2 h. The reaction dried under reduced pressure and the residue was taken up in 2.5 mL of deionized water. The suspension was cooled in an ice bath and brought to pH = 8 by the dropwise addition of 1 M HCl, when large amounts of white solid formed. The solid was collected by centrifugation. The precipitate was washed with water (2 × 1 mL) and ice-cold diethyl ether (2 mL) and dried under reduced pressure. For the final purification, preparative HPLC (gradient elution from 25% to 50% acetonitrile/water (+ 0.1% TFA)) was used to yield the TFA salt of the title compound as a colorless solid (18 mg, 28%). ^1^H NMR (400 MHz, DMSO-*d*_*6*_, δ [ppm]): 12.25 (bs, 1H, −NH-CO-CH_2_-S-), 11.20 (bs, 1H, −CO-NH-OH), 8.86 (bs, 1H, −CO-NH-OH), 7.65 (s, 1H, *N*-hydroxybenzamide H-2), 7.58 (dt, 1H, ^3^*J* = 6.7 Hz, ^4^*J* = 1.8 Hz, *N*-hydroxybenzamide H-6), 7.43–7.34 (m, 2H, *N*-hydroxybenzamide H-4,5), 7.28 (s, 1H, thiazole H-4), 6.94 (s, 1H, pyrimidine H-5), 4.12 (s, 2H, thiazole-CH_2_−), 4.08 (s, 2H, −NH-CO-CH_2_-S−), 2.28 (s, 6H, pyrimidine-CH_3_). ^13^C NMR (151 MHz, DMSO*-d*_*6*_, δ [ppm]): 168.9 q (pyrimidine C-2), 167.0 q (pyrimidine C-4,6), 166.8 q (−NH-CO-CH_2_-S−), 164.1 q (−CO-NH-OH), 158.3 q (q, ^2^*J* = 37.2 Hz, −OOC-CF_3_), 157.0 q (thiazole C-2), 140.6 q (*N*-hydroxybenzamide C-3), 134.9 (thiazole C-4), 133.1 q (*N*-hydroxybenzamide C-1), 131.1 (*N*-hydroxybenzamide C-4), 130.7 q (thiazole C-5), 128.5 (*N*-hydroxybenzamide C-2), 127.0 (*N*-hydroxybenzamide C-5), 124.8 (*N*-hydroxybenzamide C-6), 116.1 (pyrimidine C-5), 115.3 q (q, ^1^*J* = 288.4 Hz, ^–^OOC-CF_3_), 34.1 (−NH-CO-CH_2_-S−), 31.7 (thiazole-CH_2_−), 23.2 (pyrimidine-CH_3_), ^19^F NMR (377 MHz, DMSO-*d*_6_, δ [ppm]): −74.47. HRMS (ESI^+^): *m*/*z* calcd for C_19_H_19_N_5_O_3_S_2_Na^+^: 452.0822 [M + Na]^+^; found, 452.0819. LRMS *m*/*z* (ESI^+^): 430 [M + H]^+^. HPLC retention time 14.04 min, 96.0% (M1). ^*(a*) 1^H signal of and F_3_C-COOH could not be detected.

#### 4-((2-(2-((4,6-Dimethylpyrimidin-2-yl)thio)acetamido)thiazol-5-yl)methyl)-*N*-hydroxybenzamide (**22**)

Ethyl 4-((2-(2-((4,6-dimethylpyrimidin-2-yl)thio)acetamido)thiazol-5-yl)methyl)benzoate (**30**, 52.4 mg, 118 μmol, 1 equiv) was dissolved in a mixture of 0.35 mL of dichloromethane and 0.71 mL of methanol and cooled on an ice bath to 0 °C. Then 220 μL (234 mg, 50%: 117 mg, 3.55 mmol, 30 equiv) of an aqueous 50 wt % hydroxylamine solution were added, and the mixture was stirred at 0 °C for 10 min before 47.3 mg (1.18 mmol, 10 equiv) sodium hydroxide were added. After 30 min at 0 °C, the ice bath was removed, and stirring of the colorless clear solution was continued at room temperature for 2 h. The reaction mixture was dried under reduced pressure, and the residue was taken up in 2.5 mL of deionized water. The suspension was cooled in an ice bath and brought to pH = 8 by the dropwise addition of 1 M HCl, when large amounts of white solid formed. The solid was collected by filtration. The precipitate was washed with water (2 × 1 mL) and ice-cold diethyl ether (2 mL) and dried under reduced pressure. The residue was purified by flash column chromatography (MeOH (+0.5% AcOH)/dichloromethane (+0.5% AcOH): gradient 0–10%). For the final purification, the crude material from column chromatography was dissolved in 0.5 M NaOH (50 mL). The aqueous layer was washed with EtOAc (2 × 30 mL). Then, the aqueous layer was acidified to pH = 4 with 1 M HCl and extracted with EtOAc (3 × 30 mL). The combined organic layer was washed with brine (50 mL), dried over Na_2_SO_4_, filtered, and concentrated under reduced pressure to yield the title compound as a colorless solid (17 mg, 33%). ^1^H NMR (400 MHz, DMSO-*d*_6_, δ [ppm]): 12.23 (bs, 1H, −NH-CO-CH_2_-S−), 11.15 (bs, 1H, −CO-NH-OH), 9.01 (bs, 1H, −CO-NH-OH), 7.82–7.58 (m, 2H, *N*-hydroxybenzamide H-2,6), 7.34–7.30 (m, 2H, *N*-hydroxybenzamide H-3,5), 7.27 (s, 1H, thiazole H-4), 6.94 (s, 1H, pyrimidine H-5), 4.11 (s, 2H, thiazole-CH_2_−), 4.08 (s, 2H, −NH-CO-CH_2_-S−), 2.28 (s, 6H, pyrimidine-CH_3_). ^13^C NMR (101 MHz, DMSO-*d*_6_, δ [ppm]): 169.4 q (pyrimidine C-2), 167.5 q (pyrimidine C-4,6), 167.4 q (−NH-CO-CH_2_-S−), 164.6 q (−CO-NH-OH), 157.5 q (thiazole C-2), 144.0 q (*N*-hydroxybenzamide C-4), 135.5 (thiazole C-4), 131.5 q (thiazole C-5), 131.1 q (*N*-hydroxybenzamide C-1), 128.8 (*N*-hydroxybenzamide C-3,5), 127.7 (*N*-hydroxybenzamide C-2,6), 116.6 (pyrimidine C-5), 34.5 (−NH-CO-CH_2_-S−), 32.2 (thiazole-CH_2_−), 23.7 (pyrimidine −CH_3_). HRMS (ESI^+^): *m*/*z* calcd for C_19_H_19_N_5_O_3_S_2_Na^+^: 452.0822 [M + Na]^+^, found: 452.0821. LRMS *m*/*z* (ESI^+^): 430 [M + H]^+^. HPLC retention time 13.89 min, 98.0% (M1).

#### Ethyl 3-((2-Aminothiazol-5-yl)methyl)benzoate (**25**)^61^

A solution of sodium nitrite (1.93 g, 28.0 mmol, 1.08 equiv) in water (32 mL) was added dropwise to a solution of ethyl 3-aminobenzoate (4.29 g, 26.0 mmol, 1 equiv) in 18% aqueous HCl (32 mL) at −5 °C. After stirring at 0 °C for 30 min, the reaction mixture was carefully neutralized with NaHCO_3_ and subsequently added to an ice-cold suspension of acrolein (4.29 mL, 64.3 mmol, 2.47 equiv), CuCl_2_ × 2H_2_O (1.287 g, 7.55 mmol, 0.29 equiv), and CaO (429 mg, 7.65 mmol, 0.29 equiv) in acetone (64 mL). The solution was stirred at room temperature for 2 h, until the nitrogen formation stopped. Acetone was removed by evaporation, and the resulting mixture was extracted with dichloromethane, filtered, dried over MgSO_4_, and concentrated *in vacuo*. The α-chloropropanal intermediate (**23**) was directly dissolved in ethanol (85 mL), and thiourea (2.37 g, 31.2 mmol, 1.2 equiv) was added. The mixture was refluxed for 24 h. After cooling to room temperature, the reaction mixture was neutralized by the addition of NaHCO_3_. The resulting mixture was filtered, and the filtrate was concentrated under reduced pressure. The crude product was purified by flash column chromatography (EtOAc/isohexane: gradient 0–100%) to yield the title compound as a brown resin (140 mg, 2%). H NMR (400 MHz, DMSO-*d*_6_, δ [ppm]): 7.86–7.75 (m, 2H, ethyl benzoate H-2,6), 7.55–7.40 (m, 2H, ethyl benzoate H-4,5), 6.78–6.70 (m, 3H, −NH_2_, thiazole H-4), 4.30 (q, 2H, ^3^*J* = 7.1 Hz, H_3_C-CH_2_-O-CO−), 3.99 (s, 2H, thiazole-CH_2_−), 1.31 (t, 3H, ^3^*J* = 7.1 Hz, H_3_C-CH_2_-O-CO−). ^13^C NMR (151 MHz, DMSO-*d*_6_, δ [ppm]): 168.2 q (thiazole C-2), 165.7 q (H_3_C-CH_2_-O-CO−), 141.3 q (ethyl benzoate C-3), 135.8 (thiazole C-4), 133.1 (ethyl benzoate C-4), 130.0 q (ethyl benzoate C-1), 128.9 (ethyl benzoate C-2), 128.7 (ethyl benzoate C-5), 127.1 (ethyl benzoate C-6), 124.1 q (thiazole C-5), 60.7 (H_3_C-CH_2_-O-CO−), 32.1 (thiazole-CH_2_−), 14.2 (H_3_C-CH_2_-O-CO−). LRMS *m*/*z* (ESI^+^): 263 [M + H]^+^. The spectroscopic data are in good agreement with the literature values.^61^ The attached ^1^H and ^13^C NMR spectra contains residual solvent signals (MeOH).

#### Ethyl 4-((2-Aminothiazol-5-yl)methyl)benzoate (**26**)^62^

A solution of sodium nitrite (1.80 g, 26.1 mmol, 1.08 equiv) in water (30 mL) was added dropwise to a solution of ethyl 4-aminobenzoate (4.00 g, 24.2 mmol, 1 equiv) in 18% aqueous HCl (30 mL) at −5 °C. After stirring at 0 °C for 30 min, the reaction mixture was carefully neutralized with NaHCO_3_ and subsequently added to an ice-cold suspension of acrolein (4.00 mL, 60.0 mmol, 2.47 equiv), CuCl_2_·2H_2_O (1.20 g, 7.04 mmol, 0.29 equiv), and CaO (400 mg, 7.13 mmol, 0.29 equiv) in acetone (60 mL). The solution was stirred at room temperature for 2 h, until the nitrogen formation stopped. Acetone was removed by evaporation and the resulting mixture extracted with dichloromethane, filtered, dried over MgSO_4_, and concentrated *in vacuo*. The α-chloropropanal intermediate (**24**) was directly dissolved in ethanol (20 mL), and thiourea (2.21 g, 29.1 mmol, 1.2 equiv) was added. The mixture was refluxed for 2 h. After cooling to room temperature, water (100 mL) was added, and the mixture was neutralized with ammonia. The aqueous layer was extracted with ethyl acetate (3 × 100 mL). Then, the combined organic layer was extracted with 0.5 M HCl (5 × 100 mL). The combined aqueous layer was carefully neutralized by the addition of NaHCO_3_. The neutralized aqueous layer was extracted with EtOAc (3 × 100 mL). The combined organic layer from the last extraction step was dried over Na_2_SO_4_, filtered, and concentrated under reduced pressure. The title compound was obtained as a brown resin (1.07 g, 17%). ^1^H NMR (400 MHz, DMSO-*d*_6_, δ [ppm]): 7.93–7.86 (m, 2H, ethyl benzoate H-2,6), 7.40–7.31 (m, 2H, ethyl benzoate H-3,5), 6.76 (bs, 2H, −NH_2_), 6.73 (s, 1H, thiazole H-4), 4.29 (q, 2H, ^3^*J* = 7.1 Hz, H_3_C-CH_2_-O-CO−), 3.98 (s, 2H, thiazole-CH_2_−), 1.30 (t, 3H, ^3^*J* = 7.1 Hz, H_3_C-CH_2_-O-CO−). ^13^C NMR (151 MHz, DMSO-*d*_6_, δ [ppm]): 168.2 q (thiazole C-2), 165.6 q (H_3_C-CH_2_-O-CO−), 146.2 q (ethyl benzoate C-4), 136.0 (thiazole C-4), 129.3 (ethyl benzoate C-2,6), 128.5 (ethyl benzoate C-3,5), 128.0 q (ethyl benzoate C-1), 123.5 q (thiazole C-5), 60.6 (H_3_C-CH_2_-O-CO−), 32.4 (thiazole-CH_2_−), 14.2 (H_3_C-CH_2_-O-CO−). LRMS *m*/*z* (ESI^+^): 263 [M + H]^+^. The spectroscopic data are in good agreement with the literature values.^62^ The attached ^1^H and ^13^C NMR spectra contains residual solvent signals (MeOH).

#### Ethyl 3-((2-(2-Chloroacetamido)thiazol-5-yl)methyl)benzoate (**27**)

Ethyl 3-((2-aminothiazol-5-yl)methyl)benzoate (**25**, 120 mg, 457 μmol, 1 equiv) was dissolved in acetonitrile (4 mL) and *N*,*N*-diisopropylethylamine (139 μL, 801 μmol, 1.75 equiv). The mixture was stirred and cooled to 0 °C. Chloroacetyl chloride (64 μL, 801 μmol, 1.75 equiv) was added at 0 °C. After stirring for 2 h at room temperature, volatiles were removed under reduced pressure. The red-brown, oily residue was mixed with water (20 mL). The aqueous layer was extracted with ethyl acetate (3 × 20 mL). The combined organic layer was washed with 1 M HCl (60 mL), brine (60 mL), dried over Na_2_SO_4_, filtered, and dried under reduced pressure. The brown crude product was purified by flash column chromatography (EtOAc/isohexane: gradient 5–80%) to yield the title compound as a tan solid (88 mg, 57%). ^1^H NMR (400 MHz, DMSO-*d*_6_, δ [ppm]): 12.38 (bs, 1H, −NH-CO-CH_2_-Cl), 7.86 (dd, 1H, ^4^*J* = 1.4 Hz, 1.4 Hz, ethyl benzoate H-2), 7.83 (ddd, 1H, ^3^*J* = 7.6, ^4^*J* = 1.4 Hz, 1.4 Hz, ethyl benzoate H-6), 7.56 (ddd, 1H, ^3^*J* = 7.6, ^4^*J* = 1.4 Hz, 1.4 Hz, ethyl benzoate H-4), 7.47 (dd, 1H, ^3^*J* = 7.6 Hz, 7.6 Hz, ethyl benzoate H-5), 7.32 (s, 1H, thiazole H-4), 4.34 (s, 2H, −NH-CO-CH_2_-Cl), 4.30 (q, 2H, ^3^*J* = 7.1 Hz, H_3_C-CH_2_-O-CO−), 4.19 (s, 2H, thiazole-CH_2_−), 1.31 (t, 3H, ^3^*J* = 7.1 Hz, H_3_C-CH_2_-O-CO−). ^13^C NMR (151 MHz, DMSO-*d*_6_, δ [ppm]): 165.6 q (H_3_C-CH_2_-O-CO−), 164.7 q (−NH-CO-CH_2_-Cl), 156.6 q (thiazole C-2), 140.9 q (ethyl benzoate C-3), 135.2 (thiazole C-4), 133.3 (ethyl benzoate C-4), 131.4 q (thiazole C-5), 130.2 q (ethyl benzoate C-1), 129.0 (ethyl benzoate C-2), 128.9 (ethyl benzoate C-5), 127.3 (ethyl benzoate C-6), 60.7 (H_3_C-CH_2_-O-CO−), 42.2 (−NH-CO-CH_2_-Cl), 31.5 (thiazole-CH_2_−), 14.2 (H_3_C-CH_2_-O-CO−). HRMS (ESI^+^): *m*/*z* calcd for C_15_H_16_ClN_2_O_3_S^+^: 339.0565 [M + H]^+^, found: 339.0564. LRMS *m*/*z* (ESI^+^): 339 [M + H]^+^. The attached ^1^H and ^13^C NMR spectra contains residual solvent signals (EtOAc).

#### Ethyl 4-((2-(2-Chloroacetamido)thiazol-5-yl)methyl)benzoate (**28**)

Ethyl 4-((2-aminothiazol-5-yl)methyl)benzoate (**26**, 585 mg, 2.23 mmol, 1 equiv) was dissolved in acetonitrile (15 mL) and *N*,*N*-diisopropylethylamine (680 μL, 3.90 mmol, 1.75 equiv). The mixture was stirred and cooled to 0 °C. Chloroacetyl chloride (310 μL, 3.90 mmol, 1.75 equiv) was added at 0 °C. After stirring for 2 h at room temperature, volatiles were removed under reduced pressure. The red-brown, oily residue was mixed with water (15 mL). The aqueous layer was extracted with ethyl acetate (3 × 20 mL). The combined organic layer was washed with 1 M HCl (20 mL), brine (20 mL), dried over Na_2_SO_4_, filtered, and dried under reduced pressure. The brown crude product was purified by flash column chromatography (EtOAc/isohexane: gradient 8–75%) to yield the title compound as a tan solid (471 mg, 62%). ^1^H NMR (400 MHz, DMSO-*d*_6_, δ [ppm]): 12.38 (bs, 1H, −NH-CO-CH_2_-Cl), 7.95–7.87 (m, 2H, ethyl benzoate H-2,6), 7.48–7.37 (m, 2H, ethyl benzoate H-3,5), 7.32 (s, 1H, thiazole H-4), 4.34 (s, 2H, −NH-CO-CH_2_-Cl), 4.29 (q, 2H, ^3^*J* = 7.1 Hz, H_3_C-CH_2_-O-CO−), 4.19 (s, 2H, thiazole-CH_2_−), 1.30 (t, 3H, ^3^*J* = 7.1 Hz, H_3_C-CH_2_-O-CO−). ^13^C NMR (151 MHz, DMSO-*d*_6_, δ [ppm]): 165.5 q (H_3_C-CH_2_-O-CO−), 164.7 q (−NH-CO-CH_2_-Cl), 156.6 q (thiazole C-2), 145.7 q (ethyl benzoate C-4), 135.3 (thiazole C-4), 130.8 q (thiazole C-5), 129.5 (ethyl benzoate C-2,6), 128.7 (ethyl benzoate C-3,5), 128.2 q (ethyl benzoate C-1), 60.6 (H_3_C-CH_2_-O-CO−), 42.2 (−NH-CO-CH_2_-Cl), 31.7 (thiazole-CH_2_−), 14.2 (H_3_C-CH_2_-O-CO−). HRMS (ESI^+^): *m*/*z* calcd for C_15_H_16_ClN_2_O_3_S^+^: 339.0565 [M + H]^+^, found: 339.0566. LRMS *m*/*z* (ESI^+^): 339 [M + H]^+^. The attached ^1^H and ^13^C NMR spectra contains residual solvent signals (EtOAc).

#### Ethyl 3-((2-(2-((4,6-Dimethylpyrimidin-2-yl)thio)acetamido)thiazol-5-yl)methyl)benzoate (**29**)

4,6-Dimethyl-2-methylsulfanyl-pyrimidine (32 mg, 0.227 mmol, 1 equiv) was dissolved in dimethyl sulfoxide (DMSO, 2 mL). Na_2_CO_3_ (48 mg, 0.454 mmol, 2 equiv) and KI (38 mg, 0.227 mmol, 1 equiv) were added. The mixture was stirred for 15 min at ambient temperature. Then, ethyl 3-((2-(2-chloroacetamido)thiazol-5-yl)methyl)benzoate (**27**, 77 mg, 0.277 mmol, 1 equiv) was given to the reaction mixture and stirred for 1.5 h. After completion, water (20 mL) was added. The aqueous layer was extracted with ethyl acetate (3 × 40 mL). The combined organic layer was dried over Na_2_SO_4_, filtered, and dried under reduced pressure. The brown crude product was purified by flash column chromatography (EtOAc/isohexane: gradient 8–80%) to yield the title compound as a tan solid (91 mg, 91%). ^1^H NMR (400 MHz, DMSO-*d*_6_, δ [ppm]): 12.25 (bs, 1H, −NH-CO-CH_2_-S−), 7.84 (dd, 1H, ^4^*J* = 1.4 Hz, 1.4 Hz, ethyl benzoate H-2), 7.81 (ddd, 1H, ^3^*J* = 7.6, ^4^*J* = 1.4 Hz, 1.4 Hz, ethyl benzoate H-6), 7.53 (ddd, 1H, ^3^*J* = 7.6, ^4^*J* = 1.4 Hz, 1.4 Hz, ethyl benzoate H-4), 7.45 (dd, 1H, ^3^*J* = 7.6 Hz, 7.6 Hz, ethyl benzoate H-5), 7.29 (s, 1H, thiazole H-4), 6.93 (s, 1H, pyrimidine H-5), 4.29 (q, 2H, ^3^*J* = 7.1 Hz, H_3_C-CH_2_-O-CO−), 4.16 (s, 2H, thiazole-CH_2_−), 4.08 (s, 2H, −NH-CO-CH_2_-S−), 2.27 (s, 6H, pyrimidine −CH_3_), 1.29 (t, 3H, ^3^*J* = 7.1 Hz, H_3_C-CH_2_-O-CO−). ^13^C NMR (151 MHz, DMSO-*d*_6_, δ [ppm]): 168.9 q (pyrimidine C-2), 167.0 q (pyrimidine C-4,6), 166.9 q (−NH-CO-CH_2_-S−), 165.6 q (H_3_C-CH_2_-O-CO−), 157.1 q (thiazole C-2), 141.0 q (ethyl benzoate C-3), 135.0 (thiazole C-4), 133.2 (ethyl benzoate C-4), 130.7 q (thiazole C-5), 130.2 q (ethyl benzoate C-1), 129.0 (ethyl benzoate C-5), 128.8 (ethyl benzoate C-2), 127.3 (ethyl benzoate C-6), 116.1 (pyrimidine C-5), 60.7 (H_3_C-CH_2_-O-CO−), 34.0 (−NH-CO-CH_2_-S-), 31.5 (thiazole-CH_2_−), 23.2 (pyrimidine-CH_3_), 14.1 (H_3_C-CH_2_-O-CO−). HRMS (ESI^+^): *m*/*z* calcd for C_21_H_22_N_4_O_3_S_2_Na ^+^: 465.1026 [M + Na]^+^, found: 465.1025. LRMS *m*/*z* (ESI^+^): 443 [M + H]^+^. The attached ^1^H and ^13^C NMR spectra contains residual solvent signals (EtOAc).

#### Ethyl 4-((2-(2-((4,6-Dimethylpyrimidin-2-yl)thio)acetamido)thiazol-5-yl)methyl)benzoate (**30**)

4,6-Dimethyl-2-methylsulfanyl-pyrimidine (129 mg, 0.922 mmol) was dissolved in dimethyl sulfoxide (DMSO, 8 mL). Na_2_CO_3_ (195 mg, 1.84 mmol), and KI (154 mg, 0.922 mmol) were added. The mixture was stirred for 15 min at room temperature. Then, ethyl 4-((2-(2-chloroacetamido)thiazol-5-yl)methyl)benzoate (**28**, 312 mg, 0.922 mmol) was given to the reaction mixture and stirred for 2 h. After completion, water (20 mL) was added. The aqueous layer was extracted with ethyl acetate (3 × 40 mL). The combined organic layer was dried over Na_2_SO_4_, filtered, and dried under reduced pressure. The brown crude product was purified by flash column chromatography (EtOAc/isohexane: gradient 8–80%) to yield the title compound as a tan solid (191 mg, 47%). ^1^H NMR (400 MHz, DMSO-*d*_6_, δ [ppm]): 12.26 (bs, 1H, −NH-CO-CH_2_-S−), 7.93–7.86 (m, 2H, ethyl benzoate H-2,6), 7.43–7.36 (m, 2H, ethyl benzoate H-3,5), 7.28 (s, 1H, thiazole H-4), 6.93 (s, 1H, pyrimidine H-5), 4.28 (q, 2H, ^3^*J* = 7.1 Hz, H_3_C-CH_2_-O-CO−), 4.16 (s, 2H, thiazole-CH_2_−), 4.08 (s, 2H, −NH-CO-CH_2_-S−), 2.27 (s, 6H, pyrimidine -CH_3_), 1.29 (t, 3H, ^3^*J* = 7.1 Hz, H_3_C-CH_2_-O-CO−). ^13^C NMR (151 MHz, DMSO-*d*_6_, δ [ppm]): 168.9 q (pyrimidine C-2), 167.0 q (pyrimidine C-4,6), 166.8 q (−NH-CO-CH_2_-S−), 165.5 q (H_3_C-CH_2_-O-CO−), 157.1 q (thiazole C-2), 145.8 q (ethyl benzoate C-4), 135.1 (thiazole C-4), 130.1 q (thiazole C-5), 129.4 (ethyl benzoate C-2,6), 128.7 (ethyl benzoate C-3,5), 128.1 q (ethyl benzoate C-1), 116.1 (pyrimidine C-5), 60.6 (H_3_C-CH_2_-O-CO−), 34.0 (−NH-CO-CH_2_-S−), 31.7 (thiazole-CH_2_−), 23.2 (pyrimidine-CH_3_), 14.1 (H_3_C-CH_2_-O-CO−). HRMS (ESI^+^): *m*/*z* calcd for C_21_H_22_N_4_O_3_S_2_Na^+^: 465.1026 [M + Na]^+^, found: 465.1024. LRMS *m*/*z* (ESI^+^): 443 [M + H]^+^.

#### 4-((4-((3-((2-(2-((4,6-Dimethylpyrimidin-2-yl)thio)acetamido)thiazol-5-yl)methyl)phenoxy)methyl)-1*H*-1,2,3-triazol-1-yl)methyl)-*N*-hydroxybenzamide (**31**)

Ethyl 4-((4-((3-((2-(2-((4,6-dimethylpyrimidin-2-yl)thio)acetamido)thiazol-5-yl)methyl)phenoxy)methyl)-1*H*-1,2,3-triazol-1-yl)methyl)benzoate (**36**, 23.5 mg, 37.3 μmol, 1 equiv) was dissolved in a mixture of 0.71 mL of dichloromethane and 0.71 mL of methanol and cooled in an ice bath to 0 °C. Then 69.4 μL (74 mg, 50%: 37 mg, 1.12 mmol, 30 equiv) of an aqueous 50 wt % hydroxylamine solution were added, and the mixture was stirred at 0 °C for 10 min before 14.9 mg (0.37 mmol, 10 equiv) sodium hydroxide were added. After 30 min at 0 °C, the ice bath was removed, and stirring of the colorless clear solution was continued at ambient temperature for 3 h. The reaction mixture was dried under reduced pressure and the residue was taken up in 2 mL of deionized water. The suspension was cooled in an ice bath and brought to pH = 8 by the dropwise addition of 1 M HCl, when large amounts of white solid formed. The solid was collected by centrifugation, washed with ice-cold diethyl ether (2 mL), and dried under reduced pressure. For the final purification preparative HPLC (gradient elution from 25% to 50% acetonitrile/water (+0.1% TFA)) was used to yield the TFA salt of the title compound as a colorless solid (19 mg, 70%). ^1^H NMR^a^ (400 MHz, DMSO-*d*_6_, δ [ppm]): 12.23 (bs, 1H, −NH-CO-CH_2_-S−), 11.22 (bs, 1H, −CO-NH-OH), 9.10 (bs, 1H, −CO-NH-OH), 8.29 (s, 1H, triazole-H), 7.81–7.63 (m, 2H, *N*-hydroxybenzamide H-2,6), 7.40–7.30 (m, 2H, *N*-hydroxybenzamide H-3,5), 7.25 (s, 1H, thiazole H-4), 7.22 (t, 1H, ^3^*J* = 7.9 Hz, phenyl H-5), 6.92 (s, 1H, pyrimidine H-5), 6.91–6.79 (m, 3H, phenyl H-2,4,6), 5.65 (s, 2H, *N*-hydroxybenzamide-CH_2_-triazole), 5.10 (s, 2H, triazole-CH_2_-O−), 4.08 (s, 2H, −NH-CO-CH_2_-S−), 4.03 (s, 2H, thiazole-CH_2_-phenyl), 2.27 (s, 6H, pyrimidine-CH_3_). ^13^C NMR (151 MHz, DMSO-*d*_6_, δ [ppm]): 168.9 q (pyrimidine C-2), 167.0 q (pyrimidine C-4,6), 166.8 q (−NH-CO-CH_2_-S−), 163.7 q (−CO-NH-OH), 158.1 q (q, ^2^*J* = 35.2 Hz, −OOC-CF_3_), 158.1 q (phenyl C-3), 156.9 q (thiazole C-2), 143.0 q (triazole C-4), 141.9 q (phenyl C-1), 138.9 q (*N*-hydroxybenzamide C-4), 134.7 (thiazole C-4), 132.6 q (*N*-hydroxybenzamide C-1), 131.0 q (thiazole C-5), 129.6 (phenyl C-5), 127.9 (*N*-hydroxybenzamide C-3,5), 127.3 (*N*-hydroxybenzamide C-2,6), 124.8 (triazole C-5), 120.9 (phenyl C-6), 116.1 (pyrimidine C-5), 116.0 (q, ^1^*J* = 294.5 Hz, −OOC-CF_3_), 115.0 (phenyl C-2), 112.4 (phenyl C-4), 61.0 (triazole-CH_2_-O−), 52.4 (*N*-hydroxybenzamide-CH_2_-triazole), 34.1 (−NH-CO-CH_2_-S-), 31.9 (thiazole-CH_2_-phenyl), 23.2 (pyrimidine −CH_3_). ^19^F NMR (377 MHz, DMSO-*d*_6_, δ [ppm]): −74.0. HRMS (ESI^+^): *m*/*z* calcd for C_29_H_29_N_8_O_4_S_2_^+^: 617.1748 [M + H]^+^, found: 616.1745. LRMS *m*/*z* (ESI^+^): 617 [M + H]^+^. HPLC retention time 15.33 min, >99.5% (M1). ^(*a)*1^H signal of and F_3_C-COOH could not be detected.

#### 4-((5-(4-((3-((2-(2-((4,6-Dimethylpyrimidin-2-yl)thio)acetamido)thiazol-5-yl)methyl)phenoxy)methyl)-1*H*-1,2,3-triazol-1-yl)pentanamido)methyl)-*N*-hydroxybenzamide (**32**)

Ethyl 4-((5-(4-((3-((2-(2-((4,6-dimethylpyrimidin-2-yl)thio)acetamido)thiazol-5-yl)methyl)phenoxy)methyl)-1*H*-1,2,3-triazol-1-yl)pentanamido)methyl)benzoate (**39**, 29.8 mg, 40.9 μmol, 1 equiv) was dissolved in a mixture of 0.78 mL of dichloromethane and 0.78 mL of methanol and cooled in an ice bath to 0 °C. Then 76 μL (81 mg, 50%: 41 mg, 1.23 mmol, 30 equiv) of an aqueous 50 wt % hydroxylamine solution were added, and the mixture was stirred at 0 °C for 10 min, before 16.4 mg (0.409 mmol, 10 equiv) sodium hydroxide were added. After 30 min at 0 °C, the ice bath was removed and stirring of the colorless clear solution was continued at ambient temperature for 3 h. The reaction mixture was dried under reduced pressure, and the residue was taken up in 2 mL of deionized water. The suspension was cooled in an ice bath and brought to pH = 8 by the dropwise addition of 1 M HCl, then large amounts of white solid formed. The solid was collected by centrifugation, washed with ice-cold diethyl ether (2 mL), and dried under reduced pressure. For the final purification, preparative HPLC (gradient elution from 25% to 50% acetonitrile/water (+0.1% TFA)) was used to yield the TFA salt of the title compound as a colorless solid (16 mg, 47%). ^1^H NMR^a^ (400 MHz, DMSO-*d*_6_, δ [ppm]): 12.23 (bs, 1H, −NH-CO-CH_2_-S−), 11.18 (bs, 1H, −CO-NH-OH), 8.40 (t, 1H, ^3^*J* = 5.9 Hz, −CH_2_-NH-CO-(CH_2_)_4_-triazole), 8.20 (s, 1H, triazole-H), 7.72–7.62 (m, 2H, *N*-hydroxybenzamide H-2,6), 7.34–7.17 (m, 4H, *N*-hydroxybenzamide H-3,5, thiazole H-4, phenyl H-5), 6.94 (s, 1H, pyrimidine H-5), 6.91–6.79 (m, 3H, phenyl H-2,4,6), 5.09 (s, 2H, triazole-CH_2_-O−), 4.36 (t, 2H, ^3^*J* = 7.0 Hz, −CH_2_-NH-CO-(CH_2_)_3_-CH_2_-triazole), 4.28 (d, 2H, ^3^*J* = 5.9 Hz, −CH_2_-NH-CO-(CH_2_)_4_-triazole), 4.08 (s, 2H, -NH-CO-CH_2_-S−), 4.03 (s, 2H, thiazole-CH_2_-phenyl), 2.27 (s, 6H, pyrimidine −CH_3_), 2.19 (t, 2H, ^3^*J* = 7.4 Hz, −CH_2_-NH-CO-CH_2_-(CH_2_)_3_-triazole), 1.87–1.72 (m, 2H, −CH_2_-NH-CO-(CH_2_)_2_-CH_2_-CH_2_-triazole), 1.55–1.42 (m, 2H, −CH_2_-NH-CO-CH_2_-CH_2_-(CH_2_)_2_-triazole). ^13^C NMR (101 MHz, DMSO-*d*_6_, δ [ppm]): 171.8 q (−CH_2_-NH-CO-(CH_2_)_4_-triazole), 168.9 q (pyrimidine C-2), 167.0 q (pyrimidine C-4,6), 166.9 q (−NH-CO-CH_2_-S−), 164.0 q (−CO-NH-OH), 158.3 q (q, ^2^*J* = 37.7 Hz, −OOC-CF_3_), 158.2 q (phenyl C-3), 157.0 q (thiazole C-2), 143.0 q (*N*-hydroxybenzamide C-4), 142.6 q (triazole C-4), 141.9 q (phenyl C-1), 134.8 (thiazole C-4), 131.2 q (*N*-hydroxybenzamide C-1), 131.1 q (thiazole C-5), 129.7 (phenyl C-5), 127.0 (*N*-hydroxybenzamide C-3,5), 126.9 (*N*-hydroxybenzamide C-2,6), 124.4 (triazole C-5), 120.9 (phenyl C-6), 116.1 (pyrimidine C-5), 115.3 (q, ^1^*J* = 293.6 Hz, −OOC-CF_3_), 115.0 (phenyl C-2), 112.5 (phenyl C-4), 61.1 (triazole-CH_2_-O−), 49.1 (−CH_2_-NH-CO-(CH_2_)_3_-CH_2_-triazole), 41.8 (−CH_2_-NH-CO-(CH_2_)_4_-triazole), 34.5 (−CH_2_-NH-CO-CH_2_-(CH_2_)_3_-triazole), 34.1 (−NH-CO-CH_2_-S−), 31.9 (thiazole-CH_2_-phenyl), 29.4 (−CH_2_-NH-CO-(CH_2_)_2_-CH_2_-CH_2_-triazole), 23.3 (pyrimidine −CH_3_), 22.2 (−CH_2_-NH-CO-CH_2_-CH_2_-(CH_2_)_2_-triazole). ^19^F NMR (565 MHz, DMSO-*d*_6_, δ [ppm]): −74.6. HRMS (ESI^+^): *m*/*z* calcd for C_34_H_38_N_9_O_5_S_2_^+^: 716.2432 [M + H]^+^, found: 716.2431. LRMS *m*/*z* (ESI^+^): 716 [M + H]^+^. HPLC retention time 15.03 min, >99.5% (M1). The attached ^1^H and ^13^C NMR spectra contains residual solvent signals (MeOH, acetone). ^(*a*)1^H signals of −CONH-OH and F_3_C-COOH could not be detected.

4-((*N*-Butyl-5-(4-((3-((2-(2-((4,6-dimethylpyrimidin-2-yl)thio)acetamido)thiazol-5-yl)methyl)phenoxy)methyl)-1*H*-1,2,3-triazol-1-yl)pentanamido)methyl)-*N*-hydroxybenzamide (**33**, Mz325). Ethyl 4-((*N*-butyl-5-(4-((3-((2-(2-((4,6-dimethylpyrimidin-2-yl)thio)acetamido)thiazol-5-yl)methyl)phenoxy)methyl)-1*H*-1,2,3-triazol-1-yl)pentanamido)methyl)benzoate (**43**, 30 mg, 38.2 μmol, 1 equiv) was dissolved in a mixture of 0.78 mL of dichloromethane and 0.78 mL of methanol and cooled in an ice bath to 0 °C. Then 71 μL (76 mg, 50%: 38 mg, 1.15 mmol, 30 equiv) of an aqueous 50 wt % hydroxylamine solution were added, and the mixture was stirred at 0 °C for 10 min, before 15.2 mg (0.382 mmol, 10 equiv) sodium hydroxide were added. After 30 min at 0 °C, the ice bath was removed and stirring of the colorless clear solution was continued at ambient temperature for 3 h. The reaction mixture was dried under reduced pressure, and the residue was taken up in 2 mL of deionized water. The suspension was cooled in an ice bath and brought to pH = 8 by the dropwise addition of 1 M HCl, when large amounts of white solid formed. The solid was collected by centrifugation, washed with ice-cold diethyl ether (2 mL), and dried under reduced pressure. For the final purification, preparative HPLC (gradient elution from 35% to 56% acetonitrile/water (+0.1% TFA)) was used to yield the TFA salt of the title compound as a colorless solid (15 mg, 52%). ^1^H NMR^a^ (600 MHz, DMSO-*d*_6_, δ [ppm]): 12.19 (bs, 1H, −NH-CO-CH_2_-S−), 11.16 (bs, 1H, −CO-NH-OH), 8.20, 8.16 (2*s*, 1H, triazole-H), 7.77–7.64 (m, 2H, *N*-hydroxybenzamide H-2,6), 7.30–7.18 (m, 4H, *N*-hydroxybenzamide H-3,5, thiazole H-4, phenyl H-5), 6.93 (s, 1H, pyrimidine H-5), 6.91–6.87 (m, 2H, phenyl H-2,6), 6.85–6.79 (m, 1H, phenyl H-4), 5.10, 5.08 (2*s*, 2H, triazole-CH_2_-O−), 4.59–4.52 (2*s*, 2H, −CH_2_-N(C_4_H_9_)-CO-(CH_2_)_4_−), 4.38, 4.31 (2*t*, ^3^*J* = 7.0 Hz, 2H, −CH_2_-N(C_4_H_9_)-CO-(CH_2_)_3_-CH_2_-triazole), 4.08 (s, 2H, −NH-CO-CH_2_-S−), 4.03 (s, 2H, thiazole-CH_2_-phenyl), 3.24–3.17 (m, 2H, −CH_2_-N(CH_2_-C_3_H_7_)-CO-(CH_2_)_4_−), 2.42, 2.30 (2*t*, 2H, ^3^*J* = 7.4 Hz, −CH_2_-N(C_4_H_9_)-CO-CH_2_-(CH_2_)_3_−), 2.28 (s, 6H, pyrimidine-CH_3_), 1.89–1.74 (m, 2H, −CH_2_-N(C_4_H_9_)-CO-(CH_2_)_2_-CH_2_-CH_2_−), 1.57–1.35 (m, 4H, −CH_2_-N(CH_2_-CH_2_-C_2_H_5_)-CO-CH_2_-CH_2_-(CH_2_)_2_−), 1.28–1.15 (m, 2H, −CH_2_-N((CH_2_)_2_-CH_2_-CH_3_)-CO-(CH_2_)_4_−), 0.84, 0.83 (2*t*, 3H, ^3^*J* = 7.2 Hz, −CH_2_-N((CH_2_)_3_-CH_3_)-CO-(CH_2_)_4_−). ^13^C NMR^b^ (151 MHz, DMSO-*d*_6_, δ [ppm]): 171.71, 171.70 q (−CH_2_-N(C_4_H_9_)-CO-(CH_2_)_4_−), 168.9 q (pyrimidine C-2), 167.0 q (pyrimidine C-4,6), 166.8 q (−NH-CO-CH_2_-S−), 164.0, 163.9 q (−CO-NH-OH), 158.2 q (q, ^2^*J* = 36.7 Hz, −OOC-CF_3_), 158.2 q (phenyl C-3), 156.9 q (thiazole C-2), 142.58, 142.55 q (triazole C-4), 141.8 q (phenyl C-1), 141.8, 141.3 q (*N*-hydroxybenzamide C-4), 134.7 (thiazole C-4), 131.7, 131.4 q (*N*-hydroxybenzamide C-1), 131.0 q (thiazole C-5), 129.6 (phenyl C-5), 127.3, 126.3 (*N*-hydroxybenzamide C-3,5), 127.2, 126.9 (*N*-hydroxybenzamide C-2,6), 124.32, 124.28 (triazole C-5), 120.9 (phenyl C-6), 116.1 (pyrimidine C-5), 115.5 (q, ^1^*J* = 289.0 Hz, −OOC-CF_3_), 114.9 (phenyl C-2), 112.5 (phenyl C-4), 61.1 (triazole-CH_2_-O−), 50.0, 47.5 (−CH_2_-N(C_4_H_9_)-CO-(CH_2_)_4_−), 49.3, 49.2 (−CH_2_-N(C_4_H_9_)-CO-(CH_2_)_3_-CH_2_−), 46.8, 45.2 (−CH_2_-N(CH_2_-C_3_H_7_)-CO-(CH_2_)_4_−), 34.1 (−NH-CO-CH_2_-S−), 31.9 (thiazole-CH_2_-phenyl), 31.6, 31.2 (−CH_2_-N(C_4_H_9_)-CO-CH_2_-(CH_2_)_3_−), 30.3, 29.3 (−CH_2_-N(CH_2_-CH_2_-C_2_H_5_)-CO-(CH_2_)_4_−), 29.4, 29.2 (−CH_2_-N(C_4_H_9_)-CO-CH_2_-CH_2_-(CH_2_)_2_−), 23.2 (pyrimidine-CH_3_), 21.9, 21.8 (−CH_2_-N(C_4_H_9_)-CO-(CH_2_)_2_-CH_2_-CH_2_−), 19.6, 19.4 (−CH_2_-N((CH_2_)_2_-CH_2_-CH_3_)-CO-(CH_2_)_4_−), 13.7, 13.6 (−CH_2_-N((CH_2_)_3_-CH_3_)-CO-(CH_2_)_4_−). ^19^F NMR (377 MHz, DMSO-*d*_6_, δ [ppm]): −74.3. HRMS (ESI^+^): *m*/*z* calcd for C_38_H_46_N_9_O_5_S_2_^+^: 772.3058 [M + H]^+^, found: 772.3066. LRMS *m*/*z* (ESI^+^) 772 ([M + H]^+^, 100%). HPLC retention time 16.53 min, >99.5% (M1). ^(*a*)^Rotamers were observed, ^1^H signals of −CONH-OH and F_3_C-COOH could not be detected. ^(^^*b*)^Rotamers were observed.

#### Ethyl 4-(Azidomethyl)benzoate (**34**)^63^

Ethyl 4-(bromomethyl)benzoate (2.2 g, 9.05 mmol, 1 equiv) was dissolved in 15 mL of dimethylformamide (DMF), followed by the addition of sodium azide (0.588 g, 9.05 mmol, 1 equiv). The reaction mixture was stirred at ambient temperature for 16 h. Water (50 mL) was added to the reaction mixture, followed by the extraction of the aqueous layer with EtOAc (3 × 50 mL). The combined organic layer was washed with brine (50 mL), dried over Na_2_SO_4_, filtered, and concentrated under reduced pressure. The residue was purified by flash column chromatography (EtOAc/isohexane: gradient 0–8%) to yield the title compound as a colorless liquid (1.73 g, 93%). ^1^H NMR (400 MHz, DMSO-*d*_*6*_, δ [ppm]): 8.01–7.93 (m, 2H, ethyl benzoate H-2,6), 7.54–7.47 (m, 2H, ethyl benzoate H-3,5), 4.58 (s, 2H, −CH_2_-N_3_), 4.31 (q, 2H, ^3^*J* = 7.1 Hz, H_3_C-CH_2_-O-CO−), 1.32 (t, 3H, ^3^*J* = 7.1 Hz, H_3_C-CH_2_-O-CO−). ^13^C NMR (101 MHz, DMSO-*d*_6_, δ [ppm]): 165.4 q (H_3_C-CH_2_-O-CO−), 141.1 q (ethyl benzoate, C-4), 129.5 (ethyl benzoate C-2,6), 129.5 q (ethyl benzoate C-1), 128.5 (ethyl benzoate C-3,5), 60.8 (H_3_C-CH_2_-O-CO-), 53.0 (−CH_2_-N_3_), 14.2 (H_3_C-CH_2_-O-CO−). LRMS *m*/*z* (ESI^+^): 206 [M + H]^+^. The spectroscopic data are in good agreement with the literature values.^63^

#### Ethyl 4-((4-((3-((2-(2-((4,6-Dimethylpyrimidin-2-yl)thio)acetamido)thiazol-5-yl)methyl)phenoxy)methyl)-1*H*-1,2,3-triazol-1-yl)methyl)benzoate (**36**)

2-((4,6-Dimethylpyrimidin-2-yl)thio)-*N*-(5-(3-(prop-2-yn-1-yloxy)benzyl)thiazol-2-yl)acetamide (**35**, 30.0 mg, 71 μmol) and ethyl 4-(azidomethyl)benzoate (**34**, 16.0 mg, 78 μmol) were dissolved in a water/*tert*-BuOH mixture (1600 μL, 1:1). Tris(benzyltriazolylmethyl)amine (TBTA, 3.6 mg, 7.1 μmol) was dissolved in DMF (800 μL) and added to the mixture. An aqueous CuSO_4_ solution (70.1 μL, 0.1 M) and an aqueous solution of sodium ascorbate (140.3 μL, 0.1 M) were added in that order. The resulting reaction mixture was stirred for 12 h at ambient temperature under nitrogen atmosphere. After completion, water (30 mL) was added to the reaction mixture. The aqueous layer was extracted with EtOAc (3 × 30 mL). The combined organic layer was washed with brine (30 mL), dried over Na_2_SO_4_, and concentrated under reduced pressure. The residue was purified by flash column chromatography (EtOAc/isohexane: gradient 10–100%) to yield the title compound as a colorless solid (37 mg, 83%). ^1^H NMR (400 MHz, DMSO-*d*_6_, δ [ppm]): 12.22 (bs, 1H, −NH-CO-CH_2_-S−), 8.29 (s, 1H, triazole-H), 8.02–7.89 (m, 2H, ethyl benzoate H-2,6), 7.45–7.34 (m, 2H, ethyl benzoate H-3,5), 7.27–7.18 (m, 2H, thiazole H-4, phenyl H-5), 6.92 (s, 1H, pyrimidine H-5), 6.91–6.80 (m, 3H, phenyl H-2,4,6), 5.70 (s, 2H, ethyl benzoate-CH_2_-triazole), 5.11 (s, 2H, triazole-CH_2_-O−), 4.30 (q, 2H, ^3^*J* = 7.1 Hz, H_3_C-CH_2_-O-CO−), 4.08 (s, 2H, −NH-CO-CH_2_-S−), 4.02 (s, 2H, thiazole-CH_2_-phenyl), 2.27 (s, 6H, pyrimidine-CH_3_), 1.30 (t, 3H, ^3^*J* = 7.1 Hz, H_3_C-CH_2_-O-CO−). ^13^C NMR (101 MHz, DMSO-*d*_6_, δ [ppm]): 168.9 q (pyrimidine C-2), 167.0 q (pyrimidine C-4,6), 166.8 q (−NH-CO-CH_2_-S−), 165.3 q (H_3_C-CH_2_-O-CO−), 158.1 q (phenyl C-3), 156.9 q (thiazole C-2), 143.1 q (triazole C-4), 141.9 q (phenyl C-1), 141.2 q (ethyl benzoate, C-4), 134.8 (thiazole C-4), 131.1 q (thiazole C-5), 129.7 (phenyl C-5), 129.6 (ethyl benzoate C-2,6 and ethyl benzoate C-1), 128.1 (ethyl benzoate C-3,5), 124.9 (triazole C-5), 120.9 (phenyl C-6), 116.1 (pyrimidine C-5), 115.0 (phenyl C-2), 112.5 (phenyl C-4), 61.0 (triazole-CH_2_-O−), 60.9 (H_3_C-CH_2_-O-CO−), 52.4 (ethyl benzoate-CH_2_-triazole), 34.1 (−NH-CO-CH_2_-S−), 31.9 (thiazole-CH_2_-phenyl), 23.3 (pyrimidine-CH_3_), 14.2 (H_3_C-CH_2_-O-CO−). HRMS (ESI^+^): *m*/*z* calcd for C_31_H_32_N_7_O_4_S_2_^+^: 630.1952 [M + H]^+^, found: 630.1950. LRMS *m*/*z* (ESI^+^): 630 [M + H]^+^.

#### Ethyl 4-(Aminomethyl)benzoate (**37**)^64^

Ethyl 4-(azidomethyl)benzoate (**34**, 1.4 g, 6.82 mmol, 1 equiv) was dissolved in a mixture of 23 mL tetrahydrofuran (THF) and 4.4 mL water. Triphenylphospine (5.37 g, 29.47 mmol, 3 equiv) was added to the mixture followed by stirring at ambient temperature for 1 h and further at 75 °C for 1 h. The reaction mixture was ice-cooled, and water was added (30 mL). The mixture was adjusted to pH 2 by adding 1 M HCl and was subsequently washed with TBME. Then, the aqueous layer was neutralized with saturated aqueous sodium bicarbonate, followed by the extraction with EtOAc (3 × 30 mL). The combined organic layer was washed with brine (50 mL), dried over Na_2_SO_4_, filtered, and concentrated under reduced pressure. The title compound was obtained as a pale-yellow oil (1.18 g, 97%) and was used for subsequent reactions without any further purification. ^1^H NMR (400 MHz, DMSO-*d*_6_, δ [ppm]): 7.94–7.83 (m, 2H, ethyl benzoate H-2,6), 7.51–7.43 (m, 2H, ethyl benzoate H-3,5), 4.29 (q, 2H, ^3^*J* = 7.1 Hz, H_3_C-CH_2_-O-CO−), 3.78 (s, 2H, −CH_2_-NH_2_), 1.86 (bs, −CH_2_-NH_2_), 1.30 (t, 3H, ^3^*J* = 7.1 Hz, H_3_C-CH_2_-O-CO−). ^13^C NMR (101 MHz, DMSO-*d*_6_, δ [ppm]): 165.8 q (H_3_C-CH_2_-O-CO−), 150.1 q (ethyl benzoate, C-4), 129.0 (ethyl benzoate C-2,6), 127.8 q (ethyl benzoate C-1), 127.2 (ethyl benzoate C-3,5), 60.5 (H_3_C-CH_2_-O-CO−), 45.4 (−CH_2_-NH_2_), 14.2 (H_3_C-CH_2_-O-CO−). LRMS *m*/*z* (ESI^+^): 180 [M + H]^+^. The spectroscopic data are in good agreement with the literature values.^64^ The attached ^1^H and ^13^C NMR spectra contains residual solvent signals (EtOAc).

#### Ethyl 4-((5-Azidopentanamido)methyl)benzoate (**38**)

5-Azidopentanoic acid (98.0 mg, 685 μmol) was dissolved in DMF (1 mL). *O*-(7-Azabenzotriazol-1-yl)-*N*,*N*,*N*′,*N*′-tetramethyluronium-hexafluorphosphat (HATU, 651 mg, 1.71 mmol) dissolved in DMF (2 mL), *N*,*N*-diisopropylethylamine (DIPEA, 232 μL, 1.37 mmol), and ethyl 4-(aminomethyl)benzoate (**37**, 147.3 mg, 822 μmol) dissolved in DMF (2 mL) were added under nitrogen atmosphere and ice-cooling in this order. After 5 min of stirring at 0 °C, further DIPEA (347 μL, 2.05 mmol) was added under nitrogen atmosphere and ice-cooling. The reaction mixture was allowed to warm up to ambient temperature and was stirred for further 16 h. Then, the reaction mixture was partitioned between water (50 mL) and EtOAc (50 mL). The organic layer was washed with 1 M HCl (2 × 30 mL), saturated sodium bicarbonate solution (50 mL), water (50 mL), brine (50 mL), dried over Na_2_SO_4_, filtered, and concentrated under reduced pressure. The residue was purified by flash column chromatography (EtOAc/isohexane: gradient 10–80%) to yield the title compound as a pale-red solid (205 mg, 98%). ^1^H NMR (400 MHz, DMSO-*d*_6_, δ [ppm]): 8.44 (t, 1H, ^3^*J* = 5.9 Hz, −CH_2_-NH-CO-(CH_2_)_4_-N_3_), 7.97–7.86 (m, 2H, ethyl benzoate H-2,6), 7.41–7.33 (m, 2H, ethyl benzoate H-3,5), 4.38–4.23 (m, 4H, −CH_2_-NH-CO-(CH_2_)_4_-N_3_, H_3_C-CH_2_-O-CO−), 3.33* (t, 2H, ^3^*J* = 6.5 Hz, −CH_2_-NH-CO-(CH_2_)_3_-CH_2_-N_3_), 2.19 (t, 2H, ^3^*J* = 7.1 Hz, −CH_2_-NH-CO-CH_2_-(CH_2_)_3_-N_3_), 1.67–1.42 (m, 4H, −CH_2_-NH-CO-CH_2_-CH_2_-CH_2_-CH_2_-N_3_), 1.30 (t, 3H, ^3^*J* = 7.1 Hz, H_3_C-CH_2_O-CO−). ^13^C NMR (101 MHz, DMSO-*d*_6_, δ [ppm]): 172.0 q (−CH_2_-NH-CO-(CH_2_)_4_-N_3_), 165.6 q (H_3_C-CH_2_-O-CO−), 145.4 q (ethyl benzoate, C-4), 129.2 (ethyl benzoate C-2,6), 128.4 q (ethyl benzoate C-1), 127.3 (ethyl benzoate C-3,5), 60.7 (H_3_C-CH_2_-O-CO−), 50.4 (−CH_2_-NH-CO-(CH_2_)_3_-CH_2_-N_3_), 41.8 (−CH_2_-NH-CO-(CH_2_)_4_-N_3_), 34.7 (−CH_2_-NH-CO-CH_2_-(CH_2_)_3_-N_3_), 27.9 (−CH_2_-NH-CO-(CH_2_)_2_-CH_2_-CH_2_-N_3_), 22.5 (−CH_2_-NH-CO-CH_2_-CH_2_-(CH_2_)_2_-N_3_), 14.2 (H_3_C-CH_2_-O-CO−). HRMS (ESI^+^): *m*/*z* calcd for C_15_H_21_N_4_O_3_^+^: 305.1608 [M + H]^+^, found: 305.1614. LRMS *m*/*z* (ESI^+^): 305 [M + H]^+^. The attached ^1^H and ^13^C NMR spectra contains residual solvent signals (EtOAc).

#### Ethyl 4-((5-(4-((3-((2-(2-((4,6-Dimethylpyrimidin-2-yl)thio)acetamido)thiazol-5-yl)methyl)phenoxy)methyl)-1*H*-1,2,3-triazol-1-yl)pentanamido)methyl)benzoate (**39**)

2-((4,6-Dimethylpyrimidin-2-yl)thio)-*N*-(5-(3-(prop-2-yn-1-yloxy)benzyl)thiazol-2-yl)acetamide (**35**, 30.0 mg, 71 μmol) and ethyl 4-((5-azidopentanamido)methyl)benzoate (**38**, 23.7 mg, 78 μmol) were dissolved in a water/*tert*-BuOH mixture (1600 μL, 1:1). Tris(benzyltriazolylmethyl)amine (TBTA, 3.6 mg, 7.1 μmol) was dissolved in DMF (800 μL) and added to the mixture. An aqueous CuSO_4_ solution (70.1 μL, 0.1 M) and an aqueous solution of sodium ascorbate (140.3 μL, 0.1 M) were added in that order. The resulting reaction mixture was stirred for 12 h at room temperature under nitrogen atmosphere. After completion, water (30 mL) was added to the reaction mixture. The aqueous layer was extracted with EtOAc (3 × 30 mL). The combined organic layer was washed with brine (30 mL), dried over Na_2_SO_4_, filtered, and concentrated under reduced pressure. The residue was purified by flash column chromatography (MeOH/dichloromethane: gradient 0–8%) to yield the title compound as a colorless solid (46 mg, 89%). ^1^H NMR (400 MHz, DMSO-*d*_6_, δ [ppm]): 12.22 (bs, 1H, −NH-CO-CH_2_-S−), 8.44 (t, 1H, ^3^*J* = 6.0 Hz, −CH_2_-NH-CO-(CH_2_)_4_-triazole), 8.19 (s, 1H, triazole-H), 7.93–7.87 (m, 2H, ethyl benzoate H-2,6), 7.41–7.32 (m, 2H, ethyl benzoate H-3,5), 7.28–7.17 (m, 2H, thiazole H-4, phenyl H-5), 6.93 (s, 1H, pyrimidine H-5), 6.92–6.81 (m, 3H, phenyl H-2,4,6), 5.09 (s, 2H, triazole-CH_2_-O−), 4.44–4.24 (m, 6H, −CH_2_-NH-CO-(CH_2_)_3_-CH_2_-triazole, -CH_2_-NH-CO-(CH_2_)_4_-triazole, H_3_C-CH_2_-O-CO−), 4.08 (s, 2H, −NH-CO-CH_2_-S−), 4.03 (s, 2H, thiazole-CH_2_-phenyl), 2.27 (s, 6H, pyrimidine-CH_3_), 2.20 (t, 2H, ^3^*J* = 7.4 Hz, −CH_2_-NH-CO-CH_2_-(CH_2_)_3_-triazole), 1.89–1.73 (m, 2H, −CH_2_-NH-CO-(CH_2_)_2_-CH_2_-CH_2_-triazole), 1.55–1.44 (m, 2H, −CH_2_-NH-CO=CH_2_-CH_2_-(CH_2_)_2_-triazole), 1.30 (t, 3H, ^3^*J* = 7.1 Hz, H_3_C-CH_2_-O-CO−). ^13^C NMR (101 MHz, DMSO-*d*_6_, δ [ppm]): 171.9 q (−CH_2_-NH-CO-(CH_2_)_4_-triazole), 168.9 q (pyrimidine C-2), 167.0 q (pyrimidine C-4,6), 166.8 q (−NH-CO-CH_2_-S−), 165.6 q (H_3_C-CH_2_=O-CO−), 158.2 q (phenyl C-3), 156.9 q (thiazole C-2), 145.3 q (ethyl benzoate, C-4), 142.6 q (triazole C-4), 141.9 q (phenyl C-1), 134.8 (thiazole C-4), 131.1 q (thiazole C-5), 129.7 (phenyl C-5), 129.2 (ethyl benzoate C-2,6), 128.4 q (ethyl benzoate C-1), 127.3 (ethyl benzoate C-3,5), 124.4 (triazole C-5), 120.9 (phenyl C-6), 116.1 (pyrimidine C-5), 115.0 (phenyl C-2), 112.4 (phenyl C-4), 61.1 (triazole-CH_2_-O−), 60.7 (H_3_C-CH_2_-O-CO−), 49.1 (−CH_2_-NH-CO-(CH_2_)_3_-CH_2_-triazole), 41.8 (−CH_2_-NH-CO-(CH_2_)_4_-triazole), 34.5 (−CH_2_-NH-CO-CH_2_-(CH_2_)_3_-triazole), 34.1 (−NH-CO-CH_2_-S−), 31.9 (thiazole-CH_2_-phenyl), 29.4 (−CH_2_-NH-CO-(CH_2_)_2_-CH_2_-CH_2_-triazole), 23.3 (pyrimidine-CH_3_), 22.2 (−CH_2_-NH-CO-CH_2_-CH_2_-(CH_2_)_2_-triazole), 14.2 (H_3_C-CH_2_-O-CO−). HRMS (ESI^+^): *m*/*z* calcd for C_36_H_41_N_8_O_5_S_2_^+^: 729.2636 [M + H]^+^, found: 729.2633. LRMS *m*/*z* (ESI^+^): 729 [M + H]^+^.

#### Ethyl 4-((Butylamino)methyl)benzoate (**40**)

Butylamine (4.07 mL, 41.14 mmol) was added to a solution of ethyl 4-(bromomethyl)benzoate (2.0 g, 8.23 mmol) in THF (20 mL). After 3 h of stirring at ambient temperature, the reaction mixture was partitioned between chloroform (20 mL) and a saturated sodium bicarbonate solution (20 mL). The organic layer was dried over Na_2_SO_4_, filtered, and concentrated under reduced pressure. The title compound was obtained as a tan oil and was used for subsequent reactions without further purification (1.91 g, 98%). ^1^H NMR (400 MHz, DMSO-*d*_6_, δ [ppm]): 7.92–7.86 (m, 2H, ethyl benzoate H-2,6), 7.49–7.42 (m, 2H, ethyl benzoate H-3,5), 4.29 (q, 2H, ^3^*J* = 7.2 Hz, H_3_C-CH_2_-O-CO−), 3.74 (s, 2H, −CH_2_-NH-(CH_2_)_3_-CH_3_), 2.45 (t, 2H, ^3^*J* = 7.0 Hz, −NH-CH_2_-(CH_2_)_2_-CH_3_), 2.15 (bs, 1H, −NH-CH_2_-(CH_2_)_2_-CH_3_), 1.46–1.24 (m, 7H, −NH-CH_2_-(CH_2_)_2_-CH_3_, H_3_C-CH_2_-O-CO−), 0.85 (t, 3H, ^3^*J* = 7.2 Hz, −NH-(CH_2_)_3_-CH_3_). ^13^C NMR (151 MHz, DMSO-*d*_6_, δ [ppm]): 165.7 q (H_3_C-CH_2_-O-CO−), 147.1 q (ethyl benzoate, C-4), 128.9 (ethyl benzoate C-2,6), 128.1 q (ethyl benzoate C-1), 127.9 (ethyl benzoate C-3,5), 60.5 (H_3_C-CH_2_-O-CO−), 52.7 (−NH-CH_2_-(CH_2_)_2_-CH_3_), 48.4 (−CH_2_-NH-(CH_2_)_3_-CH_3_), 31.7 (−NH-CH_2_-CH_2_-CH_2_-CH_3_), 20.0 (−NH-CH_2_-CH_2_-CH_2_-CH_3_), 14.2 (H_3_C-CH_2_-O-CO−), 13.9 (−NH-CH_2_-CH_2_-CH_2_-CH_3_); HRMS (ESI^+^): *m*/*z* calcd for C_14_H_22_NO_2_^+^: 236.1645 [M + H]^+^, found: 236.1645. LRMS *m*/*z* (ESI^+^): 236 [M + H]^+^.

#### Ethyl 4-((5-Azido-*N*-butylpentanamido)methyl)benzoate (**42**)

5-Bromopentanoic (54.3 mg, 0.3 mmol) was dissolved in 4 mL of a mixture of dichloromethane and DMF (1:1 (v/v)). The solution was cooled to 0 °C. 2-(1*H*-Benzotriazole-1-yl)-1,1,3,3-tetramethylaminium tetrafluoroborate (TBTU, 144.5 mg, 0.45 mmol) and DIPEA (78.4 μL, 0.45 mmol) were dissolved in DMF (1.5 mL). This mixture was added dropwise at 0 °C to the flask containing the solution of 5-bromopentanoic. After addition, the mixture was stirred for 15 min at 0 °C. Then, ethyl 4-((butylamino)methyl)benzoate (**40**, 70.6 mg, 0.3 mmol) dissolved in 1.5 mL of DMF was added to the reaction mixture at 0 °C. After 2 h of stirring at ambient temperature, TLC indicated completion of the reaction (*R*_*f*_ = 0.42, isohexane/EtOAc 7:3). Then, 30 mL of water were added to the reaction mixture. The aqueous layer was extracted with EtOAc (3 × 30 mL). The combined organic layer was dried over Na_2_SO_4_, filtered, and concentrated under reduced pressure. The residue was purified by flash column chromatography (EtOAc/isohexane: gradient 4–30%) to yield 84 mg of ethyl 4-((5-bromo-*N*-butylpentanamido)methyl)benzoate (**41**) as a colorless liquid (LRMS *m*/*z* (ESI^+^): 398 ([M(^79^Br + H]^+^), 400 ([M(^81^Br + H]^+^). The obtained intermediate 4-((5-bromo-*N*-butylpentanamido)methyl)benzoate (**41**, 63.0 mg, 0.158 mmol) and NaN_3_ (15.4 mg, 0.237 mmol) were dissolved in DMSO (2.5 mL) and stirred overnight at 45 °C. After addition of water (20 mL), the aqueous layer was extracted with EtOAc (3 × 20 mL). The combined organic layer was dried over Na_2_SO_4_, filtered, and concentrated under reduced pressure. The residue was purified by flash column chromatography (EtOAc/isohexane: gradient 4–32%) to yield the title compound as a colorless solid (46 mg, 56% over two steps). ^1^H NMR^a^ (400 MHz, DMSO-*d*-_6_, δ [ppm]): 7.98–7.88 (m, 2H, ethyl benzoate H-2,6), 7.36–7.30 (m, 2H, ethyl benzoate H-3,5), 4.65, 4.57 (2*s*, 2H, −CH_2_-N(C_4_H_9_)-CO-(CH_2_)_4_-N_3_), 4.30, 4.29 (2*q*, 2H, ^3^*J* = 7.0 Hz, H_3_C-CH_2_-O-CO−), 3.37–3.18* (m, 4H, −CH_2_-N(CH_2_-C_3_H_7_)-CO-(CH_2_)_3_-CH_2_-N_3_), 2.44, 2.27 (2*t*, 2H, ^3^*J* = 7.0 Hz, −CH_2_-N(C_4_H_9_)-CO-CH_2_-(CH_2_)_3_-N_3_), 1.67–1.14 (m, 11H, H_3_C-CH_2_-O-CO-, −CH_2_-N(CH_2_-(CH_2_)_2_-CH_3_)-CO-CH_2_-(CH_2_)_2_-CH_2_-N_3_)), 0.85, 0.84 (2*t*, 3H, ^3^*J* = 7.3 Hz, −CH_2_-N((CH_2_)_3_-CH_3_)-CO-(CH_2_)_4_-N_3_). ^13^C NMR^a^ (101 MHz, DMSO-*d*_*6*_, δ [ppm]): 171.9 q (−CH_2_-N(C_4_H_9_)-CO-(CH_2_)_4_-N_3_), 165.6, 165.5 q (H_3_C-CH_2_-O-CO−), 144.3, 143.8 q (ethyl benzoate, C-4), 129.6, 129.2 (ethyl benzoate C-2,6), 128.8, 128.5 q (ethyl benzoate C-1), 127.5, 126.7 (ethyl benzoate C-3,5), 60.7, 60.7 (H_3_C-CH_2_-O-CO−), 50.54, 50.45 (−CH_2_-N(C_4_H_9_)-CO-(CH_2_)_3_-CH_2_-N_3_), 50.1, 47.7 (−CH_2_-N(C_4_H_9_)-CO-(CH_2_)_4_-N_3_), 47.0, 45.4 (−CH_2_-N(CH_2_-C_3_H_7_)-CO-(CH_2_)_4_-N_3_), 31.7, 31.4 (−CH_2_-N(C_4_H_9_)-CO-CH_2_-(CH_2_)_3_-N_3_), 30.4, 29.3 (−CH_2_-N(CH_2_-CH_2_-C_2_H_5_)-CO-(CH_2_)_4_-N_3_), 28.0, 27.8 (−CH_2_-N(C_4_H_9_)-CO-CH_2_-CH_2_-(CH_2_)_2_-N_3_), 22.2, 22.0 (−CH_2_-N(C_4_H_9_)-CO-(CH_2_)_2_-CH_2_-CH_2_-N_3_), 19.6, 19.5 (−CH_2_-N((CH_2_)_2_-CH_2_-CH_3_)-CO-(CH_2_)_4_-N_3_), 14.2 (H_3_C-CH_2_-O-CO−), 13.8, 13.7 (−CH_2_-N((CH_2_)_3_-CH_3_)-CO-(CH_2_)_4_-N_3_). HRMS (ESI^+^): *m*/*z* calcd for C_19_H_29_N_4_O_3_^+^: 361.2234 [M + H]^+^, found: 361.2239; LRMS *m*/*z* (ESI^+^) 361 ([M + H]^+^, 100%). ^(*a*)^Rotamers were observed.

#### Ethyl 4-((*N*-Butyl-5-(4-((3-((2-(2-((4,6-dimethylpyrimidin-2-yl)thio)acetamido)thiazol-5-yl)methyl)phenoxy)methyl)-1*H*-1,2,3-triazol-1-yl)pentanamido)methyl)benzoate (**43**)

2-((4,6-Dimethylpyrimidin-2-yl)thio)-*N*-(5-(3-(prop-2-yn-1-yloxy)benzyl)thiazol-2-yl)acetamide (**35**, 26.0 mg, 61 μmol) and ethyl 4-((5-azido-*N*-butylpentanamido)methyl)benzoate (**42**, 22.1 mg, 61 μmol) were dissolved in a water/*tert*-BuOH mixture (1600 μL, 1:1). Tris(benzyltriazolylmethyl)amine (TBTA, 3.3 mg, 6.1 μmol) was dissolved in DMF (800 μL) and added to the mixture. An aqueous CuSO_4_ solution (60.8 μL, 0.1 M) and an aqueous solution of sodium ascorbate (121.6 μL, 0.1 M) were added in that order. The resulting reaction mixture was stirred overnight at ambient temperature under nitrogen atmosphere. After completion, water (30 mL) was added to the reaction mixture. The aqueous layer was extracted with EtOAc (3 × 30 mL). The combined organic layer was washed with brine (30 mL), dried over Na_2_SO_4_, filtered, and concentrated under reduced pressure. The residue was purified by flash column chromatography (MeOH/dichloromethane: gradient 0.4–5%) to yield the title compound as a colorless solid (42 mg, 88%). ^1^H NMR^a^ (400 MHz, DMSO-*d*_6_, δ [ppm]): 12.22 (bs, 1H, −NH-CO-CH_2_-S−), 8.21, 8.16 (2*s*, 1H, triazole-H), 7.98–7.87 (m, 2H, ethyl benzoate H-2,6), 7.38–7.17 (m, 4H, ethyl benzoate H-3,5, thiazole H-4, phenyl H-5), 6.95–6.78 (m, 4H, pyrimidine H-5, phenyl H-2,4,6), 5.09, 5.07 (2*s*, 2H, triazole-CH_2_-O−), 4.63, 4.56 (2*s*, 2H, −CH_2_-N(C_4_H_9_)-CO-(CH_2_)_4_−), 4.41–4.24 (m, 4H, −CH_2_-N(C_4_H_9_)-CO-(CH_2_)_3_-CH_2_-triazole, H_3_C-CH_2_-O-CO−), 4.08 (s, 2H, −NH-CO-CH_2_-S−), 4.03 (s, 2H, thiazole-CH_2_-phenyl), 3.27–3.15 (m, 2H, −CH_2_-N(CH_2_-C_3_H_7_)-CO-(CH_2_)_4_−), 2.47–2.19 (m, 8H, −CH_2_-N(C_4_H_9_)-CO-CH_2_-(CH_2_)_3_–, pyrimidine-CH_3_), 1.97–1.66 (m, 2H, −CH_2_-N(C_4_H_9_)-CO-(CH_2_)_2_-CH_2_-CH_2_−), 1.56–1.35 (m, 4H, −CH_2_-N(CH_2_-CH_2_-C_2_H_5_)-CO-CH_2_-CH_2_-(CH_2_)_2_−), 1.31, 1.30 (2*t*, 3H, ^3^*J* = 7.5 Hz, H_3_C-CH_2_-O-CO−), 1.25–1.11 (m, 2H, −CH_2_-N((CH_2_)_2_-CH_2_-CH_3_)-CO-(CH_2_)_4_−), 0.83, 0.82 (2*t*, 3H, ^3^*J* = 7.2 Hz, −CH_2_-N((CH_2_)_3_-CH_3_)-CO-(CH_2_)_4_−). ^13^C NMR^a^ (151 MHz, DMSO-*d*_6_, δ [ppm]): 171.8 q (−CH_2_-N(C_4_H_9_)-CO-(CH_2_)_4_−), 168.9 q (pyrimidine C-2), 167.0 q (pyrimidine C-4,6), 166.8 q (−NH-CO-CH_2_-S−), 165.54, 165.46 q (H_3_C-CH_2_-O-CO−), 158.2 q (phenyl C-3), 156.9 q (thiazole C-2), 144.2, 143.7 q (ethyl benzoate, C-4), 142.6, 142.5 q (triazole C-4), 141.8 q (phenyl C-1), 134.8 (thiazole C-4), 131.0 q (thiazole C-5), 129.6 (phenyl C-5), 129.5, 129.2 (ethyl benzoate C-2,6), 128.8, 128.5 q (ethyl benzoate C-1), 127.5, 126.6 (ethyl benzoate C-3,5), 124.3, 124.3 (triazole C-5), 120.9 (phenyl C-6), 116.1 (pyrimidine C-5), 114.9 (phenyl C-2), 112.4 (phenyl C-4), 61.1 (triazole-CH_2_-O−), 60.7, 60.5 (H_3_C-CH_2_-O-CO−), 50.1, 47.7 (−CH_2_-N(C_4_H_9_)-CO-(CH_2_)_4_−), 49.3, 49.2 (−CH_2_-N(C_4_H_9_)-CO-(CH_2_)_3_-CH_2_−), 46.9, 45.3 (−CH_2_-N(CH_2_-C_3_H_7_)-CO-(CH_2_)_4_−), 34.0 (−NH-CO-CH_2_-S−), 31.9 (thiazole-CH_2_-phenyl), 31.6, 31.2 (−CH_2_-N(C_4_H_9_)-CO-CH_2_-(CH_2_)_3_−), 30.3, 29.2 (−CH_2_-N(CH_2_-CH_2_-C_2_H_5_)-CO-(CH_2_)_4_−), 29.3, 29.2 (−CH_2_-N(C_4_H_9_)-CO-CH_2_-CH_2_-(CH_2_)_2_−), 23.2 (pyrimidine-CH_3_), 21.88, 21.7 (−CH_2_-N(C_4_H_9_)-CO-(CH_2_)_2_-CH_2_-CH_2_−), 19.6, 19.4 (−CH_2_-N((CH_2_)_2_-CH_2_-CH_3_)-CO-(CH_2_)_4_−), 14.2 (H_3_C-CH_2_-O-CO−), 13.7, 13.6 (−CH_2_-N((CH_2_)_3_-CH_3_)-CO-(CH_2_)_4_−). HRMS (ESI^+^): *m*/*z* calcd for C_40_H_49_N_8_O_5_S_2_^+^: 785.3262 [M + H]^+^, found: 785.3271. LRMS *m*/*z* (ESI^+^): 785 [M + H]^+^. ^(*a*)^Rotamers were observed.

#### 4-((4-(1-(4-((3-((2-(2-((4,6-Dimethylpyrimidin-2-yl)thio)acetamido)thiazol-5-yl)methyl)phenoxy)methyl)-1*H*-1,2,3-triazol-1-yl)-3,6,9,12-tetraoxapentadecan-15-amido)benzamido)methyl)-*N*-hydroxybenzamide (**44**)

After swelling of the resin **49** (0.10 mmol, 1.00 equiv) in DMF for 30 min, Fmoc deprotection was performed by treatment with 20% piperidine in DMF (1.5 mL, 2 × 5 min). Afterward, the resin was washed with DMF (5 × 2 mL), CH_2_Cl_2_ (5 × 2 mL), and DMF (5 × 2 mL). For the subsequent amide coupling reaction, a solution of N_3_-PEG_4_-COOH (87.0 mg, 0.30 mmol, 3.00 equiv), HATU (114 mg, 0.30 mmol, 3.00 equiv), and DIPEA (70.0 μL, 0.40 mmol, 4.00 equiv) in DMF (500 μL) was agitated for 5 min and then added to the resin. The reaction was shaken for 5 h at ambient temperature followed by washing with DMF (5 × 2 mL), CH_2_Cl_2_ (5 × 2 mL), and DMF (5 × 2 mL). The obtained azido-functionalized preferential HDAC6i precursor resin **52** was directly used for the subsequent reaction. For the Huisgen cycloaddition, a solution of SirReal-alkyne **35** (85.0 mg, 0.20 mmol, 2.00 equiv) in DMF (1.00 mL), TBTA (13.2 mg, 25.0 μmol, 0.25 equiv), 0.1 M aqueous CuSO_4_·5H_2_O (252 μL, 25.0 μmol, 0.25 equiv) in DMF (0.50 mL), 0.2 M aqueous ascorbic acid (252 μL, 50.0 μmol, 0.50 equiv) in DMF (0.50 mL), and *t*-BuOH (990 μL) were added in that sequence to the resin. The reaction was shaken for 18 h at ambient temperature. After washing with DMF (5 × 2 mL) and CH_2_Cl_2_ (5 × 2 mL), the resin was dried under reduced pressure followed by the cleavage of the crude product from the resin by treatment with cleavage solution (5% TFA in CH_2_Cl_2_, 1 mL/40 mg resin) for 1 h at ambient temperature. The filtrates were concentrated under reduced pressure, and the crude final product was purified by preparative HPLC (gradient elution from 5% to 95% acetonitrile (+0.1% TFA)/water (+0.1% TFA)). Lyophilization of the respective fractions yielded the title compound (47.5 mg, 43.3 μmol, 43%) as a TFA salt. ^1^H NMR^a^ (400 MHz, DMSO-*d*_6_, δ [ppm]): 12.20 (bs, 1H, −NH-CO-CH_2_-S−), 11.16 (bs, 1H, −CO-NH-OH), 10.15 (s, 1H, phenyl-NH-CO−), 8.95 (t, ^3^*J* = 6.0 Hz, 1H, *N*-hydroxybenzamide-CH_2_-NH-CO−), 8.15 (s, 1H, triazole-H), 7.95–7.82 (m, 2H, 4-acylamidobenzamide H-2,6), 7.75–7.61 (m, 4H, *N*-hydroxybenzamide H-2,6, 4-acylamidobenzamide H-3,5), 7.40–7.32 (m, 2H, *N*-hydroxybenzamide H-3,5), 7.26–7.19 (m, 2H, thiazole H-4, phenyl H-5), 6.93 (s, 1H, pyrimidine H-5), 6.91–6.86 (m, 2H, phenyl H-2,4), 6.83 (dt, ^3^*J* = 7.6, ^4^*J* = 1.2 Hz, 1H, phenyl H-6), 5.09 (s, 2H, −triazole-CH_2_-O−), 4.56–4.41 (m, 4H, *N*-hydroxybenzamide-CH_2_-NH-CO–, −O-CH_2_-CH_2_-triazole−), 4.08 (s, 2H, −NH-CO-CH_2_-S−), 4.03 (s, 2H, −thiazole-CH_2_-phenyl−), 3.82–3.76* (m, 2H, −O-CH_2_-CH_2_-triazole−), 3.68* (t, ^3^*J* = 6.2 Hz, 2H, −NH-CO-CH_2_-CH_2_-O−), 3.51–3.37 (m, 12H, 3 × −O-CH_2_-CH_2_-O−), 2.56 (t, ^3^*J* = 6.2 Hz, 2H, −NH-CO-CH_2_-CH_2_-O−), 2.28 (s, 6H, pyrimidine −CH_3_). ^13^C NMR (101 MHz, DMSO-*d*_6_, δ [ppm]): 169.7 q (−NH-CO-CH_2_-CH_2_-O−), 168.9 q (pyrimidine C-2), 167.0 q (pyrimidine C-4,6), 166.8 q (−NH-CO-CH_2_-S−), 165.8 q (*N*-hydroxybenzamide-CH_2_-NH-CO−), 164.1 q (−CO-NH-OH), 158.2 q (phenyl C-3), 156.9 q (thiazole C-2), 143.1 q (*N*-hydroxybenzamide C-4), 142.5 q (triazole C-4), 141.9 q (4-acylamidobenzamide C-4), 141.8 q (phenyl C-1), 134.7 (thiazole C-4), 131.3 q (*N*-hydroxybenzamide C-1), 131.1 q (thiazole C-5), 129.6 (phenyl C-5), 128.5 q (4-acylamidobenzamide C-1), 128.1 (4-acylamidobenzamide C-2,6), 127.1 (*N*-hydroxybenzamide C-3,5), 126.9 (*N*-hydroxybenzamide C-2,6), 124.9 (triazole C-5), 120.9 (phenyl C-6), 118.2 (4-acylamidobenzamide C-3,5), 116.1 (pyrimidine C-5), 114.9 (phenyl C-2), 112.4 (phenyl C-4), 69.7 (−O-CH_2_-CH_2_-O−), 69.7 (−O-CH_2_-CH_2_-O−), 69.7 (−O-CH_2_-CH_2_-O−), 69.6 (−O-CH_2_-CH_2_-O−), 69.6 (−O-CH_2_-CH_2_-O−), 69.5 (−O–CH_2_-CH_2_-O−), 68.6 (−O-CH_2_-CH_2_-triazole−), 66.5 (−NH-CO-CH_2_-CH_2_-O−), 61.0 (−triazole-CH_2_-O−), 49.4 (−O-CH_2_-CH_2_-triazole−), 42.4 (*N*-hydroxybenzamide-CH_2_-NH-CO−), 37.2 (−NH-CO-CH_2_-CH_2_-O−), 34.1 (−NH-CO-CH_2_-S−), 31.9 (thiazole-CH_2_-phenyl), 23.2 (pyrimidine −CH_3_). ^19^F NMR (377 MHz, DMSO-*d*_6_, δ [ppm]): −74.9. HRMS (ESI^+^): *m*/*z* calcd for C_47_H_55_N_10_O_10_S_2_^+^: 983.3539 [M + H]^+^, found: 983.3536. HPLC retention time 16.04 min, 97.4% (M3). ^(*a*)1^H signals of −CONH-OH and F_3_C-COOH could not be detected. ^(^^*b*)13^C signals of F_3_C-COOH could not be detected.

#### 4-((4-(1-(4-((3-((2-(2-((4,6-Dimethylpyrimidin-2-yl)thio)acetamido)thiazol-5-yl)methyl)phenoxy)methyl)-1*H*-1,2,3-triazol-1-yl)-3,6,9,12,15,18,21,24,27,30-decaoxatritriacontan-33-amido)benzamido)methyl)-*N*-hydroxybenzamide (**45**)

After swelling of the resin **49** (0.10 mmol, 1.00 equiv) in DMF for 30 min, Fmoc deprotection was performed by treatment with 20% piperidine in DMF (1.5 mL, 2 × 5 min). Afterward, the resin was washed with DMF (5 × 2 mL), CH_2_Cl_2_ (5 × 2 mL), and DMF (5 × 2 mL). For the subsequent amide coupling reaction, a solution of N_3_-PEG_10_-COOH (100 mg, 0.18 mmol, 1.80 equiv), HATU (114 mg, 0.30 mmol, 3.00 equiv), and DIPEA (70.0 μL, 0.40 mmol, 4.00 equiv) in DMF (500 μL) was agitated for 5 min and then added to the resin. The reaction was shaken for 5 h at ambient temperature followed by washing with DMF (5 × 2 mL), CH_2_Cl_2_ (5 × 2 mL), and DMF (5 × 2 mL). The obtained azido-functionalized preferential HDAC6i precursor resin **53** was directly used for the subsequent reaction. For the Huisgen cycloaddition solutions of SirReal-alkyne **35** (85.0 mg, 0.20 mmol, 2.00 equiv) in DMF (1.00 mL), TBTA (13.2 mg, 25.0 μmol, 0.25 equiv), 0.1 M aqueous CuSO_4_·5H_2_O (252 μL, 25.0 μmol, 0.25 equiv) in DMF (0.50 mL), 0.2 M aqueous ascorbic acid (252 μL, 50.0 μmol, 0.50 equiv) in DMF (0.50 mL), and *tert*-BuOH (990 μL) were added in that sequence to the resin. The reaction was shaken for 18 h at ambient temperature. After washing with DMF (5 × 2 mL) and CH_2_Cl_2_ (5 × 2 mL), the resin was dried under reduced pressure followed by the cleavage of the crude product from the resin by treatment with cleavage solution (5% TFA in CH_2_Cl_2_, 1 mL/40 mg resin) for 1 h at ambient temperature. The filtrates were concentrated under reduced pressure, and the crude final product was purified by preparative HPLC (gradient elution from 5% to 95% acetonitrile (+0.1% TFA)/water (+0.1% TFA)). Lyophilization of the respective fractions yielded the title compound (37.0 mg, 27,2 μmol, 27%) as a TFA salt. ^1^H NMR^a^ (400 MHz, DMSO-*d*_6_, δ [ppm]): 12.22 (bs, 1H, −NH-CO-CH_2_-S−), 11.18 (bs, 1H, −CO-NH-OH), 10.18 (s, 1H, phenyl-NH-CO−), 8.97 (t, ^3^*J* = 6.0 Hz, 1H, *N*-hydroxybenzamide-CH_2_-NH-CO−), 8.17 (s, 1H, triazole-H), 7.92–7.78 (m, 2H, 4-acylamidobenzamide H-2,6), 7.78–7.60 (m, 4H, *N*-hydroxybenzamide H-2,6,4-acylamidobenzamide H-3,5), 7.44–7.31 (m, 2H, *N*-hydroxybenzamide H-3,5), 7.28–7.15 (m, 2H, thiazole H-4, phenyl H-5), 6.94 (s, 1H, pyrimidine H-5), 6.92–6.87 (m, 2H, phenyl H-2,4), 6.83 (dt, ^3^*J* = 7.7, ^4^*J* = 1.2 Hz, 1H, phenyl H-6), 5.09 (s, 2H, −triazole-CH_2_-O−), 4.64–4.35 (m, 4H, *N*-hydroxybenzamide-CH_2_-NH-CO–, −O-CH_2_-CH_2_-triazole−), 4.08 (s, 2H, −NH-CO-CH_2_-S−), 4.03 (s, 2H, −thiazole-CH_2_-phenyl−), 3.90–3.75* (m, 2H, −O-CH_2_-CH_2_-triazole−), 3.70* (t, ^3^*J* = 6.2 Hz, 2H, −NH-CO-CH_2_-CH_2_-O−), 3.59–3.32 (m, 36H, 9 × −O-CH_2_-CH_2_-O−), 2.58 (t, ^3^*J* = 6.2 Hz, 2H, -NH-CO-CH_2_-CH_2_-O−), 2.28 (s, 6H, pyrimidine −CH_3_). ^13^C NMR^b^ (151 MHz, DMSO-*d*_6_, δ [ppm]): 169.6 q (−NH-CO-CH_2_-CH_2_-O−), 168.9 q (pyrimidine C-2), 167.0 q (pyrimidine C-4,6), 166.8 q (−NH-CO-CH_2_-S−), 165.7 q (*N*-hydroxybenzamide-CH_2_-NH-CO−), 164.1 q (−CO-NH-OH), 158.2 q (phenyl C-3), 158.1 q (q, ^2^*J* = 35.7 Hz, F_3_C-COO−),156.9 q (thiazole C-2), 143.1 q (*N*-hydroxybenzamide C-4), 142.5 q (triazole C-4), 141.9 q (4-acylamidobenzamide C-4), 141.8 q (phenyl C-1), 134.7 (thiazole C-4), 131.2 q (*N*-hydroxybenzamide C-1), 131.0 q (thiazole C-5), 129.6 (phenyl C-5), 128.5 q (4-acylamidobenzamide C-1), 128.1 (4-acylamidobenzamide C-2,6), 127.0 (*N*-hydroxybenzamide C-3,5), 126.9 (*N*-hydroxybenzamide C-2,6), 124.8 (triazole C-5), 120.9 (phenyl C-6), 118.2 (4-acylamidobenzamide C-3,5), 116.1 (pyrimidine C-5), 114.9 (phenyl C-2), 112.4 (phenyl C-4), 69.73 (7 × −O-CH_2_-CH_2_-O−), 69.67 (−O-CH_2_-CH_2_-O−), 69.65 (−O-CH_2_-CH_2_-O−), 69.6 (−O-CH_2_-CH_2_-O−), 69.5 (−O-CH_2_-CH_2_-O−), 68.6 (−O-CH_2_-CH_2_-triazole−), 66.5 (−NH-CO-CH_2_-CH_2_-O−), 61.0 (−triazole-CH_2_-O−), 49.4 (−O-CH_2_-CH_2_-triazole−), 42.3 (*N*-hydroxybenzamide-CH_2_-NH-CO−), 37.2 (−NH-CO-CH_2_-CH_2_-O−), 34.0 (−NH-CO-CH_2_-S−), 31.9 (thiazole-CH_2_-phenyl), 23.2 (pyrimidine −CH_3_). ^19^F-NMR (565 MHz, DMSO-*d*_6_, δ [ppm]): −74.1. HRMS (ESI^+^): *m*/*z* calcd for C_59_H_79_N_10_O_16_S_2_^+^: 1247.5111 [M + H]^+^, found: 1247.5105; HPLC retention time 16.07 min, 95.0% (M3). The attached ^1^H and ^13^C NMR spectra contains residual solvent signals (DMSO). ^(*a*)^^1^H signals of −CONH-OH and F_3_C-COOH could not be detected. ^(^^*b*)13^C signal of F_3_C-COOH could not be detected.

#### 4-(1-(4-((3-((2-(2-((4,6-Dimethylpyrimidin-2-yl)thio)acetamido)thiazol-5-yl)methyl)phenoxy)methyl)-1*H*-1,2,3-triazol-1-yl)-3,6,9,12-tetraoxapentadecan-15-amido)-*N*-(7-(hydroxyamino)-7-oxoheptyl)benzamide (**46**)

After swelling of the resin **51** (0.10 mmol, 1.00 equiv) in DMF for 30 min, Fmoc deprotection was performed by treatment with 20% piperidine in DMF (1.5 mL, 2 × 5 min). Afterward, the resin was washed with DMF (5 × 2 mL), CH_2_Cl_2_ (5 × 2 mL), and DMF (5 × 2 mL). For the subsequent amide coupling reaction, a solution of N_3_-PEG_4_-COOH (87.0 mg, 0.30 mmol, 3.00 equiv), HATU (114 mg, 0.30 mmol, 3.00 equiv), and DIPEA (70.0 μL, 0.40 mmol, 4.00 equiv) in DMF (500 μL) was agitated for 5 min and then added to the resin. The reaction was shaken for 5 h at ambient temperature followed by washing with DMF (5 × 2 mL), CH_2_Cl_2_ (5 × 2 mL), and DMF (5 × 2 mL). The obtained azido-functionalized nonselective HDACi precursor resin **54** was directly used for the subsequent reaction. For the Huisgen cycloaddition a solution of SirReal-alkyne **35** (85.0 mg, 0.20 mmol, 2.00 equiv) in DMF (1.00 mL), TBTA (13.2 mg, 25.0 μmol, 0.25 equiv), 0.1 M aqueous CuSO_4_·5H_2_O (252 μL, 25.0 μmol, 0.25 equiv) in DMF (0.50 mL), 0.2 M aqueous ascorbic acid (252 μL, 50.0 μmol, 0.50 equiv) in DMF (0.50 mL), and and *tert*-BuOH (990 μL) were added in that sequence to the resin. The reaction was shaken for 18 h at ambient temperature. After washing with DMF (5 × 2 mL) and CH_2_Cl_2_ (5 × 2 mL), the resin was dried under reduced pressure followed by the cleavage of the crude product from the resin by treatment with cleavage solution (5% TFA in CH_2_Cl_2_, 1 mL/40 mg resin) for 1 h at ambient temperature. The filtrates were concentrated under reduced pressure, and the crude final product was purified by preparative HPLC (gradient elution from 5% to 95% acetonitrile (+0.1% TFA)/water (+0.1% TFA)). Lyophilization of the respective fractions yielded the title compound (47.0 mg, 43.1 μmol, 43%) as a TFA salt. ^1^H NMR^a^ (400 MHz, DMSO-*d*_6_, δ [ppm]): 12.20 (bs, 1H, −NH-CO-CH_2_-S−), 10.33 (bs, 1H, −CO-NH-OH), 10.12 (s, 1H, phenyl-NH-CO−), 8.29 (t, ^3^*J* = 6.1 Hz, 1H, −(CH_2_)_6_-NH-CO-phenyl−), 8.15 (s, 1H, triazole-H), 7.83–7.74 (m, 2H, 4-acylamidobenzamide H-2,6), 7.68–7.59 (m, 2H, 4-acylamidobenzamide H-3,5), 7.28–7.18 (m, 2H, thiazole H-4, phenyl H-5), 6.95–6.86 (m, 3H, pyrimidine H-5, phenyl H-2,4), 6.83 (dt, ^3^*J* = 7.5, ^4^*J* = 1.2 Hz, 1H, phenyl H-6), 5.09 (s, 2H, −triazole-CH_2_-O−), 4.51 (t, ^3^*J* = 5.1 Hz, 2H, −O-CH_2_-CH_2_-triazole−), 4.08 (s, 2H, −NH-CO-CH_2_-S−), 4.03 (s, 2H, −thiazole-CH_2_-phenyl−), 3.79 (t, ^3^*J* = 5.2 Hz, 2H, −O-CH_2_-CH_2_-triazole−), 3.67 (t, ^3^*J* = 6.2 Hz, 2H, −NH-CO-CH_2_-CH_2_-O−), 3.54–3.37 (m, 12H, 3 × −O-CH_2_-CH_2_-O−), 3.22 (q, ^3^*J* = 6.1 Hz, 2H, −(CH_2_)_5_-CH_2_-NH-CO−), 2.56 (t, ^3^*J* = 6.2 Hz, 2H, −NH-CO-CH_2_-CH_2_-O−), 2.28 (s, 6H, pyrimidine −CH_3_), 1.94 (t, ^3^*J* = 7.3 Hz, 2H, HO-NH-CO-CH_2_-(CH_2_)_5_−), 1.56–1.41 (m, 4H, HO-NH-CO-CH_2_-CH_2_-(CH_2_)_2_-CH_2_-CH_2_−), 1.35–1.18 (m, 4H, HO-NH-CO-(CH_2_)_2_-CH_2_-CH_2_-(CH_2_)_2_−). ^13^C NMR^b^ (101 MHz, DMSO-*d*_6_, δ [ppm]): 169.6 q (−NH-CO-CH_2_-CH_2_-O−), 169.1 q (−CO-NH-OH), 168.9 q (pyrimidine C-2), 167.0 q (pyrimidine C-4,6), 166.8 q (−NH-CO-CH_2_-S−), 165.5 q (−(CH_2_)_6_-NH-CO-phenyl−), 158.4 q (q, ^2^*J* = 38.4 Hz, F_3_C-COO−), 158.2 q (phenyl C-3), 156.9 q (thiazole C-2), 142.5 q (triazole C-4), 141.9 q (phenyl C-1), 141.6 q (4-acylamidobenzamide C-4), 134.7 (thiazole C-4), 131.1 q (thiazole C-5), 129.6 (phenyl C-5), 129.1 q (4-acylamidobenzamide C-1), 128.0 (4-acylamidobenzamide C-2,6), 124.9 (triazole C-5), 120.9 (phenyl C-6), 118.1 (4-acylamidobenzamide C-3,5), 116.1 (pyrimidine C-5), 114.9 (phenyl C-2), 112.4 (phenyl C-4), 69.7 (−O-CH_2_-CH_2_-O−), 69.71 (−O-CH_2_-CH_2_–O−), 69.66 (−O-CH_2_-CH_2_-O−), 69.64 (−O-CH_2_-CH_2_-O−), 69.57 (−O-CH_2_-CH_2_-O−), 69.5 (−O-CH_2_-CH_2_-O−), 68.6 (−O-CH_2_-CH_2_-triazole−), 66.5 (−NH-CO-CH_2_-CH_2_-O−), 61.0 (−triazole-CH_2_-O−), 49.4 (−O-CH_2_-CH_2_-triazole−), 37.2 (−NH-CO-CH_2_-CH_2_-O−), 34.1 (−NH-CO-CH_2_-S−), 32.2 (HO-NH-CO-CH_2_-(CH_2_)_5_−), 31.9 (thiazole-CH_2_-phenyl), 29.1 (HO-NH-CO-(CH_2_)_4_-CH_2_-CH_2_−), 28.4 (HO-NH-CO-(CH_2_)_2_-CH_2_-(CH_2_)_3_−), 26.3 (HO-NH-CO-(CH_2_)_3_-CH_2_-(CH_2_)_2_−), 25.1 (HO-NH-CO-CH_2_-CH_2_-(CH_2_)_4_−), 23.2 (pyrimidine −CH_3_). ^19^F-NMR (377 MHz, DMSO-*d*_6_, δ [ppm]): 75.1. HRMS (ESI^+^): *m*/*z* calcd for C_46_H_60_N_10_O_10_S_2_Na^+^: 999.3828 [M + H]^+^, found: 999.3835. HPLC retention time 16.10 min, 95.2% (M3). ^(*a*)1^H signals of −CONH-OH and F_3_C-COOH could not be detected. ^(*b*)13^C signal of F_3_C-COOH and HO-NH-CO-(CH_2_)_5_-CH_2_– (not reported due to overlap with DMSO signal) could not be detected.

#### Preloaded Resin **48**

After swelling of the modified resin **47** (1.00 g, estimated loading 1.50 mmol/g, 1.00 mmol, 1.00 equiv) for 30 min in DMF, the resin was washed with MeOH (3 × 5 mL). The phthaloyl protecting group was removed by treatment with 5% hydrazine monohydrate in MeOH for 15 min (2 × 5 mL/g resin). Afterward, the resin was washed with DMF (3 × 5 mL), MeOH (3 × 5 mL), CH_2_Cl_2_ (3 × 5 mL), and DMF (3 × 5 mL). For the subsequent amide coupling reaction, a solution of Fmoc-4-aminomethylbenzoic acid (1.12 g, 3.00 mmol, 2.00 equiv), HATU (1.14 g, 3.00 mmol, 2.00 equiv), HOBt·H_2_O (459 mg, 3.00 mmol, 2.00 equiv), and DIPEA (766 μL, 4.50 mmol, 3.00 equiv) in DMF (3 mL) was agitated for 5 min and then added to the resin. The amide coupling was performed for 20 h at ambient temperature. Afterward, the resin was washed with DMF (3 × 5 mL) and CH_2_Cl_2_ (3 × 5 mL). Completion of the reaction was monitored via TNBS test. The preloaded resin **48** was dried under reduced pressure and stored at 4 °C. Determined loading: 0.69 mmol/g.

#### Preferential HDAC6i Precursor Resin **49**

After swelling of the preloaded resin **48** (725 mg, 0.50 mmol, 1.00 equiv) in DMF for 30 min, Fmoc deprotection was performed by treatment with 20% piperidine in DMF. Afterward, the resin was washed with DMF (3 × 5 mL), CH_2_Cl_2_ (3 × 5 mL), and DMF (3 × 5 mL). For the subsequent amide coupling reaction, a solution of Fmoc-aminobenzoic acid (539 mg, 1.50 mmol, 3.00 equiv), HATU (570 mg, 1.50 mmol, 3.00 equiv), and DIPEA (350 μL, 2.00 mmol, 4.00 equiv) in DMF (1.5 mL) was agitated for 5 min and then added to the resin. After shaking the reaction mixture for 20 h, the resin was washed with DMF (3 × 5 mL) and CH_2_Cl_2_ (3 × 5 mL). Completion of the reaction was monitored via TNBS test. The resin was dried under reduced pressure and stored at 4 °C.

#### Preloaded Resin **50**

After swelling of the modified resin **47** (500 mg, estimated loading 1.50 mmol/g, 0.75 mmol, 1.00 equiv) for 30 min in DMF, the resin was washed with MeOH (3 × 5 mL). The phthaloyl protecting group was removed by treatment with 5% hydrazine monohydrate in MeOH for 15 min (2 × 3 mL). Afterward, the resin was washed with DMF (3 × 5 mL), MeOH (3 × 5 mL), CH_2_Cl_2_ (3 × 5 mL), and DMF (3 × 5 mL). For the subsequent amide coupling reaction a solution of Fmoc-7-aminoheptanoic acid (551 mg, 1.50 mmol, 2.00 equiv), HATU (570 mg, 1.50 mmol, 2.00 equiv), HOBt·H_2_O (230 mg, 1.50 mmol, 2.00 equiv), and DIPEA (383 μL, 2.25 mmol, 3.00 equiv) in DMF (2 mL) was agitated for 5 min and then added to the resin. The amide coupling was performed for 20 h at ambient temperature. Afterward, the resin was washed with DMF (3 × 5 mL) and CH_2_Cl_2_ (3 × 5 mL). Completion of the reaction was monitored via TNBS test. Upon completion of the reaction and washing, the resin was dried under reduced pressure and a loading between 0.77 mmol/g and 0.97 mmol/g was determined for different batches.

#### Nonselective HDACi Precursor Resin **51**

After swelling of the resin **50** (676 mg, 0.97 mmol/g, 0.65 mmol, 1,00 equiv) in DMF for 30 min, Fmoc-deprotection was performed by treatment with 20% piperidine in DMF. Afterward, the resin was washed with DMF (3 × 5 mL), CH_2_Cl_2_ (3 × 5 mL), and DMF (3 × 5 mL). For subsequent amide coupling reaction, a solution of Fmoc-4-aminobenzoic acid (701 mg, 1.95 mmol, 3.00 equiv), HATU (741 mg, 1.95 mmol, 3.00 equiv), and DIPEA (454 μL, 2.60 mmol, 4.00 equiv) in DMF (1.5 mL) was agitated for 5 min and then added to the resin. After 20 h reaction time at room temperature, the resin was washed with DMF (3 × 5 mL) and CH_2_Cl_2_ (3 × 5 mL). Completion of the reaction was monitored via TNBS test. The resin was dried under reduced pressure and stored at 4 °C.

#### 4-(Acetamidomethyl)-*N*-hydroxybenzamide (**55**)

Ethyl 4-(acetamidomethyl)benzoate (**58**, 163 mg, 0.737 mmol, 1 equiv) was dissolved in a mixture of 14.1 mL of dichloromethane and 14.1 mL of methanol and cooled in an ice bath to 0 °C. Then 1.354 mL (1.46 g, 50%: 730 mg, 22.1 mmol, 30 equiv) of an aqueous 50 wt % hydroxylamine solution were added, and the mixture was stirred at 0 °C for 10 min before 294.6 mg (7.37 mmol, 10 equiv) sodium hydroxide were added. After 30 min at 0 °C, the ice bath was removed and stirring of the colorless clear solution was continued at room temperature for 2 h. The reaction mixture was freed of all volatiles under reduced pressure, and the residue was taken up in 40 mL of deionized water. The suspension was cooled in an ice bath and brought to pH = 6 by the dropwise addition of 1 M HCl. The mixture was freed of all volatiles under reduced pressure. The residue was purified by flash column chromatography (MeOH (+1% AcOH)/dichloromethane (+1% AcOH): gradient 0–10%). For the final purification, normal phase preparative thin layer chromatography was used. As an eluent we used a mixture of dichloromethane, methanol, and AcOH (89/10/1). The title compound was obtained as a colorless solid (61 mg, 40%). ^1^H NMR (400 MHz, DMSO-*d*_6_, δ [ppm]): 11.21 (bs, 1H, −CO-NH-OH), 9.02 (bs, 1H, −CO-NH-OH), 8.44 (t, 1H, ^3^*J* = 5.7 Hz, −CH_2_-NH-CO-CH_3_), 7.80–7.64 (m, 2H, *N*-hydroxybenzamide H-2,6), 7.37–7.24 (m, 2H, *N*-hydroxybenzamide H-3,5), 4.28 (d, 2H, ^3^*J* = 5.7 Hz, −CH_2_-NH-CO-CH_3_), 1.88 (s, 3H, −CH_2_-NH-CO-CH_3_). ^13^C NMR (101 MHz, DMSO-*d*_6_, δ [ppm]): 169.3 q (−CH_2_-NH-CO-CH_3_), 164.1 q (−CO-NH-OH), 143.0 q (*N*-hydroxybenzamide C-4), 131.3 q (*N*-hydroxybenzamide C-1), 127.1 (*N*-hydroxybenzamide C-3,5), 126.9 (*N*-hydroxybenzamide C-2,6), 41.8 (−CH_2_-NH-CO-CH_3_), 22.6 (−CH_2_-NH-CO-CH_3_). HRMS (ESI^+^): *m*/*z* calcd for C_10_H_13_N_2_O_3_^+^: 209.0921 [M + H]^+^, found: 209.0920. LRMS *m*/*z* (ESI^+^): 439 [2M + Na]^+^. HPLC retention time 9.11 min, 99.2% (M2).

#### 4-(Benzamidomethyl)-*N*-hydroxybenzamide (**56**)

Ethyl 4-(benzamidomethyl)benzoate (**59**, 199 mg, 0.702 mmol, 1 equiv) was dissolved in a mixture of 13.4 mL of dichloromethane and 13.4 mL of methanol and cooled in an ice bath to 0 °C. Then 1.291 mL (1.39 g, 50%: 696 mg, 21.1 mmol, 30 equiv) of an aqueous 50 wt % hydroxylamine solution were added, and the mixture was stirred at 0 °C for 10 min before 281.0 mg (7.02 mmol, 10 equiv) sodium hydroxide were added. After 30 min at 0 °C, the ice bath was removed and stirring of the colorless clear solution was continued at room temperature for 2 h. The reaction mixture was freed of all volatiles under reduced pressure, and the residue was taken up in 40 mL of deionized water. The solution was cooled in an ice bath and brought to pH = 8 by the dropwise addition of 1 M HCl, when large amounts of white solid formed. The precipitate was collected by filtration, washed with water (2 × 1 mL) and ice-cold diethyl ether (2 × 2 mL), and dried under reduced pressure. For the final purification preparative HPLC (gradient elution from 10% to 35% acetonitrile/water (+0.1% TFA)) was used to yield the title compound as a pale-red solid (67 mg, 35%). ^1^H NMR (600 MHz, DMSO-*d*_6_, δ [ppm]): 11.16 (bs, 1H, −CO-NH-OH), 9.84–8.21 (m, 2H, −CH_2_-NH-CO-C_6_H_5_, −CO-NH-OH), 7.94–7.87 (m, 2H, benzamide H-2,6), 7.75–7.68 (m, 2H, *N*-hydroxybenzamide H-2,6), 7.54 (t, 1H, ^3^*J* = 7.4 Hz, benzamide H-4), 7.48 (t, 2H, ^3^*J* = 7.4 Hz, benzamide H-3,5), 7.42–7.35 (m, 2H, *N*-hydroxybenzamide H-3,5), 4.51 (d, 2H, ^3^*J* = 5.9 Hz, −CH_2_-NH-CO-C_6_H_5_). ^13^C NMR (151 MHz, DMSO-*d*_6_, δ [ppm]): 169.3 q (−CH_2_-NH-CO-C_6_H_5_), 164.1 q (−CO-NH-OH), 142.9 q (*N*-hydroxybenzamide C-4), 134.2 q (benzamide C-1), 131.29 (benzamide C-4), 131.26 q (*N*-hydroxybenzamide C-1), 128.3 (benzamide C-3,5), 127.2 (benzamide C-2,6), 127.0 (*N*-hydroxybenzamide C-3,5), 126.9 (*N*-hydroxybenzamide C-2,6), and 42.4 (−CH_2_-NH-CO-C_6_H_5_). HRMS (ESI^+^): *m*/*z* calcd for C_15_H_15_N_2_O_3_^+^: 271.1077 [M + H]^+^, found: 271.1080. LRMS *m*/*z* (ESI^+^): 541 [2M+H]^+^. HPLC retention time 12.10 min, >99.5% (M1).

#### 4-((*N*-Butylpentanamido)methyl)-*N*-hydroxybenzamide (**57**)

Ethyl 4-((*N*-butylpentanamido)methyl)benzoate (**60**, 120 mg, 0.376 mmol, 1 equiv) was dissolved in a mixture of 5 mL dichloromethane and 5 mL methanol and cooled in an ice bath to 0 °C. 698 μL (747 mg, 50%: 373.7 mg, 11.27 mmol, 30 equiv) of an aqueous 50 wt % hydroxylamine solution were added, and the mixture was stirred at 0 °C for 10 min before 149.5 mg (3.76 mmol, 10 equiv) of sodium hydroxide were added. After 30 min at 0 °C, the ice bath was removed and stirring of the colorless clear solution was continued at room temperature for 3 h. The reaction mixture was freed of all volatiles under reduced pressure and the residue was taken up in 8 mL of deionized water. The suspension was cooled in an ice bath and brought to pH = 8 by the dropwise addition of 1 M HCl, when large amounts of white solid formed. The solid was collected by centrifugation, washed with ice-cold water (5 mL), and dried under reduced pressure. The residue was purified by reversed phase flash column chromatography (acetonitrile/water (1% TFA (v/v)): gradient 3–40%) to yield the title compound as a colorless solid (64 mg, 56%). ^1^H NMR^a^ (400 MHz, DMSO-*d*_6_, δ [ppm]): 11.20, 11.17 (2 × bs, 1H, −CO-NH-OH), 8.97 (bs, 1H, −CO-NH-OH), 7.77–7.66 (m, 2H, *N*-hydroxybenzamide H-2,6), 7.29–7.21 (m, 2H, *N*-hydroxybenzamide H-3,5), 4.60, 4.53 (2*s*, 2H, −CH_2_-N(C_4_H_9_)-CO-C_4_H_9_), 3.24, 3.21 (2*t*, 2H, ^3^*J* = 7.7 Hz, −CH_2_-N(CH_2_-C_3_H_7_)-CO-C_4_H_9_), 2.38, 2.23 (2*t*, 2H, ^3^*J* = 7.4 Hz, −CH_2_-N(C_4_H_9_)-CO-CH_2_-C_3_H_7_), 1.60–1.15 (m, 8H, −CH_2_-N(CH_2_-(CH_2_)_2_-CH_3_)-CO-CH_2_-(CH_2_)_2_-CH_3_), 0.93–0.77 (m, 6H, −CH_2_-N((CH_2_)_3_-CH_3_)-CO-(CH_2_)_3_-CH_3_). ^13^C NMR^a^ (101 MHz, DMSO-*d*_6_, δ [ppm]): 172.2, 172.1 q (−CH_2_-N(C_4_H_9_)-CO-C_4_H_9_), 164.1, 163.9 q (−CO-NH-OH), 141.9, 141.5 q (*N*-hydroxybenzamide C-4), 131.6, 131.4 q (*N*-hydroxybenzamide C-1), 127.3, 126.3 (*N*-hydroxybenzamide C-3,5), 127.2, 127.0 (*N*-hydroxybenzamide C-2,6), 50.1, 47.5 (−CH_2_-N(C_4_H_9_)-CO-C_4_H_9_), 46.9, 45.3 (−CH_2_-N(CH_2_-C_3_H_7_)-CO-C_4_H_9_), 32.1, 31.7 (−CH_2_-N(C_4_H_9_)-CO-CH_2_-C_3_H_7_), 30.4, 29.3 (−CH_2_-N(CH_2_-CH_2_-C_2_H_5_)-CO-C_4_H_9_), 27.2, 27.1 (−CH_2_-N(C_4_H_9_)-CO-CH_2_-CH_2_-C_2_H_5_), 22.0, 21.8 (−CH_2_-N(C_4_H_9_)-CO-(CH_2_)_2_-CH_2_-CH_3_), 19.6, 19.5 (−CH_2_-N((CH_2_)_2_-CH_2_-CH_3_)-CO-C_4_H_9_), 13.9, 13.8 (−CH_2_-N(C_4_H_9_)-CO-(CH_2_)_3_-CH_3_), 13.8, and 13.7 (−CH_2_-N((CH_2_)_3_-CH_3_)-CO-C_4_H_9_). HRMS (ESI^+^): *m*/*z* calcd for C_17_H_27_N_2_O_3_^+^: 307.2016 [M + H]^+^, found: 307.2019; LRMS *m*/*z* (ESI^+^): 307 [M + H]^+^. HPLC retention time 15.56 min, 99.3% (M1). ^(*a*)^Rotamers were observed.

#### Ethyl 4-(Acetamidomethyl)benzoate (**58**)^65^

Ethyl 4-(aminomethyl)benzoate (**37**, 206 mg, 1.212 mmol, 1 equiv) and DIPEA (235 mg, 316.5 μL, 1.818 mmol, 1.5 equiv) were dissolved in 7 mL of dichloromethane and cooled in an ice bath to 0 °C. Acetyl chloride (143 mg, 130 μL, 1.818 mmol, 1.5 equiv) was added, and the mixture was stirred at 0 °C for 10 min. Then, the ice bath was removed and stirring was continued at room temperature for 1 h. After complete conversion, 50 mL of dichloromethane were added and the reaction mixture was washed aqueous HCl (0.5 M, 2 × 50 mL), saturated NaHCO_3_ solution (50 mL), and brine (50 mL). The organic layer was dried over Na_2_SO_4_, filtered, and dried under reduced pressure. The residue was purified by flash column chromatography (EtOAc/isohexane: gradient 12–100%) to yield the title compound as a colorless solid (192 mg, 72%). ^1^H NMR (400 MHz, DMSO-*d*_6_, δ [ppm]): 8.45 (t, 1H, ^3^*J* = 5.7 Hz, −CH_2_-NH-CO-CH_3_), 8.00–7.80 (m, 2H, ethyl benzoate H-2,6), 7.47–7.28 (m, 2H, ethyl benzoate H-3,5), 4.39–4.24 (m, 4H, −CH_2_-NH-CO-CH_3_, H_3_C-CH_2_-O-O−), 1.90 (s, 3H, −CH_2_-NH-CO-CH_3_), 1.32 (t, 3H, ^3^*J* = 7.1 Hz, H_3_C-CH_2_-O-CO−). ^13^C NMR (101 MHz, DMSO-*d*_6_, δ [ppm]): 169.3 q (−CH_2_-NH-CO-CH_3_), 165.6 q (H_3_C–CH_2_-O-CO−), 145.3 q (ethyl benzoate, C-4), 129.2 (ethyl benzoate C-2,6), 128.4 q (ethyl benzoate C-1), 127.3 (ethyl benzoate C-3,5), 60.7 (H_3_C-CH_2_-O-CO−), 41.9 (−CH_2_-NH-CO–CH_3_), 22.6 (−CH_2_-NH-CO-CH_3_), 14.2 (H_3_C-CH_2_-O-CO−). HRMS (ESI^+^): *m*/*z* calcd for C_12_H_16_NO_3_^+^: 222.1125 [M + H]^+^, found: 222.1126. LRMS *m*/*z* (ESI^+^): 443 [2M + H]^+^. The spectroscopic data are in good agreement with the literature values.^65^

#### Ethyl 4-(Benzamidomethyl)benzoate (**59**)

Ethyl 4-(aminomethyl)benzoate (**37**, 206 mg, 1.212 mmol, 1 equiv) and DIPEA (235 mg, 316.5 μL, 1.818 mmol, 1.5 equiv) were dissolved in 7 mL of dichloromethane and cooled in an ice bath to 0 °C. Benzoyl chloride (256 mg, 211 μL, 1.818 mmol, 1.5 equiv) was added, and the mixture was stirred at 0 °C for 10 min. Then, the ice bath was removed and stirring was continued at room temperature for 1 h. After complete conversion, 50 mL of dichloromethane were added and the reaction mixture was washed aqueous HCl (0.5 M, 2 × 50 mL), saturated NaHCO_3_ solution (50 mL), and brine (50 mL). The organic layer was dried over Na_2_SO_4_, filtered, and dried under reduced pressure. The residue was purified by flash column chromatography (EtOAc/isohexane: gradient 3–45%) to yield the title compound as a colorless solid (296 mg, 86%). ^1^H NMR (400 MHz, DMSO-*d*_6_, δ [ppm]): 9.14 (t, 1H, ^3^*J* = 6.0 Hz, −CH_2_-NH-CO-C_6_H_5_), 7.96–7.87 (m, 4H, ethyl benzoate H-2,6 and benzamide H-2,6), 7.59–7.52 (m, 1H, benzamide H-4), 7.51–7.42 (m, 4H, ethyl benzoate H-3,5 and benzamide H-3,5), 4.55 (d, 2H, ^3^*J* = 6.0 Hz, −CH_2_-NH-CO-C_6_H_5_), 4.30 (q, 2H, ^3^*J* = 7.1 Hz, H_3_C-CH_2_-O-CO−), 1.31 (t, 3H, ^3^*J* = 7.1 Hz, H_3_C-CH_2_-O-CO−). ^13^C NMR (101 MHz, DMSO-*d*_6_, δ [ppm]): 166.3 q (−CH_2_-NH-CO-C_6_H_5_), 165.6 q (H_3_C-CH_2_-O-CO−), 145.3 q (ethyl benzoate, C-4), 134.1 q (benzamide C-1), 131.4 (benzamide C-4), 129.2 (ethyl benzoate C-2,6), 128.4 (ethyl benzoate C-1 and benzamide C-3,5), 127.33 (ethyl benzoate C-3,5), 127.27 (benzamide C-2,6), 60.7 (H_3_C-CH_2_-O-CO−), 42.5 (−CH_2_-NH-CO-C_6_H_5_), and 14.2 (H_3_C-CH_2_-O-CO−). HRMS (ESI^+^): *m*/*z* calcd for C_17_H_18_NO_3_^+^: 284.1281 [M + H]^+^, found: 284.1279. LRMS *m*/*z* (ESI^+^): 567 [2M + H]^+^.

#### Ethyl 4-((*N*-Butylpentanamido)methyl)benzoate (**60**)

Ethyl 4-((butylamino)methyl)benzoate (**40**, 150 mg, 0.637 mmol) was dissolved in 3 mL of dichloromethane. The solution was cooled to 0 °C. Pentanoyl chloride (113.5 μL, 0.956 mmol) was added. Then, pyridine (102.7 μL, 1.28 mmol) was added in a dropwise manner. The reaction mixture was allowed to warm up to ambient temperature and was stirred for further 4 h. After completion of the reaction, the reaction mixture was partitioned between dichloromethane (20 mL) and a saturated aqueous solution of sodium bicarbonate (20 mL). The organic layer was separated, and the aqueous layer was extracted with dichloromethane (2 × 20 mL). The combined organic layer was dried over Na_2_SO_4_, filtered, and concentrated under reduced pressure. The residue was purified by flash column chromatography (EtOAc/isohexane: gradient 3–27%) to yield the title compound as a colorless liquid (187 mg, 92%). ^1^H NMR^a^ (600 MHz, DMSO-*d*_6_, δ [ppm]): 7.98–7.88 (m, 2H, ethyl benzoate H-2,6), 7.35–7.29 (m, 2H, ethyl benzoate H-3,5), 4.65, 4.57 (2*s*, 2H, −CH_2_-N(C_4_H_9_)-CO-C_4_H_9_), 4.31, 4.30 (2*q*, 2H, ^3^*J* = 7.0 Hz, H_3_C-CH_2_-O-CO−), 3.26, 3.22 (2*t*, 2H, ^3^*J* = 7.0 Hz, −CH_2_-N(CH_2_-C_3_H_7_)-CO-C_4_H_9_), 2.38, 2.22 (2*t*, 2H, ^3^*J* = 7.4 Hz, −CH_2_-N(C_4_H_9_)-CO-CH_2_-C_3_H_7_), 1.60–1.12 (m, 11H, H_3_C-CH_2_-O-CO–, −CH_2_-N(CH_2_-(CH_2_)_2_-CH_3_)-CO-CH_2_-(CH_2_)_2_-CH_3_), 0.93–0.74 (m, 6H, −CH_2_-N((CH_2_)_3_-CH_3_)-CO-(CH_2_)_3_-CH_3_). ^13^C NMR^a^ (151 MHz, DMSO-*d*_6_, δ [ppm]): 172.2, 172.1 q (−CH_2_-N(C_4_H_9_)-CO-C_4_H_9_), 165.54, 165.47 q (H_3_C-CH_2_-O-CO−), 144.3, 143.9 q (ethyl benzoate, C-4), 129.5, 129.2 (ethyl benzoate C-2,6), 128.7, 128.5 q (ethyl benzoate C-1), 127.5, 126.6 (ethyl benzoate C-3,5), 60.7, 60.6 (H_3_C-CH_2_-O-CO−), 50.1, 47.6 (−CH_2_-N(C_4_H_9_)-CO-C_4_H_9_), 47.0, 45.4 (−CH_2_-N(CH_2_-C_3_H_7_)-CO-C_4_H_9_), 32.0, 31.6 (−CH_2_-N(C_4_H_9_)-CO-CH_2_-C_3_H_7_), 30.4, 29.3 (−CH_2_-N(CH_2_-CH_2_-C_2_H_5_)-CO-C_4_H_9_), 27.1, 27.0 (−CH_2_-N(C_4_H_9_)-CO-CH_2_–CH_2_-C_2_H_5_), 21.9, 21.8 (−CH_2_-N(C_4_H_9_)-CO-(CH_2_)_2_-CH_2_-CH_3_), 19.6, 19.4 (−CH_2_-N((CH_2_)_2_-CH_2_-CH_3_)-CO-C_4_H_9_), 14.2 (H_3_C-CH_2_-O-CO−), 13.8, 13.7 (−CH_2_-N(C_4_H_9_)-CO-(CH_2_)_3_-CH_3_).13.7, 13.6 (−CH_2_-N((CH_2_)_3_-CH_3_)-CO-C_4_H_9_). HRMS (ESI^+^): *m*/*z* calcd for C_19_H_30_NO_3_^+^: 320.2220 [M + H]^+^, found: 320.2220. LRMS *m*/*z* (ESI^+^): 320 [M + H]^+^. ^(*a*)^Rotamers were observed.

### Protein Expression and Purification

Recombinant human Sirt1_134–747_, Sirt2_56–356_, and Sirt3_118–395_ were expressed as described previously.^66^ Chemically competent *Escherichia coli* BL21 Star (DE3) cells were transformed with the expression vectors pET30S-hSirt1_134–747_, pET30S-hSirt2_56–356_, or pET15dt-Sirt3_118–395_. Cells were grown to an OD_600_ of 0.6 at 37 °C in 2XYT medium (supplemented with 50 μg mL^–1^ kanamycin). Overexpression of sirtuins was induced by IPTG (final concentration 1 mM) and after further cultivation at 20 °C for 12 h cells were harvested by centrifugation (15 min, 5000*g*). Cells were resuspended in lysis buffer (100 mM Tris/HCl buffer at pH 8.0, 150 mM NaCl and 10% (v/v) glycerol) and cell lysis was achieved by ultrasonication (Branson Digital Sonifier 250) at 70% amplitude for 10 min (3 s working, 10 s pause). After centrifugation with 100 000*g* for 1 h the supernatant was loaded on a Strep-Tactin Superflow cartridge (5 mL bed volume, IBA Lifescience, Germany). After elution with lysis buffer containing d-desthiobiotin (5 mM, IBA Lifescience, Germany), the proteins were further purified by size-exclusion chromatography (Superdex S200 26/60, GE Healthcare, IL, USA) equilibrated with Tris/HCl buffer (20 mM, 150 mM NaCl, pH 8.0) and concentrated. Purity and identity of the target proteins were verified by SDS–PAGE and protein concentration was determined by BCA-assay using BSA as a standard.

### *In Vitro* Deacetylation Activity Assays for Sirt1–3 as Well as HDAC6 and HDAC1–3

Inhibition of Sirt1, Sirt2, and Sirt3 activities was determined using a trypsin-based fluorescence assay, previously described by Heltweg et al.^45^ In black 96-well plates (OptiPlateTM-96F, black, 96-well, Pinch bar design, PerkinElmer, USA), the respective sirtuin (Sirt1_134–747_, Sirt2_56–356_, or Sirt3_118–395_) was mixed with 5 μL of ZMAL (12.6 mM stock solution in DMSO, 10.5 μM final assay concentration), 3 μL of inhibitor in DMSO in varying concentrations or DMSO as a control (final DMSO concentration 5% (v/v)) and filled up to 55 μL with assay buffer (50 mM Tris/HCl, 137 mM NaCl, 2.7 mM KCl, 1 mM MgCl_2_, pH 8.0). Substrate conversion was adjusted to 15–30% for the DMSO control and a blank control with no enzyme and a 100% conversion control without enzyme but with AMC (12.6 mM stock solution in DMSO, 10.5 μM final assay concentration) instead of ZMAL were performed as well. The enzymatic reaction was started by addition of 5 μL NAD^+^ (6 mM, final assay concentration 500 μM), and plates were incubated at 37 °C and 140 rpm for 4 h. Addition of 60 μL of a trypsin containing stop solution (50 mM Tris, 100 mM NaCl, 6.7% (v/v) DMSO, 5.5 U μL^–1^ trypsin, 8 μM nicotinamide, pH 8.0) stopped the catalytic reaction. Further incubation at 37 °C and 140 rpm for 20 min led to release of the fluorophore AMC. Fluorescence intensity was measured using a microplate reader (λ_Ex_ = 390 nm, λ_Em_ = 460 nm, BMG POLARstar Optima, BMG Labtech, Germany). All experiments were performed in duplicate series in at least two independent experiments. Inhibition was calculated in % in relation to the DMSO control after blank subtraction. IC_50_ values were determined using OriginPro 9G (OriginLab, USA) or GraphPad 7.0 by a nonlinear regression to fit the dose response curve. All experiments were performed at least in duplicates.

*In vitro* deacetylation assays for HDAC6 and HDAC1–3 were performed as previously reported.^44^ All reactions were performed in OptiPlate-96 black microplates (PerkinElmer) with duplicate series in at least two independent experiments. An assay end volume of 50 μL was set for each experiment, and all reactions were carried out in assay buffer (50 mM Tris-HCl, pH 8.0, 137 mM NaCl, 2.7 mM KCl, 1.0 mM MgCl_2_, 0.1 mg/mL BSA) with appropriate concentrations of substrate and inhibitors obtained by dilution from 10 mM stock solutions. Control wells without enzyme and control wells with vorinostat as reference were included in each plate. After addition of 5 μL of test compound or control in assay buffer, 35 μL of the fluorogenic substrate ZMAL (21.43 μM in assay buffer) and 10 μL of the enzyme solution, the plates were incubated at 37 °C for 90 min. Human recombinant HDAC1 (BPS Bioscience, catalogue no. 50051), HDAC2 (BPS Bioscience, catalogue no. 50052), HDAC3/NcoR2 (BPS Bioscience, catalogue no. 50003), or HDAC6 (BPS Bioscience, catalogue no. 50006) was applied. Afterward, 50 μL of 0.4 mg/mL trypsin in trypsin buffer (50 mM Tris-HCl, pH 8.0, 100 mM NaCl) was added, followed by further incubation at 37 °C for 30 min. Plates were analyzed using a Fluoroskan Ascent microplate reader (Thermo Scientific), and fluorescence was measured with an excitation wavelength of 355 nm and an emission wavelength of 460 nm.

### *In Vitro* Demyristoylation Activity Assays for Sirt2

The discontinuous Sirt2 demyristoylation assay that was used to determine the IC_50_ values given in Table 2 was performed as previously described.^47^ In brief, the assay was performed in a similar manner as described for sirtuin deacetylation activity. However, instead of ZMAL, a myristoylated substrate (ZMML) was used, and the Tris buffer was exchanged for a HEPES buffer (25 mM 4-(2-hydroxyethyl)-1-piperazineethanesulfonic acid (HEPES), 137 mM NaCl, 2.7 mM KCl, 1 mM MgCl_2_, 0.015% Triton X-100, pH 8.0). AMC was used again as 100% conversion control. All experiments were performed in duplicate series in at least two independent experiments. OriginPro 9.0 and GraphPad 7.0 were used for the analysis of results.

As a second method to confirm the inhibition of Sirt2-mediated demyristoylation by **33**, we used a continuous Sirt2 demyristoylation assay as reported by Zessin et al.^48^ In brief, this assay was performed in a black 384-well fluorescence plate in a total volume of 40 μL of Sirt assay buffer (20 mM Tris-HCl, 150 mM NaCl, 5 mg/mL MgCl_2_, 2 mg/mL BSA, pH 7.8). Dilution series of the inhibitor was done in DMSO. A total of 2 μL of the inhibitor dilutions were incubated with 14 μL of the peptide substrate, referred to as F4 in Zessin et al.^48^ (final concentration 40 nM) and 14 μL of Sirt2 solution (final concentration 1 nM) for 5 min at room temperature. In addition to the inhibitor-containing samples, a sample without inhibitors was measured as a positive control, and a sample without enzymes was measured as a negative control. The DMSO concentration was set to 5% (v/v). The reaction was started by the addition of 10 μL of NAD^+^ (final concentration 500 μM). The product formation was monitored by the change in fluorescence intensity, and the reaction rate represented the activity. Fluorescence readout was performed with a BMG Fluostar Omega plate reader at following wavelengths: Excitation wavelength: λ_Ex_ = 485 ± 10 nm, emission wavelength: λ_Em_ = 530 ± 10 nm. Inhibition was calculated using the following equation:
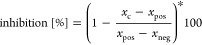
With *x*_c_, slope of compound; *x*_pos_, mean slope of positive control; *x*_neg_, mean slope of negative control. Data fitting was carried out by GraphPad 9.0.2 using nonlinear fit ([inhibitor] vs response–variable slope (four parameters)). Experiments were performed in triplicate.

### *In Vitro* Deacylation Activity Assays for Sirt5–6

These discontinuous HPLC assays for Sirt5 and Sirt6, respectively, were performed as previously described.^67^ In brief, the reactions of Sirt5 and Sirt6 were performed in a total volume of 120 μL in Sirt buffer, containing 20 mm TRIS (pH 7.8), 150 mM NaCl, 5 mM MgCl_2_, with 0.2 mg/mL BSA. The inhibitors were dissolved in DMSO, and the final DMSO concentration was 5% (v/v). The inhibitors (20–500 μM) were preincubated together with the master mix for 5 min at 37 °C. The master mix consisted of NAD^+^ (1 mM) and the respective substrate (Sirt5: Bz-GVLK(Succ)EYGV-NH_2_ final conc = 5 μM and Sirt6: Ac-EALPKK(Myr)Y(NO_2_)GG-NH_2_ final conc = 20 μM). The reactions were started by the addition of enzyme (Sirt5 = 100 nM, Sirt6 = 500 nM). After shaking at 37 °C for 5 min for Sirt5 or after 1 h for Sirt6, 100 μL of the reaction was added to 20 μL of stop solution consisting of 1% (v/v) trifluoroacetic acid (TFA) and 10% (v/v) ACN in dd. H_2_O. The reaction solution in a volume of 100 μL was injected into the RP-HPLC system, and compounds were separated using linear gradients.

The solvents used were water (solvent A) and ACN (solvent B), both containing 0.1% TFA. The separations were performed on a 3.0 mm × 50 mm reversed phase column (Phenomenex Kinetex XB C-18, 2.6 μm) with a flow rate of 0.6 mL/min. The mixtures of the Sirt5 reactions were separated using a linear gradient from 10% to 60% solvent B within 6 min. The absorbance at 220 nm was used to quantify product and substrate peak areas. The mixtures of the Sirt6 reactions were separated using a linear gradient from 5% to 95% solvent B within 6 min. The absorbance of the *p*-nitrotyrosine (λ = 360 nm) was used to quantify product and substrate peak areas. The reactions were performed once in duplicates. The conversion in the noninhibited reaction was assigned to 100%, and the conversion in a reaction without enzyme was defined as 0%. The normalized conversions were plotted as a function of the logarithm inhibitor concentration and the IC_50_ values were calculated using nonlinear regression for normalized dose–response curves with GraphPad Prism8.

### *In Vitro* Deacylation Activity Assay for HDAC4–5, HDAC7, and HDAC9–10

These continuous fluorescence-based assays for monitoring the enzymatic activity of HDAC4, 5, 7, and 9 were performed as previously described.^68^ In brief, all reactions were performed in 384-well plates (GreinerBioONe, catalogue no. 784900) with a total volume of 40 μL per well in HDAC buffer (20 mM HEPES buffer (pH 7.4), 140 mM NaCl, 10 mM KCl, 1 mM TCEP, and 0.2 mg/mL BSA). Inhibitors were dissolved in DMSO and the final DMSO concentration was 3% (v/v). DMSO only was used for both negative and positive control. The inhibitors (6–20000 nM) were preincubated with the enzyme (HDAC4 = 5 nM, HDAC5 = 10 nM, HDAC7 = 5 nM, HDAC 9 = 20 nM) for 5 min at room temperature while shaking. The reaction was started by adding the substrate Abz-SRGGK(STFA)FRR-NH_2_ (STFA = thiotrifluoroacetyl residue = fluorescence quencher). The change in florescence was monitored over 30 min every 30 s. Then 20 mM HEPES buffer (pH 7.4) containing 0.5 mg/mL BSA was used for the HDAC10 measurements. The inhibitors and the enzyme (5 nM) were prepared as described above. The reaction was also started by the addition of the substrate (an intramolecular quenched spermidine derivative, final conc = 50 μM). The change in florescence was monitored over 60 min every 50 s. All measurements were performed on an EnVision 2104 Multilabel reader (PerkinElmer, Waltham, MA) with a 330 ± 75 nm excitation filter and a 430 ± 8 nm emission filter. All measurements were performed in triplicates with *n* = 2. The fluorescence intensity was plotted as a function of time. The initial slope of these plots was set as activity. The activity in the noninhibited reaction was assigned to 100%, and the activity in a reaction without enzyme was defined as 0%. The normalized activity was plotted as a function of the logarithm inhibitor concentration and the IC_50_ values were calculated using nonlinear regression for normalized dose–response curves with GraphPad Prism8.

### *In Vitro* Deacylation Activity Assay for HDAC8

This assay was performed as previously described.^69^ In brief, inhibition of HDAC8 was measured in 1/2 AREAPLATE- 96 F microplates (PerkinElmer) with a total assay volume of 30 μL. HDAC8 enzyme was obtained as described before.^70,71^ The conversion rate of the enzyme was adjusted to 10–20%. 22.5 μL of enzyme in HDAC buffer (50 mM KH_2_PO_4_, 15 mM Tris, pH 7.5, 3 mM MgSO_4_·7 H_2_O, 10 mM MgSO_4_) was mixed with 2.5 μL of inhibitor in DMSO and 5 μL of *Z*-l-Lys(ε-trifluoroacetyl)-AMC (also referred to as ZMTFAL, 150 μM). The plate was incubated at 37 °C for 90 min. The reaction was stopped by adding 30 μL of freshly prepared stop solution to each well, composed of trichostatin A (2.75 μM), trypsin from bovine pancreas [10 000 BAEE units mg^–1^ at 1 mg mL^–1^, trypsin buffer pH 8.0 (50 mM Tris·HCl, 100 mM NaCl)]. After incubation for 20 min at 37 °C, fluorescence intensity was measured at an excitation wavelength of 390 nm and an emission wavelength of 460 nm in a microtiter plate reader (BMG Fluostar). IC_50_ values were determined by using 10 different compound concentrations. All experiments were performed in triplicate. Calculations were performed using standard methods and the GraphPad Prism software package (version 9.0.2).

### Cell-Based Immunofluorescence Microscopy Studies on α-Tubulin Hyperacetylation

The detection of acetylation levels of α-tubulin by immunofluorescence was performed as previously described.^47^ In brief, PC-3M-luc cells (25 000 cells per well) were plated in Ibidi 8-well slides (Ibidi, catalogue no. 80826) and incubated overnight at 37 °C, 5% CO_2_. Next, cells were treated with 20 μM of inhibitor. After 5 h, the medium was removed and the cells washed with PBS and fixed with 4% PFA for 8–10 min at ambient temperature. Cells were rinsed three times with PBS and lysed with extraction buffer (PBS, 0.1% Triton X-100) for 3–5 min at ambient temperature. After another washing step with PBS, blocking buffer (PBS, 0.1% Triton, 5% FCS) was added for at least 10 min before incubating with monoclonal acetylated α-tubulin antibody (1:500, Sigma-Aldrich, catalogue no. T6793) in blocking buffer overnight at 4 °C. The cells were rinsed three times with blocking buffer and incubated with goat antimouse IgG H&L Alexa Fluor 647 (Abcam, no. ab150115), diluted 1:2000 in blocking buffer, for 30 min in the dark. The cells were rinsed two times with blocking buffer and once with PBS and DAPI in mounting medium (VECTASHIELD HardSet Antifade Mounting Medium with DAPI, no. H-1500-10) diluted 1:50 in PBS was added and it was incubated for 10 min in the dark. Confocal microscopy was performed with a Leica SP8 confocal microscope equipped with a 40×/1.40 oil objective (Leica Microsystems) keeping the laser settings of the images constant to allow direct comparison of signal intensities between images.

### Cell-Based NanoBRET Assay to Determine Sirt2 Target Engagement

NanoBRET experiments were performed as previously described.^47^ In brief, HEK293T cells were plated in 6-well plates (Sarstedt, catalogue no. 83.1839.300) at a density of 8 × 10^5^ cells per well and incubated 2–4 h at 37 °C and 5% CO_2_ before transfection. The fusion protein plasmids were transfected using Fugene HD transfection reagent (Promega) according to the manufacturer’s protocol. First, 2 μg of fusion protein DNA were dissolved in 100 μL of medium without serum and phenol red to obtain a concentration of 0.02 μg of DNA per μL. Next, Fugene reagent was added to form DNA:Fugene complexes in a ratio of 1:3 and the mix was shortly vortexed and incubated for 15 min at ambient temperature. The mix was added dropwise to the HEK293T cells followed by incubation for 20–24 h at 37 °C and 5% CO_2_. Cells were trypsinized, resuspended in medium without serum and phenol red, and adjusted to a concentration of 2 × 10^5^ cells per mL. All compounds were prepared as concentrated stock solutions dissolved in DMSO. To determine affinities of the inhibitors, a final tracer concentration of 2 μM was used. As a fluorescent Sirt2 tracer, we used SirReal-TAMRA with a reported *K*_d_ value of 0.25 μM.^47^ Serially diluted inhibitors and tracer were added to the cell suspension, and 100 μL of the mixture were seeded in 96-well white, sterile nonbinding surface plates (Greiner Bio-One, catalogue no. 655083). Plates were incubated at 37 °C and 5% CO_2_ for 2 h. For BRET measurements, 25 μL of 1:100 diluted NanoBRET NanoGlo Substrate (Promega catalogue no. N1571) was added to the wells according to the manufacturer’s protocol and incubated for 2–3 min at ambient temperature. For all measurements, the 2102 EnVisionTM Multilabel reader (PerkinElmer) was used, equipped with 460 nm filter (donor) and 615 nm (acceptor) filter. The BRET ratios were calculated as difference between the acceptor/donor channel emission ratio of the sample and the acceptor/donor emission ratio of the control:
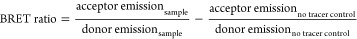


The obtained NanoBRET ratios of the samples were normalized to the DMSO control. IC_50_ values were calculated with a four-parameter logistic fit. Data analysis was performed using GraphPad Prism 7.0.

### Cell Viability Assays

Cell viability assays of HGC27 and PC-3M-luc cells were performed by using the Celltiter 96 Aqueous Nonradioactive Proliferation Assay (Promega). Cells were seeded in sterile 96-well plates at a density of 2000 cells per well and incubated overnight at 37 °C and 5% CO_2_. Compound or vehicle were added to a final concentration of 0.5% DMSO. After 72 h of incubation time, 20 μL of a mixture (20:1) consisting of MTS (3-(4,5-dimethylthiazol-2-yl)-5-(3-carboxymethoxyphenyl)-2-(4-sulfophenyl-2*H*-tetrazolium) and PMS (phenazine methosulfate) were added to each well. Absorbance was measured after another 2–4 h with a BMG LABTECH POLARstar OPTIMA plate reader (BMG Labtechnologies, Germany). Experiments were performed in triplicates, and EC_50_ values were calculated using the Prism (GraphPad Software, San Diego, CA, USA). EC_50_ was defined as the concentration that led to 50% viable cells. Cell viability assays with W1 and MCF-7 cells were performed by applying an MTT assay using 3-(4,5-dimethylthiazol-2-yl)-2,5-diphenyltetrazolium bromide (BioChemica, Applichem GmbH, Darmstadt, Germany). Cells were seeded at densities of 10^4^ cells/well for W1 cells or 2 × 10^3^ cells/well for MCF-7 cells. Tests were performed in 96-well plates (Sarstedt AG, Nümbrecht, Germany). After incubation overnight, cells were supplemented with half logarithmic dilution series of the indicated compounds and DPBS (PAN Biotech GmbH, Aidenbach, Germany) as control. Then 72 h after addition of cytostatic treatment, the MTT solution (20 μL, 5 mg/mL) was added into the wells for 1 h at 37 °C and 5% CO_2_ until formazan crystals were formed. The supernatant was removed, and the cells were dissolved in 200 μL of DMSO. Absorption was analyzed at 570 nm, with background subtraction at 690 nm, using a plate reader (Thermomultiscan EX, Thermo, Schwerte, Germany). Cell viability was then determined by nonlinear regression and a four-parameter logistic equation with variable hill slope. All experiments were performed in triplicate (*n* = 4), and inflection points of sigmoidal curves were used to calculate EC_50_ values Prism (GraphPad Software, San Diego, CA, USA). EC_50_ was defined as the concentration that led to 50% viable cells. The significance of the difference of the effects of **33** and **4** + **57** was determined by using an unpaired, two-tailed *t* test with Welch’s correction, implemented in GraphPad Prism 8.4.0 (GraphPad, USA). Statistical significance was accepted if *p* < 0.05. Asterisks indicate *p < 0.05, **p < 0.01.

### Western Blotting

Western blots on histone H3 hyperacetylation in MCF-7 cells were performed according to a previously published protocol.^72^ In brief, MCF-7 were centrifuged (450*g*, 4 min, 22 °C) and lysed with cell extraction buffer (Thermo Fisher), supplemented with 0.1 mM PMSF, Halt Protease Inhibitor (Thermo Fisher), and Natriumorthovanadat (Thermo Fisher). Precast gels with a polymerization degree of 4–15% were used (Mini-PROTEAN TGX Stain-Free; Bio-Rad Laboratories GmbH, Munich, Germany). Proteins were transferred to Trans-Blot Turbo-PVDF membrane (Bio-Rad). The membrane was blocked with skimmed milk powder in Tris-buffered saline-Tween 20 (with 0.2% Tween 20) for 60 min, followed by three washing cycles of 10 min using Tris-buffered saline-Tween 20. Afterward, membranes were incubated with primary antibodies for ac.-Histon H3 (Cell Signal, rabbit) and GAPDH (Gene Tex, Irvine, USA, mouse) for a total of 60 min at room temperature and then incubated at 4 °C overnight. Membranes were rinsed again three times before applying the secondary antirabbit IgG HRP-conjugated mAbs (R&D Systems, Inc., Minneapolis, USA) or antimouse IgG HRP-conjugated mAbs (Santa Cruz Biotechnology, Texas, USA) for 90 min. Primary antibodies were diluted 1:500 (ac.-Histon H3) or 1:20.000 (GAPDH), respectively, and the secondary antibodies were diluted 1:20 000. For HDAC6 rescue experiments, precast gels with a polymerization degree of 10% were used (Mini-PROTEAN TGX Stain-Free; Bio-Rad Laboratories GmbH, Munich, Germany), and the primary antibody for HDAC6 (Cell Signal, rabbit) was diluted 1:1000. After rinsing of the secondary antibody, membranes were detected using ClarityECL Western Blotting Substrate (Bio-Rad). For quantitative determination, the StainFree technique was employed (Bio-Rad), as well as normalization, against the housekeeping protein GAPDH, which allows the imaging of whole lysates in SDS-PAGE before blotting and normalization against the total protein. Pixel density analysis was performed with the IMAGE LAB software (Bio-Rad).

### Crystallization

For cocrystallization experiments with Sirt2, human Sirt2_56–356_ was expressed and purified as described previously with minor modifications summarized hereafter.^18^ Sirt2_56–356_ was overexpressed in 2xYT medium (5 g/L NaCl, 16 g/L tryptone, 10 g/L yeast extract) using strain *E. coli* BL21 Star (DE3) at 20 °C overnight. Overexpression was induced with IPTG (isopropyl β-d-1-thiogalactopyranoside, final concentration of 1.0 mM) at an OD_600_ of 0.6–0.8. The cleavage of the His_10_-Tag via TEV protease was performed in lysis buffer (50 mM Tris/HCl, 500 mM NaCl, 10% (v/v) glycerol, 0.5 mM Tris(2-carboxyethyl)phosphine (TCEP), pH 8.0) at 4 °C for 36 h. Sirt2_56–356_ was applied on a HisTrap HP column (5 mL, GE Healthcare) again to obtain pure fractions of Sirt2_56–356_ with cleaved His_10_-Tag in the flowthrough. For the last purification step a Superdex S75 26/600 gel filtration column (20 mM HEPES, 150 mM NaCl, pH 7.5) was used. Crystallization assays were set up with the Oryx Nano pipetting robot (Douglas Instruments, East Garston, UK) using the vapor diffusion sitting drop method (MRC 2 Well UVP plate, SWISSCI, Buckinghamshire, England) at 20 °C. Sirt2_56–356_ (13.2 mg/mL) was incubated with 1.8 mM compound **5** (100 mM stock solution in DMSO, 1.8% (v/v) final DMSO concentration) on ice for 1 h prior to crystallization. To remove precipitates, the solution was centrifuged 10 min at 4 °C. Crystals of the Sirt2–**5** complex formed after 30 days in wells with a 1:1 ratio of 0.3 μL protein solution to 0.3 μL reservoir solution containing 25% (w/v) PEG 3,350 in 0.1 M Bis-Tris at pH 6.5. The crystals were cryoprotected with a mixture of 10% (v/v) 2*R*,3*R*-(−)-butanediol and reservoir solution, mounted on a nylon loop, and flash-cooled in liquid nitrogen.

For co-crystallization experiments with HDAC6, the catalytic domain 2 of HDAC6 from *Danio rerio* (for simplicity, referred to as “HDAC6” hereafter) encoded in the His6-MBP-TEV-HDAC6-pET28a-(+) vector was heterologously expressed in *Escherichia coli* BL21(DE3) cells. Protein was purified as described.^73^ The HDAC6–**55** and HDAC6–**57** complexes were crystallized via sitting drop vapor diffusion at 13 °C. Briefly, the protein solution [10.2 mg/mL HDAC6, 50 mM 4-(2-hydroxyethyl)-1-piperazineethanesulfonic acid (HEPES) (pH 7.5), 100 mM KCl, 5% glycerol (v/v), 1 mM tris(2-carboxyethyl)-phosphine] was mixed with 2 mM inhibitor and equilibrated on ice at 4 °C for 1 h. The enzyme–inhibitor solution was spin-filtered with a 0.22 μm centrifuge filter prior to crystallization. Using a mosquito crystallization robot (SPT Labtech), a 300 nL drop of enzyme–inhibitor solution was mixed with a 150 nL drop of precipitant solution from the Additive Screen (Hampton Research) [0.1 M bis-Tris (pH 6.0), 0.2 M KSCN, 20% PEG 3350 (w/v)] and 25 nL of seeding solution prepared from crystals of wild-type HDAC6. The resulting 475 nL sitting drop was equilibrated against 80 μL of precipitant solution in the crystallization well reservoir. Precipitant solutions used for crystallization of HDAC6 complexes contained the following additives: HDAC6–**55** complex, 0.2 M NDSB-211 (Hampton Research); HDAC6–**57** complex, 3% (v/v) ethanol. Crystals appeared after 1 day and grew to full size after 3 days. Prior to harvesting, crystals were soaked in a cryoprotectant solution containing mother liquor along with 25% (v/v) ethylene glycol and flash-cooled in liquid nitrogen.

### Data Collection and Structure Determination

For the Sirt2–**5** complex, X-ray diffraction data were collected on ID30A-3 (MASSIF-3) beamline^74^ at the European Synchrotron Radiation Facility (ESRF, Grenoble, France) using an Eiger X 4M detector. The data set was processed using autoPROC^75^ and scaled using Aimless.^76^ The structure was solved by molecular replacement with Phaser^77^ using the Sirt2–**3**–AcLysH3 complex (PDB 4RMH)^18^ as a search model. The model was built and refined in iterative steps with COOT^78^ and either REFMAC^79^ or Phenix.refine.^80^ Restraints for **5** were generated with the Grade Web Server (Global Phasing Ltd., United Kingdom). All residues except for Met299, Ile300, Met301, Gly302, and Leu303 of the Sirt2-specific insertion are defined in the electron density. Amino acids Gly102–Tyr114 exhibit poor electron density and higher *B*-factors compared to the rest of the protein. The electron density of **5** is well refined. Final structures were validated using MolProbity.^81^ All data collection and refinement statistics are reported in the Supporting Information (SI), Table S2.

For the HDAC6–**55** complex, X-ray diffraction data were collected on NE-CAT beamline 24-ID-C at the Advanced Photon Source, Argonne National Laboratory. Data were processed using Rapid Automated Processing of Data with X-ray detector software (XDS).^82^ For the HDAC6–**57** complex, X-ray diffraction data were collected on the NSLS-II FMX beamline at Brookhaven National Laboratory.^83^ Data were processed and analyzed using autoPROC.^75^ The initial electron density map of each complex was phased by molecular replacement using Phaser^77^ with the atomic coordinates of unliganded HDAC6 (PDB 5EEM)^49^ as a search model. Iterative model building and refinement cycles were performed using Coot^78^ and Phenix,^80^ respectively. In the final stages of refinement, inhibitor and solvent molecules were added into well-defined electron density. Final structures were validated using MolProbity.^81^ All data collection and refinement statistics are reported in SI, Table S2.

### Ligand Docking

The 3D structures of HDAC6 and Sirt2 were taken from the crystal structures solved in this study (Sirt2 cocrystallized with **5**, PDB 8OWZ, HDAC6 cocrystallized with **55**, PDB 8G1Z and **57**, PDB 8G20). Protein preparation and ligand docking were performed using the Schrödinger software version 2021.3v.^84^ Protein structure preparation for docking was done using the Protein Preparation Wizard within Schrödinger software using default setting. This included addition of missing hydrogen atoms and amino acid side chains, removal of solvent molecules and metal ions, optimization of protonation states and minimization applying the OPLS Force Field implemented in Schrödinger version 2021.3v. Docking was done using Glide in standard precision (SP) mode. The grid box including the information on active site coordinates of the proteins was defined with 20 Å radius around the ligand. Ten docking poses were employed for each ligand. The other settings were kept as default. The docking setup was previously used for Sirt2/HDAC inhibitor docking and could be validated by the structures of cocrystallized inhibitors.^19,85,86^ Redocking procedure was also able to reproduce the binding mode of **5** in Sirt2 with RMSD value of 0.227 Å and that of **55**/**57** in HDAC6 with RMSD values of 0.548 and 1.448 Å, respectively (crystal structures of the present work, 8G1Z, 8G20, and 8OWZ). RMDS value of **57** was slightly higher due to the flexibility of the two *n*-butyl chains in the capping group.

### Molecular Dynamics (MD) Simulations

GPU-based MD simulations were performed using the AMBER22 program.^87^ With the *pdb4amber* command, analysis, and cleaning of the protein–ligand complexes’ PDB files were done for further usage within the tLEaP program. Generations of the topology and force field parameters of the ligands were done with the Antechamber tool.^88^ Semi empirical AM1-BCC (Austin Model 1 with bond charge correction) atomic charge model were used.^89^ Using the AMBER22 tLEaP module the protein–ligand complexes were generated. The AMBER force field (GAFF2) was used as ligand force field, while force field 14 Stony Brook-ff14SB was used for the protein structures.^90^ For the catalytic Zn^2+^ ion the 12–6–4 LJ-type nonbonded ion model was applied.^91^ The final complexes were solvated by TIP3P water model as octahedral box around the protein (10 Å margin). Na^+^ and Cl^–^ ions were added for neutralization of the whole system. Parameter/topology files of the entire system were saved to use as starting point of the MD simulations. The solvated and neutralized systems were subjected to two energy minimization steps involving 1000 cycles of steepest descent followed by 2000 cycles of conjugate gradient minimization. In the first minimization solvent molecules and counterions (Na^+^ and Cl^–^) were minimized using a force constant of 10 kcal·mol^–1^·Å^–2^ for the protein, ligands, and zinc ion. In the second minimization step, the whole system was minimized. Afterward, the entire system was heated over 100 ps from 0 to 300 K. Constant volume periodic boundary was set to equilibrate the temperature of the system by Langevin thermostat using a collision frequency of 2 ps^–1^. Thereafter, a pressure equilibration routine at 300 K was performed for 100 ps with a constant pressure of 1 bar. Ultimately, free MD simulation utilizing the Particle Mesh Ewald method with the time step of 2 fs was applied for 100 ns. During these 100 ns, the system temperature was kept at 300 K and pressure of the system was maintained at 1 bar implementation of the SHAKE algorithm was done to constrain all bond containing hydrogens. As resul0,t 1000 frames were written for each 100 ns long simulation. Simulations of the crystal structures as well as the docking poses were repeated two times with nonidentical random seeds. Figures were generated using Pymol implemented in the Schrödinger software suite.

### PAINS Analysis

For the identification of potential pan-assay-interferers (PAINS), all compounds were reviewed using http://zinc15.docking.org/patterns/ home/. None of the final compounds (**21**, **22**, **31**–**33**, **44**–**46**, **55**–**57**) was flagged as PAINS. Intermediates **34**, **38**, and **42** were flagged due their azide group but were only used for synthesis and were not evaluated in biological assays.

## Supporting Information Available

Supplementary figures and tables, NMR spectra, and HPLC chromatograms (PDF)
Crystal structure of Sirt2 in complex with **5** (PDB)
Crystal structure of *Danio rerio* HDAC6 CD2 in complex with **55** (PDB)
Crystal structure of *Danio rerio* HDAC6 CD2 in complex with **57** (PDB)
Molecular formula strings for compounds (CSV)

## Supplementary Material

jm3c01385_si_001.pdf

jm3c01385_si_002.pdb

jm3c01385_si_003.pdb

jm3c01385_si_004.pdb

## Accession Codes

Protein Data Bank (PDB): Sirt2–**5** complex, 8OWZ; HDAC6–**55** complex, 8G1Z; HDAC6–**57** complex, 8G20. Authors will release the atomic coordinates upon article publication.

## Abbreviations Used

AMC: 7-amino-4-methylcoumarin
AR: androgen receptor
CD: catalytical domain
DAD: diode array detector
DIPEA: *N*,*N*-diisopropylethylamine
EtOAc: ethyl acetate
HATU: 1-[bis(dimethylamino)methylene]-1*H*-1,2,3-triazolo[4,5-*b*]pyridinium 3-oxide hexafluorophosphate
HDAC: histone deacetylase
HEPES: 4-(2-hydroxyethyl)-1-piperazineethanesulfonic acid
HOBt: 1-hydroxybenzotriazole
K-RAS: Kirsten rat sarcoma virus protein
LSD1: lysine-specific histone demethylase 1
mTOR: mammalian target of rapamycin
NAD^+^: nicotinamide adenine dinucleotide
NanoBRET: nano bioluminescence resonance energy transfer
MBP: maltose-binding protein
PEG: polyethylene glycol
PROTAC: proteolysis targeting chimera
RMSD: root mean-square deviation
SAHA: suberoylanilide hydroxamic acid
SD: standard deviation
SirReal: sirtuin rearranging ligand
Sirt: sirtuin
SMILES: simplified molecular input line entry specification
TBTA: tris(benzyltriazolylmethyl)amine
TBTU: 2-(1*H*-benzotriazole-1-yl)-1,1,3,3-tetramethyluronium tetrafluoroborate
TCEP: tris(2-carboxyethyl)phosphine
TEV: tobacco etch virus
TLC: thin layer chromatography
TNBS: 2,4,6-trinitrobenzenesulfonic acid
ZMAL: Z-(Ac)Lys-AMC
