# Supplementary material for: Development of First-in-Class Dual Sirt2/HDAC6 Inhibitors as Molecular Tools for Dual Inhibition of Tubulin Deacetylation
Source: J Med Chem. 2023 Oct 30;66(21):14787–814. doi: 10.1021/acs.jmedchem.3c01385 (PMC10641818; doi:10.1021/acs.jmedchem.3c01385)
Supplement: Supplementary file 1 — jm3c01385_si_001.pdf [file jm3c01385_si_001.pdf]

Supporting Information

Development of First-in-Class Dual Sirt2/HDAC6 Inhibitors as Molecular Tools for Dual Inhibition of Tubulin Deacetylation

Laura Sinatra,[§,#] *Anja Vogelmann,*[Δ,#] *Florian Friedrich,*[Δ] *Margarita A. Tararina,*[□] *Emilia Neuwirt,*[ǁ,‡] *Arianna Colcerasa,*[Δ] *Philipp König,*[◊] *Lara Toy,*[⊥] *Talha Z. Yesiloglu,*[ǂ] *Sebastian Hilscher,*[ǂ,≡] *Lena Gaitzsch,*[Δ] *Niklas Papenkordt,*[Δ] *Shiyang Zhai,*[◊] *Lin Zhang,*[⌂] *Christophe Romier,*[⁼] *Oliver Einsle,*[⌂] *Wolfgang Sippl,*[ǂ] *Mike Schutkowski,*[≡] *Olaf Gross,*[ǁ,‡,$] *Gerd Bendas,*[◊] *David W. Christianson,*[□] *Finn K. Hansen,*[◊,§] *Manfred Jung,*[Δ] *Matthias Schiedel,*[⊥,Ŧ,*]

[§] Institute for Drug Discovery, Medical Faculty, Leipzig University, Brüderstraße 34, 04103 Leipzig, Germany

[Δ] Institute of Pharmaceutical Sciences, University of Freiburg, Albertstraße 25, 79104 Freiburg, Germany

[□] Roy and Diana Vagelos Laboratories, Department of Chemistry, University of Pennsylvania, 231 South 34th Street, Philadelphia, Pennsylvania 19104-6323, United States

[ǁ] Institute of Neuropathology, Medical Center – University of Freiburg, Faculty of Medicine, University of Freiburg, Breisacherstraße 64, 79106 Freiburg, Germany

[‡] CIBSS – Centre for Integrative Biological Signalling Studies, University of Freiburg, Schänzlestraße 18, 79104 Freiburg, Germany

[◊] Department of Pharmaceutical & Cell Biological Chemistry, Pharmaceutical Institute, University of Bonn, An der Immenburg 4, 53121 Bonn, Germany

[⊥] Department of Chemistry and Pharmacy, Medicinal Chemistry, Friedrich-Alexander-University Erlangen-Nürnberg, Nikolaus-Fiebiger-Straße 10, 91058 Erlangen, Germany

[ǂ] Department of Medicinal Chemistry, Institute of Pharmacy, Martin-Luther University of Halle-Wittenberg, Wolfgang-Langenbeck-Straße 2-4, 06120 Halle (Saale), Germany

[⌂] Institute of Biochemistry, University of Freiburg, Albertstraße 21, 79104 Freiburg, Germany

[⁼] Institut de Génétique et de Biologie Moléculaire et Cellulaire (IGBMC), Université de Strasbourg, CNRS UMR 7104, Inserm UMR-S 1258, 1 rue Laurent Fries, F-67400 Illkirch, France

[≡] Department of Enzymology, Charles Tanford Protein Center, Institute of Biochemistry and Biotechnology, Martin-Luther-University Halle-Wittenberg, 06120 Halle, Germany

[$] Center for Basics in NeuroModulation (NeuroModulBasics), Faculty of Medicine, University of Freiburg, Breisacherstraße 64, 79106 Freiburg, Germany

[Ŧ] Institute of Medicinal and Pharmaceutical Chemistry, Technische Universität Braunschweig, Beethovenstraße 55, 38106 Braunschweig, Germany

*Correspondence: Prof. Dr. Matthias Schiedel, Institute of Medicinal and Pharmaceutical Chemistry, Technische Universität Braunschweig, Beethovenstraße 55, 38106 Braunschweig, Germany,
Email: matthias.schiedel@tu-braunschweig.de

Table of Contents

S3	Supplementary Figures
S14	Supplementary Tables
S16	NMR Spectra
S95	HPLC Chromatograms
S106	Supplementary References

Supplementary Figures

Figure S1. The dual Sirt2/HDAC6 inhibitor **33** also shows an inhibition of Sirt2-mediated demyristoylation in a biochemical activity assay,[1] which is based on the conversion of a myristoylated peptide substrate (triplicate measurement, see Experimental Section for experimental details). The Sirt2 selective inhibitor **5**,[2] which was previously shown to inhibit Sirt2-mediated demyristoylation,[3] was used as a control.

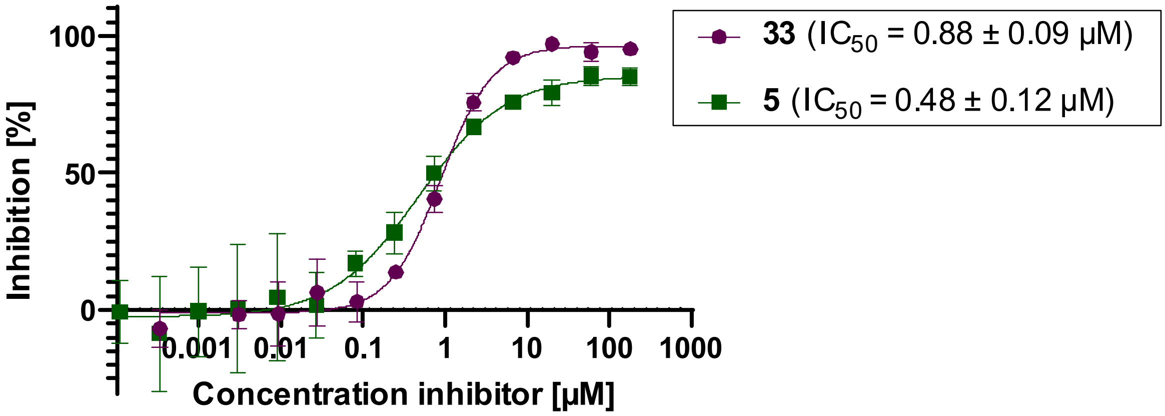

Figure S2. Results of the MD simulation of the Sirt2-**5** crystal structure (PDB ID: 8OWZ) for two repeated MD runs (100 ns). a) RMSD plot of protein heavy atoms. b) RMSD plot of ligand heavy atoms. RMSD plot of MD 1 is colored red, for MD run 2 it is colored cyan.

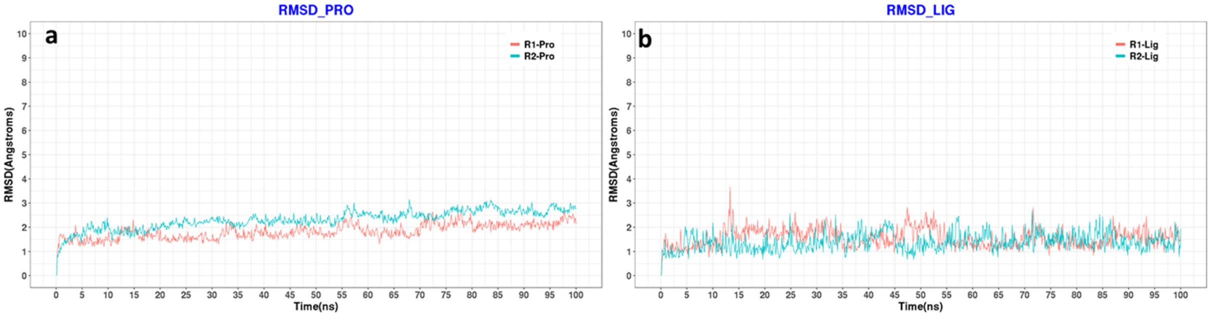

Figure S3. Results of the MD simulation of the HDAC6-**57** crystal structure (PDB ID 8G20) for two repeated MD runs (100 ns). a) RMSD plot of protein heavy atoms. b) RMSD plot of ligand heavy atoms. c) RMSD plot of the zinc ion. d) Distance plot of the coordination of the hydroxamate oxygens O1 (NO^-) and O2 (CO) to the zinc ion. RMSD plot of MD 1 is colored red, for MD run 2 it is colored cyan.

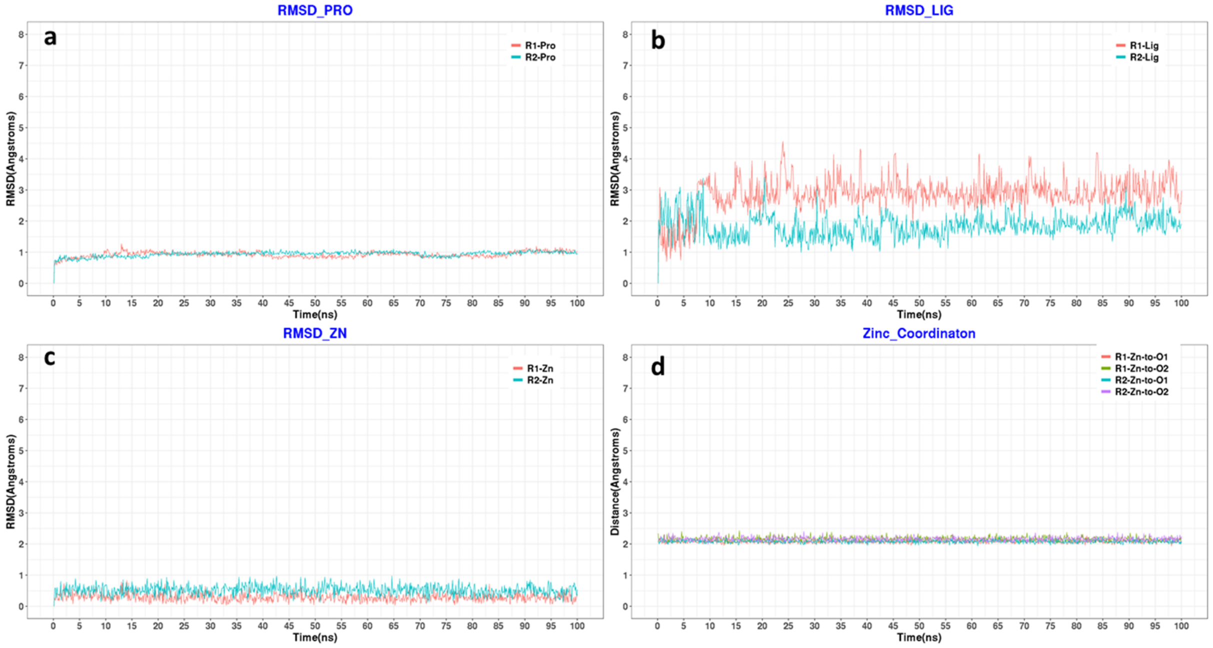

Figure S4. Results of the MD simulation of the Sirt2-**33** docking complex for two repeated MD runs (100 ns). a) RMSD plot of protein heavy atoms. b) RMSD plot of ligand heavy atoms. RMSD plot of MD 1 is colored red, for MD run 2 it is colored cyan.

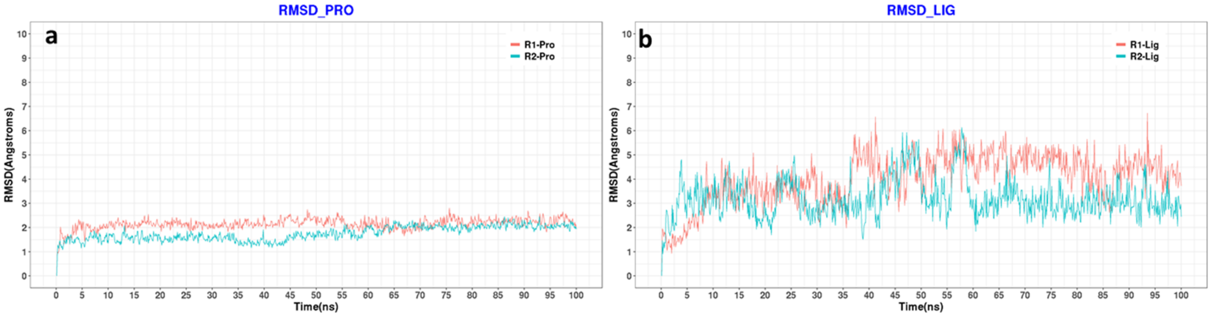

Figure S5. Results of the MD simulation of the Sirt2-**32** docking complex for two repeated MD runs (100 ns). a) RMSD plot of protein heavy atoms. b) RMSD plot of ligand heavy atoms. RMSD plot of MD 1 is colored red, for MD run 2 it is colored cyan.

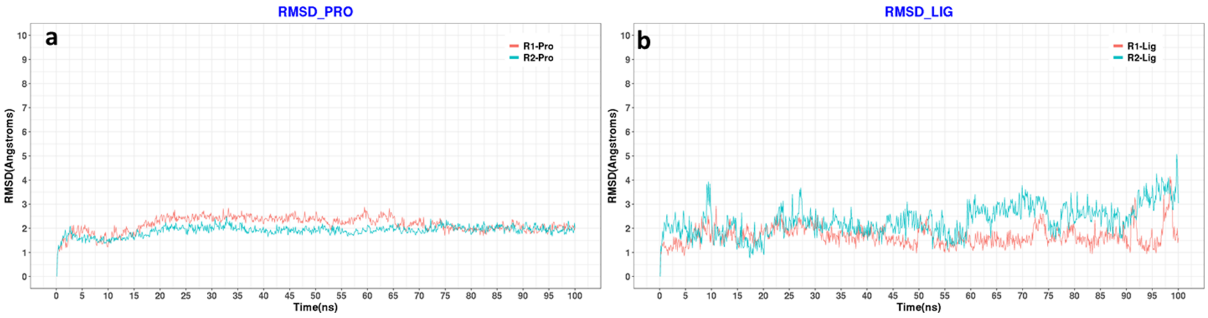

Figure S6. Shown are selected frames of MD simulations of Sirt2 ligand complexes at 0 ns (lime carbon atoms), 50 ns (magenta carbon atoms) and 100 ns (yellow carbon atoms). Ligands are shown as sticks, proteins are shown as white cartoons for clarity. a) Frames of the MD run 1 of Sirt2-**5** crystal structure (PDB ID: 8OWZ). b) Frames of the MD run 2 of Sirt2-**5** crystal structure (PDB ID: 8OWZ). c) Frames of the MD run 1 of Sirt2-**33** docking complex. d) Frames of the MD run 2 of Sirt2-**33** docking complex. e) Frames of the MD run 1 of Sirt2-**32** docking complex. f) Frames of the MD run 2 of Sirt2-**32** docking complex.

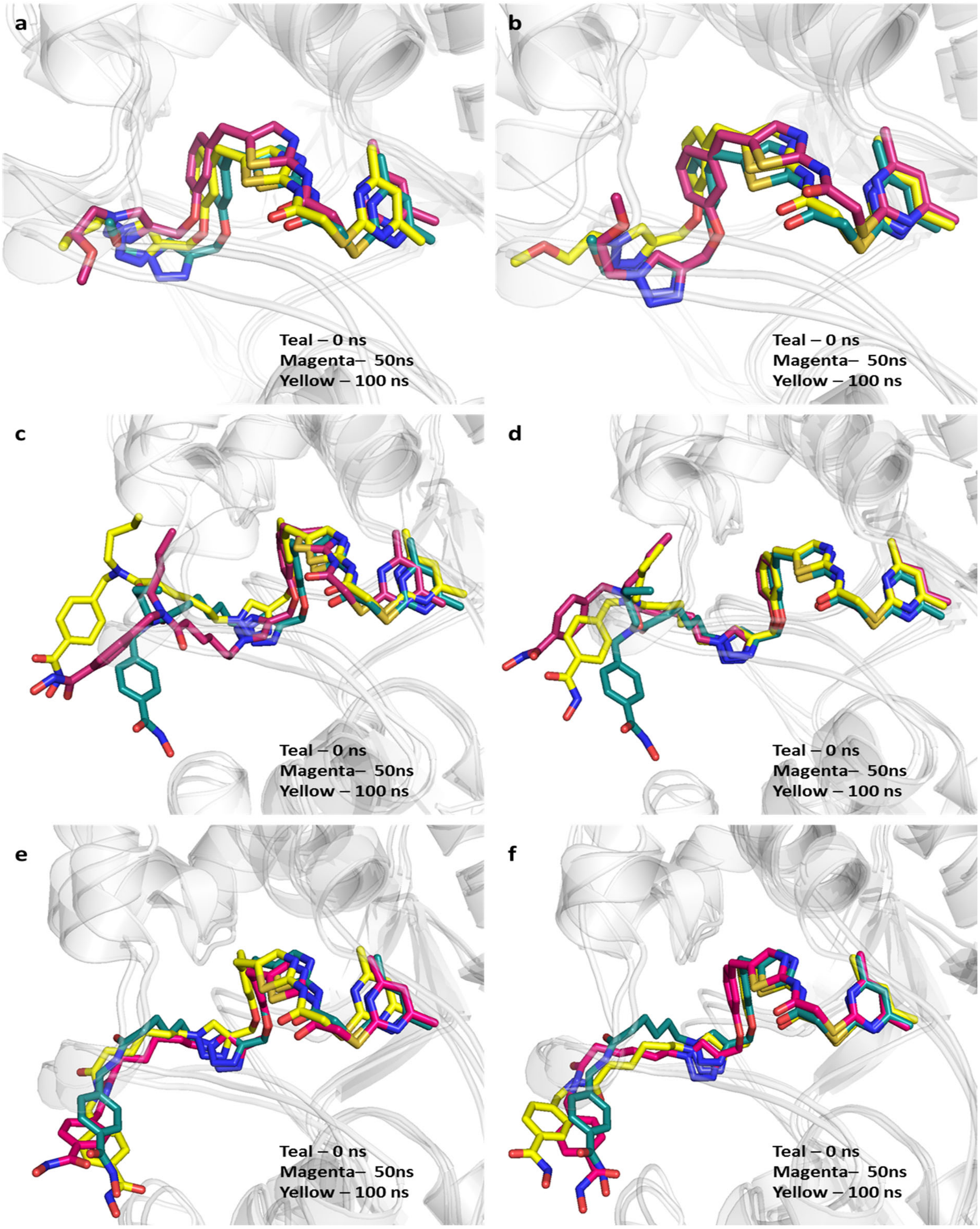

Figure S7. Results of the MD simulation of the HDAC6-**33** docking complex for two repeated MD runs (100 ns). a) RMSD plot of protein heavy atoms. b) RMSD plot of ligand heavy atoms. c) RMSD plot of the zinc ion. d) Distance plot of the coordination of the hydroxamate oxygens O1 (NO^-) and O2 (CO) to the zinc ion. RMSD plot of MD 1 is colored red, for MD run 2 it is colored cyan.

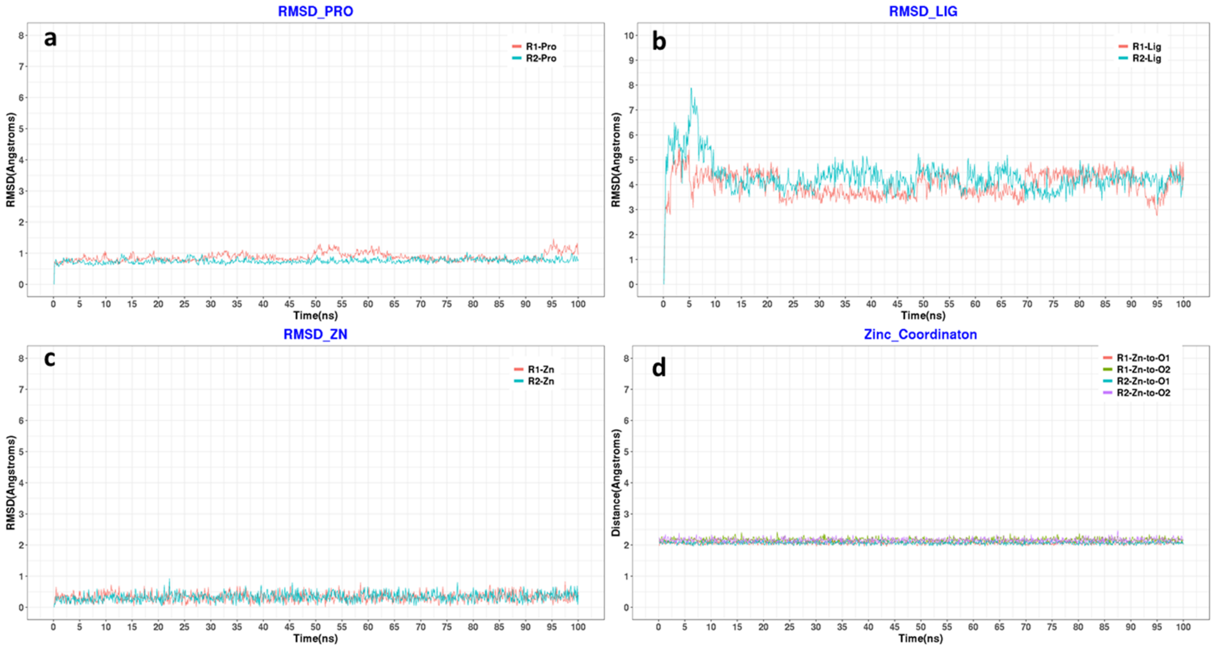

Figure S8. Results of the MD simulation of the HDAC6-**32** docking complex for two repeated MD runs (100 ns). a) RMSD plot of protein heavy atoms. b) RMSD plot of ligand heavy atoms. c) RMSD plot of the zinc ion. d) Distance plot of the coordination of the hydroxamate oxygens O1 (NO^-) and O2 (CO) to the zinc ion. RMSD plot of MD 1 is colored red, for MD run 2 it is colored cyan.

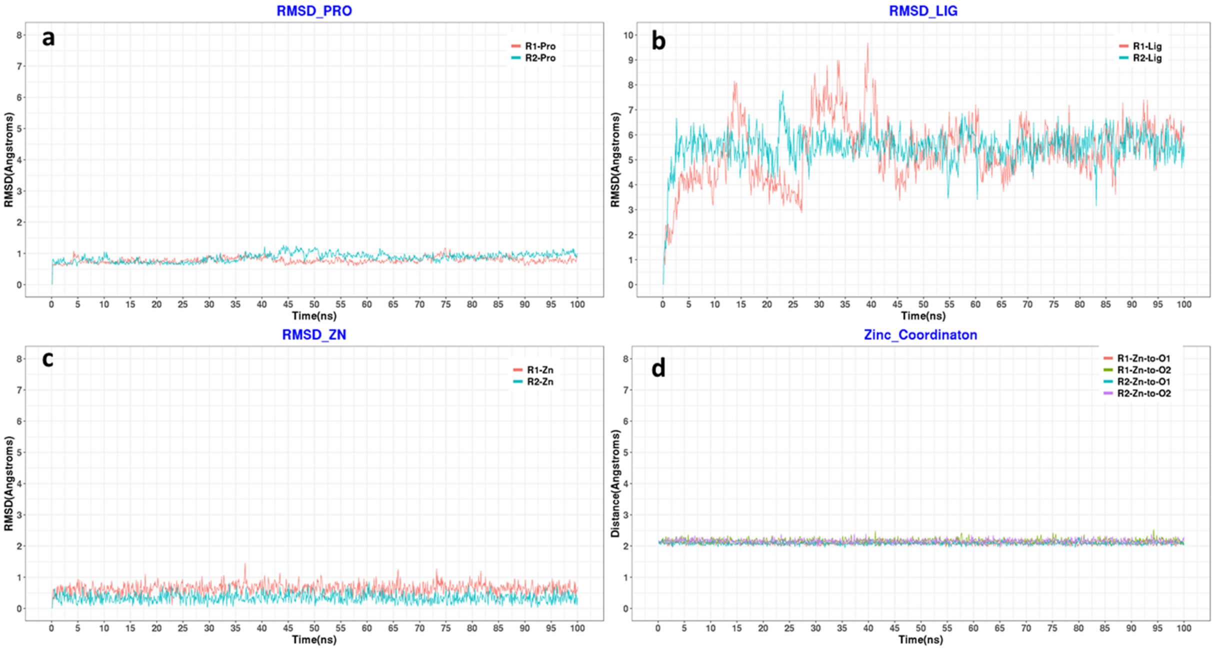

Figure S9. Shown are selected frames of the MD simulations of HDAC6-ligand complexes at 0 ns (lime carbon atoms), 50 ns (magenta carbon atoms) and 100 ns (yellow carbon atoms). Ligands are shown as sticks, proteins are shown as white cartoons for clarity and the zinc ions are shown as orange spheres. a) Frames of the MD run 1 of the HDAC6-**57** crystal structure. b) Frames of the MD run 2 of the HDAC6-**57** crystal structure. c) Frames of the MD run 1 of HDAC6-**33** docking complex and d) Frames of the MD run 2 of HDAC6-**33** docking complex. e) Frames of the MD run 1 of HDAC6-**32** docking complex. f) Frames of the MD run 2 of HDAC6-**32** docking complex.

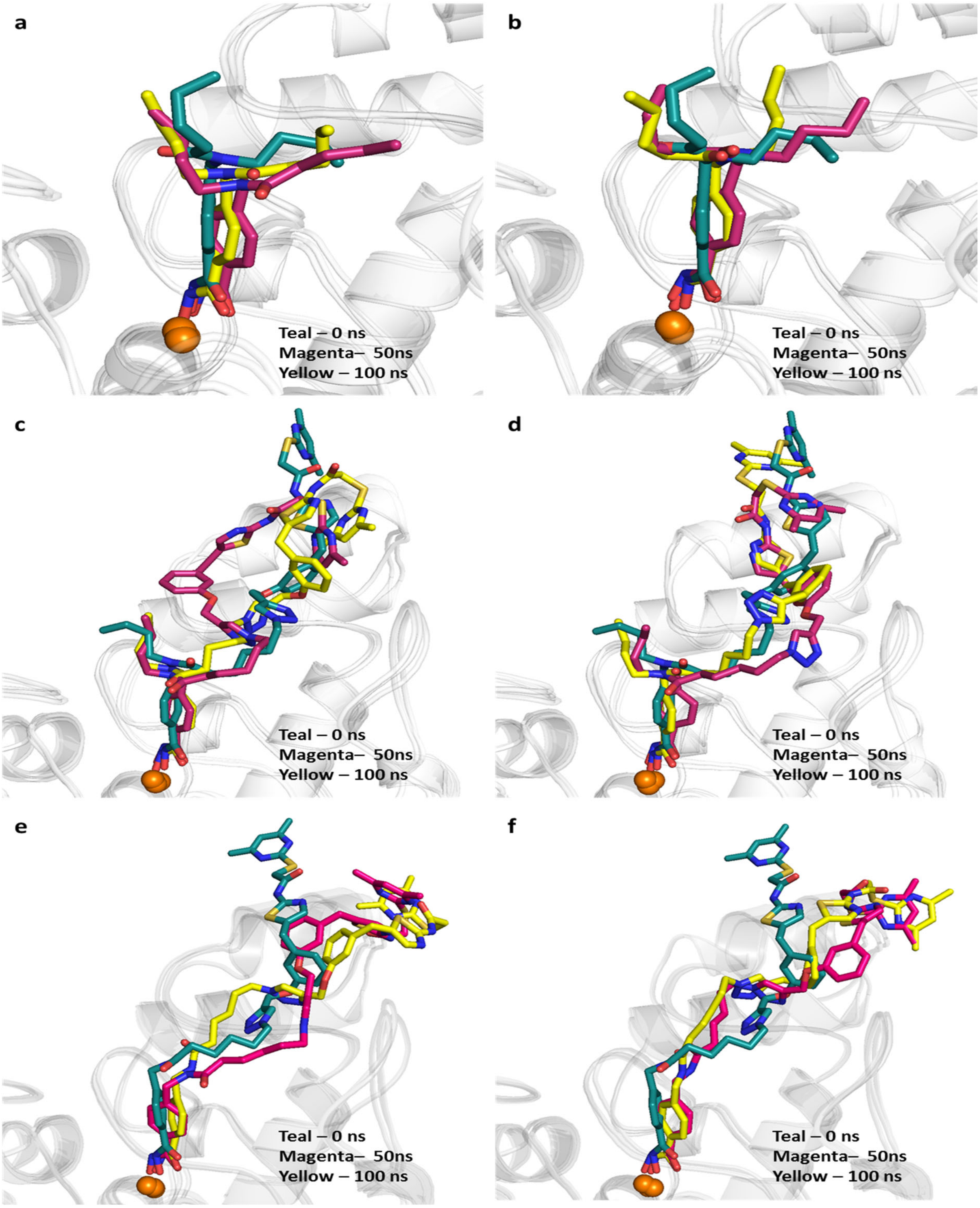

Figure S10. Chemical structures of molecular tools used to demonstrate cellular target engagement for the dual Sirt2/HDAC6 inhibitor **33**. A) Sirt2 selective fluorescent probe SirReal-TAMRA (**61**),[3, 4] which was used to demonstrate cellular Sirt2 target engagement for the dual Sirt2/HDAC6 inhibitor **33** *via* a cellular NanoBRET assay. B) HDAC6 selective PROTAC B4 (**62**),[5] which was used to demonstrate cellular HDAC6 target engagement for the dual Sirt2/HDAC6 inhibitor **33** *via* a degradation rescue experiment.

A

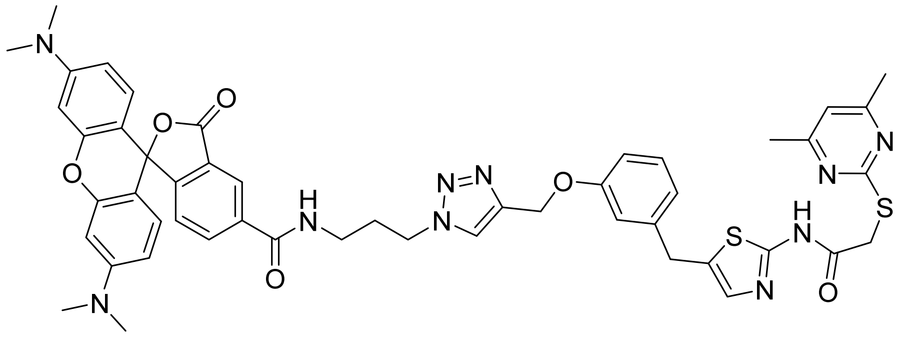

Sirt2-targeted fluorescent probe SirReal-TAMRA (**61**)

B

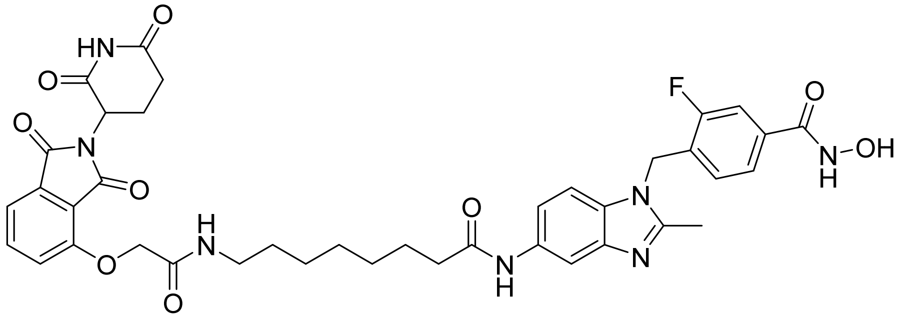

HDAC6-targeted PROTAC B4 (**62**)

Figure S11. Dual Sirt2/HDAC6 inhibitors **32** and **33** evoke no significant off-target inhibition of class I HDACs at a cellular level, as shown by the investigation of histone H3 hyperacetylation, a marker for cellular inhibition of HDAC1-3. Tests were performed with MCF-7 breast cancer cells, a well-established cell line for the investigation of class I HDAC inhibition.[6-8] A) Representative western blot from whole cell lysates of MCF-7 cells after 24 h treatment with dual Sirt2/HDAC6 inhibitors **32** or **33**, unconjugated Sirt2 inhibitor **4**, unconjugated HDAC6 inhibitor **57**, and the Sirt2/HDAC inhibitor **46**. SAHA was used as a positive control and DMSO as a vehicle control. Compounds were tested at a concentration of 10 µM. B) Western blot quantification (n = 3). C) Effects of **33** on histone H3 acetylation at compound concentrations of 10 µM and 20 µM. Representative western blot from whole cell lysates of MCF-7 breast cancer cells after 24 h treatment with the dual Sirt2/HDAC6 inhibitors **33**. D) Western blot quantification (n = 3).

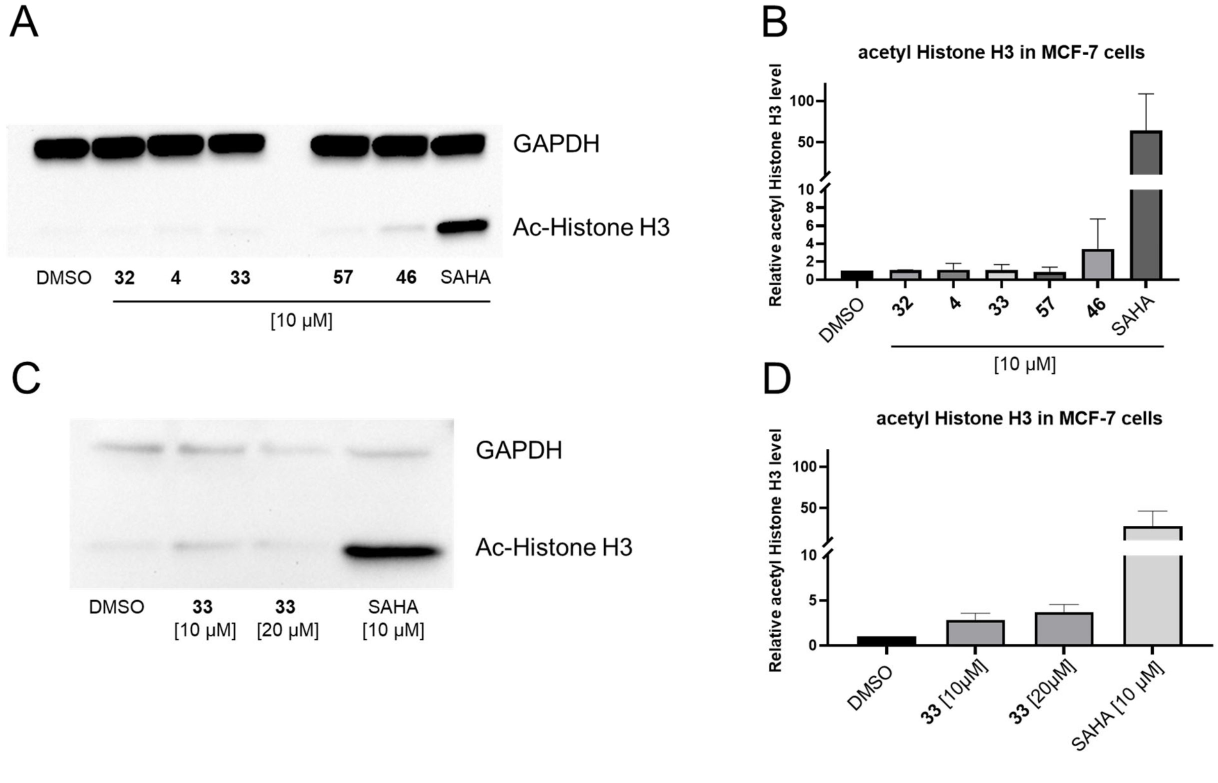

Supplementary Tables

Table S1. Dual Sirt2/HDAC6 inhibitor **33** tested by means of biochemical *in vitro* deacylation assays (see Experimental Section for more experimental details).[9-11] IC_{50} values [µM, mean ± SD] or percentual inhibition at a given concentration of the dual Sirt2/HDAC6 inhibitor. Nicotinamide (NA) and panobinostat (Pano) were used as reference compounds for sirtuin and Zn^{2+}-dependent HDAC inhibition, respectively.

Cmpd	Sirt5[a]	Sirt6[a]	HDAC4[b]	HDAC5[b]	HDAC7[b]	HDAC8[c]	HDAC9[b]	HDAC10[b]
**33**	> 500 µM (-1% @ 500 µM, -2% @ 200 µM)	> 500 µM (10% @ 500 µM, 0% @ 200 µM)	> 20 µM (7% @ 20 µM, 4% @ 6 µM)	> 6 µM (57% @ 20 µM, 18% @ 6 µM)	> 6 µM (57% @ 20 µM, 29% @ 6 µM)	2.94 ± 0.45 µM	> 20 µM (37% @ 20 µM, 17% @ 6 µM)	> 6 µM (64% @ 20 µM, 27% @ 6 µM)
NA	140 ± 17 µM	590 ± 73 µM	n.t.[d]	n.t.[d]	n.t.[d]	n.t.[d]	n.t.[d]	n.t.[d]
Pano	n.t.[d]	n.t.[d]	0.303 ± 0.038 µM	0.0423 ± 0.0116 µM	1.25 ± 0.91 µM	0.51 ± 0.05 µM	1.65 ± 0.83 µM	0.00262 ± 0.00002 µM

[a] Test performed in duplicate; [b] Tests performed in triplicate (n = 2); [c] Tests performed in triplicate; [d] n.t. = not tested

Table S2. Crystallographic data collection and refinement statistics for the Sirt2- and HDAC6-inhibitor complexes.

**Data Collection**			
Inhibitor	**5**	**55**	**57**
Protein	Sirt2	HDAC6	HDAC6
Space group	$P2_1$	$P2_1$	$P1$
a, b, c (Å)	35.81, 73.46, 55.33	55.66, 48.39, 74.52	48.24, 55.54, 74.40
α, β, γ (deg)	90.00, 95.34, 90.00	90, 106.18, 90	73, 89.90, 82.82
Wavelength (Å)	0.9677	0.97918	0.97934
Resolution (Å)	55.09 – 1.65 (1.68 – 1.65)	71.57 – 1.87 (1.91 – 1.87)	29.05 – 1.77 (1.81 – 1.77)
Total/unique no. of reflections	227,570/33,934	114,480/31,171	187,535/69,137
R_{merge}[a,b]	0.096 (1.016)	0.280 (1.308)	0.131 (0.552)
R_{pim}[a,c]	0.040 (0.411)	0.237 (1.164)	0.121 (0.352)
$CC_{1/2}$[a,d]	0.998 (0.741)	0.974 (0.389)	0.986 (0.649)
$I/\sigma(I)$[a]	11.3 (2.0)	7.6 (1.1)	5.4 (1.5)
Redundancy[a]	6.7 (7.0)	3.7 (2.9)	2.7 (2.6)
Completeness (%)[a]	98.7 (99.1)	98.3 (91.1)	96.1 (89.2)
**Refinement**			
No. of reflections used in refinement/test set	33,902 (3,429)	31,044 (2,903)	69,115 (6,516)
R_{work}[e]	0.168 (0.234)	0.204 (0.400)	0.192 (0.270)
R_{free}[f]	0.197 (0.250)	0.254 (0.424)	0.229 (0.344)
Number of Atoms[g]			2439
protein	2439	2780	5506
ligand	80	30	83
solvent	230	209	400
Average *B* Factors (Å^2)			
protein	26.5	26	15
ligand	32.1	34	20
solvent	31.0	31	19
RMS Deviations			
bonds (Å)	0.007	0.006	0.010
angles (deg)	0.94	0.79	0.99
Ramachandran plot (%)[h]			
favored	97.26	97.2	96.9
allowed	2.74	2.8	3.1
outliers	0	0	0
PDB accession code	8OWZ	8G1Z	8G20

[a] Values in parentheses refer to the highest-resolution shell of the data.

[b] $R_{merge} = \sum|I_h - \langle I_h\rangle|/\sum\langle I_h\rangle$; I_h = intensity measure for reflection *h;* $\langle I_h\rangle$ = average intensity for reflection *h* calculated from replicate data.

[c] $R_{pim} = \sum(1/(n-1))^{1/2}|I_h - \langle I_h\rangle|/\sum\langle I_h\rangle$; n = number of observations (redundancy).

[d] $CC_{1/2} = \sigma_\tau^2/(\sigma_\tau^2 + \sigma_\varepsilon^2)$, where σ_τ^2 is the true measurement error variance and σ_ε^2 is the independent measurement error variance.

[e] $R_{work} = \sum||F_o| - |F_c||/\sum|F_o|$ for reflections contained in the working set. $|F_o|$ and $|F_c|$ are the observed and calculated structure factor amplitudes, respectively.

[f] $R_{free} = \sum||F_o| - |F_c||/\sum|F_o|$ for reflections contained in the test set held aside during refinement.

[g] Per asymmetric unit.

[h] Assessed by MolProbity.

NMR spectra

NMR spectra for compounds **21-22**, **25-40**, **42-46** and **55-60** can be found on the following pages.

^{1}H NMR (400 MHz, DMSO-d_6) for compound **21**

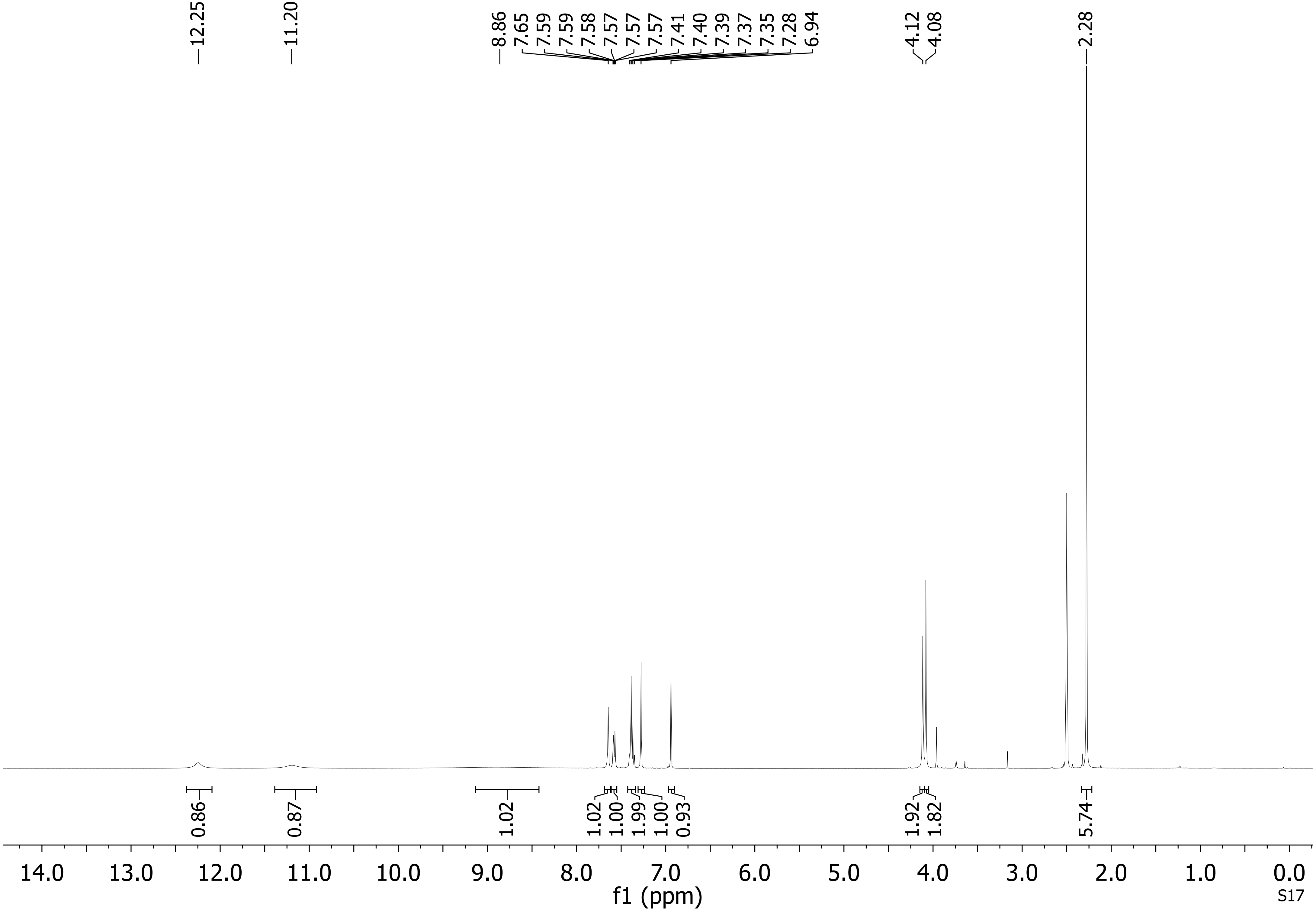

^{13}C NMR (151 MHz, DMSO-d_6) for compound **21**

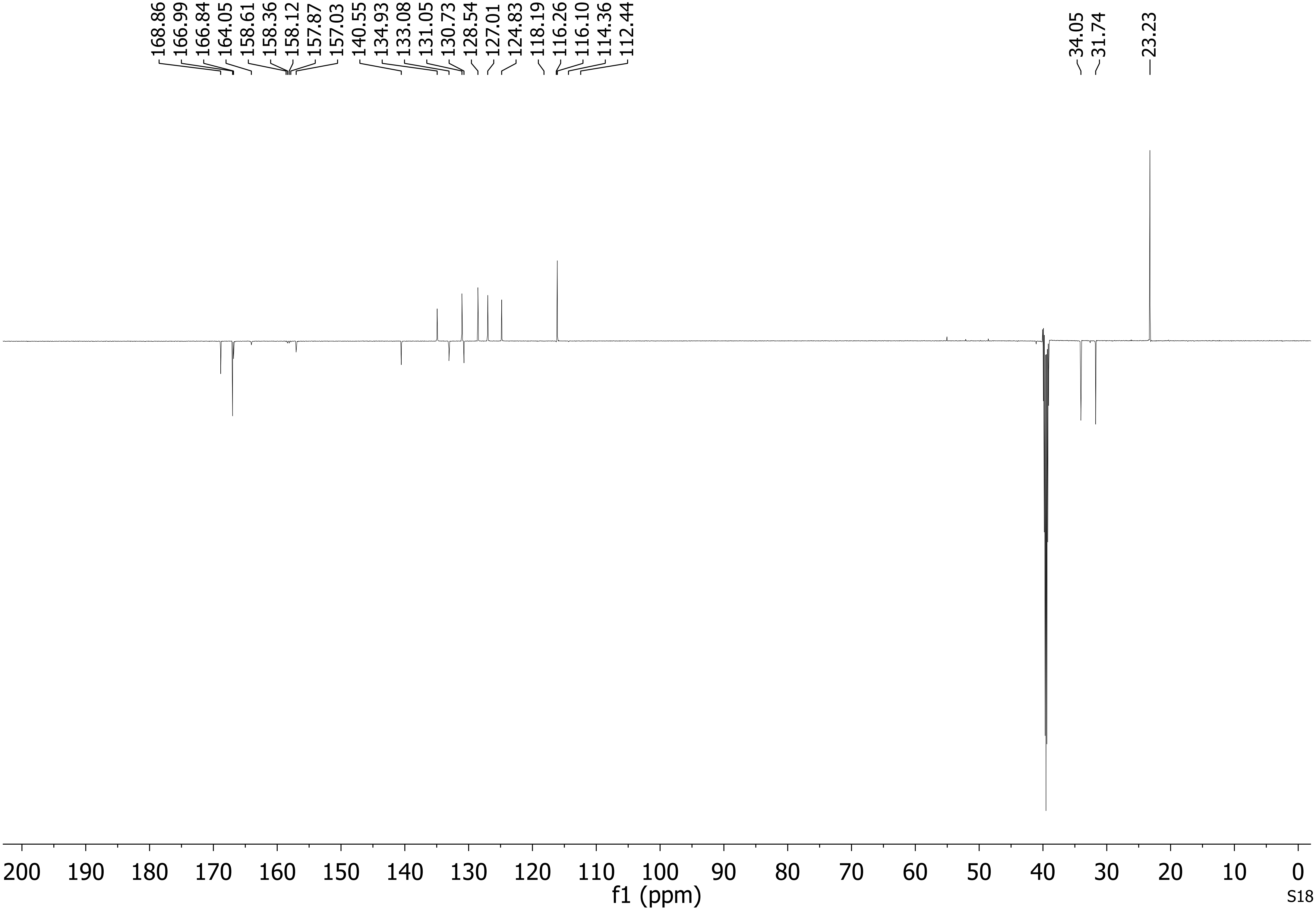

^{19}F NMR (377 MHz, DMSO-d_6) for compound **21**

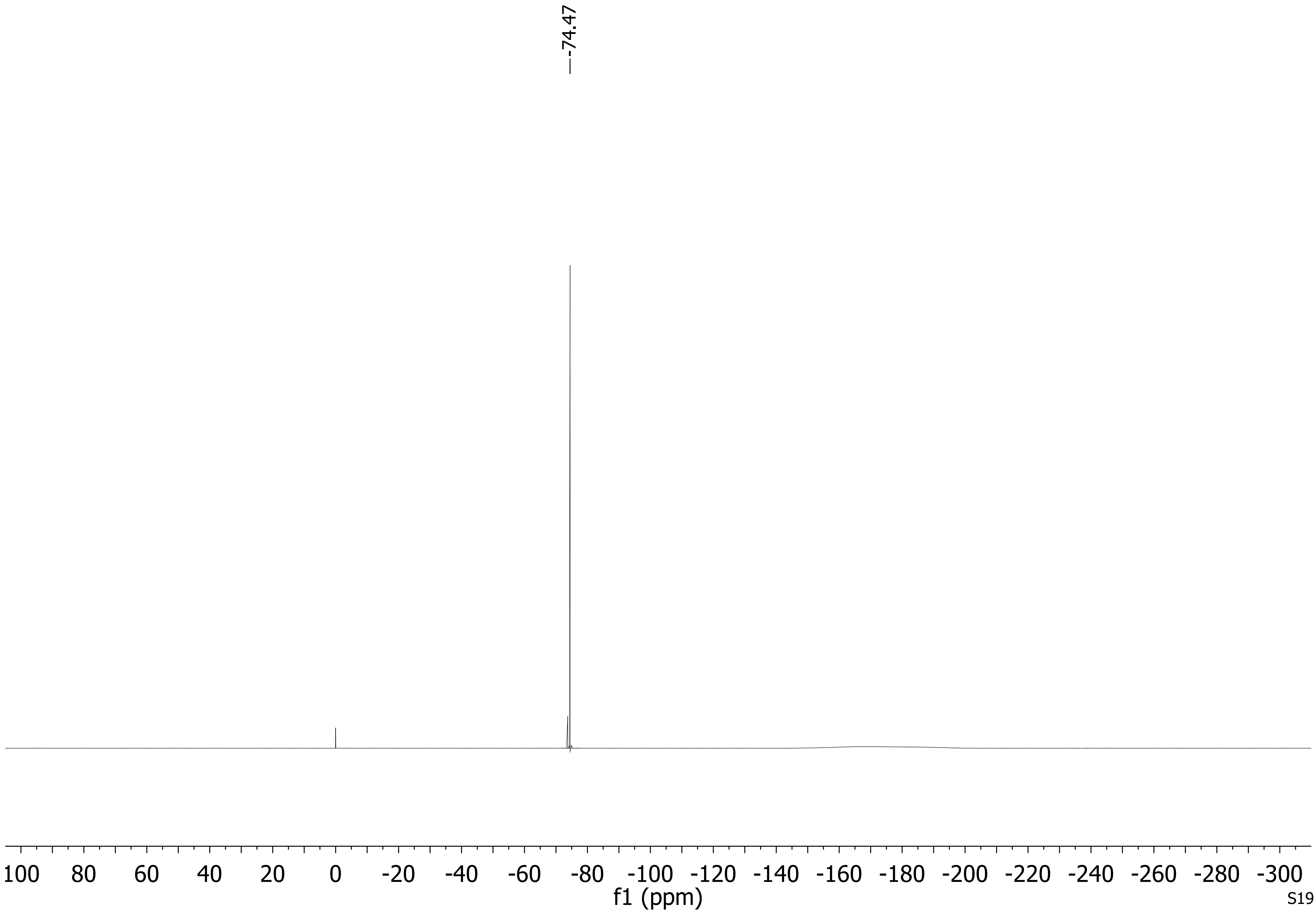

^{1}H NMR (400 MHz, DMSO-d_6) for compound **22**

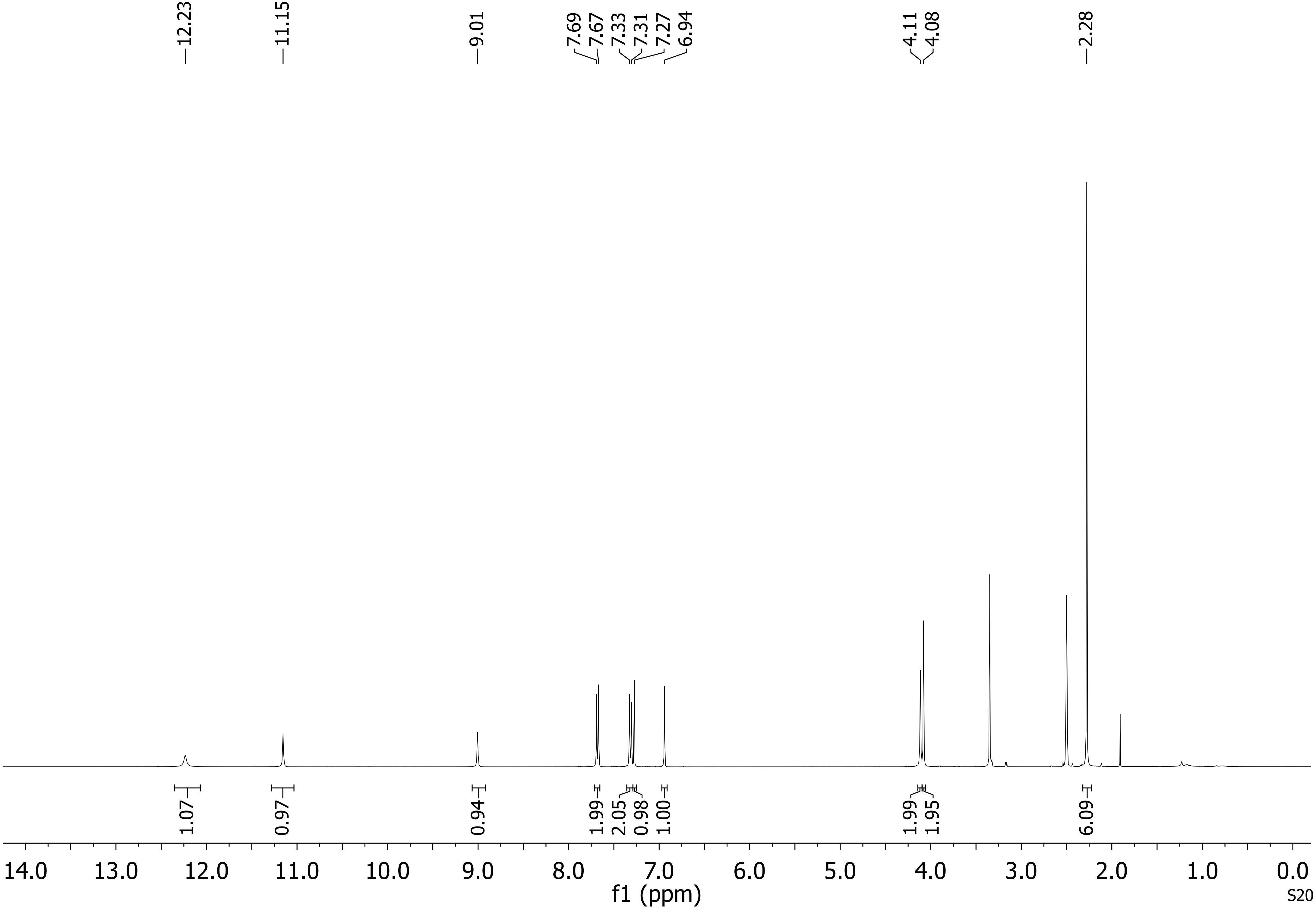

^{13}C NMR (101 MHz, DMSO-d_6) for compound **22**

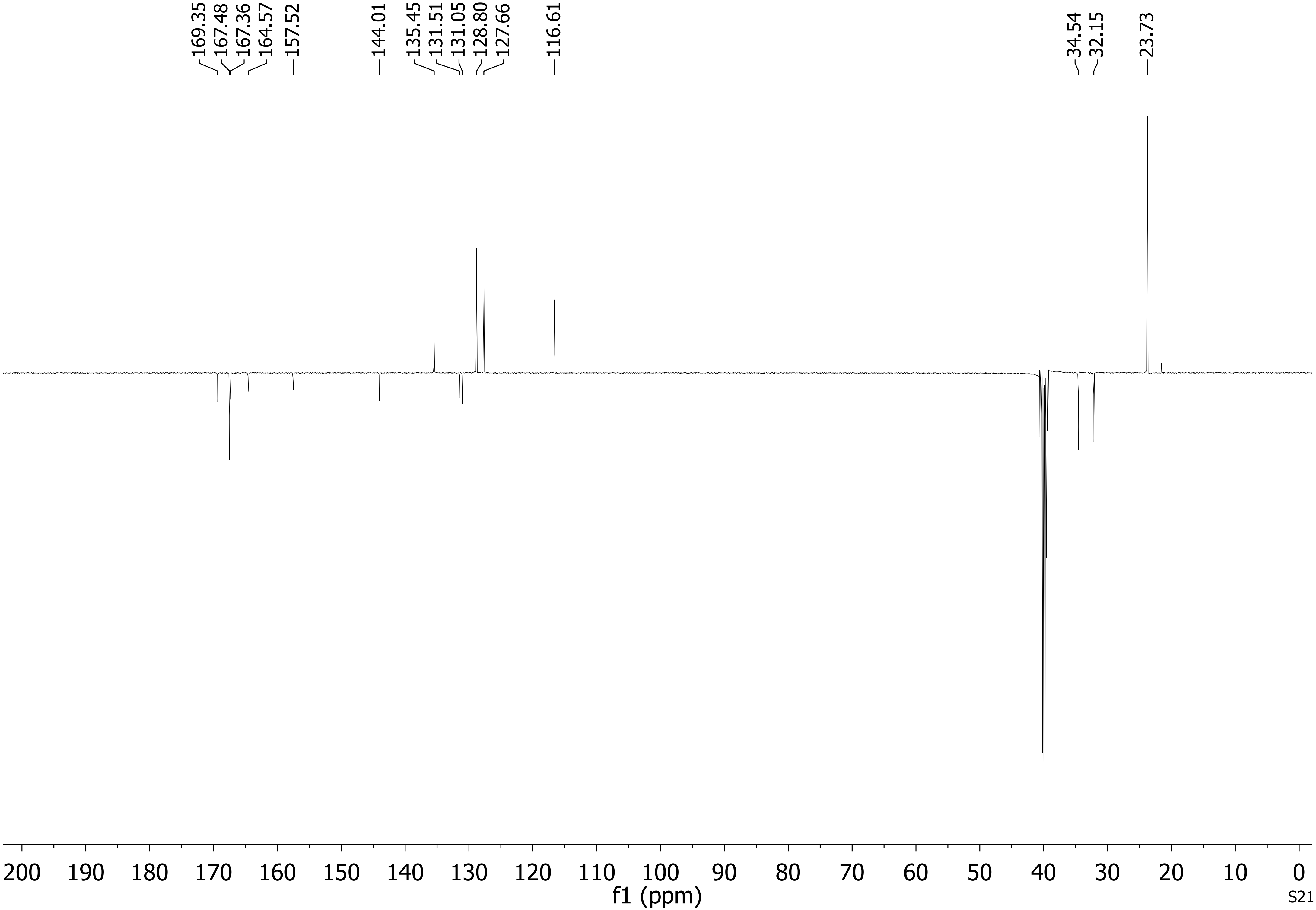

^{1}H NMR (400 MHz, DMSO-d_6) for compound **25**

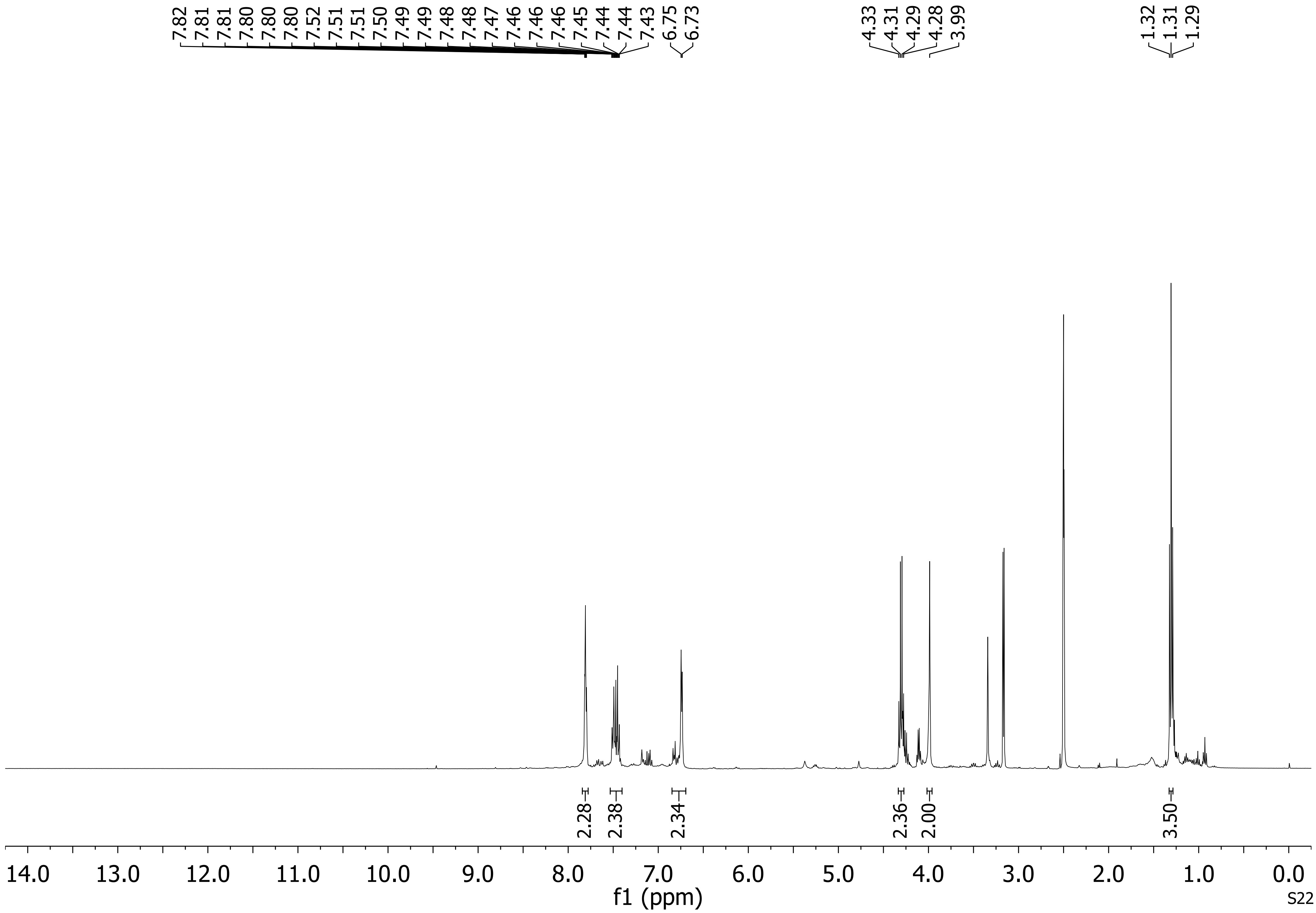

^{13}C NMR (151 MHz, DMSO-d_6) for compound **25**

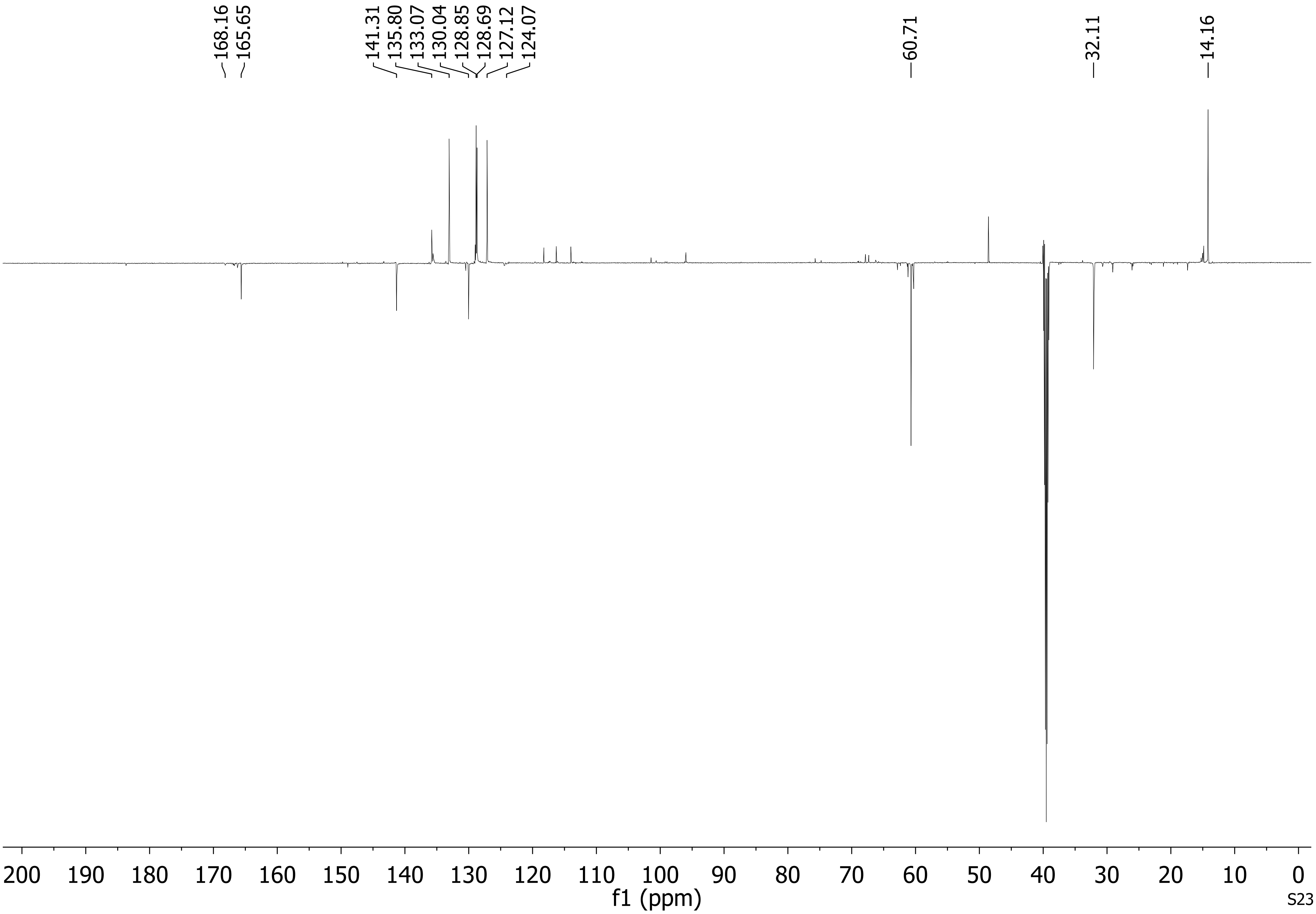

^{1}H NMR (400 MHz, DMSO-d_6) for compound **26**

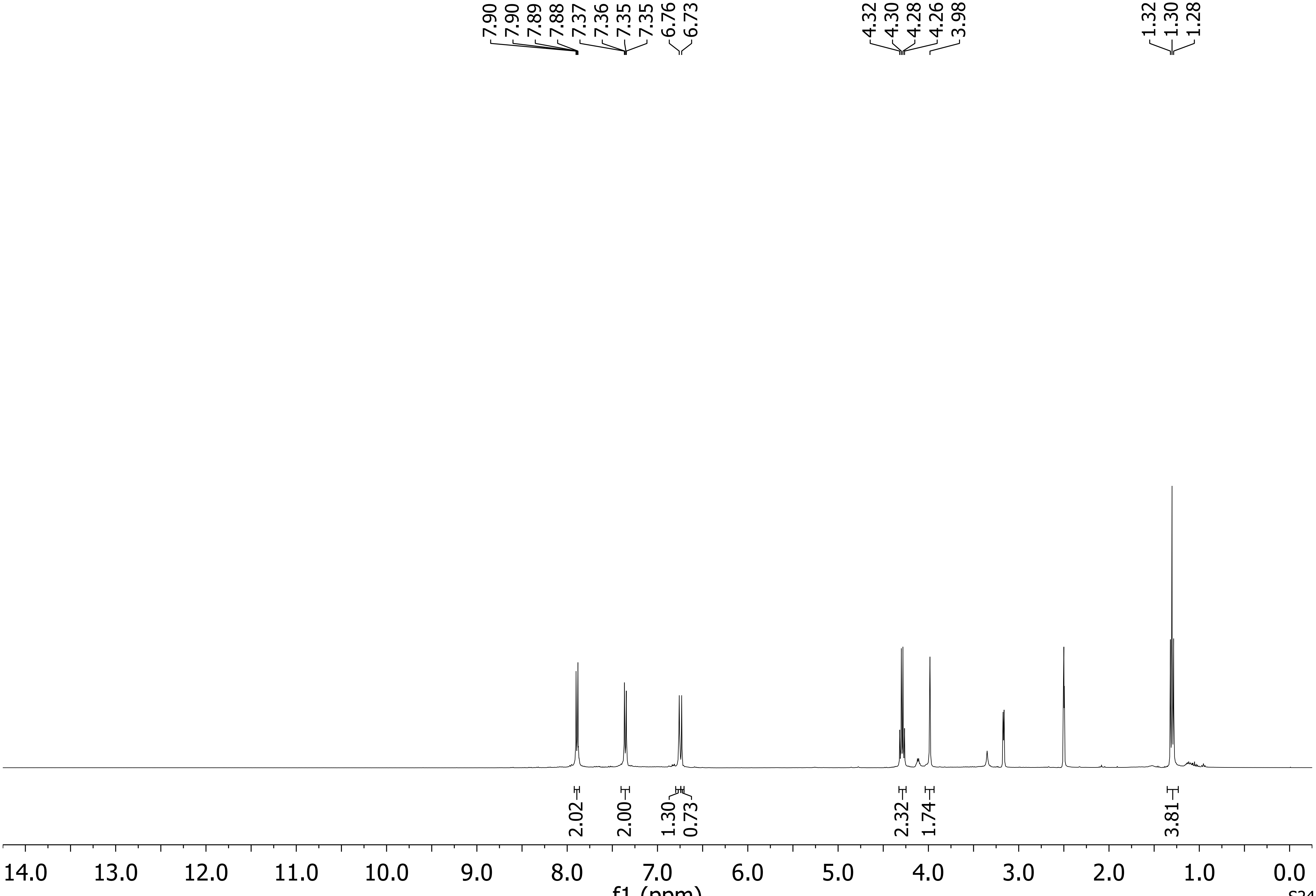

^{13}C NMR (151 MHz, DMSO-d_6) for compound **26**

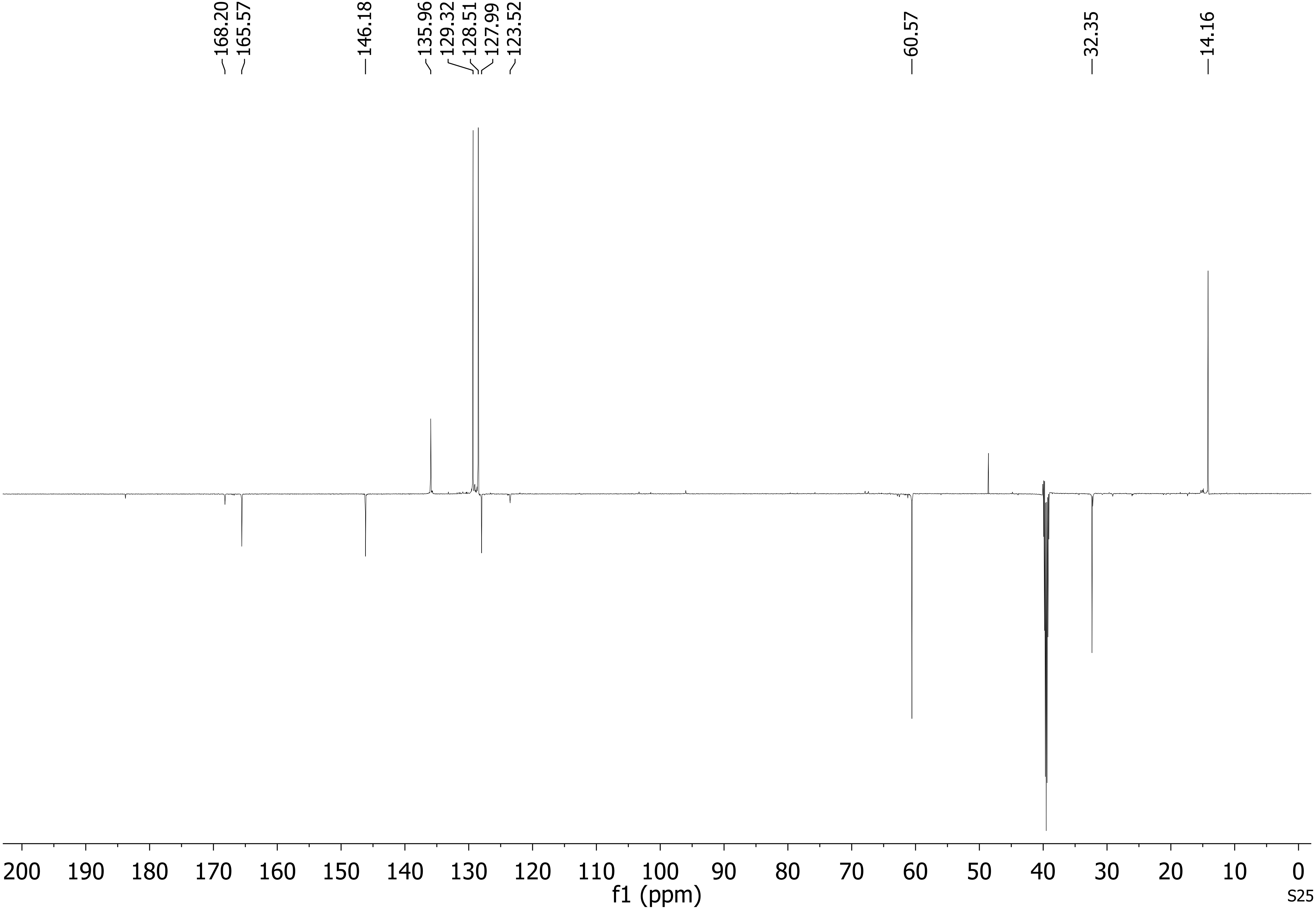

^{1}H NMR (400 MHz, DMSO-d_6) for compound **27**

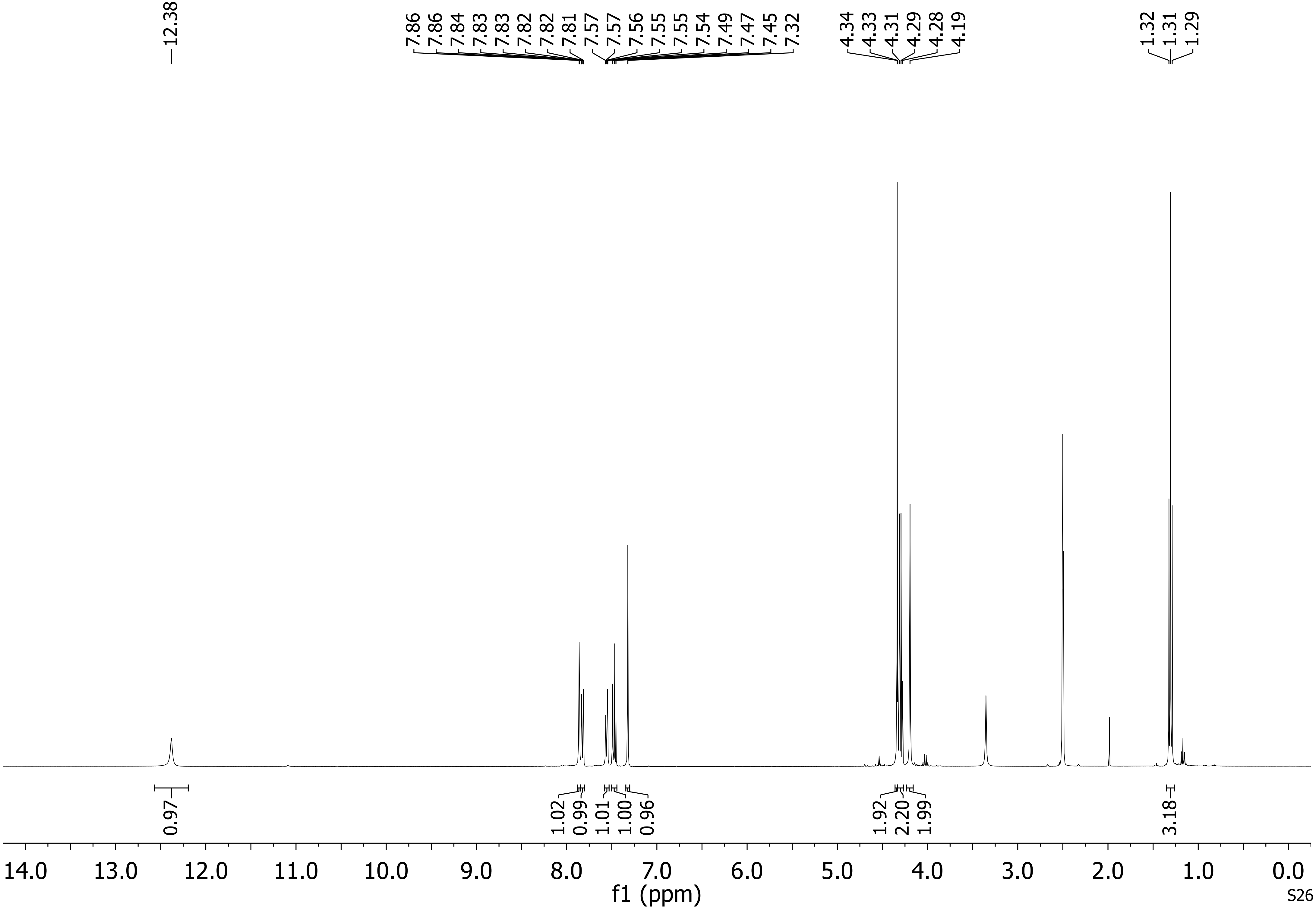

^{13}C NMR (151 MHz, DMSO-d_6) for compound **27**

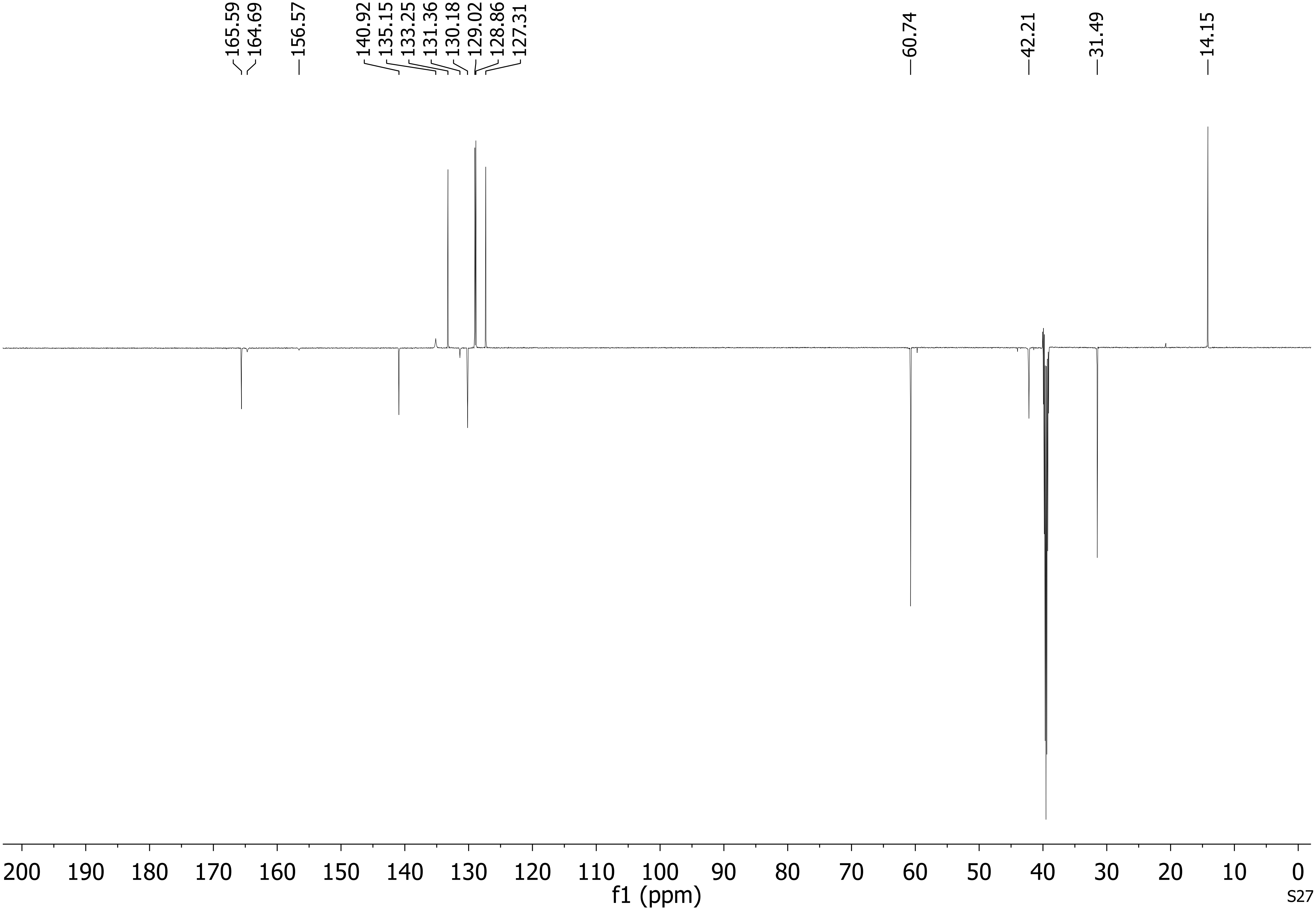

^{1}H NMR (400 MHz, DMSO-d_6) for compound **28**

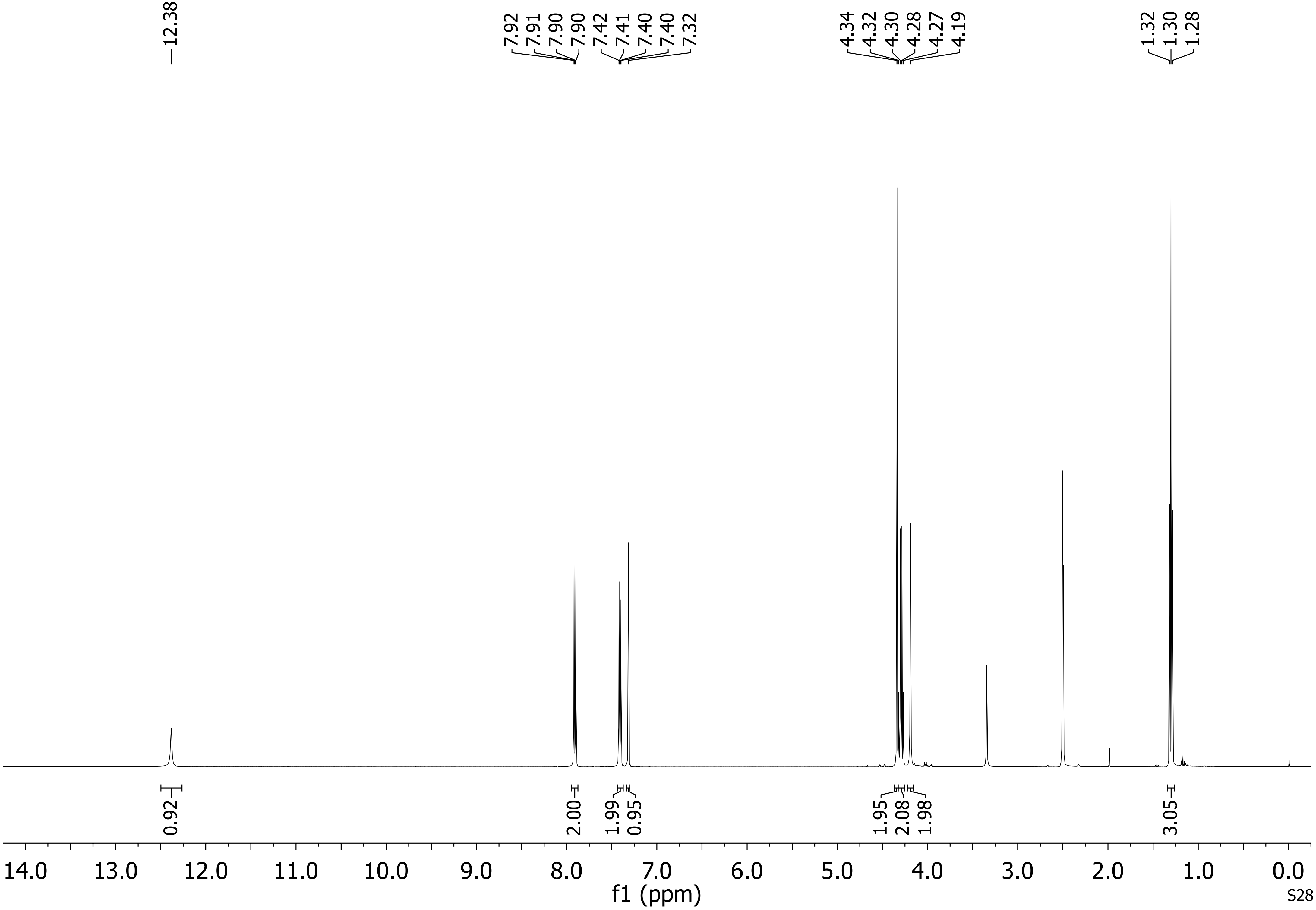

^{13}C NMR (151 MHz, DMSO-d_6) for compound **28**

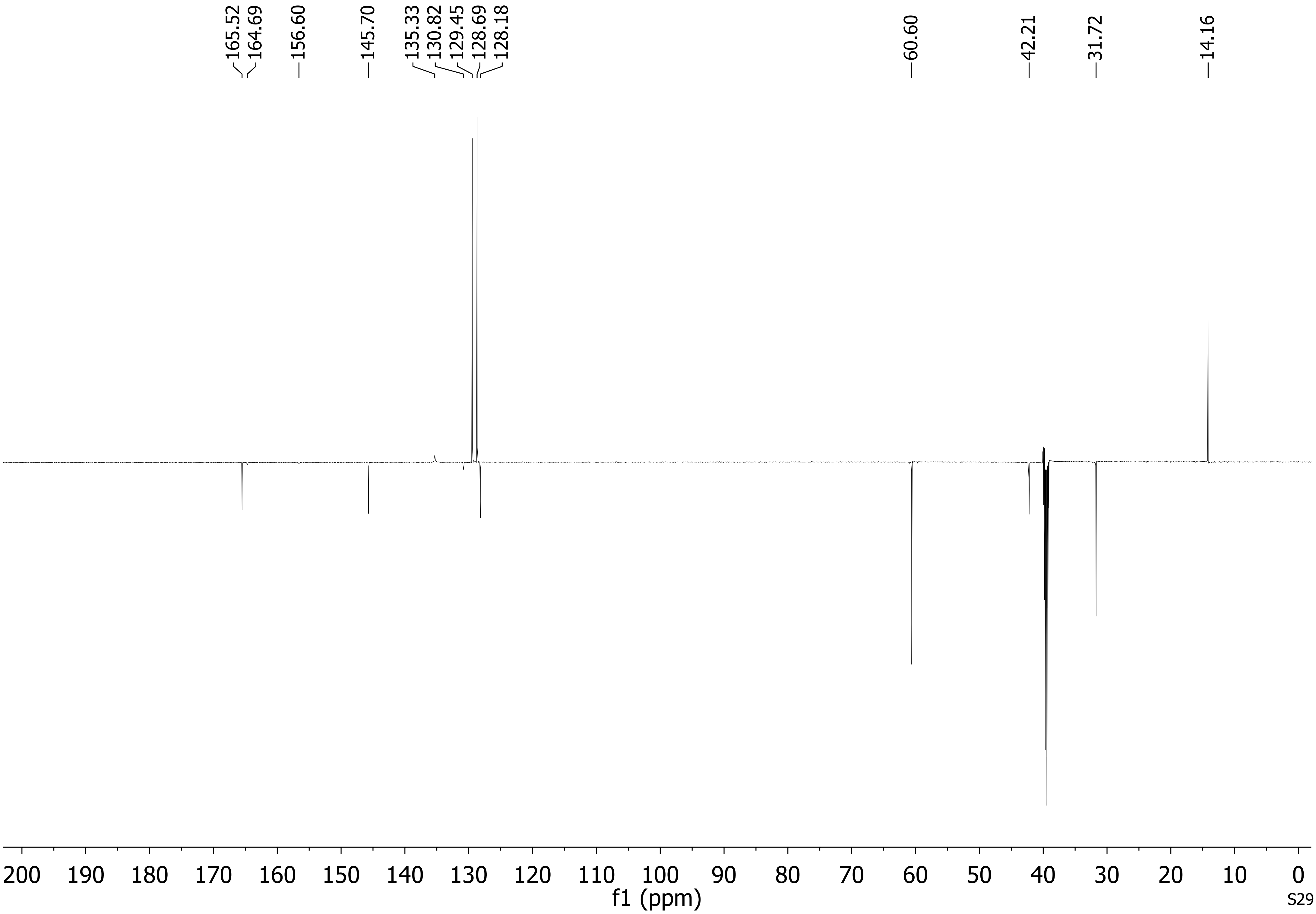

^{1}H NMR (400 MHz, DMSO-d_6) for compound **29**

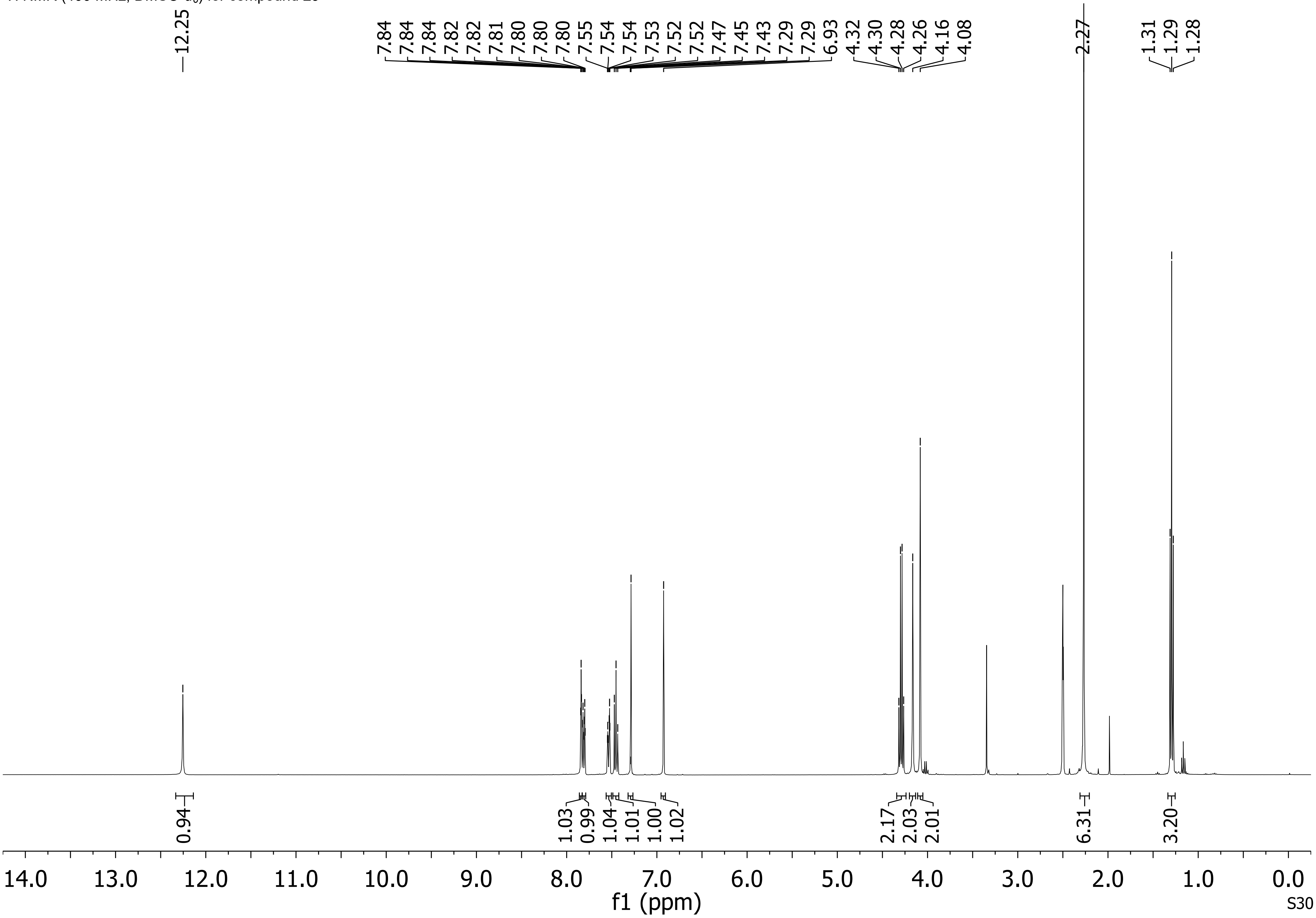

^{13}C NMR (151 MHz, DMSO-d_6) for compound **29**

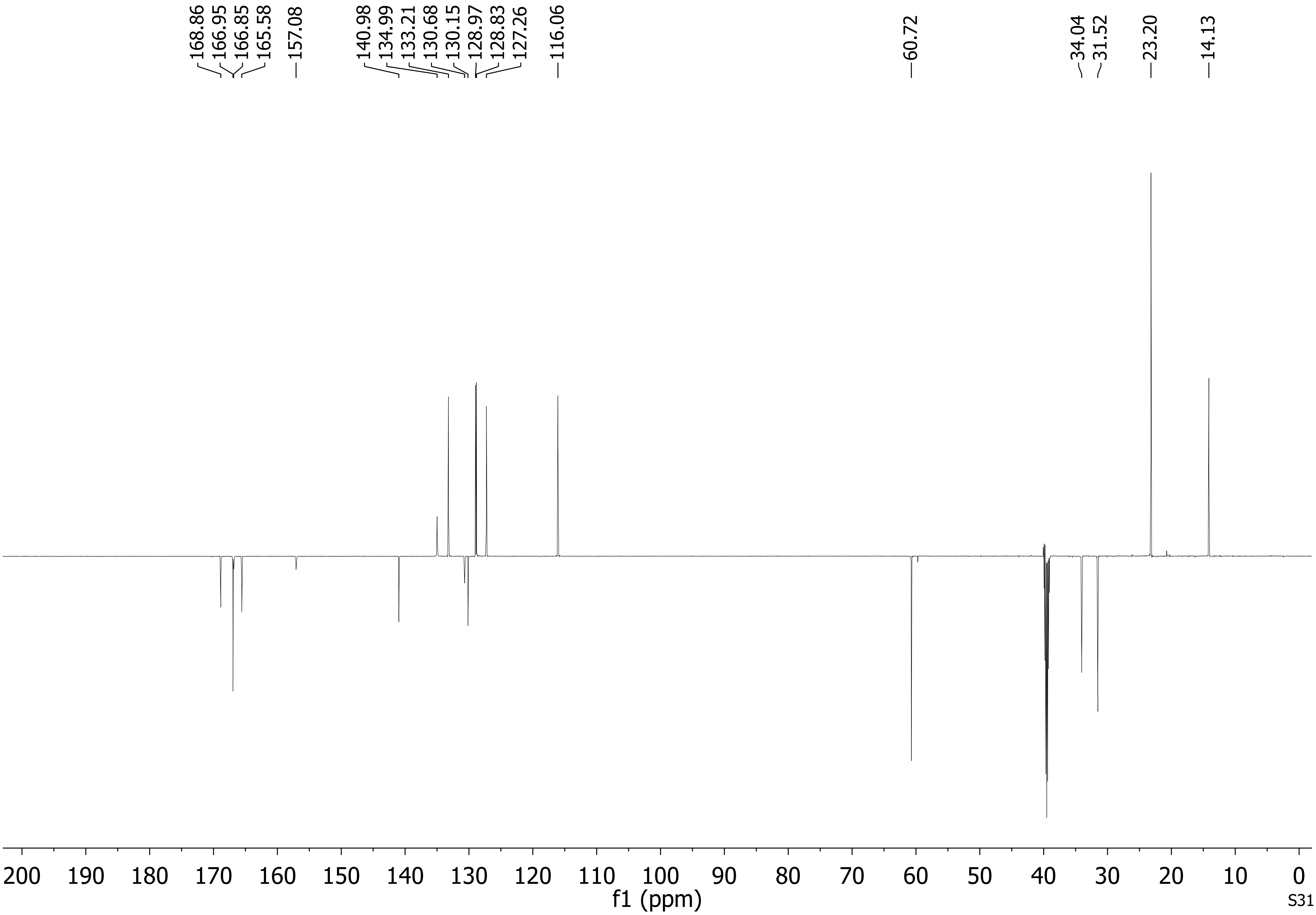

^1H NMR (400 MHz, DMSO-d_6) for compound **30**

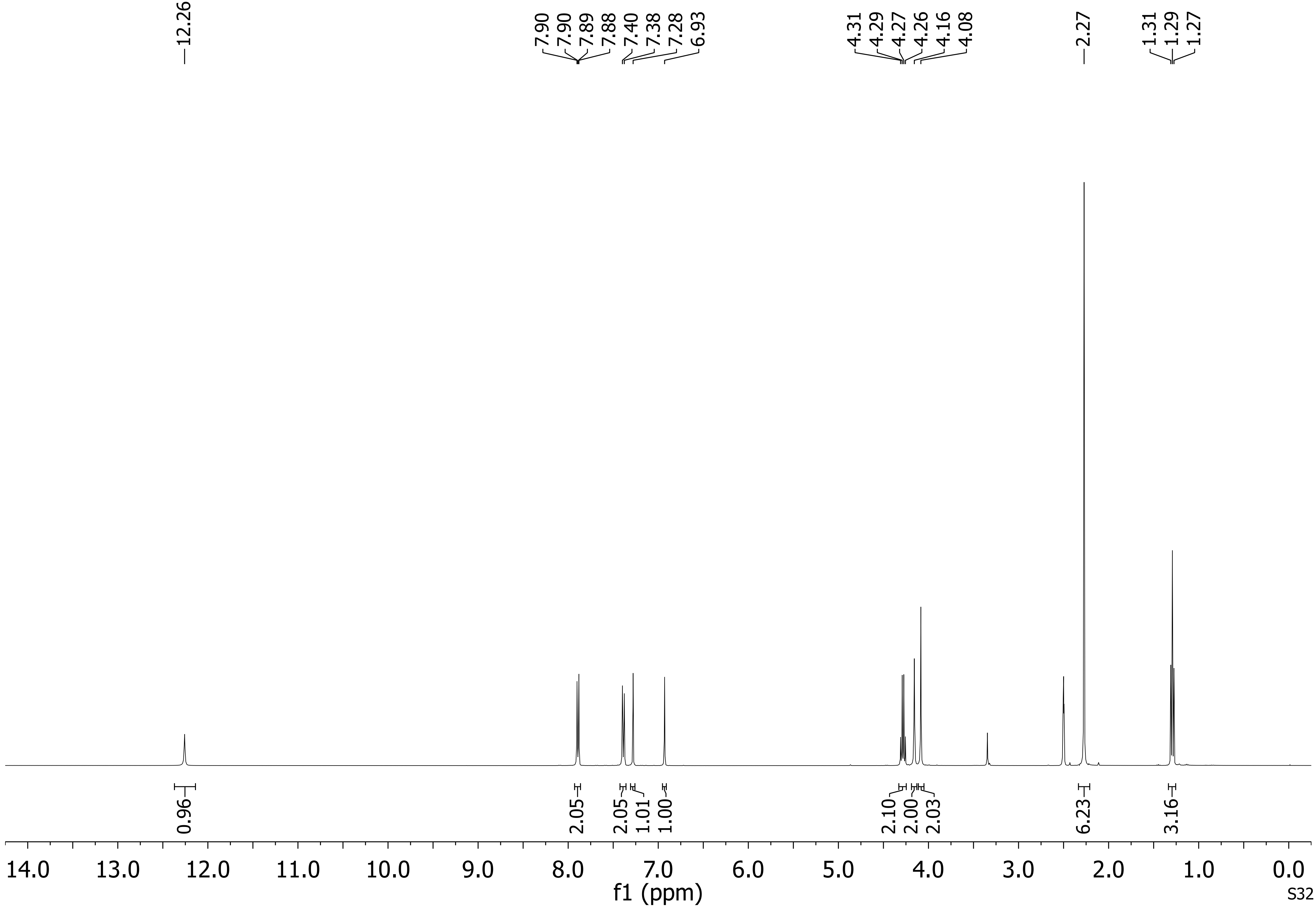

^{13}C NMR (151 MHz, DMSO-d_6) for compound **30**

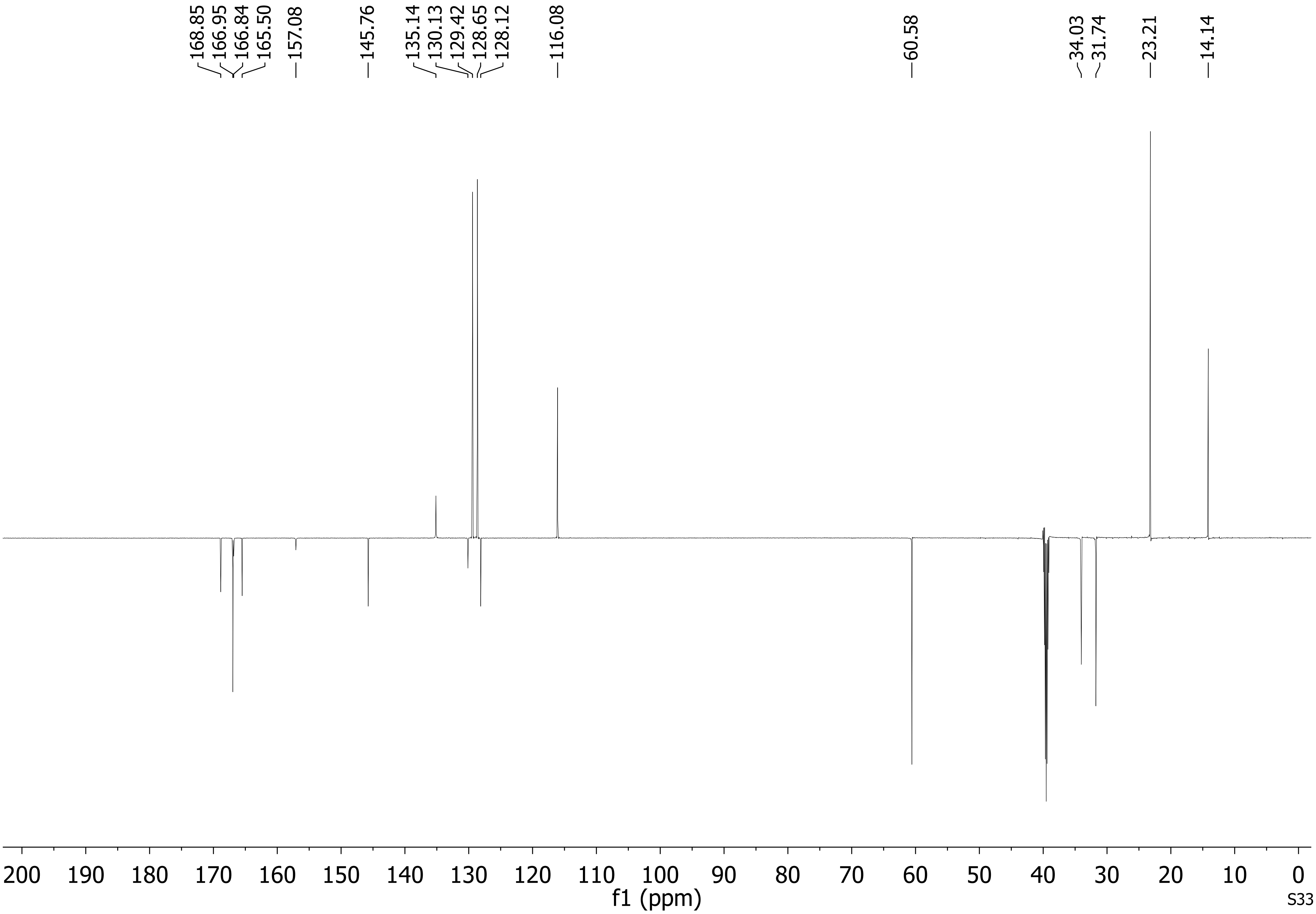

^{1}H NMR (400 MHz, DMSO-d_6) for compound **31**

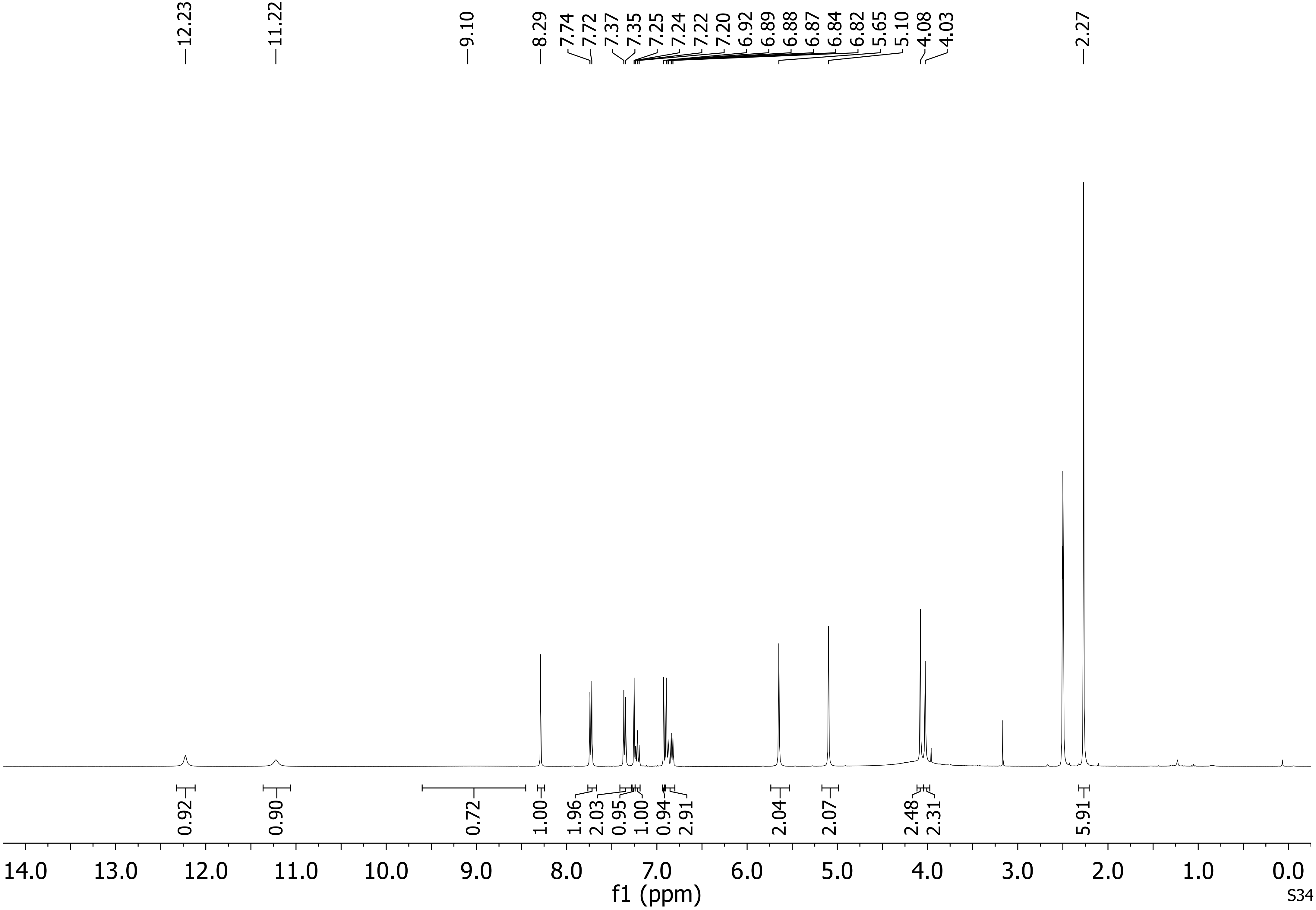

^{13}C NMR (151 MHz, DMSO-d_6) for compound **31**

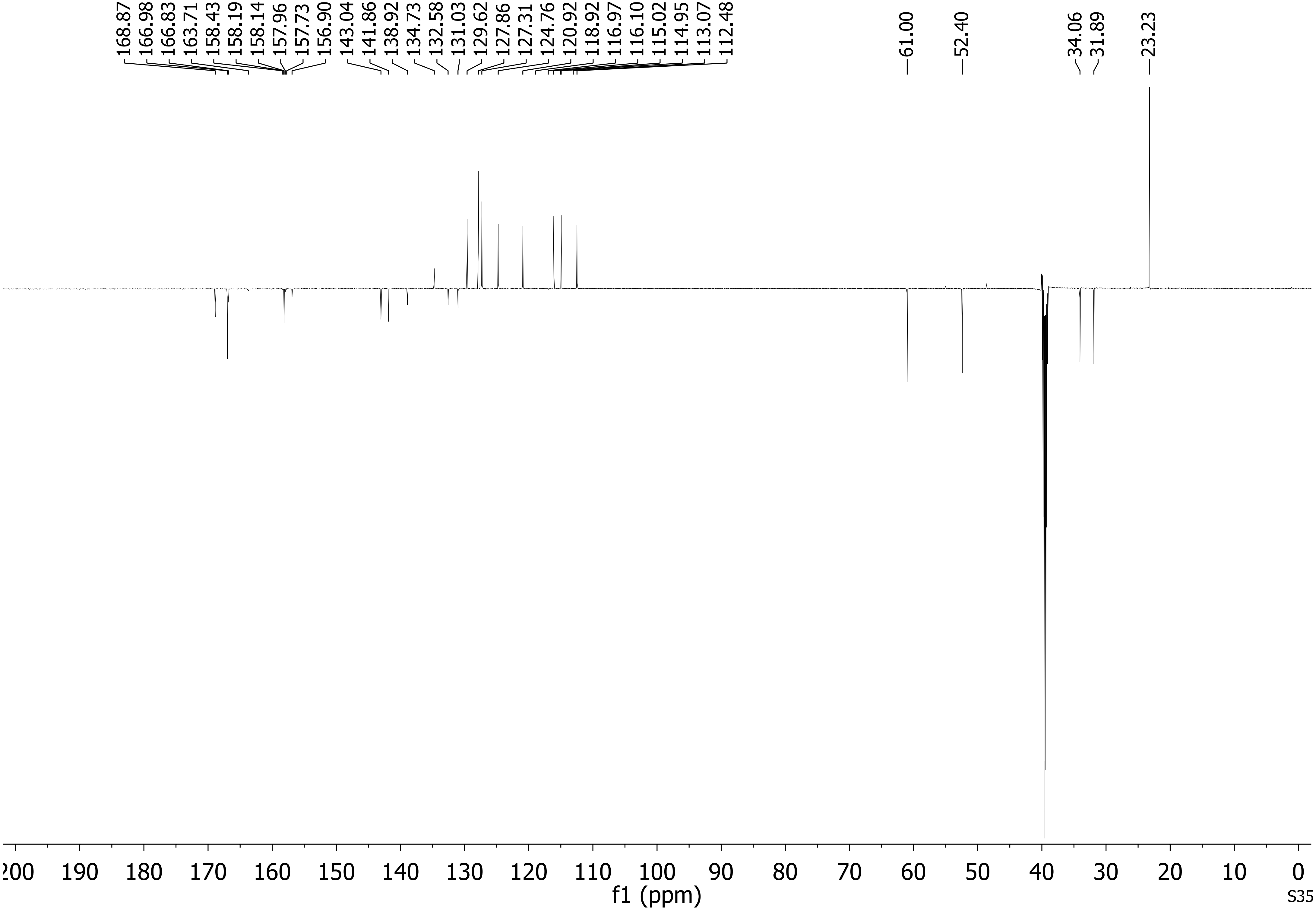

^{19}F NMR (377 MHz, DMSO-d_6) for compound **31**

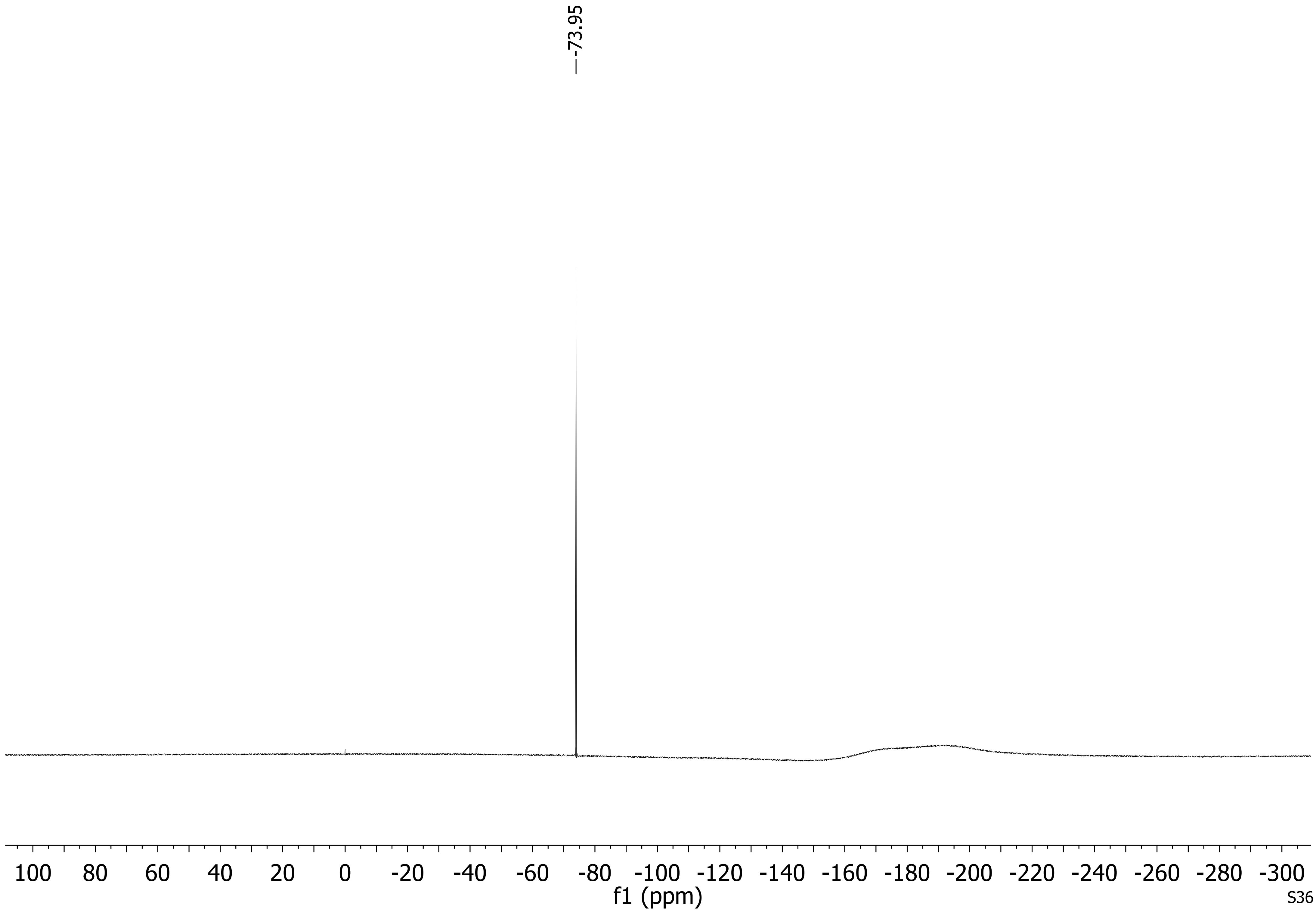

^{1}H NMR (400 MHz, DMSO-d_6) for compound **32**

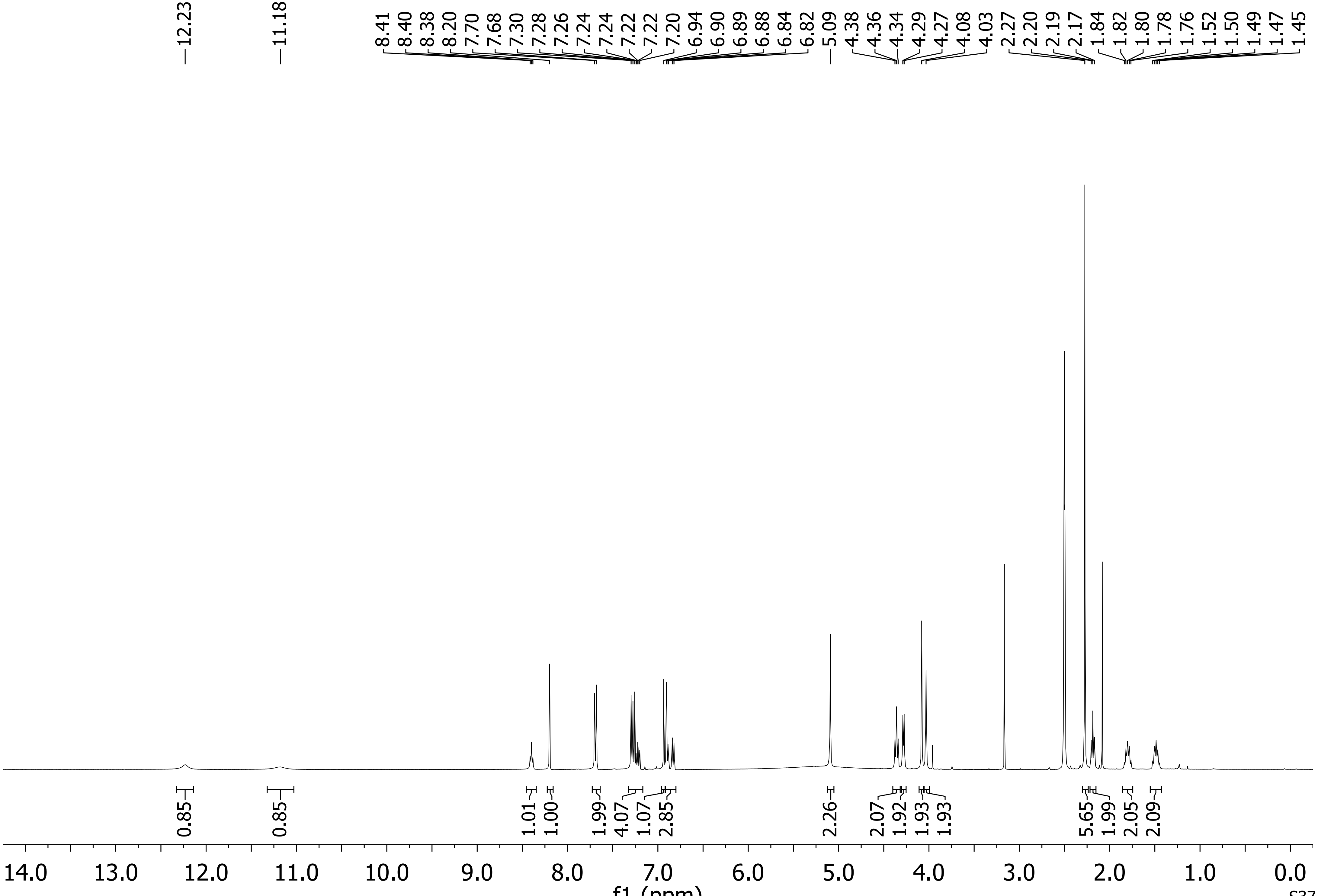

^{13}C NMR (101 MHz, DMSO-d_6) for compound **32** (full view)

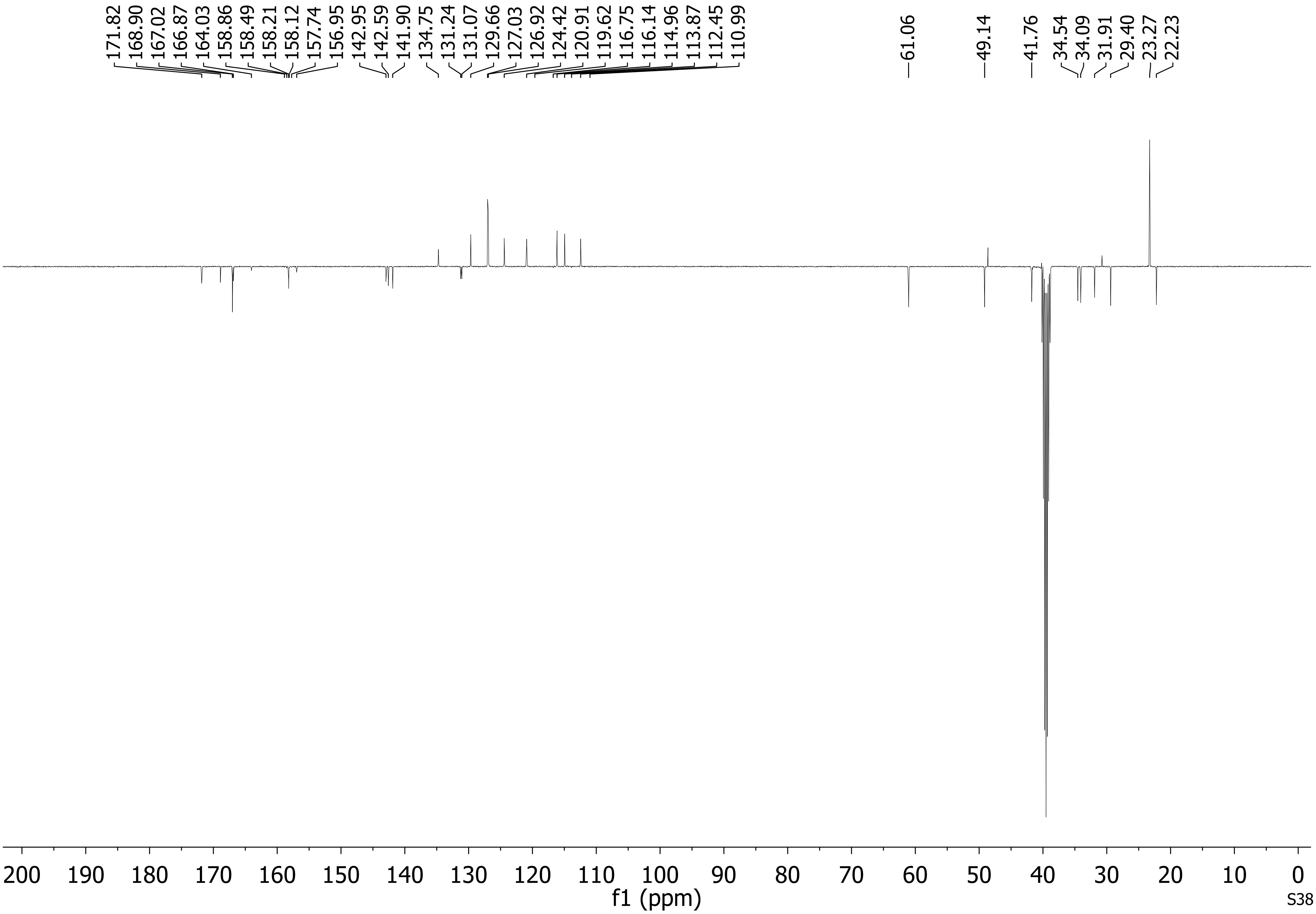

^{13}C NMR (101 MHz, DMSO-d_6) for compound **32** (zoomed-in view)

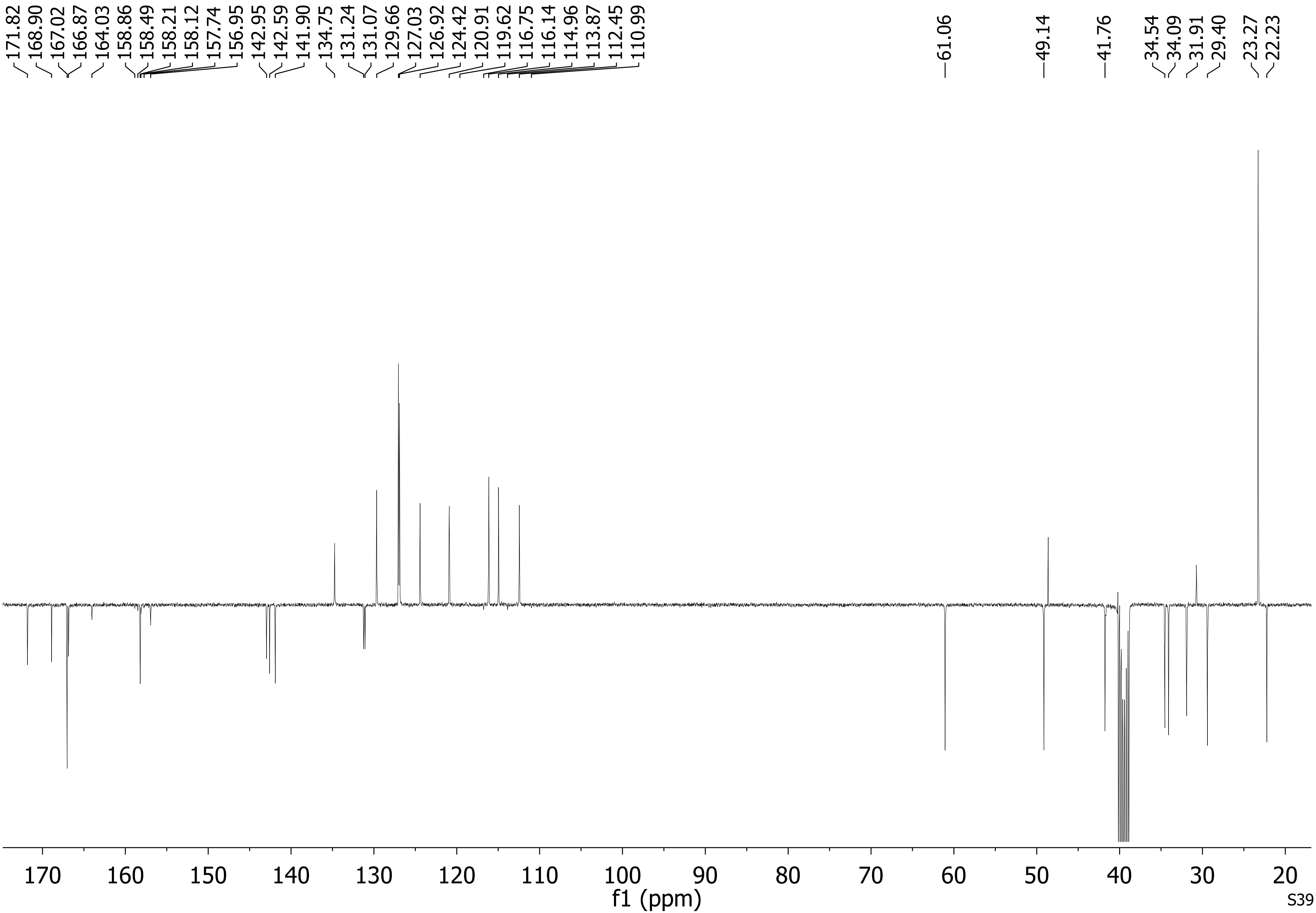

^{19}F NMR (565 MHz, DMSO-d_6) for compound **32**

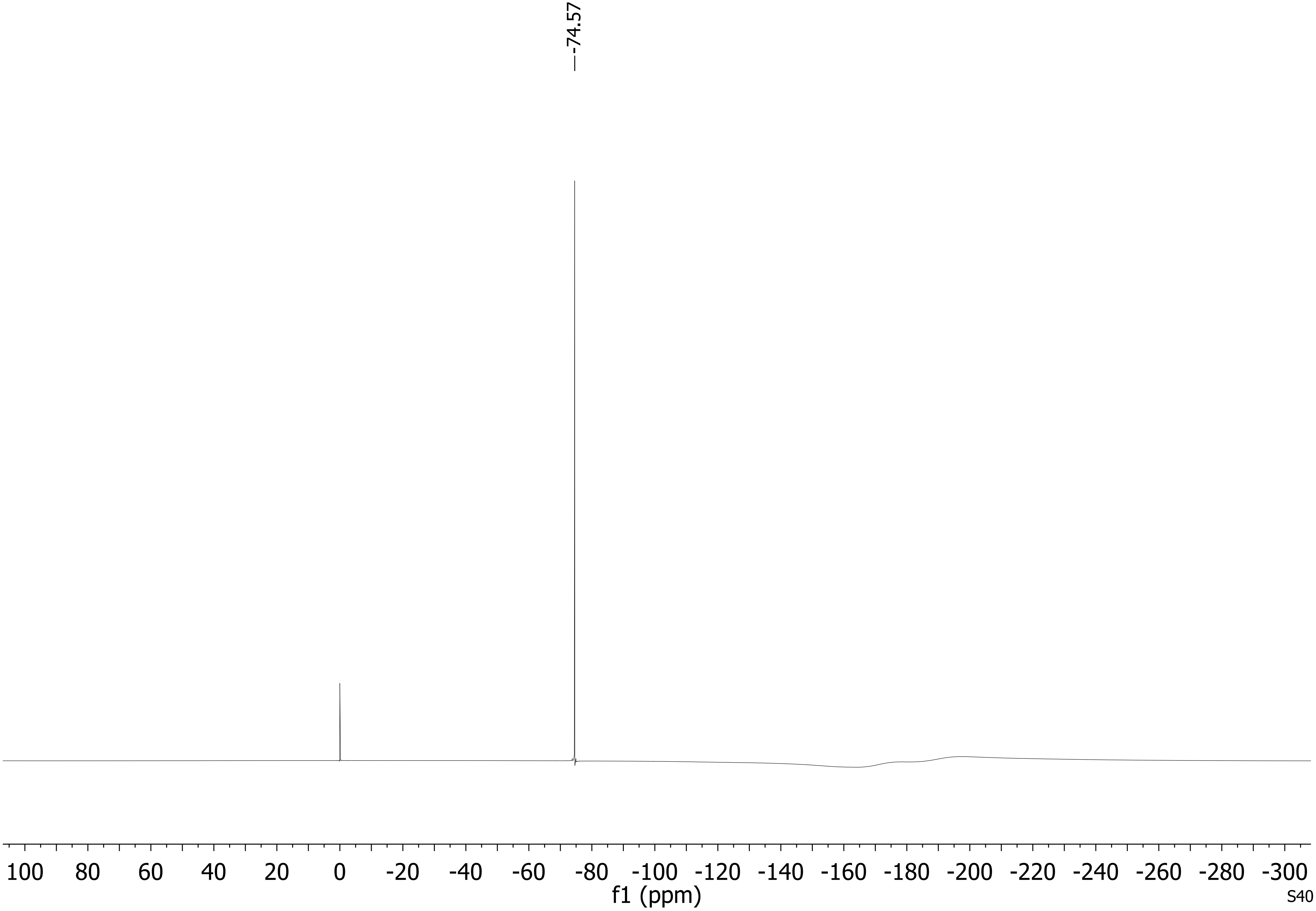

^{1}H NMR (600 MHz, DMSO-d_6) for compound **33**

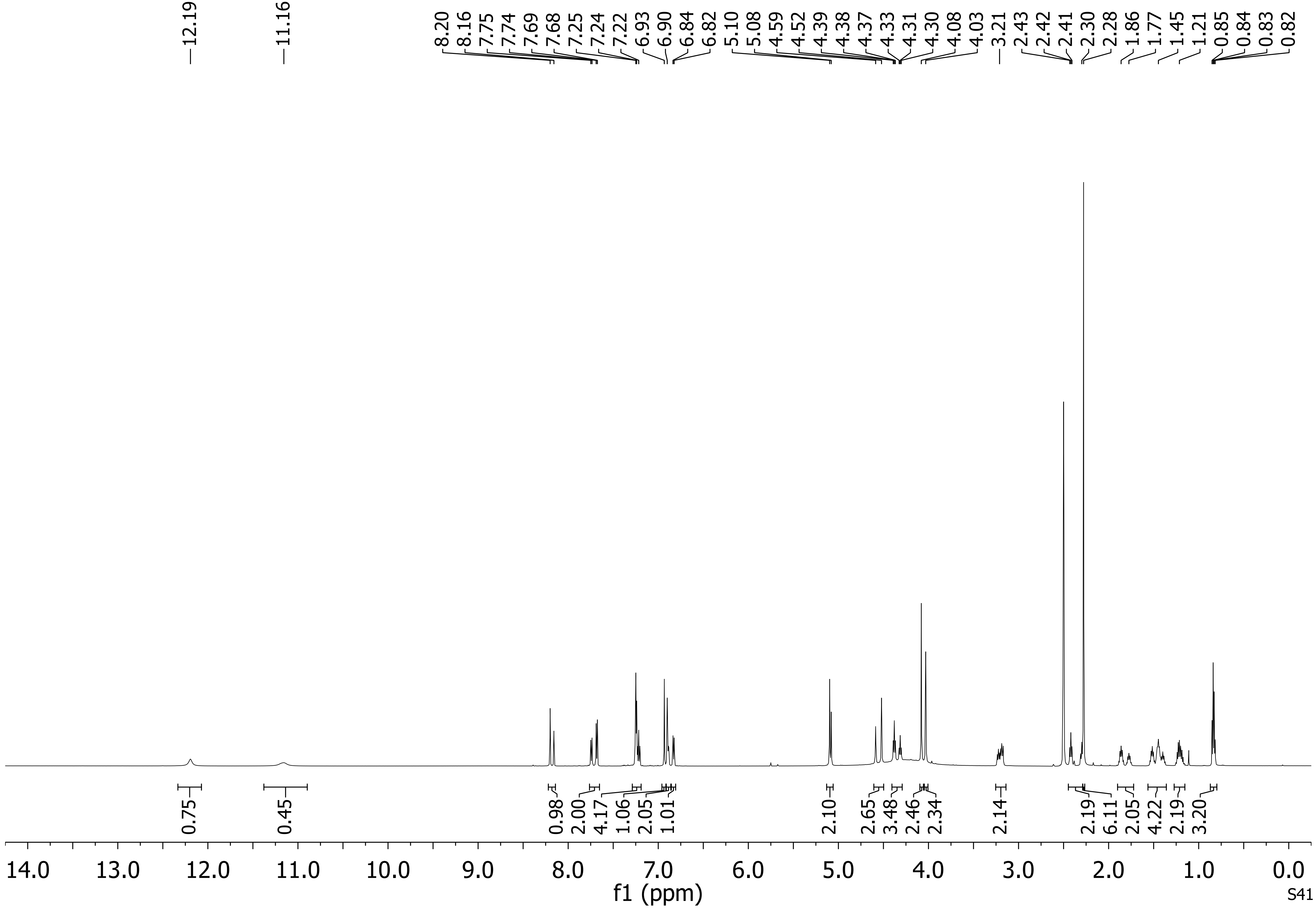

^{13}C NMR (151 MHz, DMSO-d_6) for compound **33**

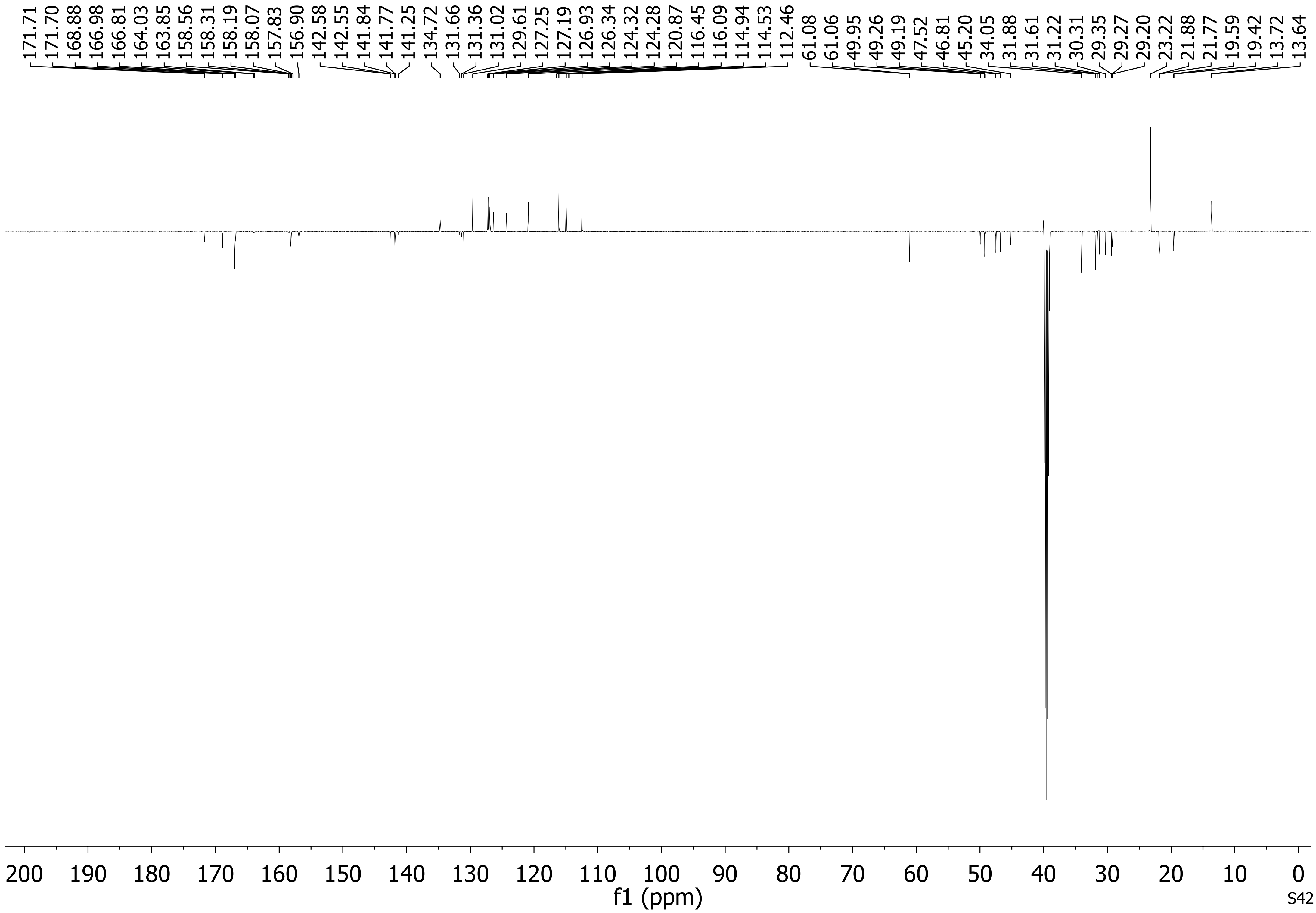

^{13}C NMR (151 MHz, DMSO-d_6) for compound **33** (zoomed-in view 1)

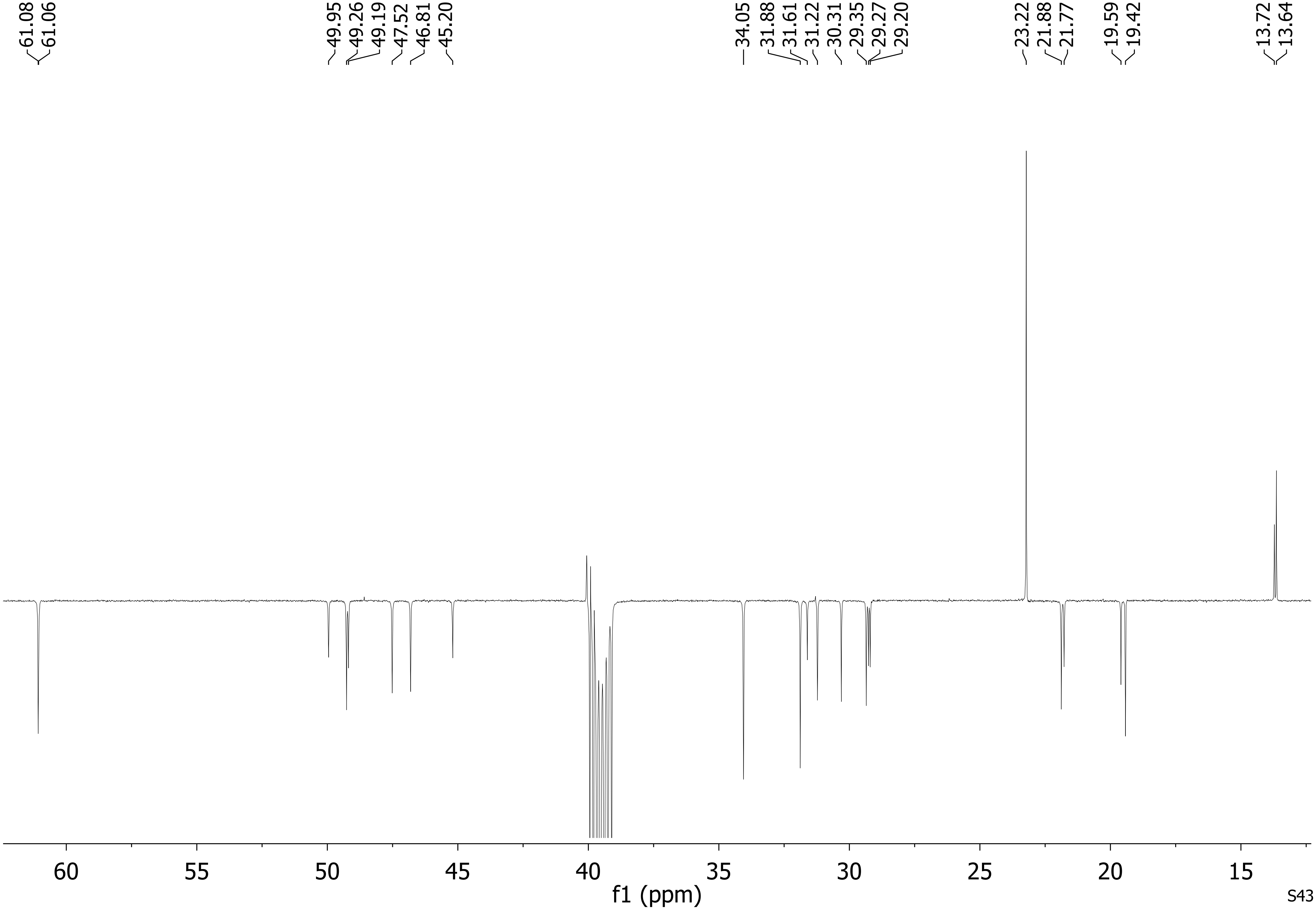

^{13}C NMR (151 MHz, DMSO-d_6) for compound **33** (zoomed-in view 2)

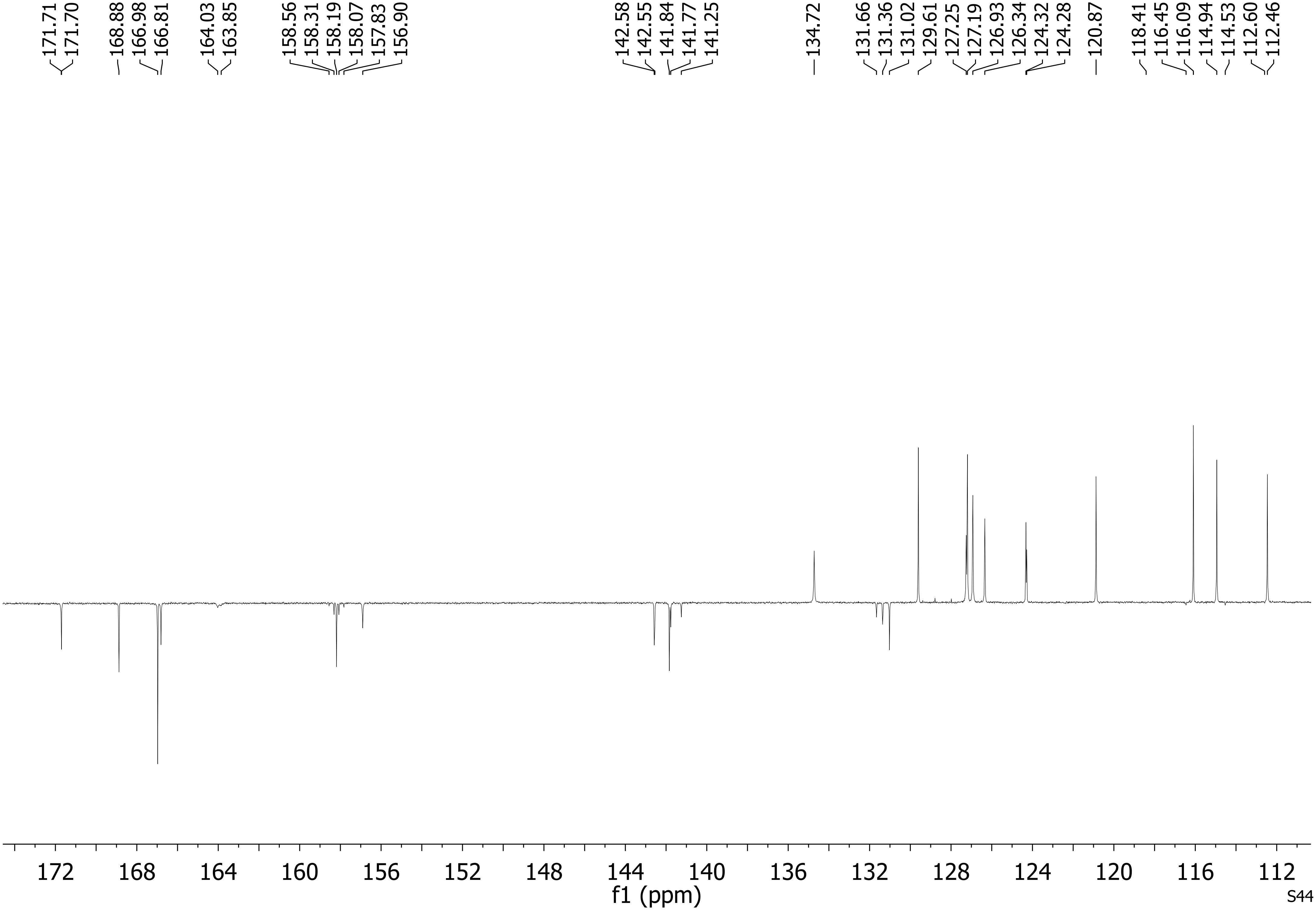

^{19}F NMR (377 MHz, DMSO-d_6) for compound **33**

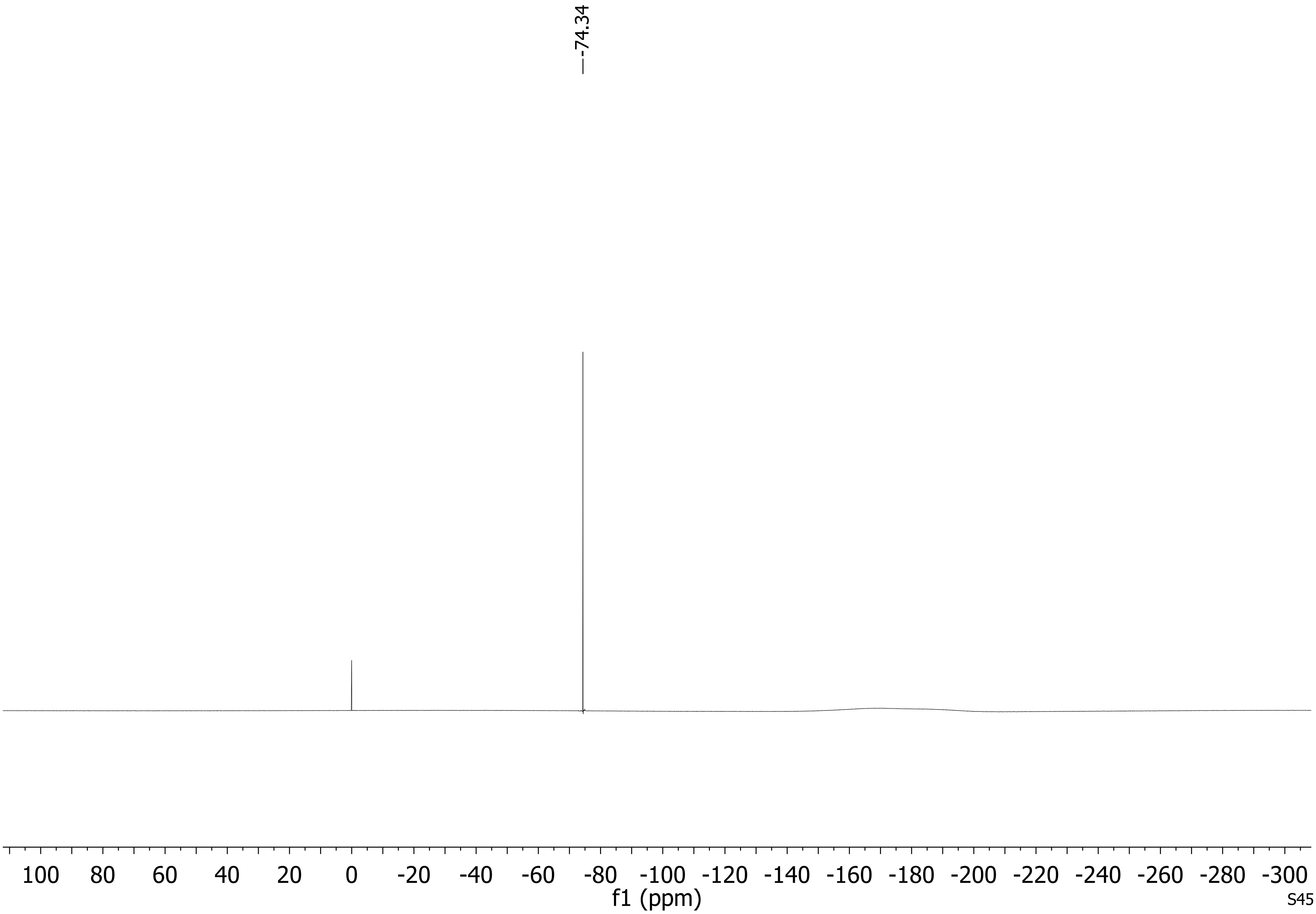

^1H NMR (400 MHz, DMSO-d_6) for compound **34**

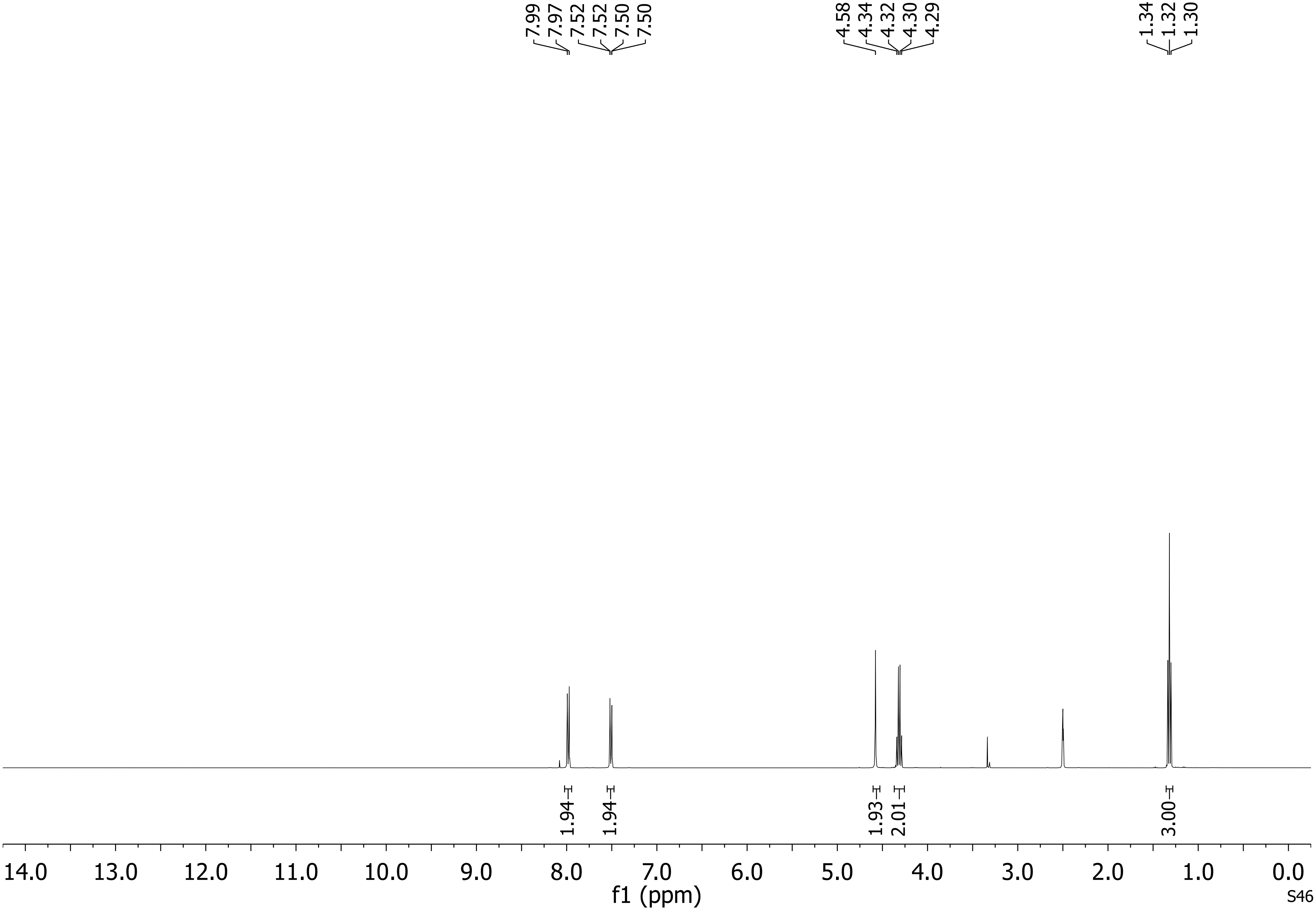

^{13}C NMR (101 MHz, DMSO-d_6) for compound **34**

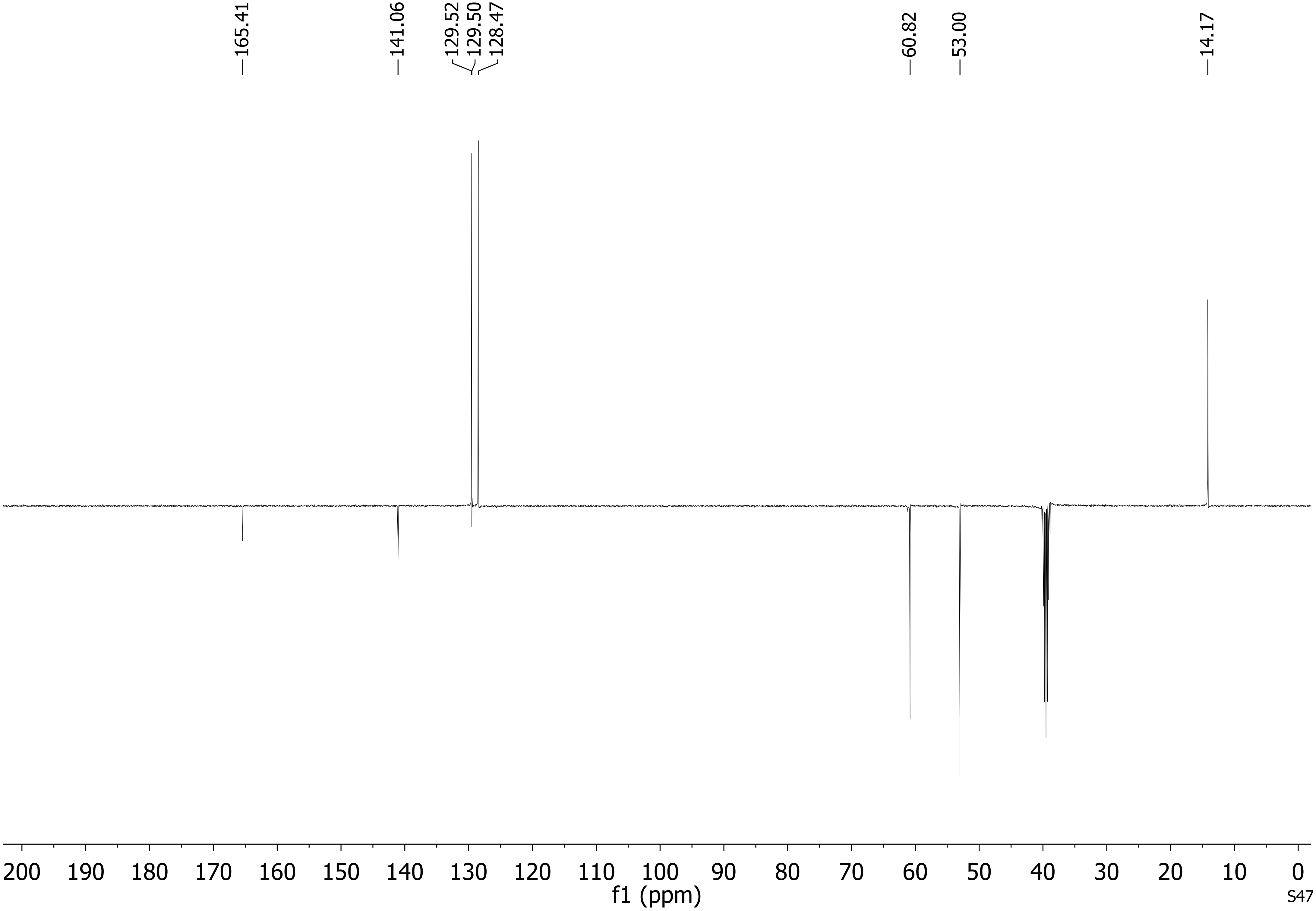

^{1}H NMR (400 MHz, DMSO-d_6) for compound **36**

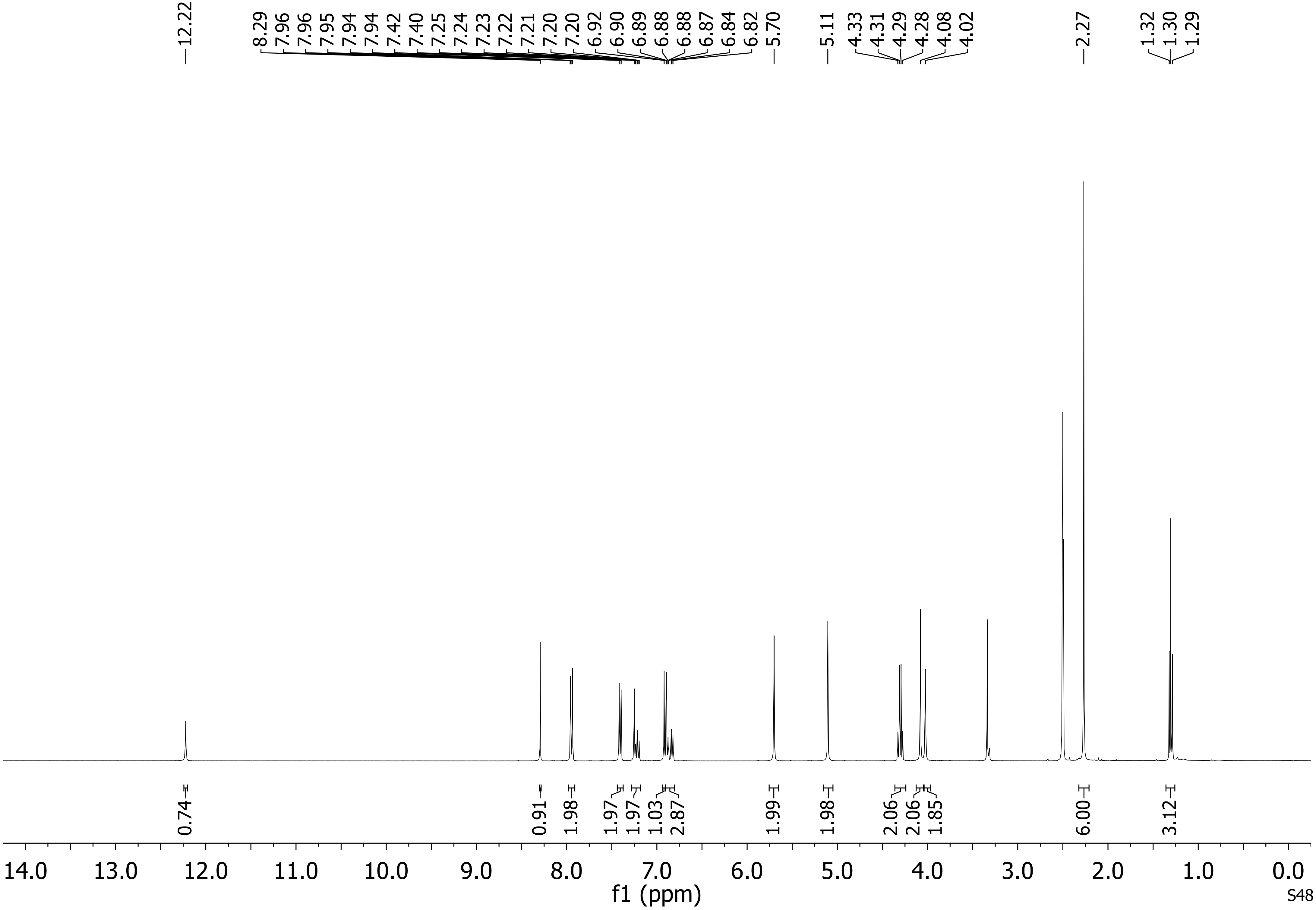

^{13}C NMR (101 MHz, DMSO-d_6) for compound **36**

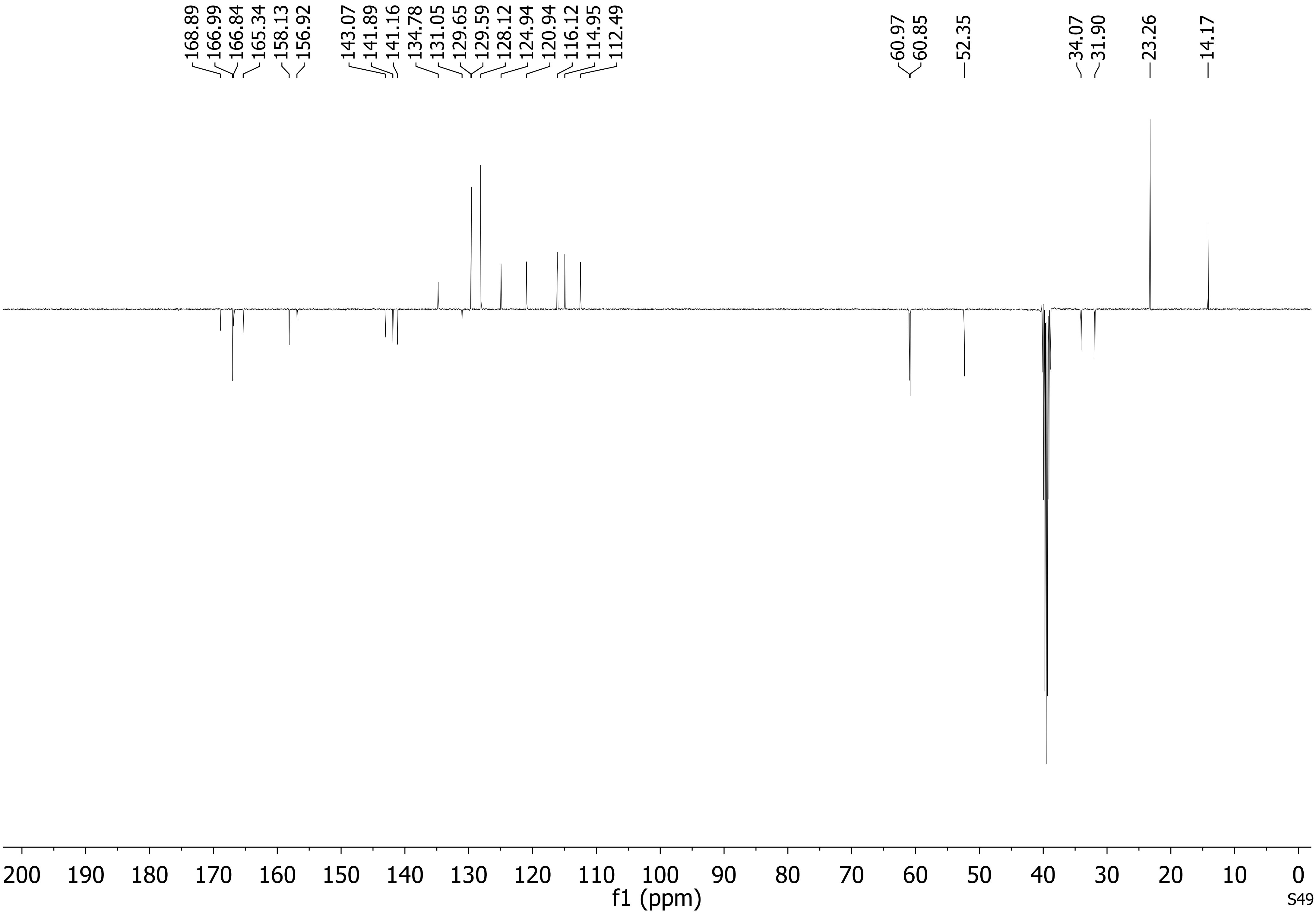

^{1}H NMR (400 MHz, DMSO-d_6) for compound **37**

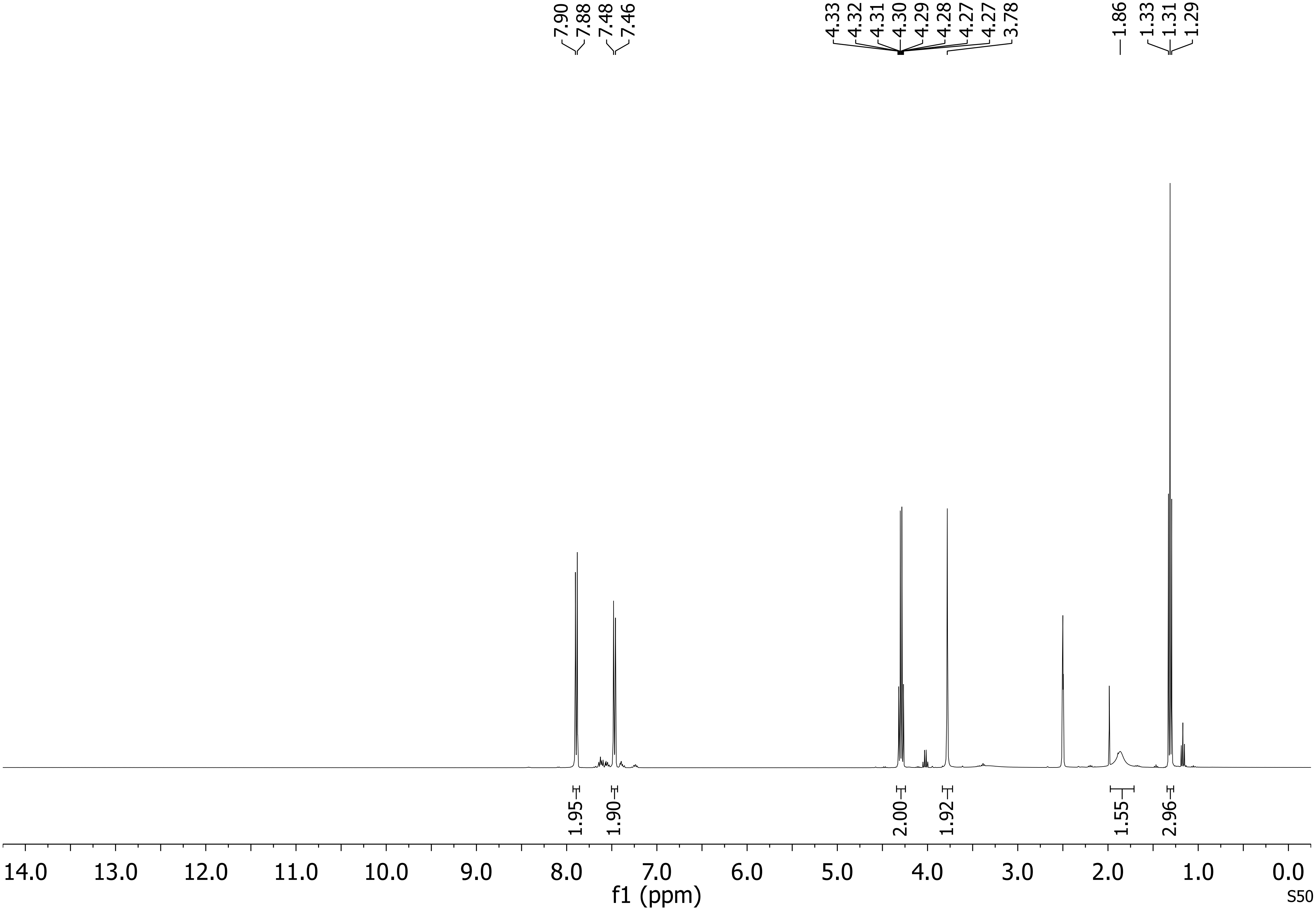

^{13}C NMR (101 MHz, DMSO-d_6) for compound **37**

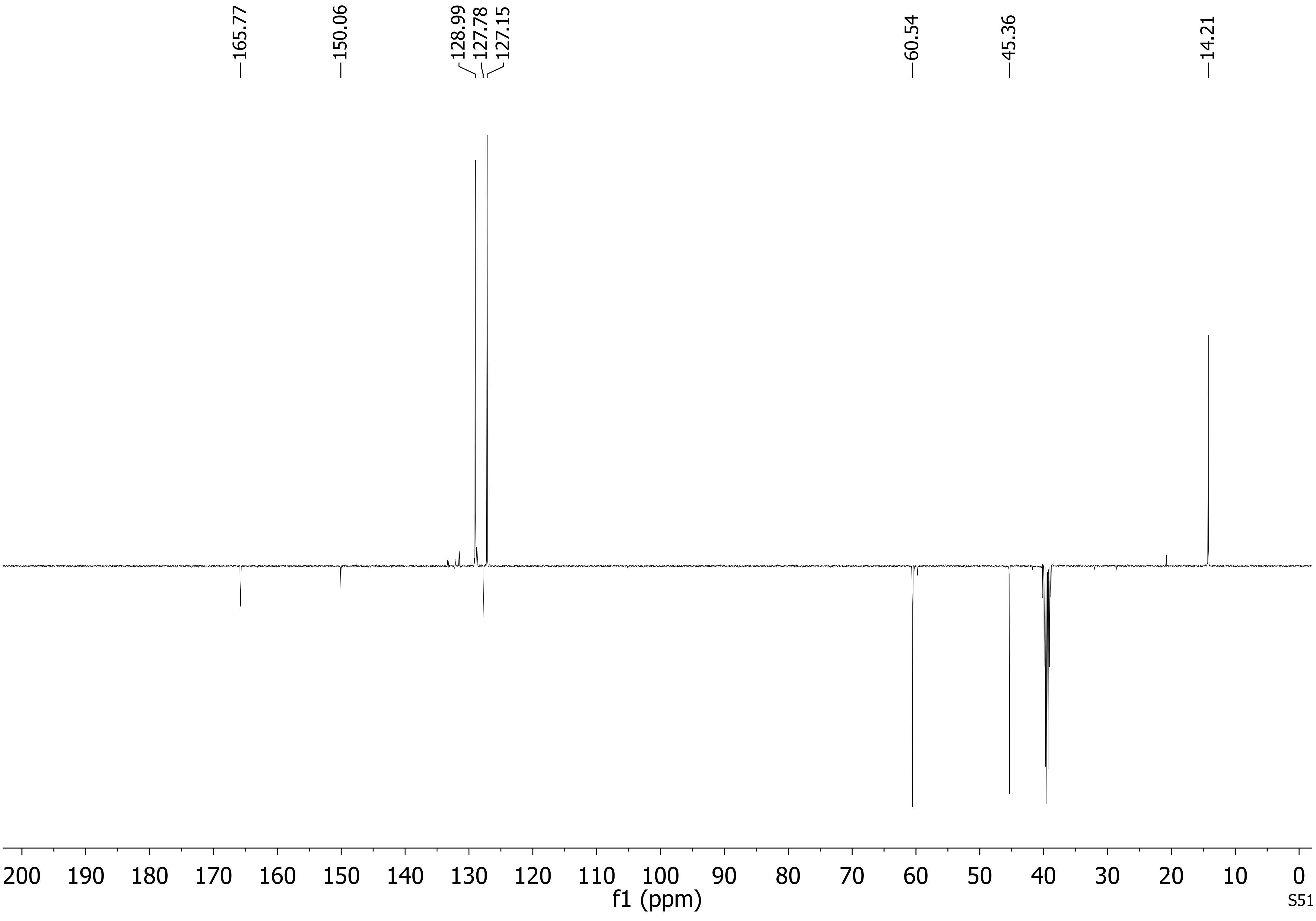

^{1}H NMR (400 MHz, DMSO-d_6) for compound **38**

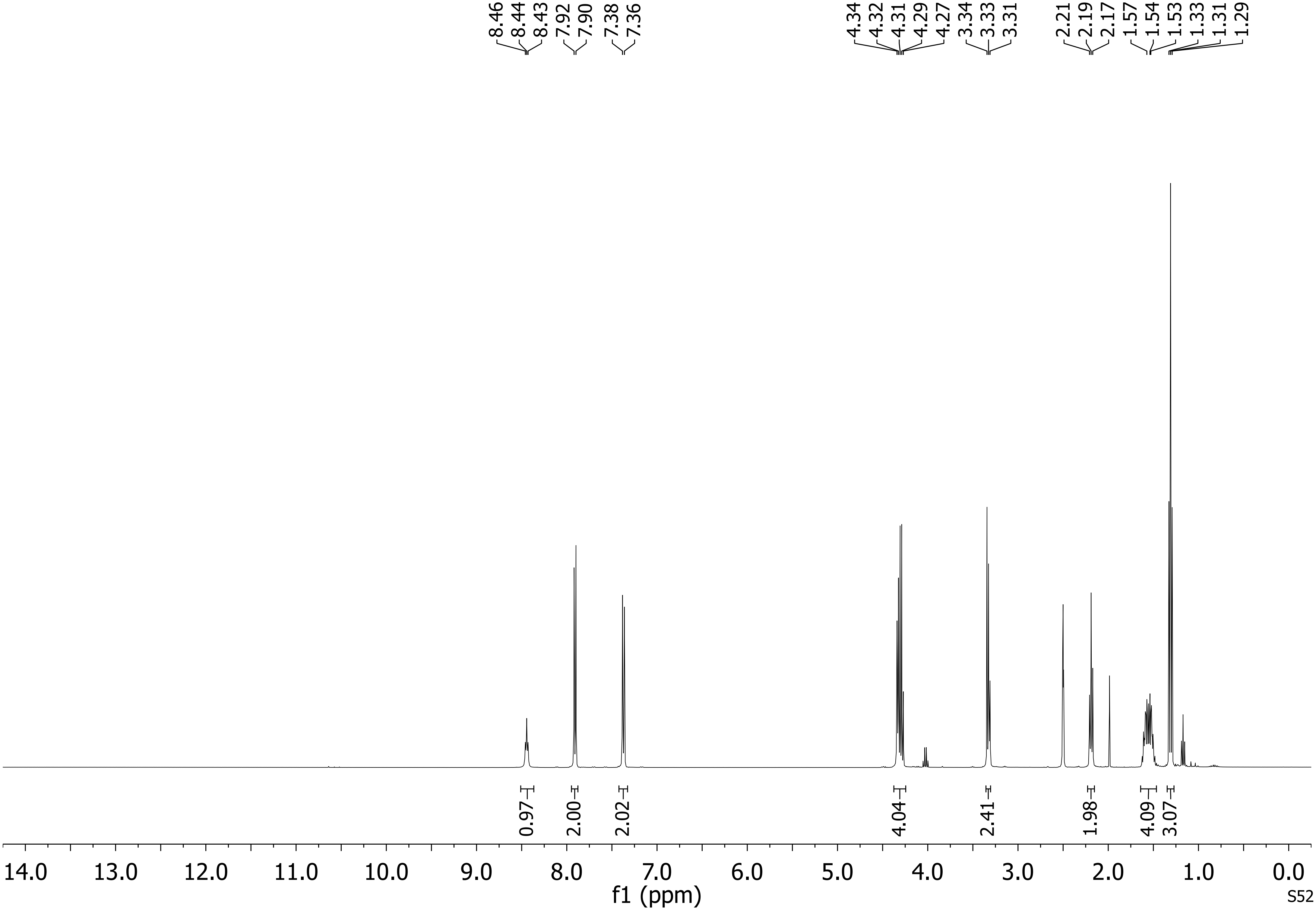

^{13}C NMR (101 MHz, DMSO-d_6) for compound **38**

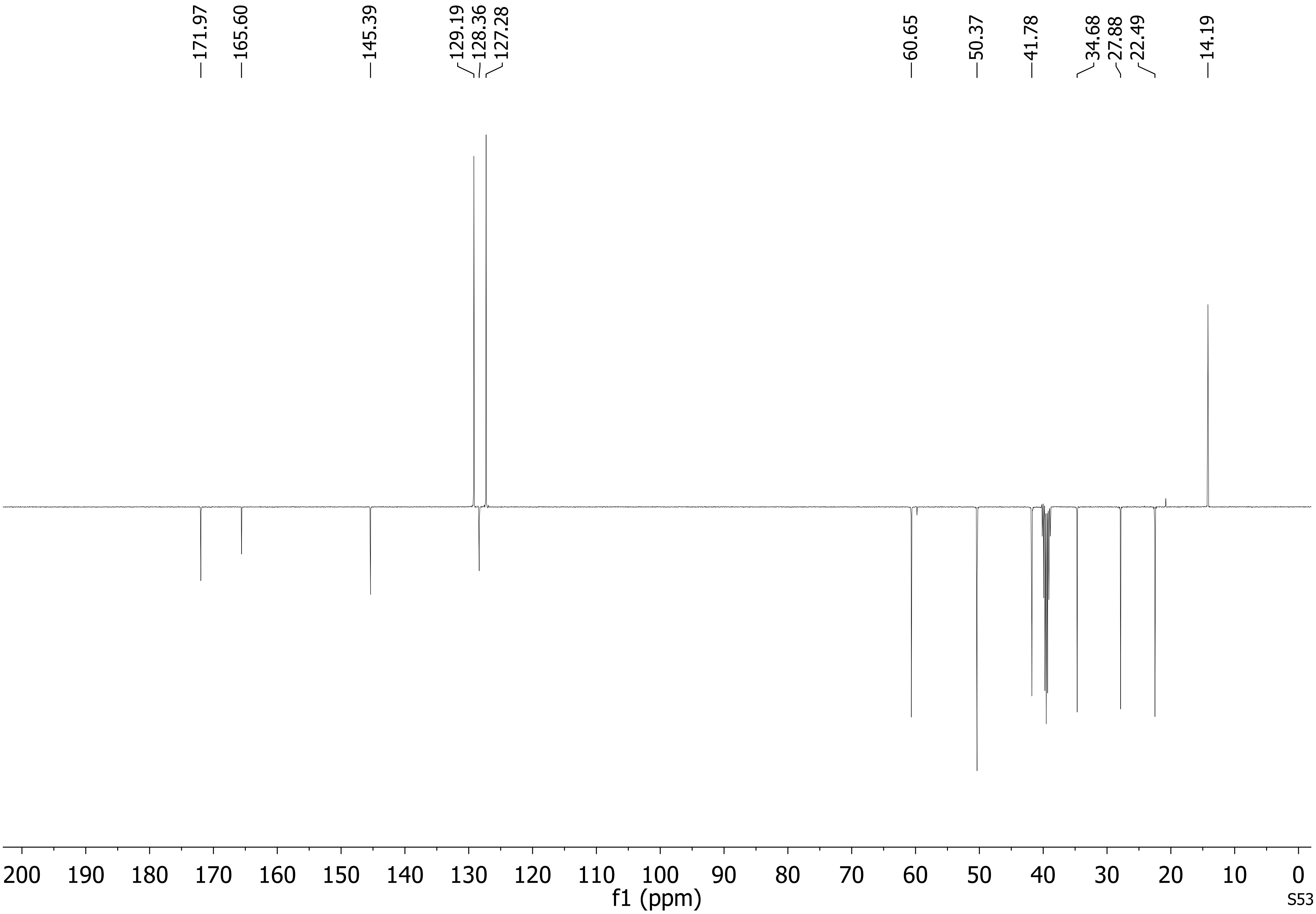

^{1}H NMR (400 MHz, DMSO-d_6) for compound **39**

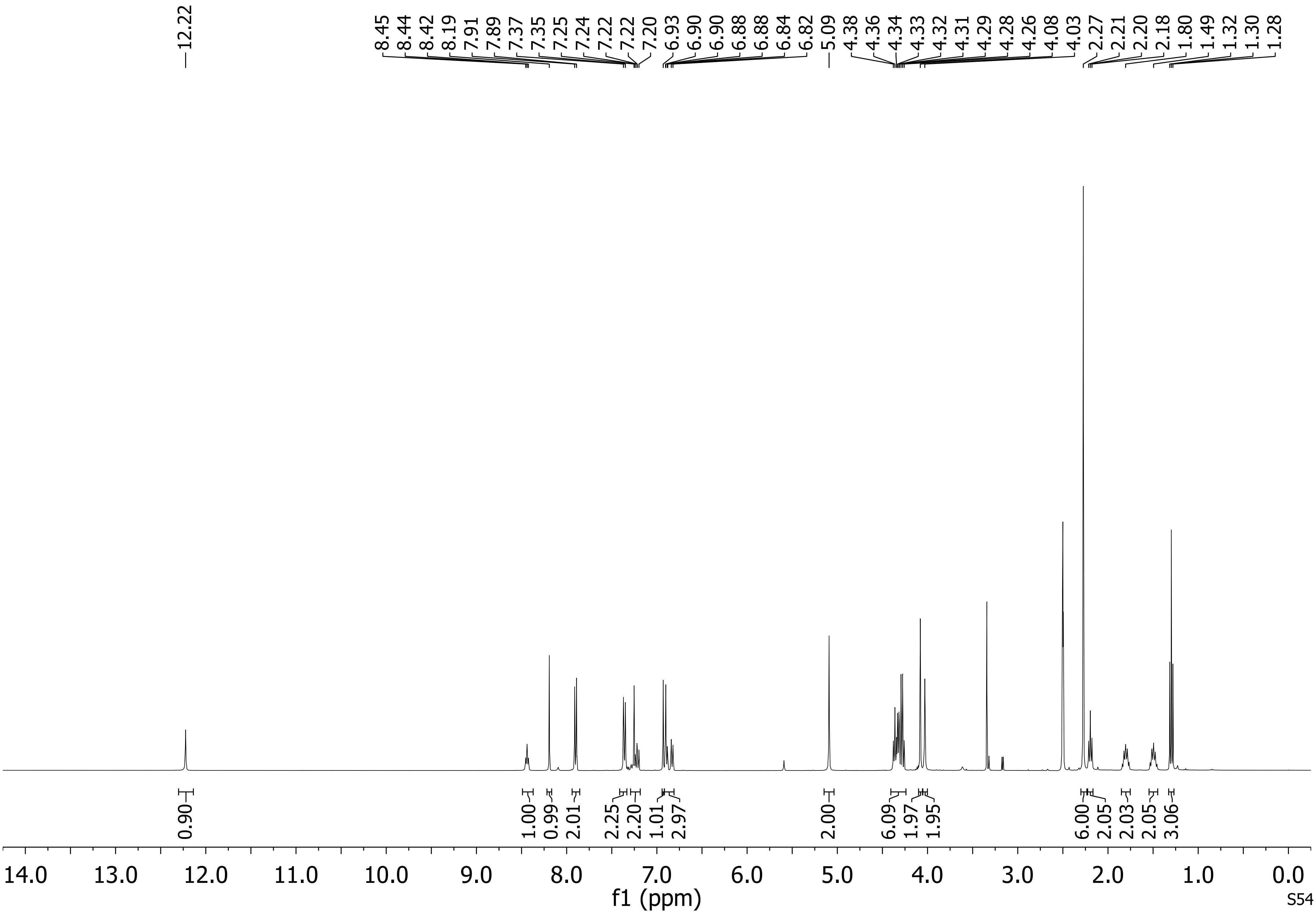

^{13}C NMR (101 MHz, DMSO-d_6) for compound **39**

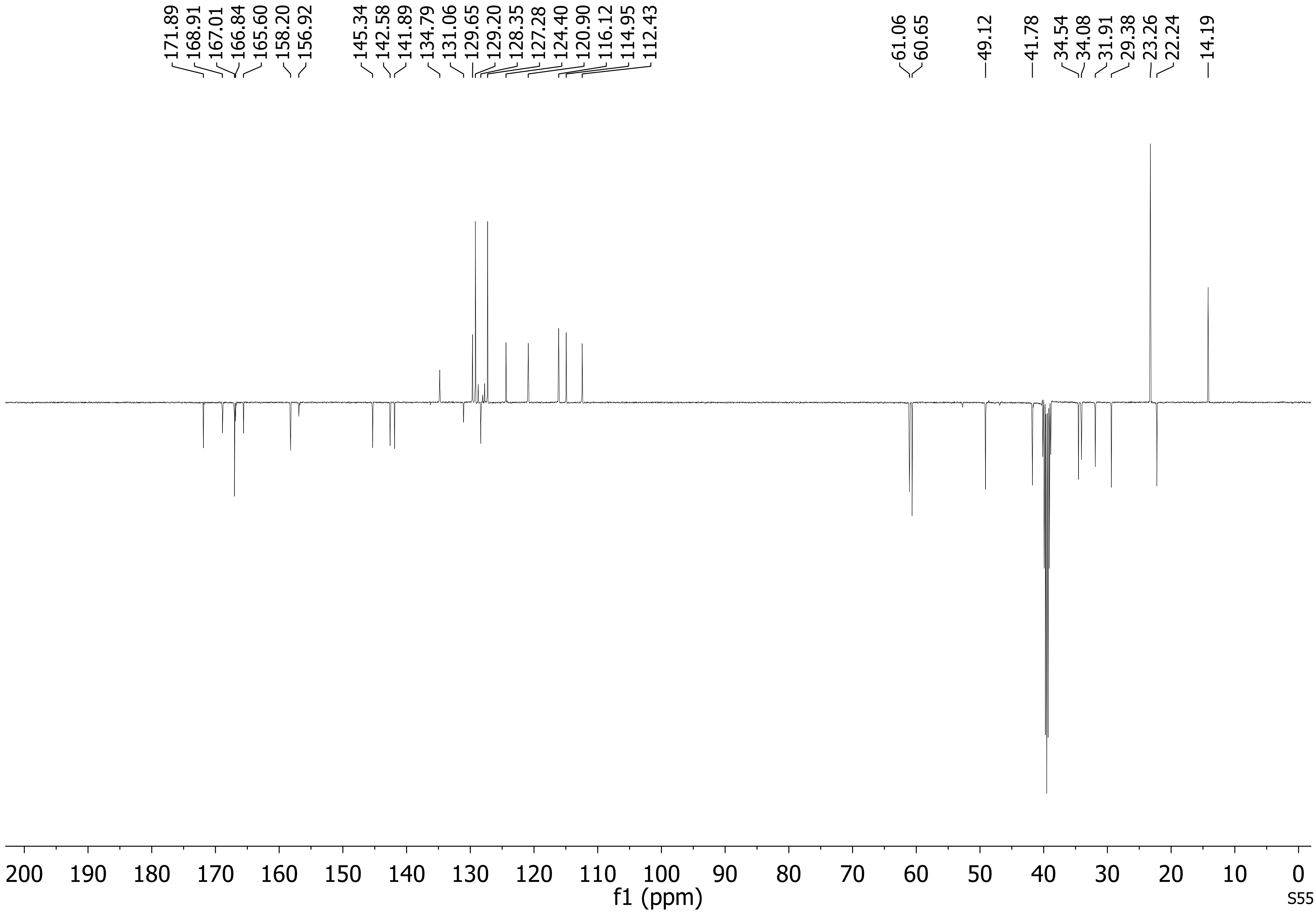

^{1}H NMR (400 MHz, DMSO-d_6) for compound **40**

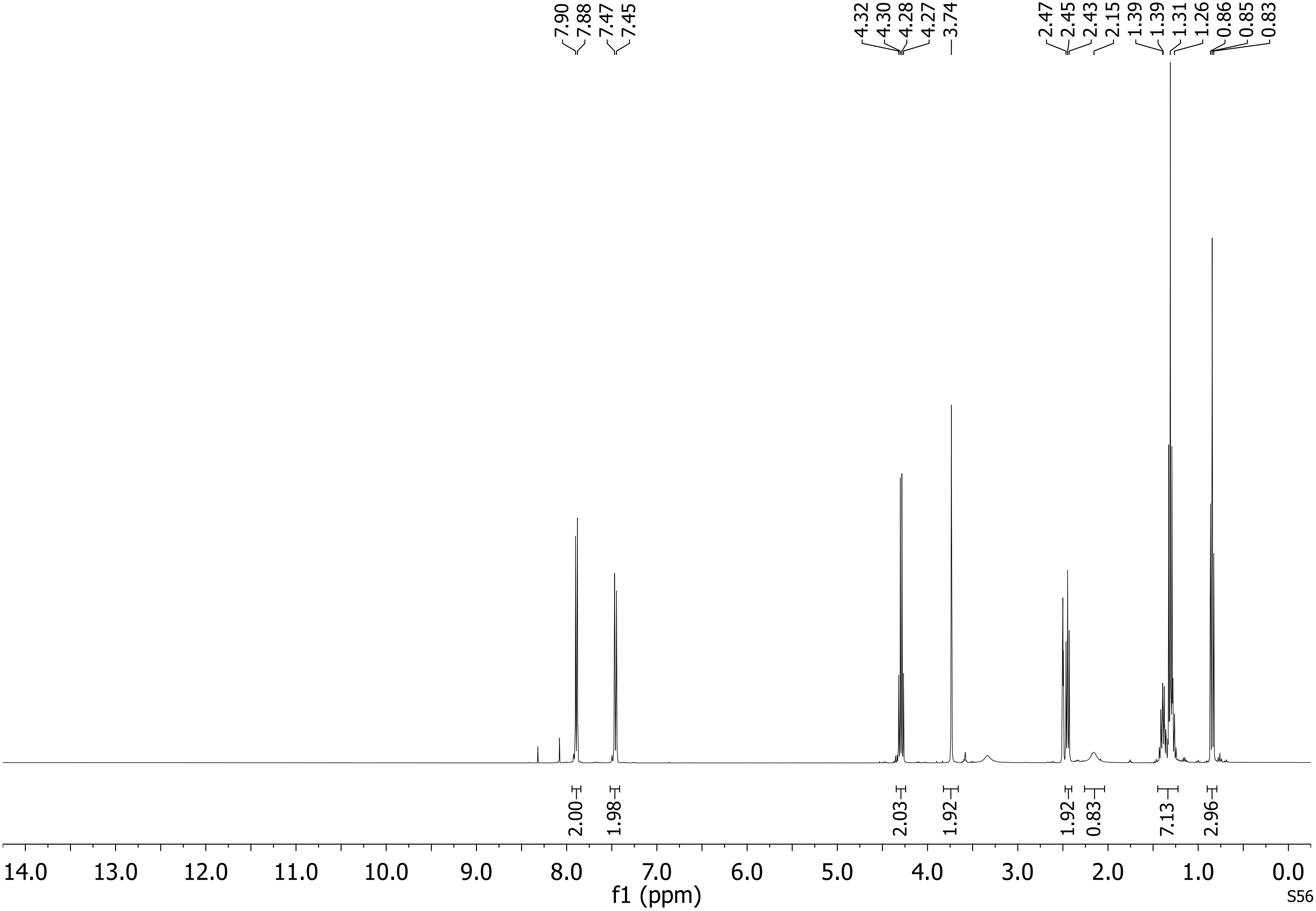

^{13}C NMR (151 MHz, DMSO-d_6) for compound **40**

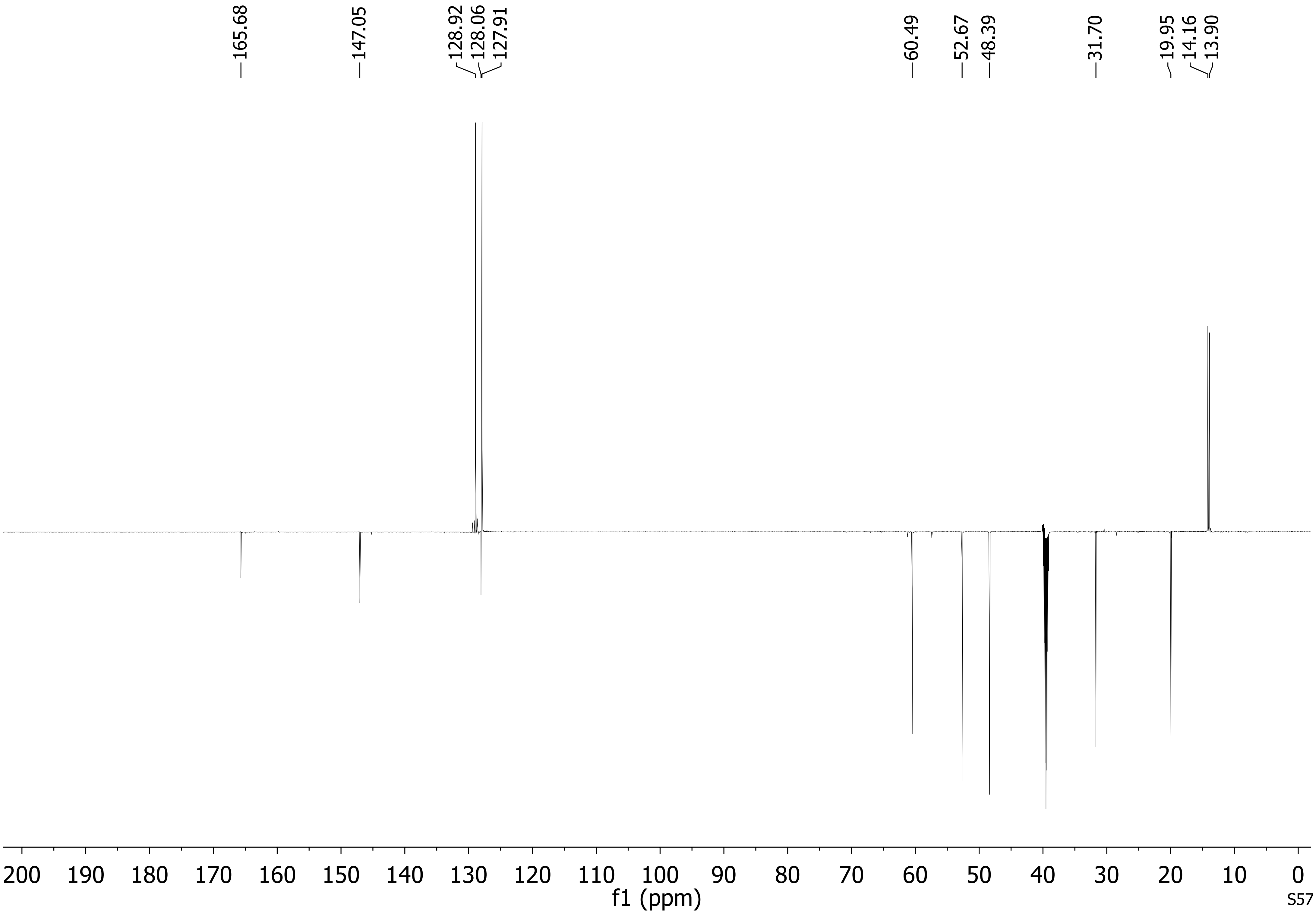

^{1}H NMR (400 MHz, DMSO-d_6) for compound **42**

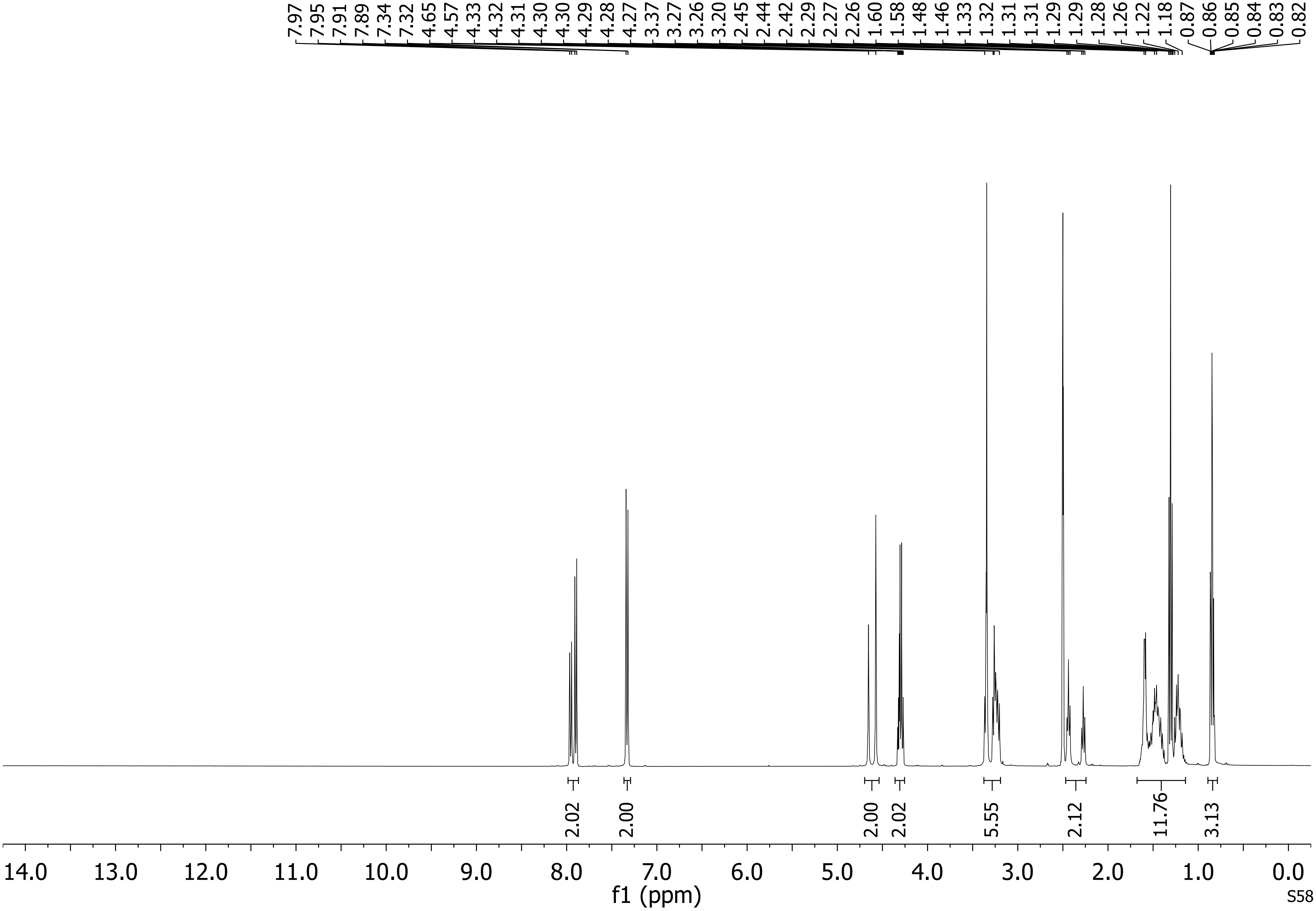

^{13}C NMR (101 MHz, DMSO-d_6) for compound **42**

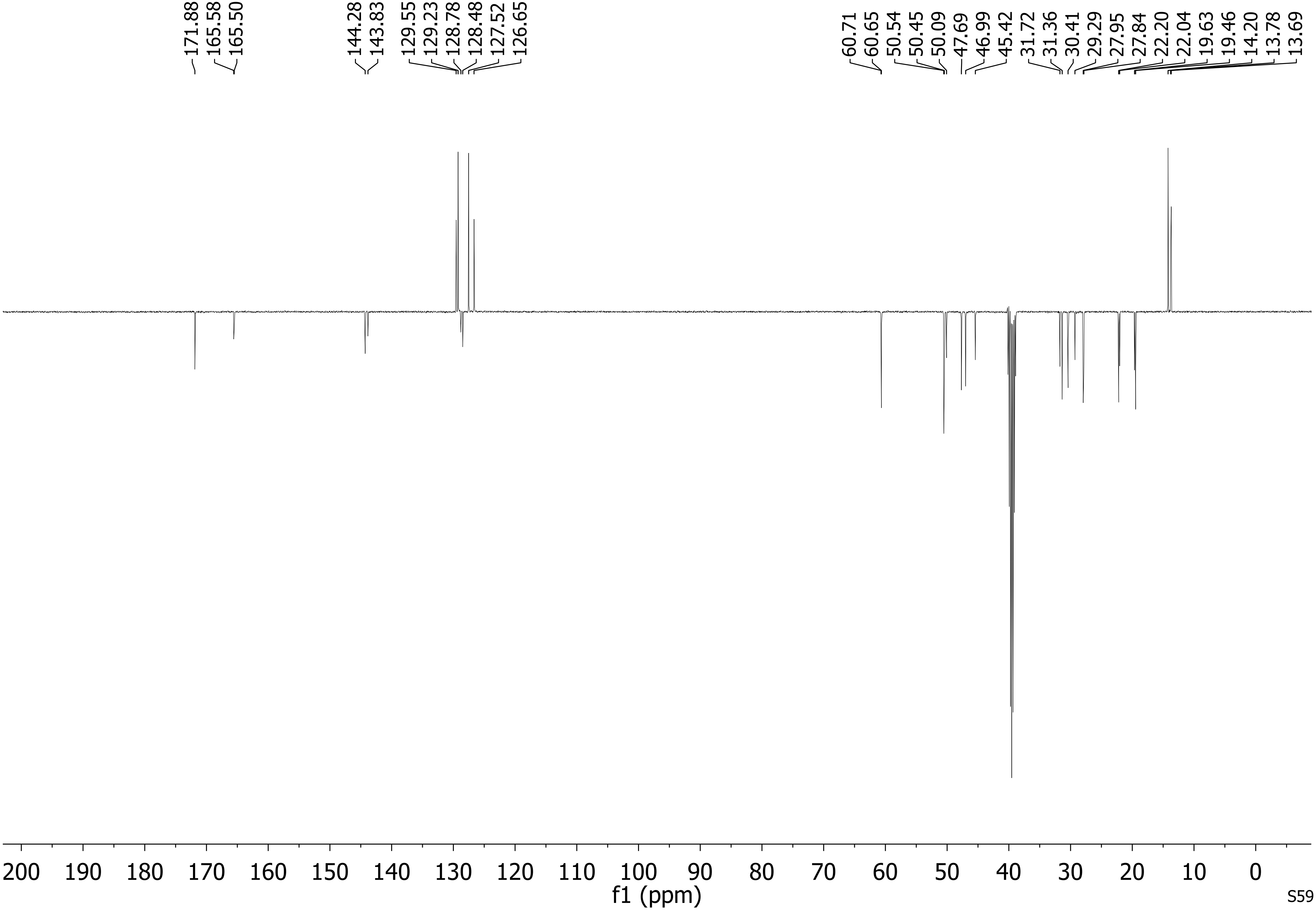

^{1}H NMR (400 MHz, DMSO-d_6) for compound **43**

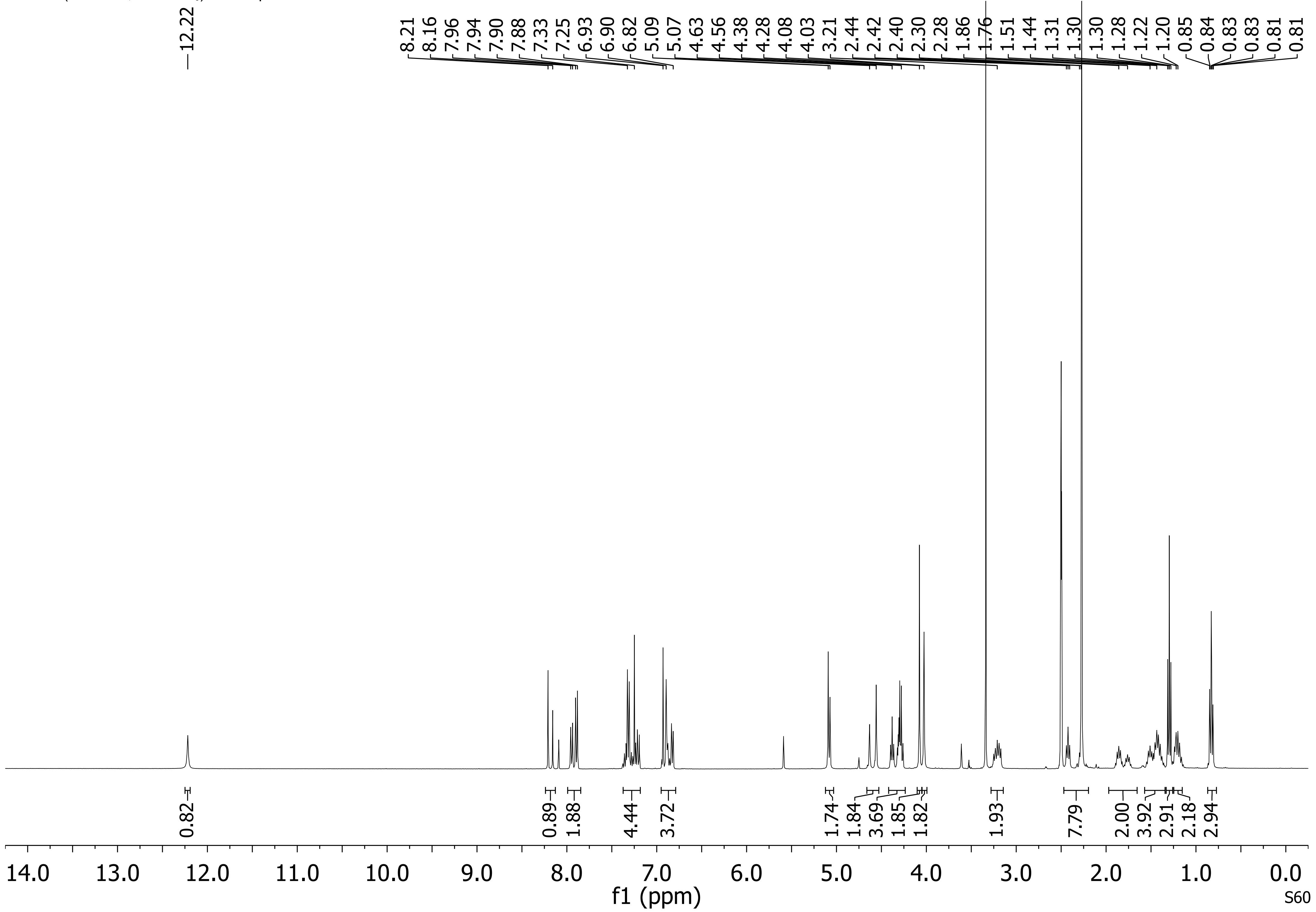

^{13}C NMR (151 MHz, DMSO-d_6) for compound **43** (full view)

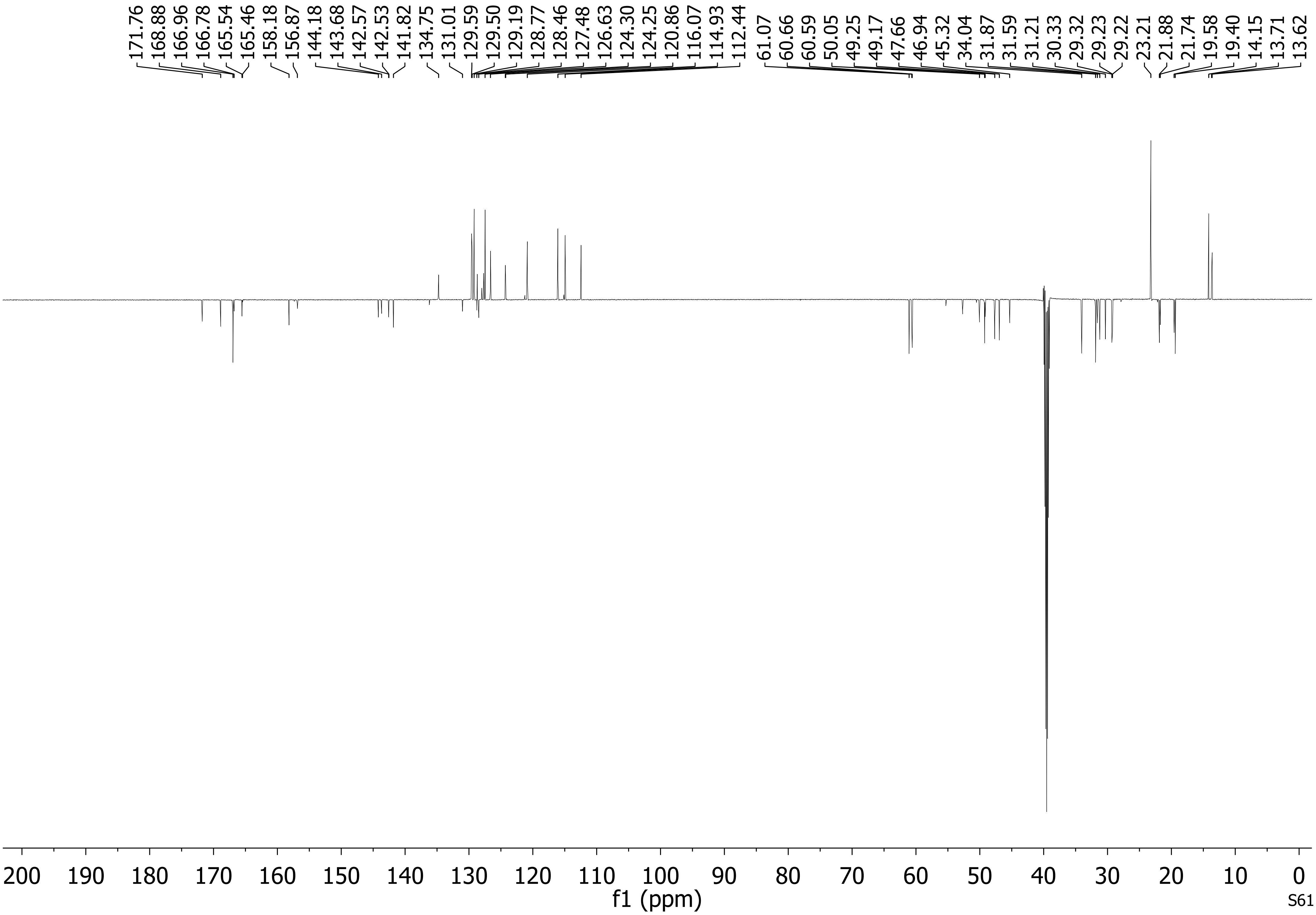

^{13}C NMR (151 MHz, DMSO-d_6) for compound **43** (zoomed-in view 1)

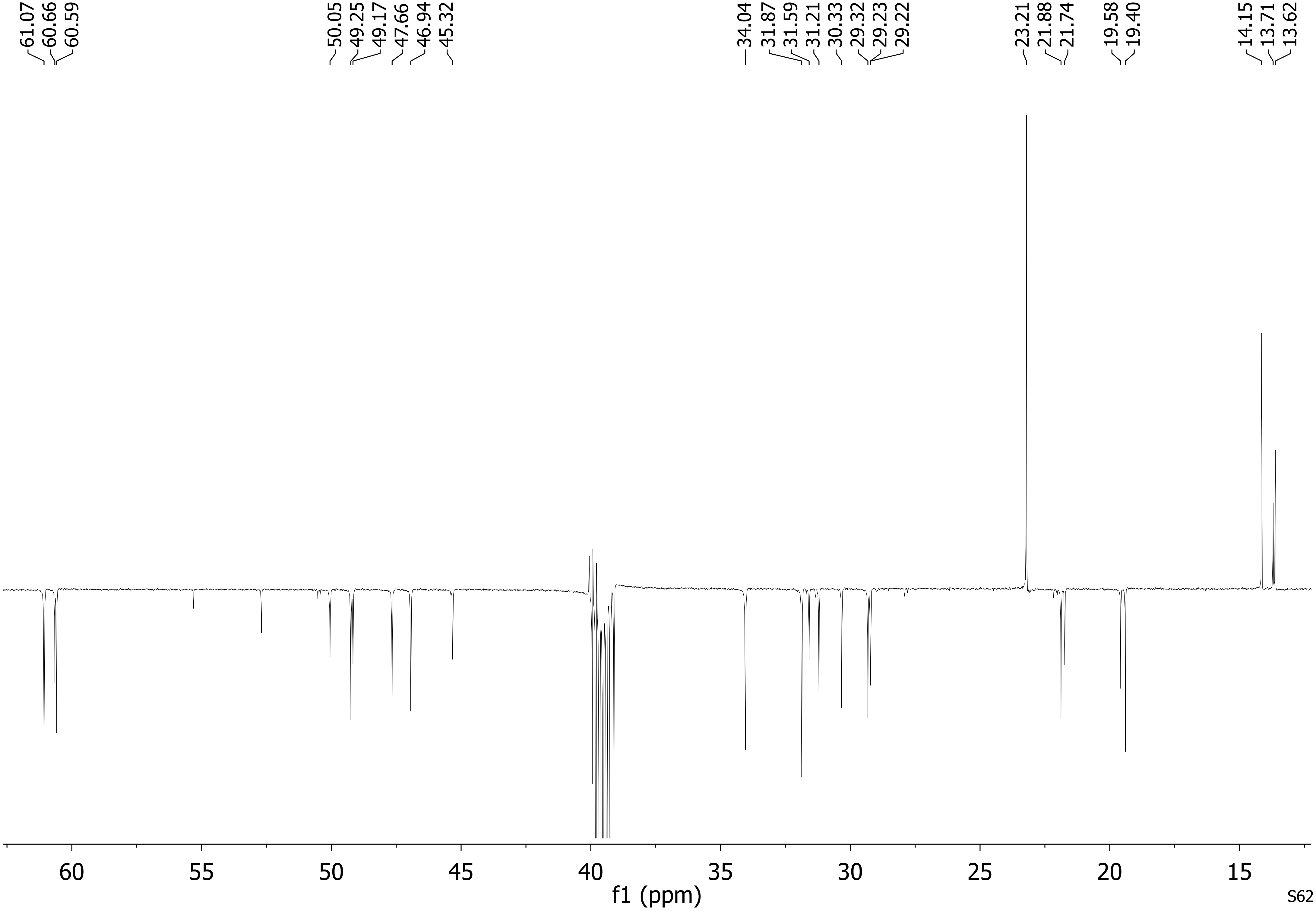

^{13}C NMR (151 MHz, DMSO-d_6) for compound **43** (zoomed-in view 2)

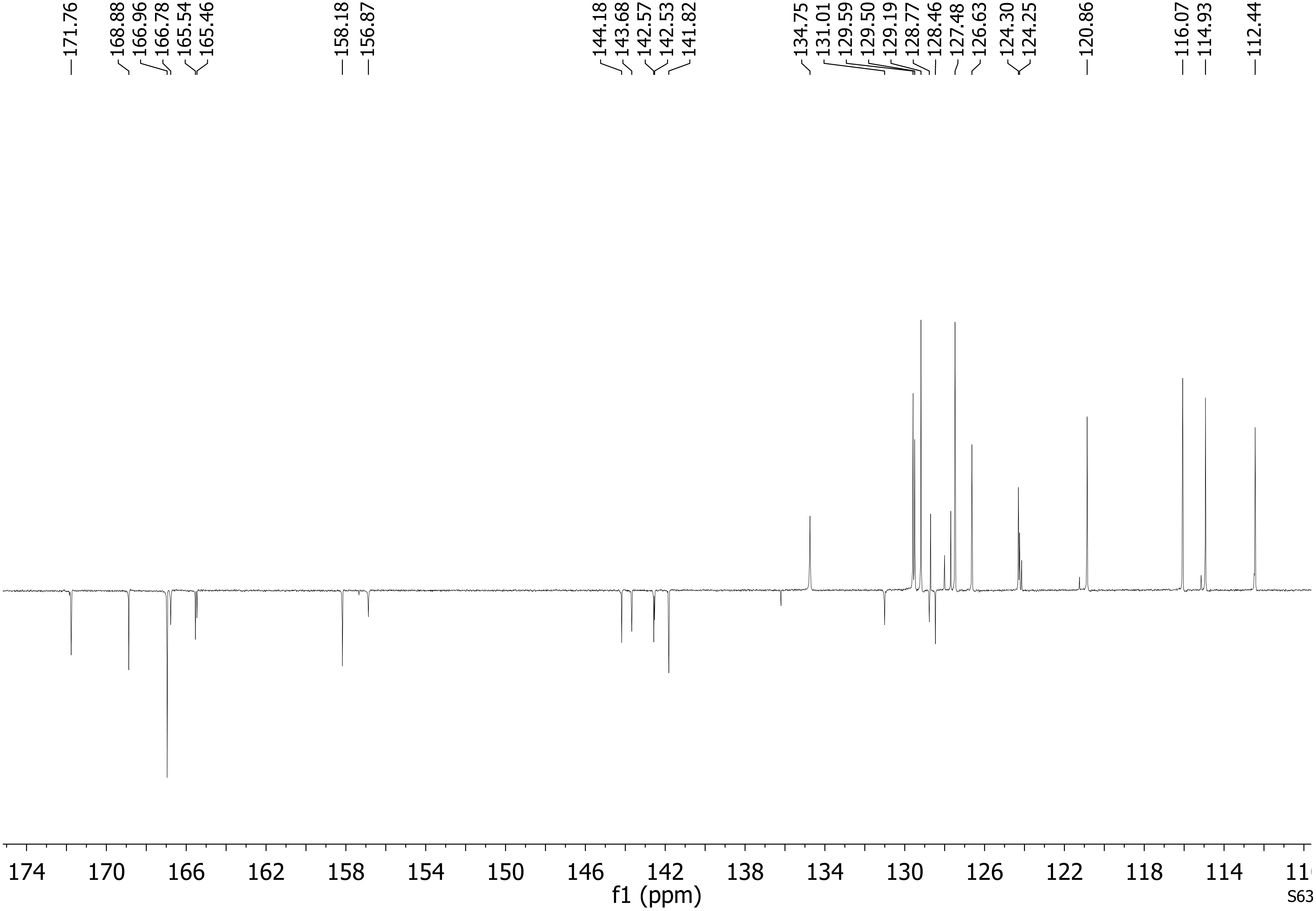

^{1}H NMR (400 MHz, DMSO-d_6) for compound **44**

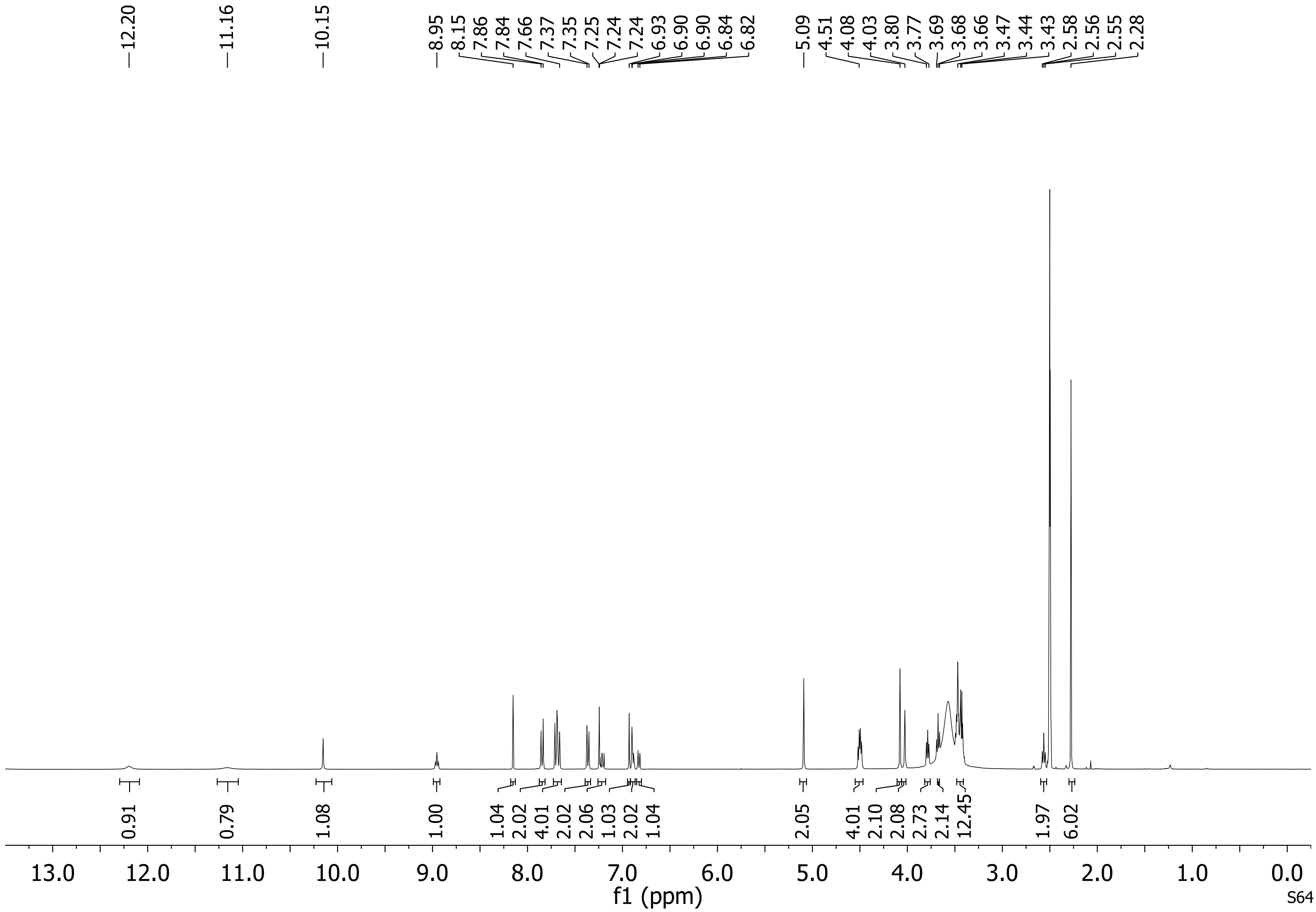

^{13}C NMR (101 MHz, DMSO-d_6) for compound **44** (full view)

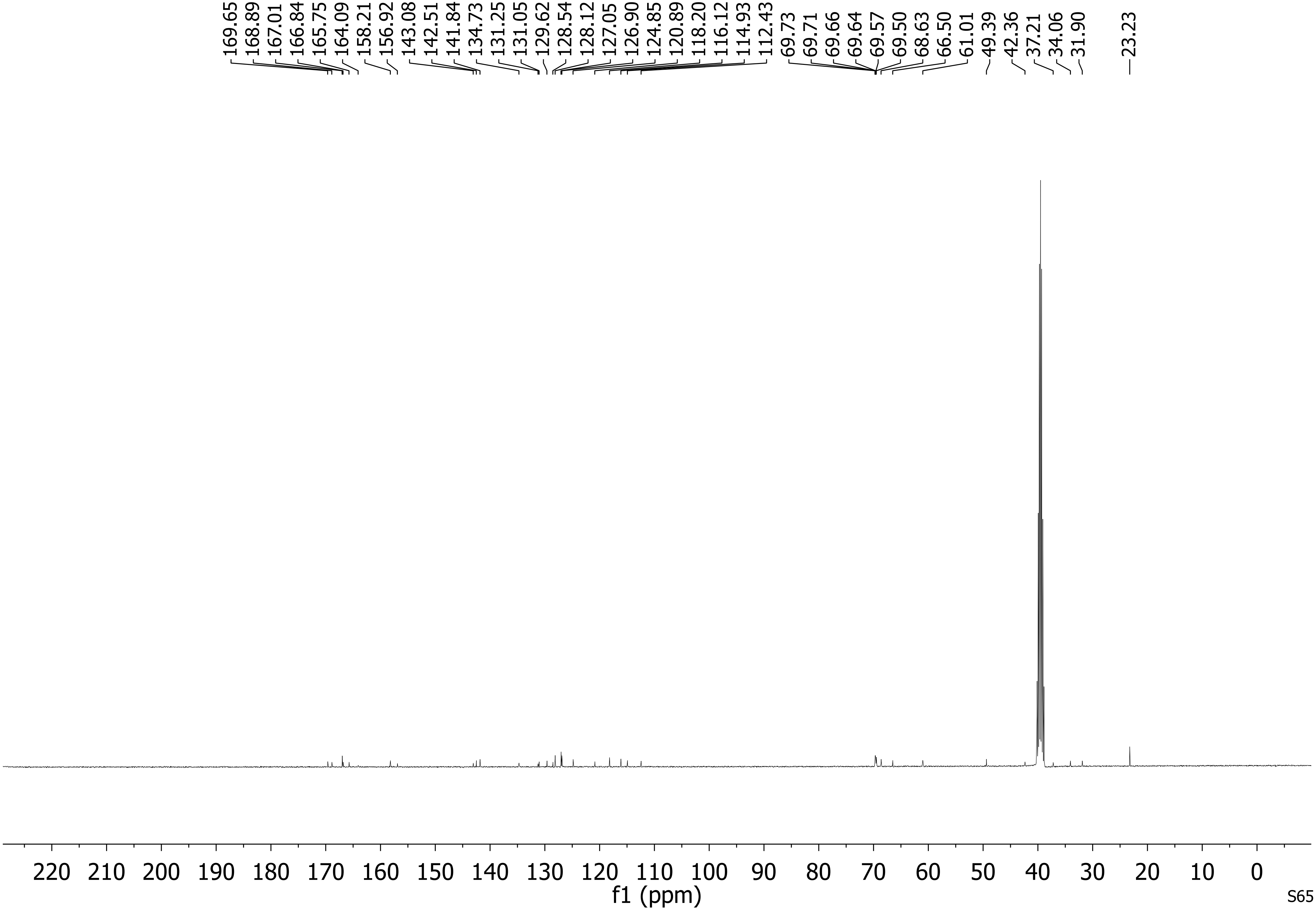

^{13}C NMR (101 MHz, DMSO-d_6) for compound **44** (zoomed-in view 1)

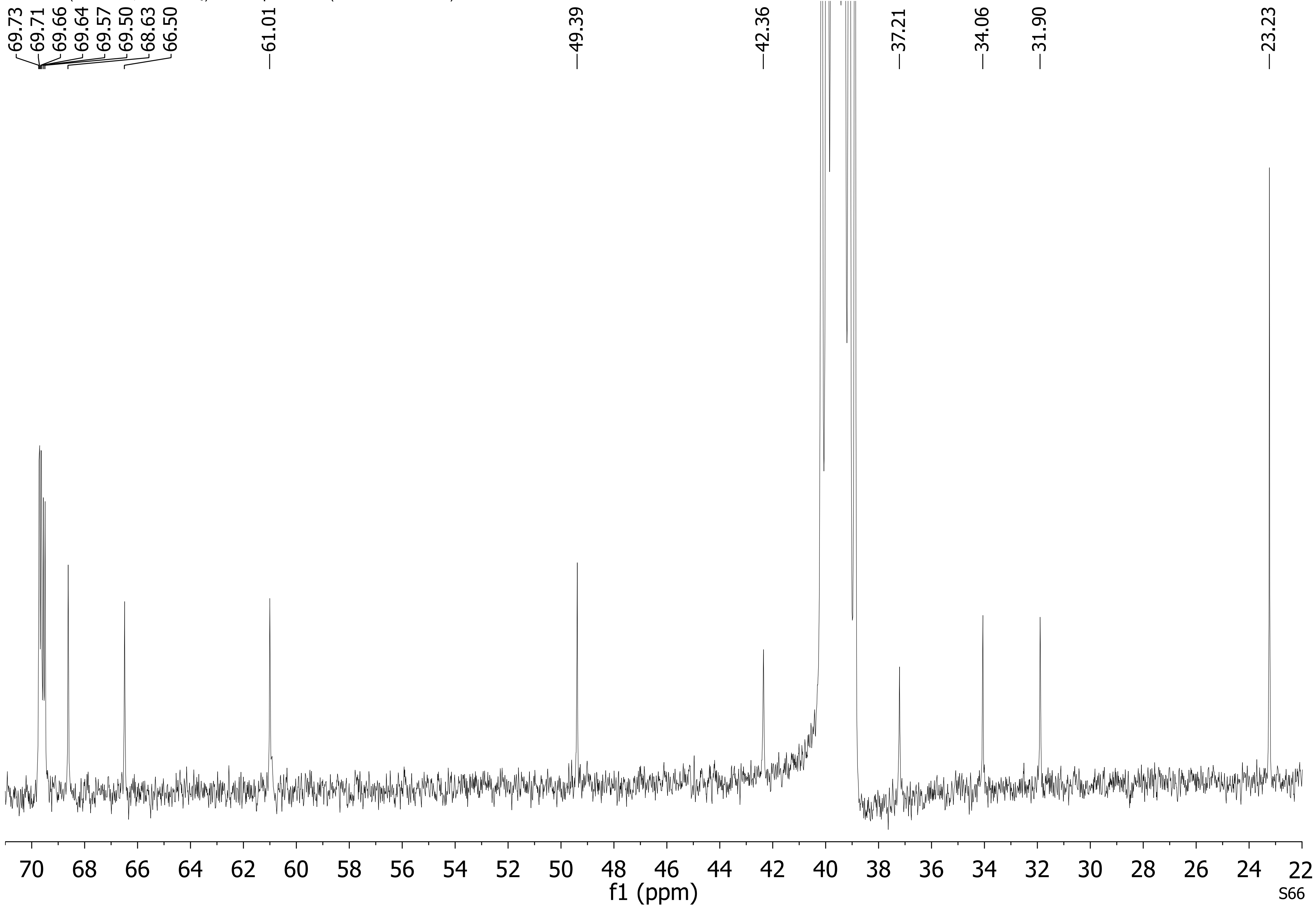

^{13}C NMR (101 MHz, DMSO-d_6) for compound **44** (zoomed-in view 2)

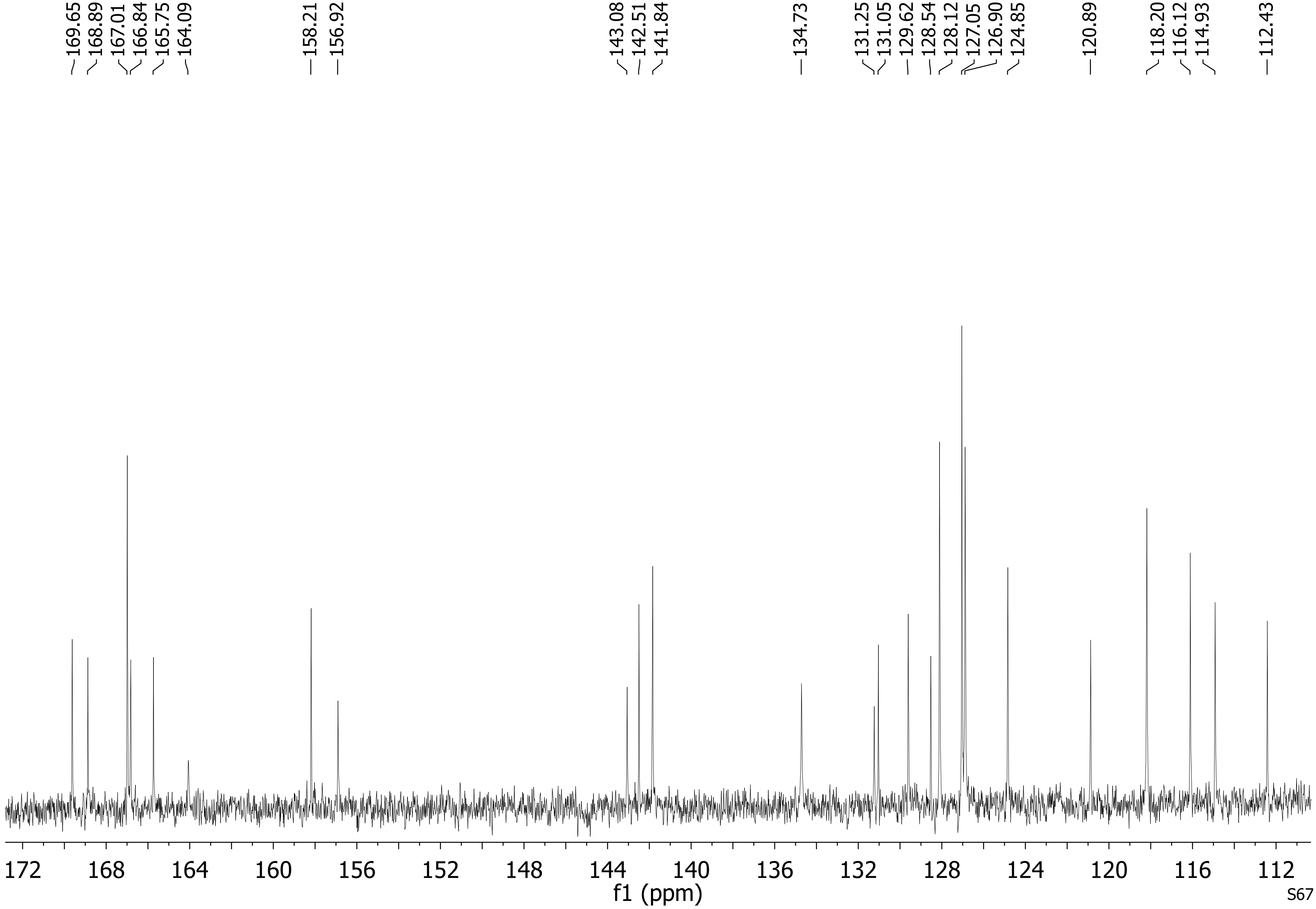

^{19}F NMR (377 MHz, DMSO-d_6) for compound **44**

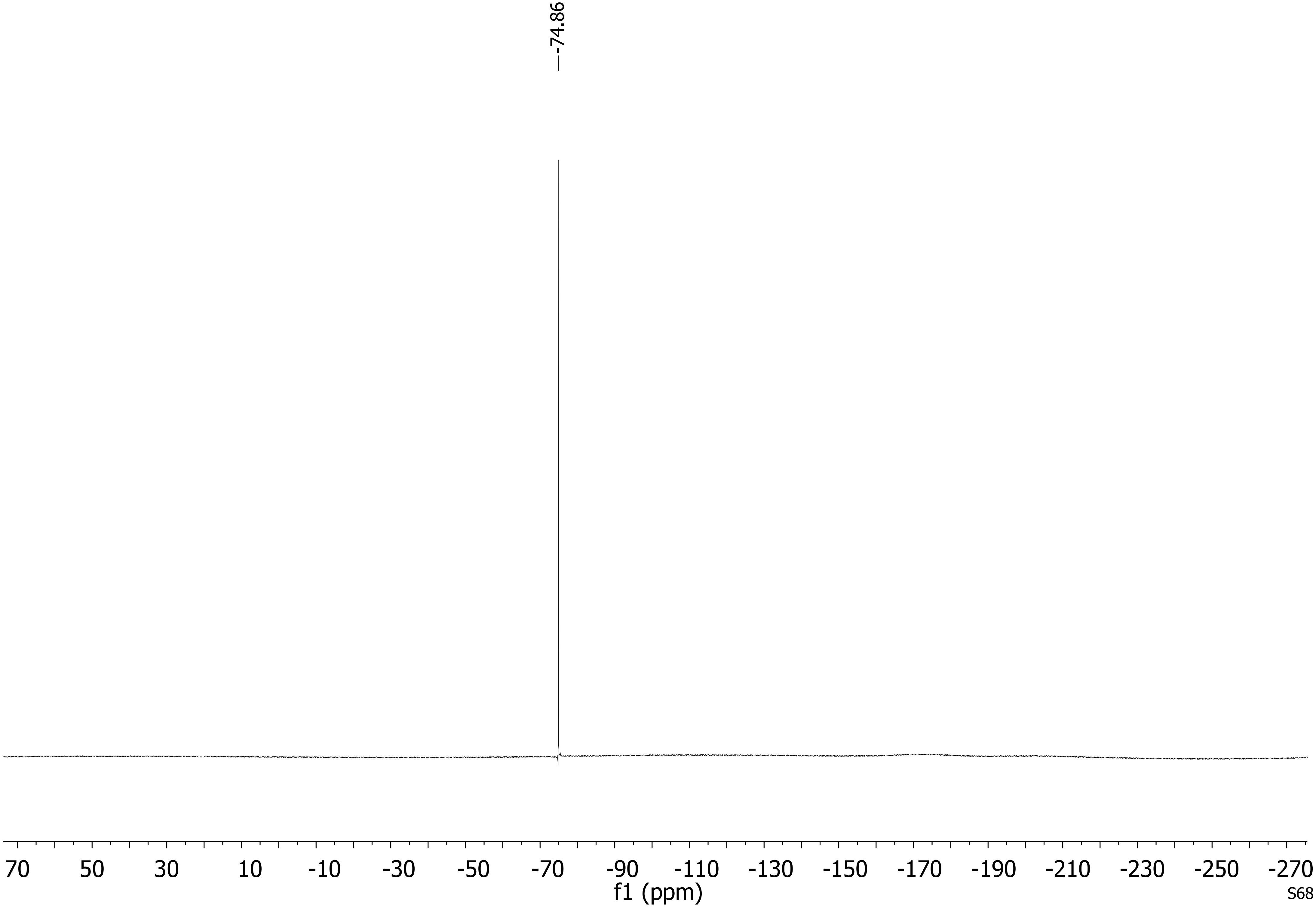

^1H NMR (400 MHz, DMSO-d_6) for compound **45**

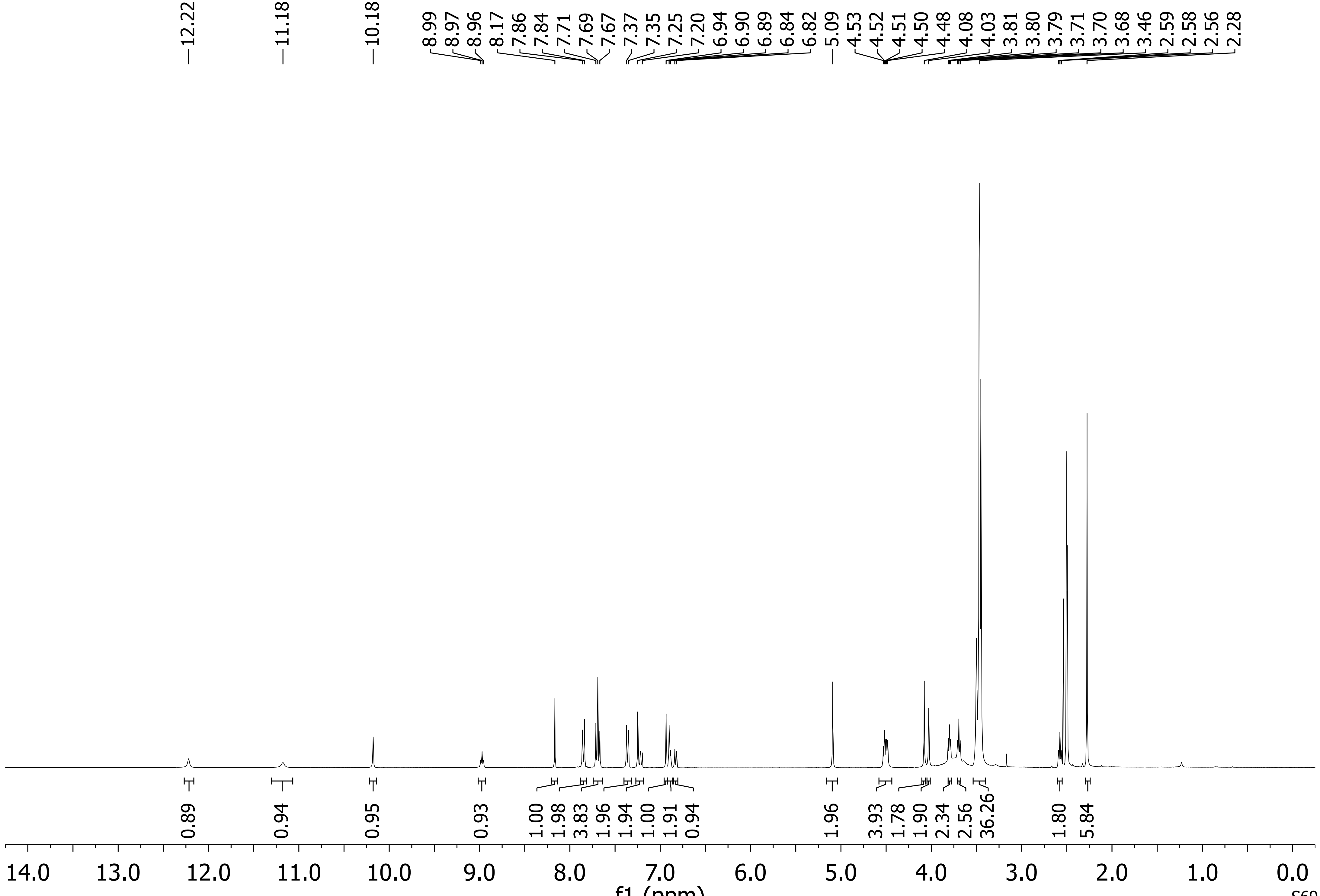

^{13}C NMR (151 MHz, DMSO-d_6) for compound **45** (full view)

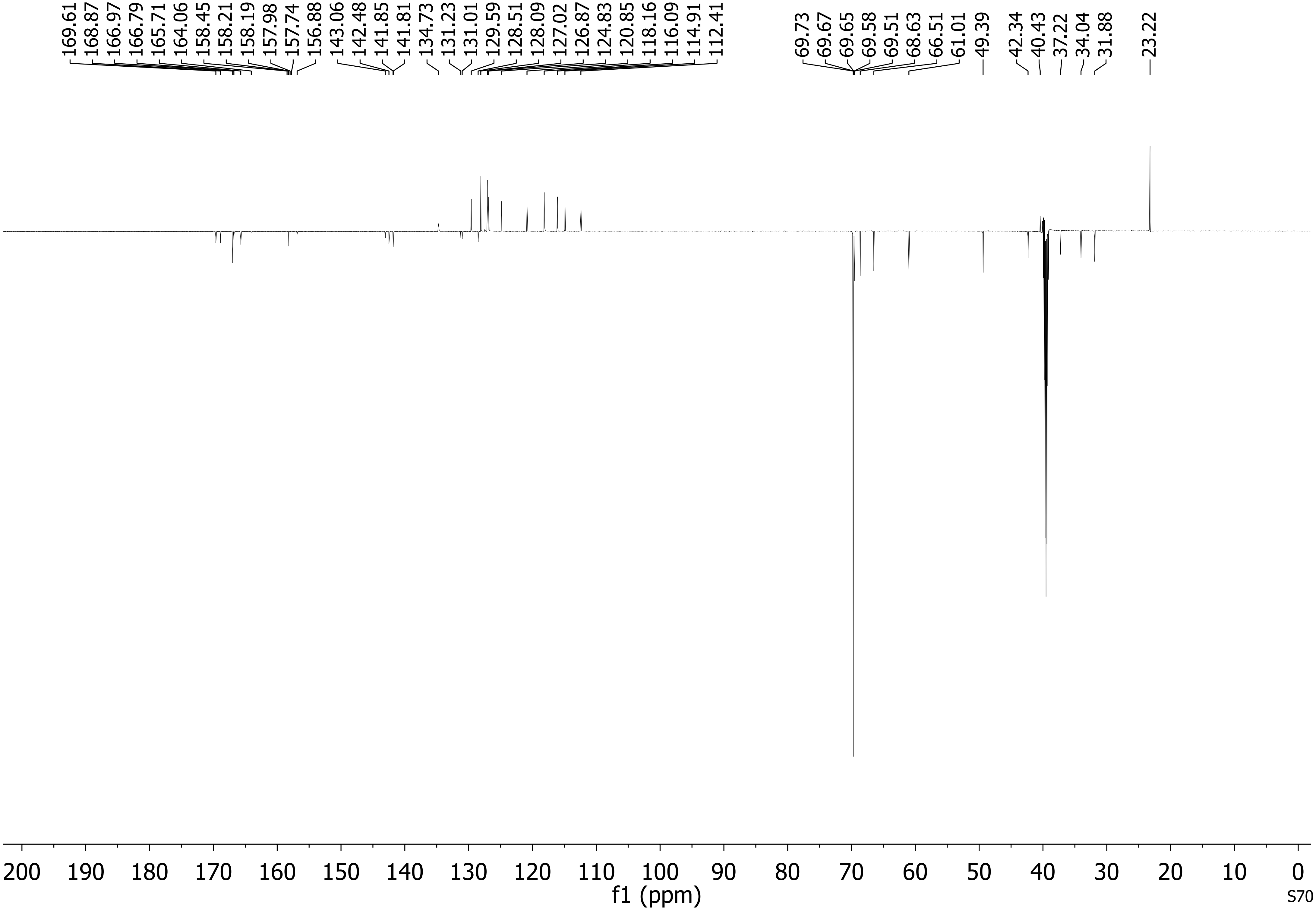

^{13}C NMR (151 MHz, DMSO-d_6) for compound **45** (zoomed-in view 1)

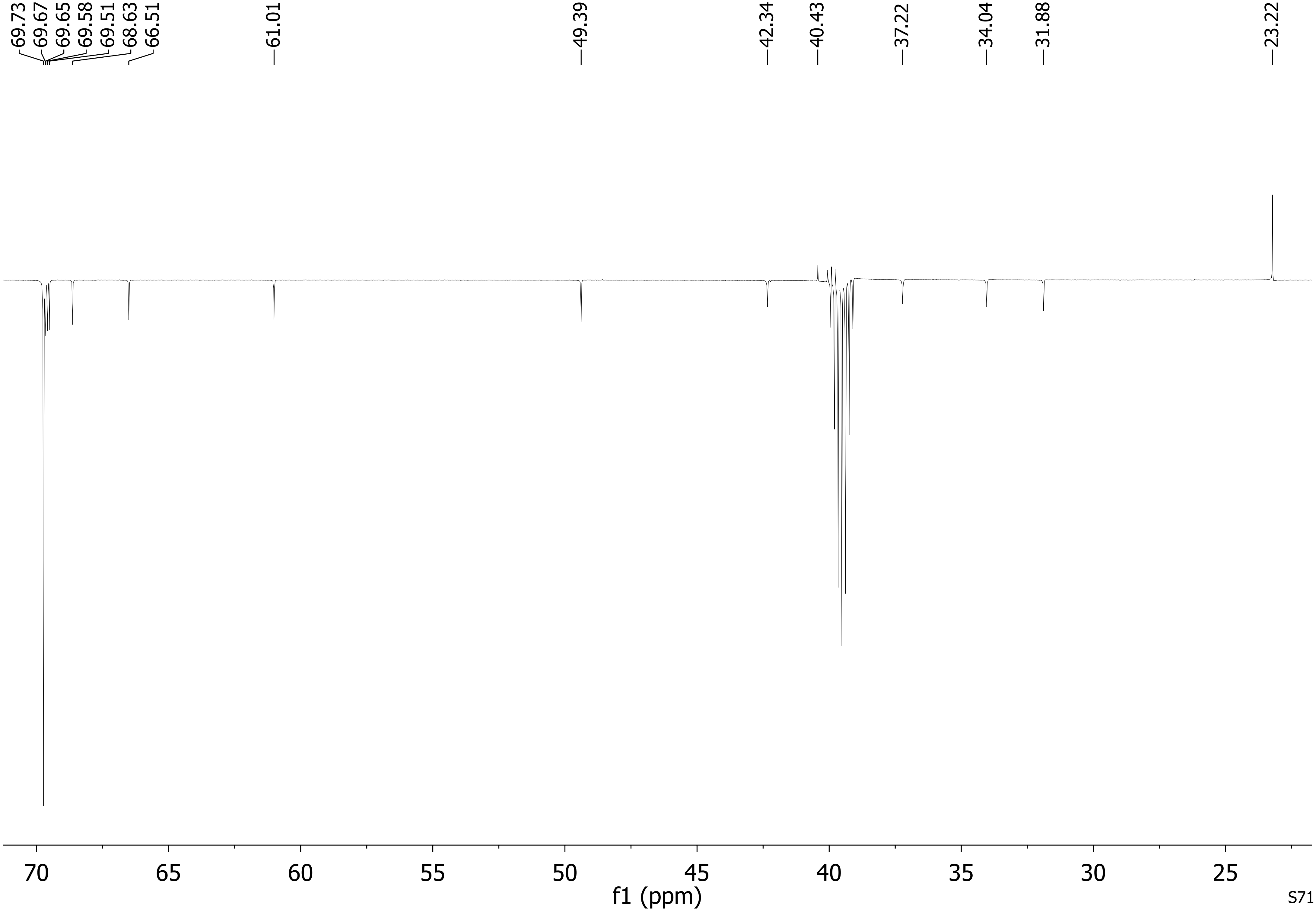

^{13}C NMR (151 MHz, DMSO-d_6) for compound **45** (zoomed-in view 2)

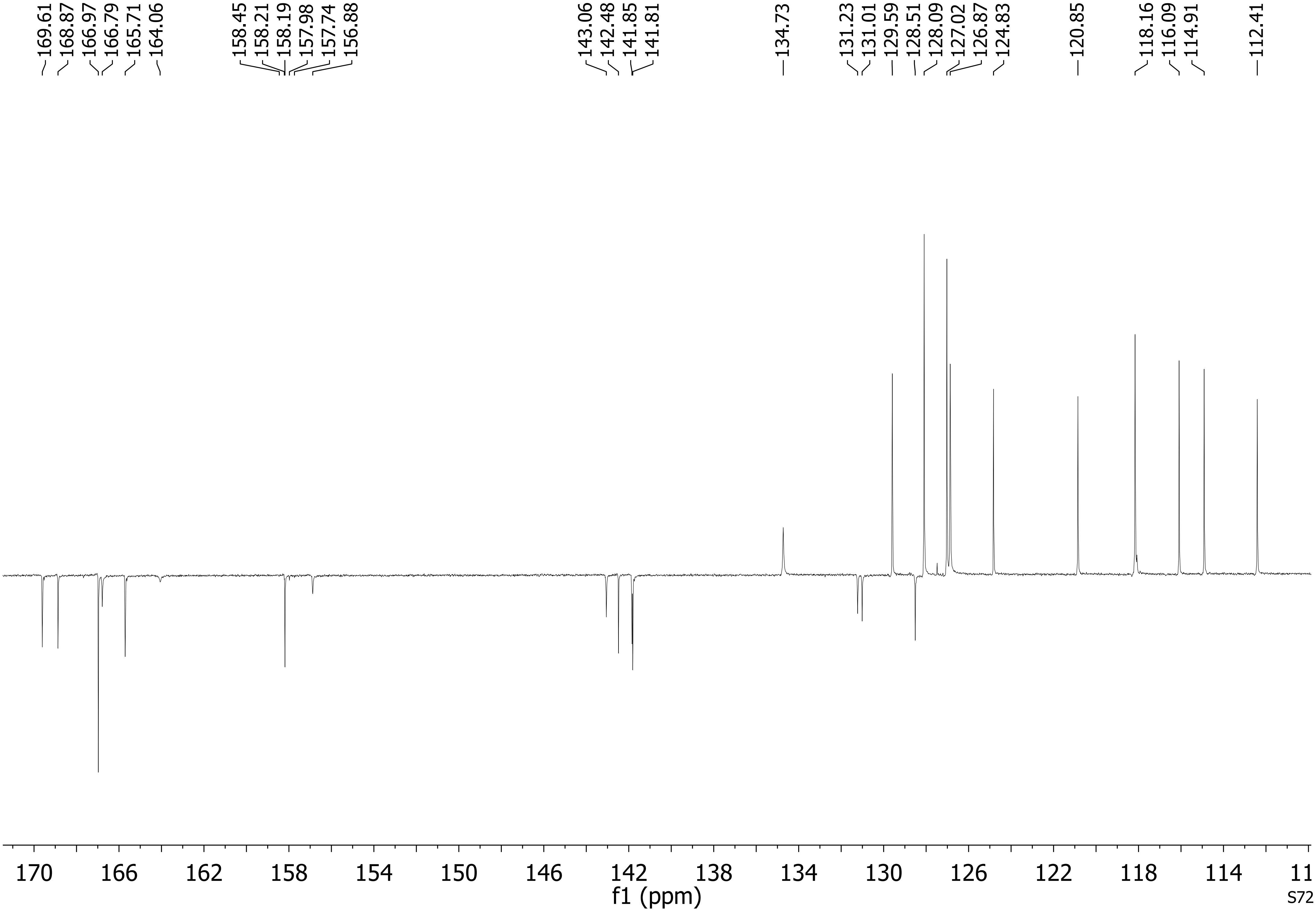

^{19}F NMR (565 MHz, DMSO-d_6) for compound **45**

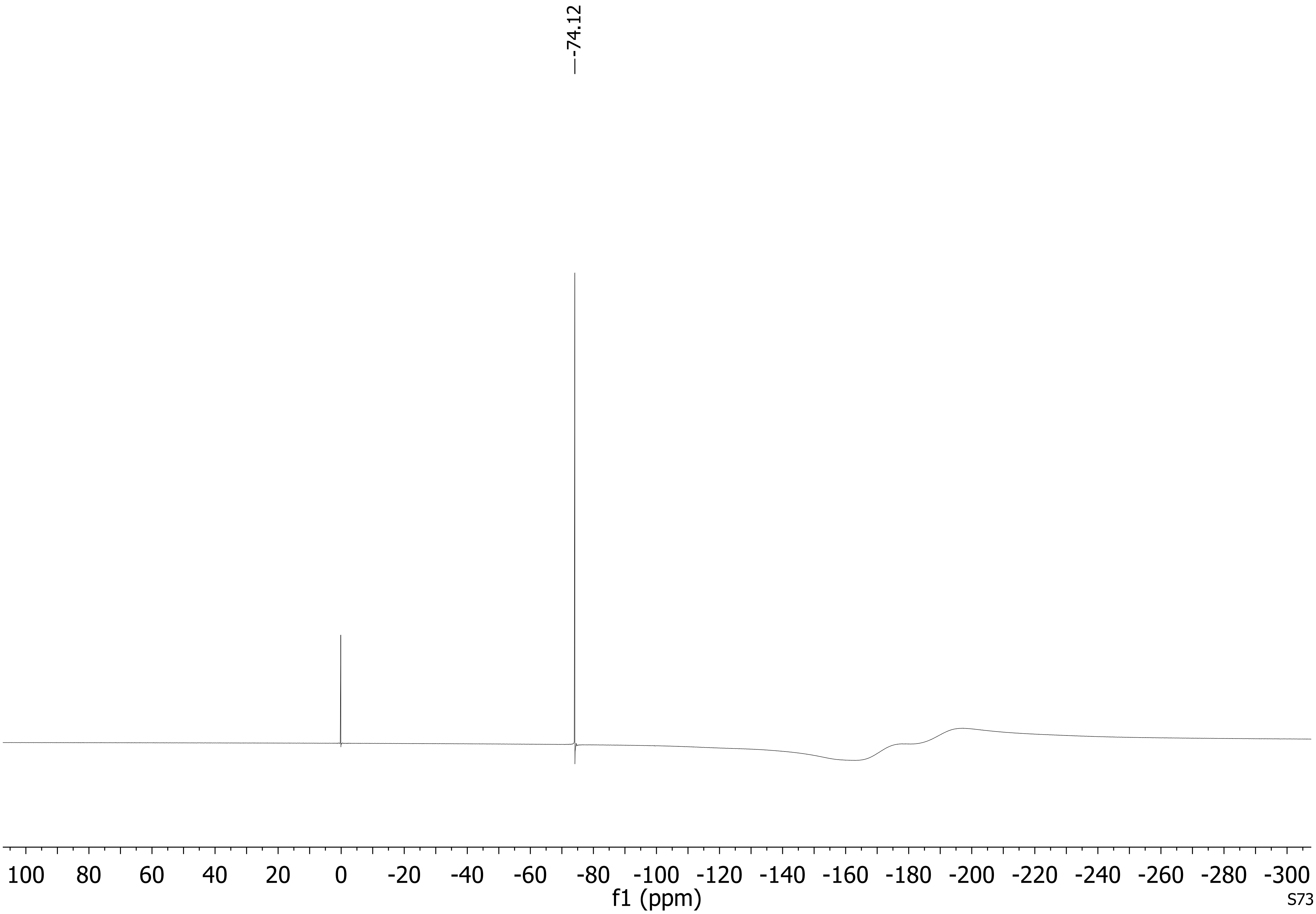

^{1}H NMR (400 MHz, DMSO-d_6) for compound **46**

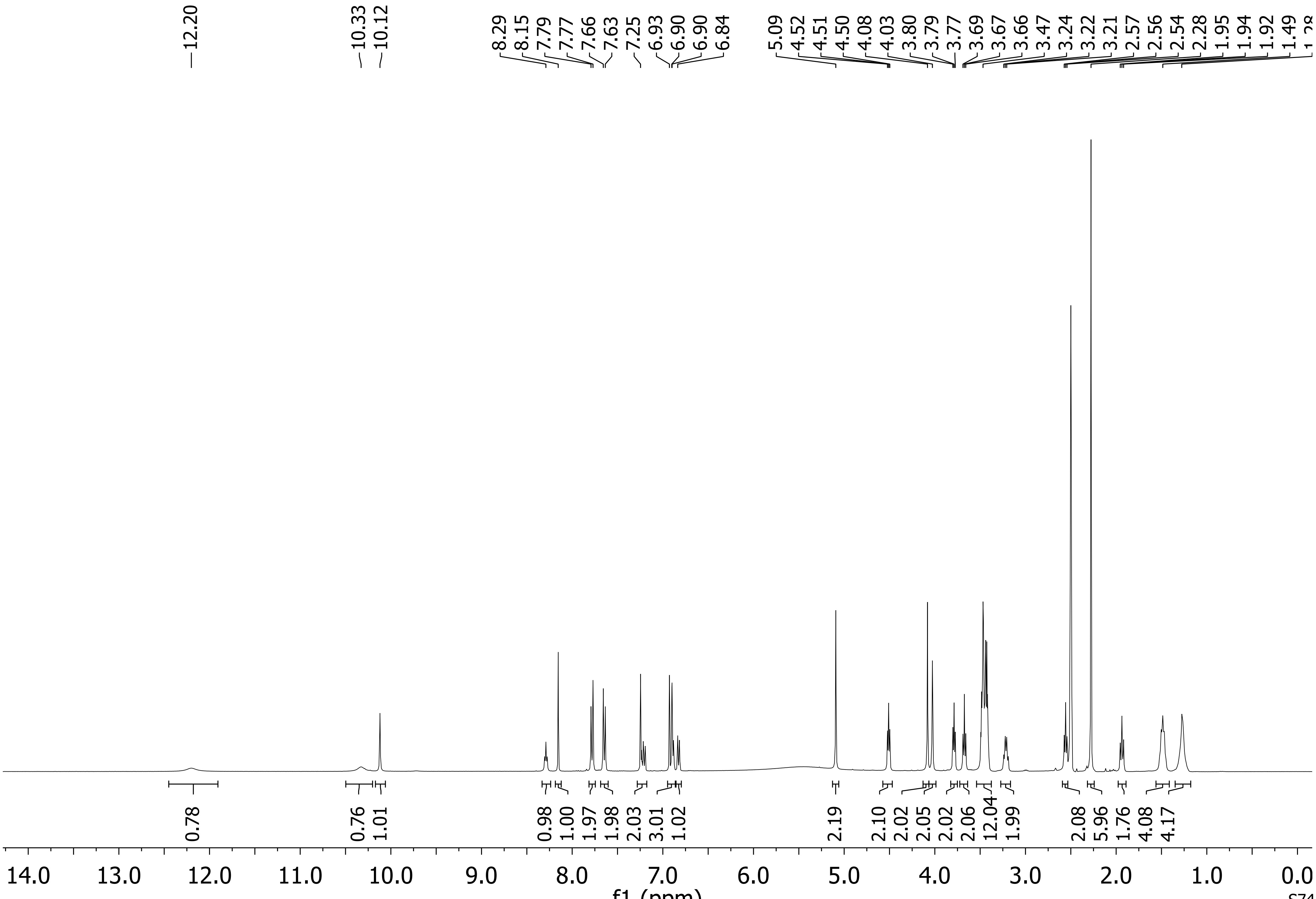

^{13}C NMR (101 MHz, DMSO-d_6) for compound **46** (full view)

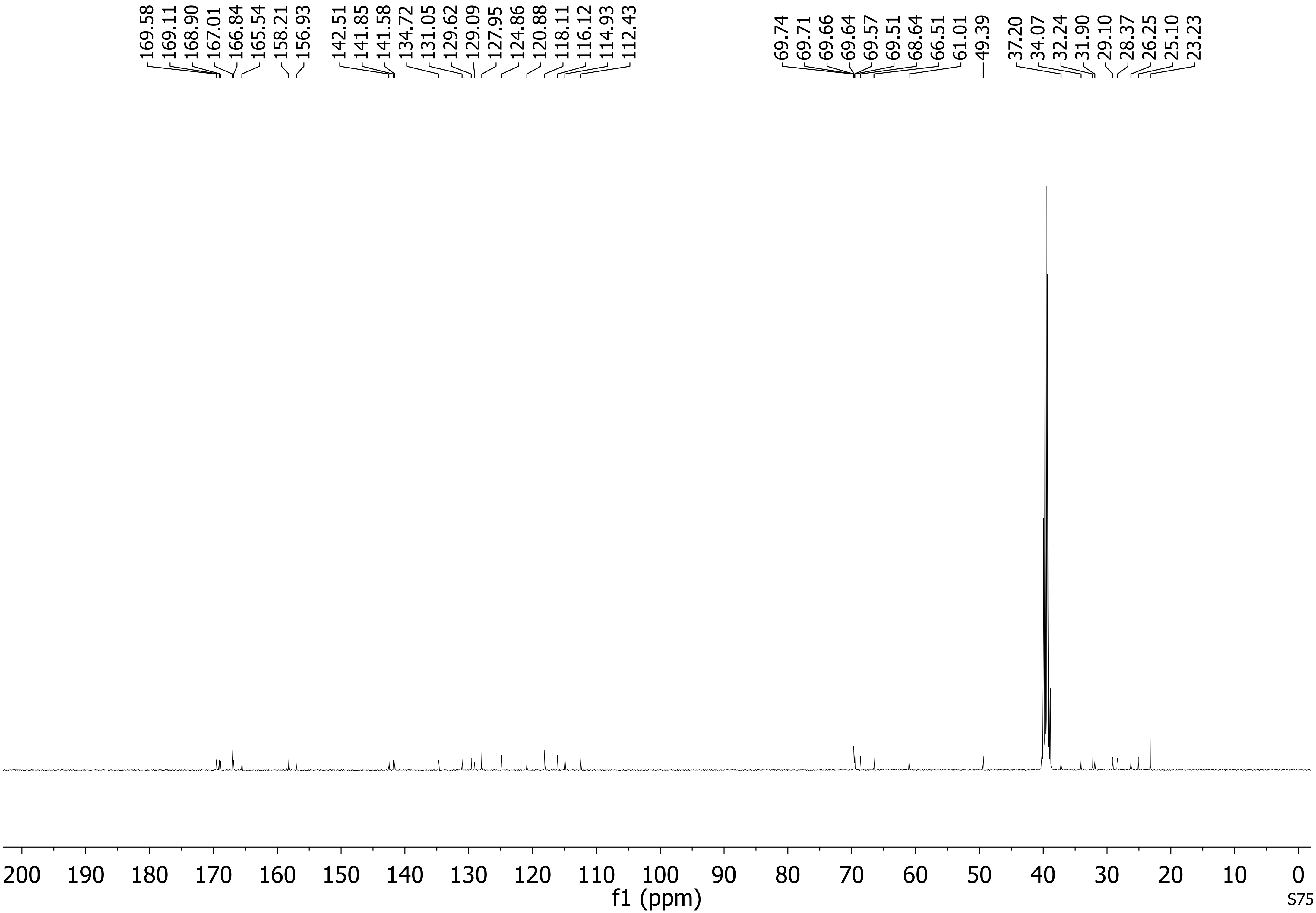

^{13}C NMR (101 MHz, DMSO-d_6) for compound **46** (zoomed-in view 1)

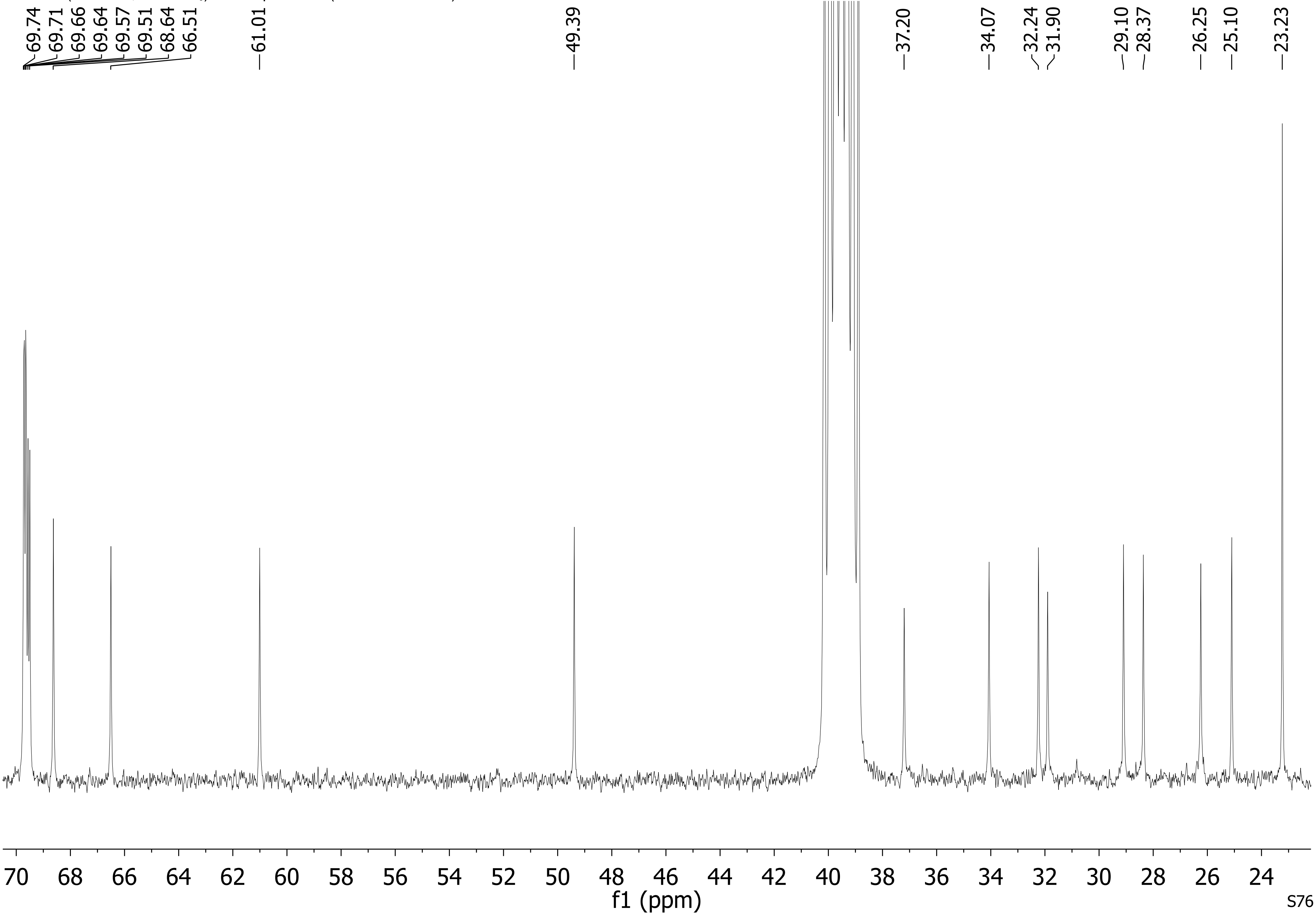

^{13}C NMR (101 MHz, DMSO-d_6) for compound **46** (zoomed-in view 2)

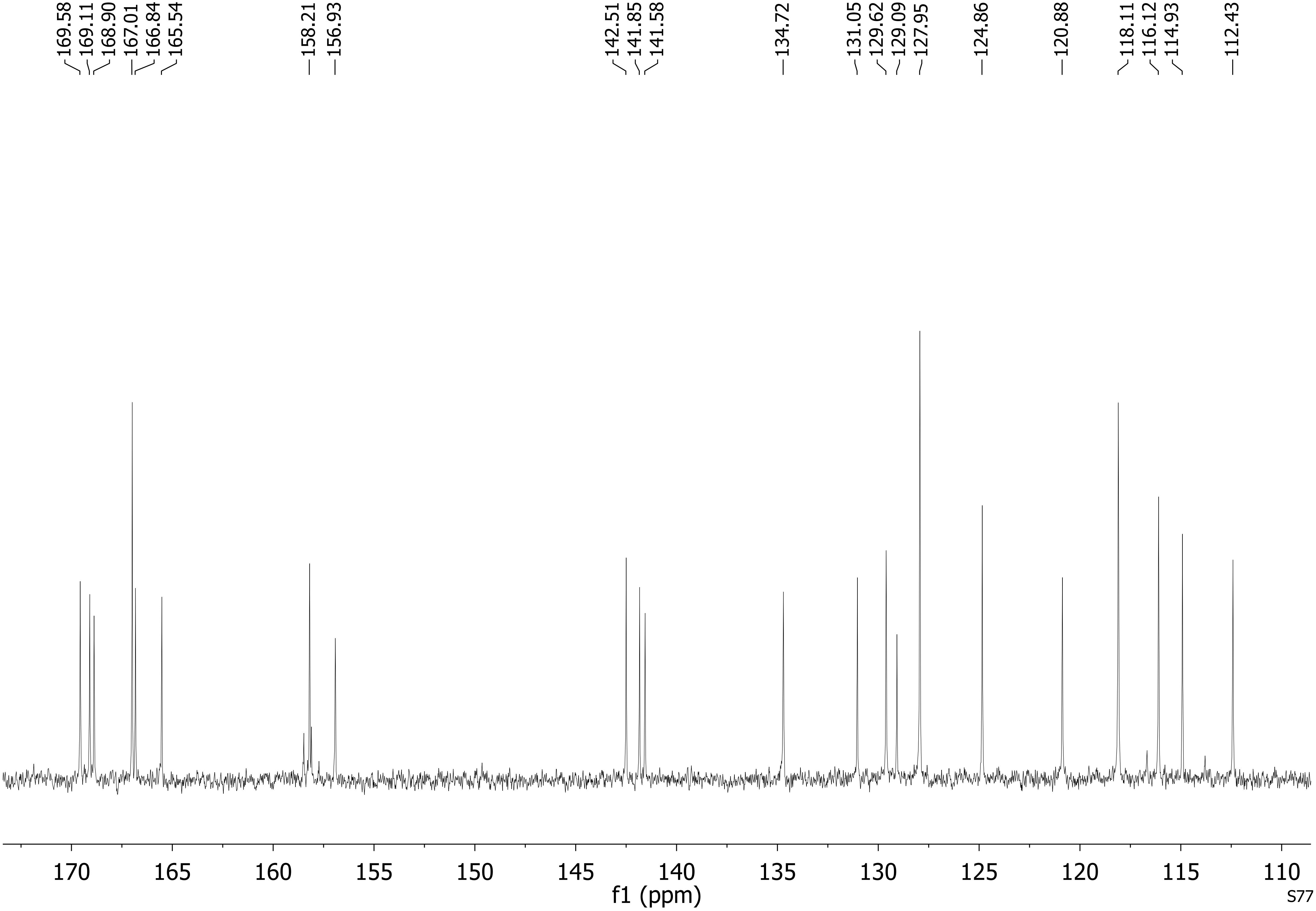

^{19}F NMR (377 MHz, DMSO-d_6) for compound **46**

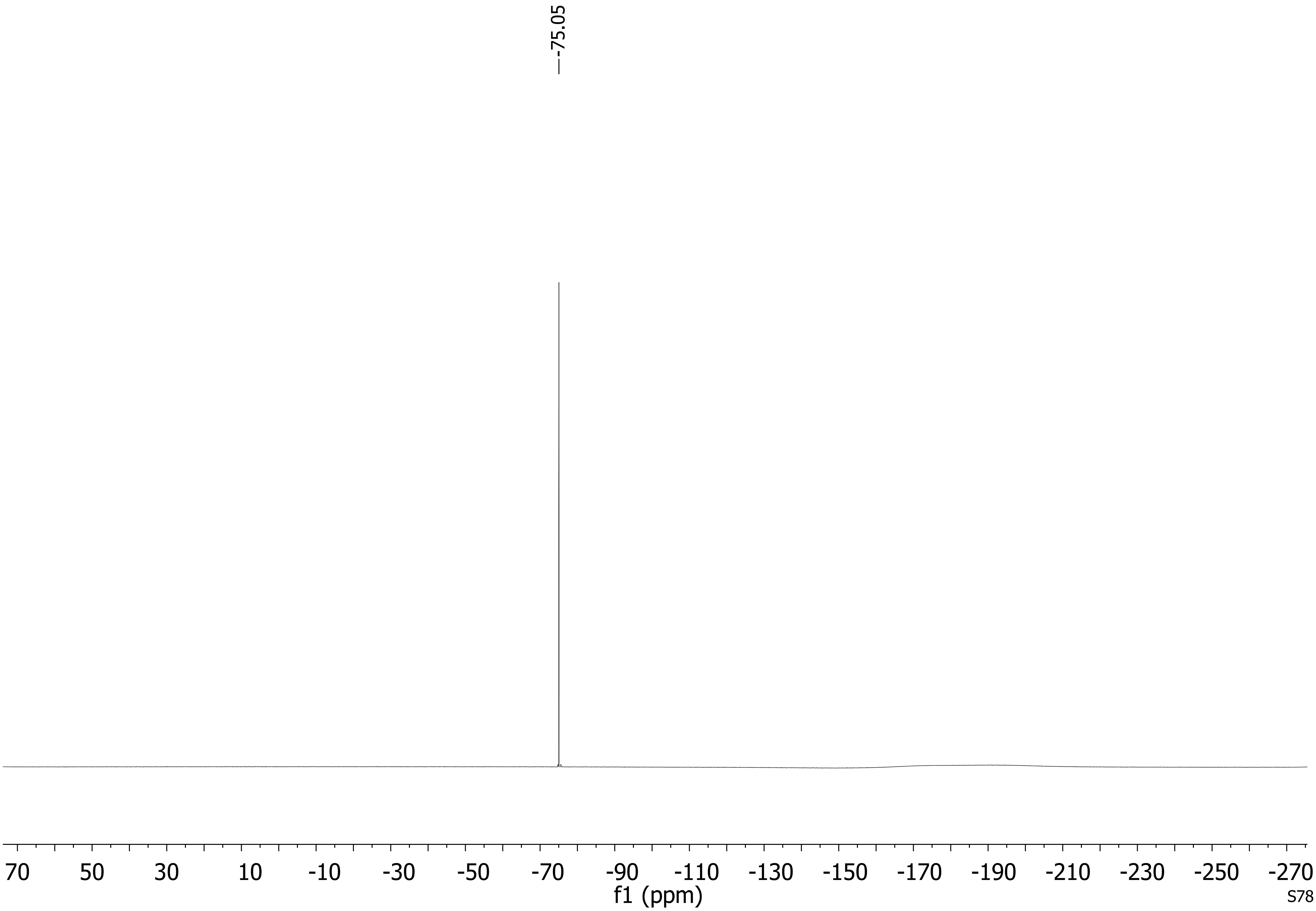

^{1}H NMR (400 MHz, DMSO-d_6) for compound **55**

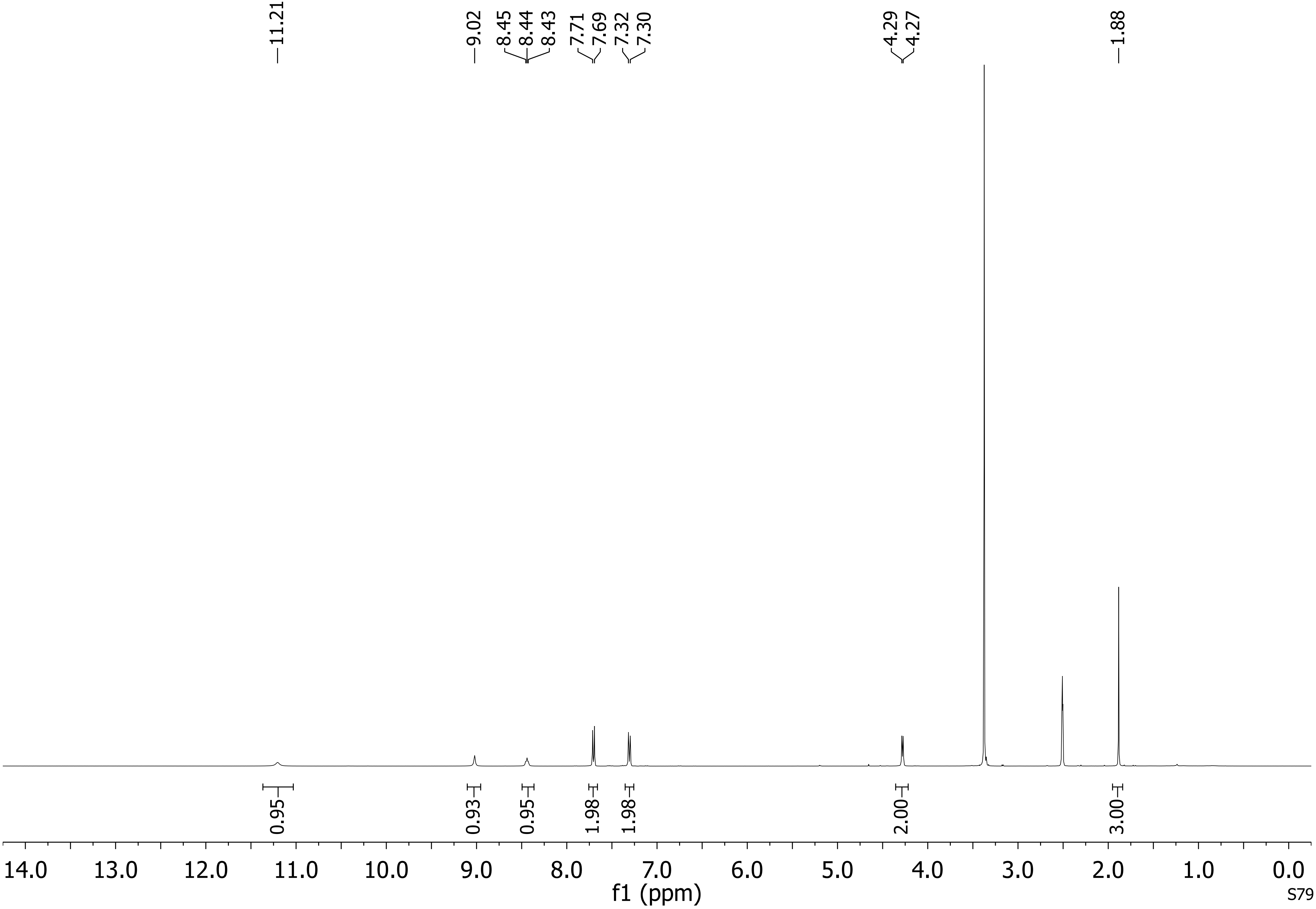

^{13}C NMR (101 MHz, DMSO-d_6) for compound **55**

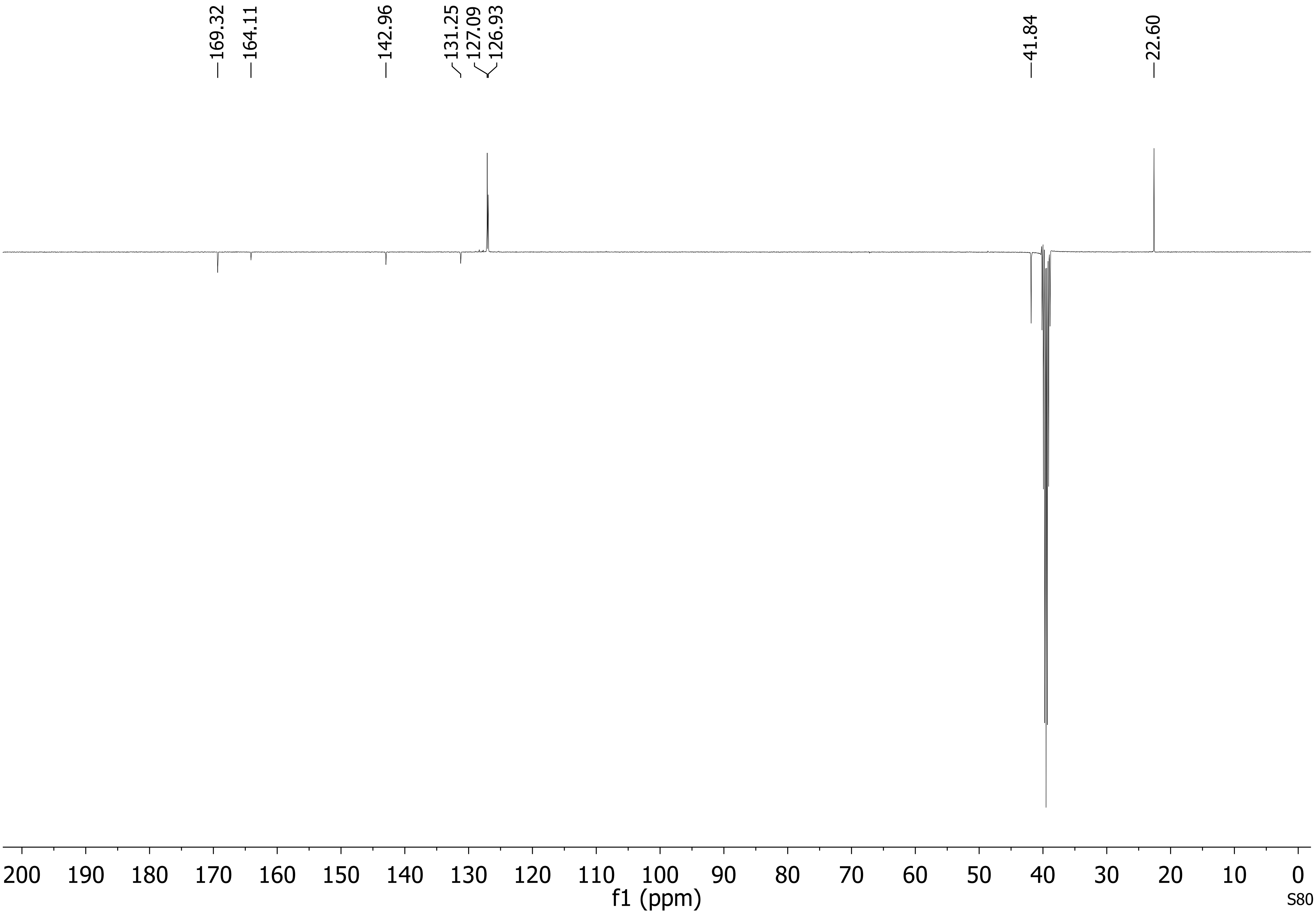

^{1}H NMR (600 MHz, DMSO-d_6) for compound **56**

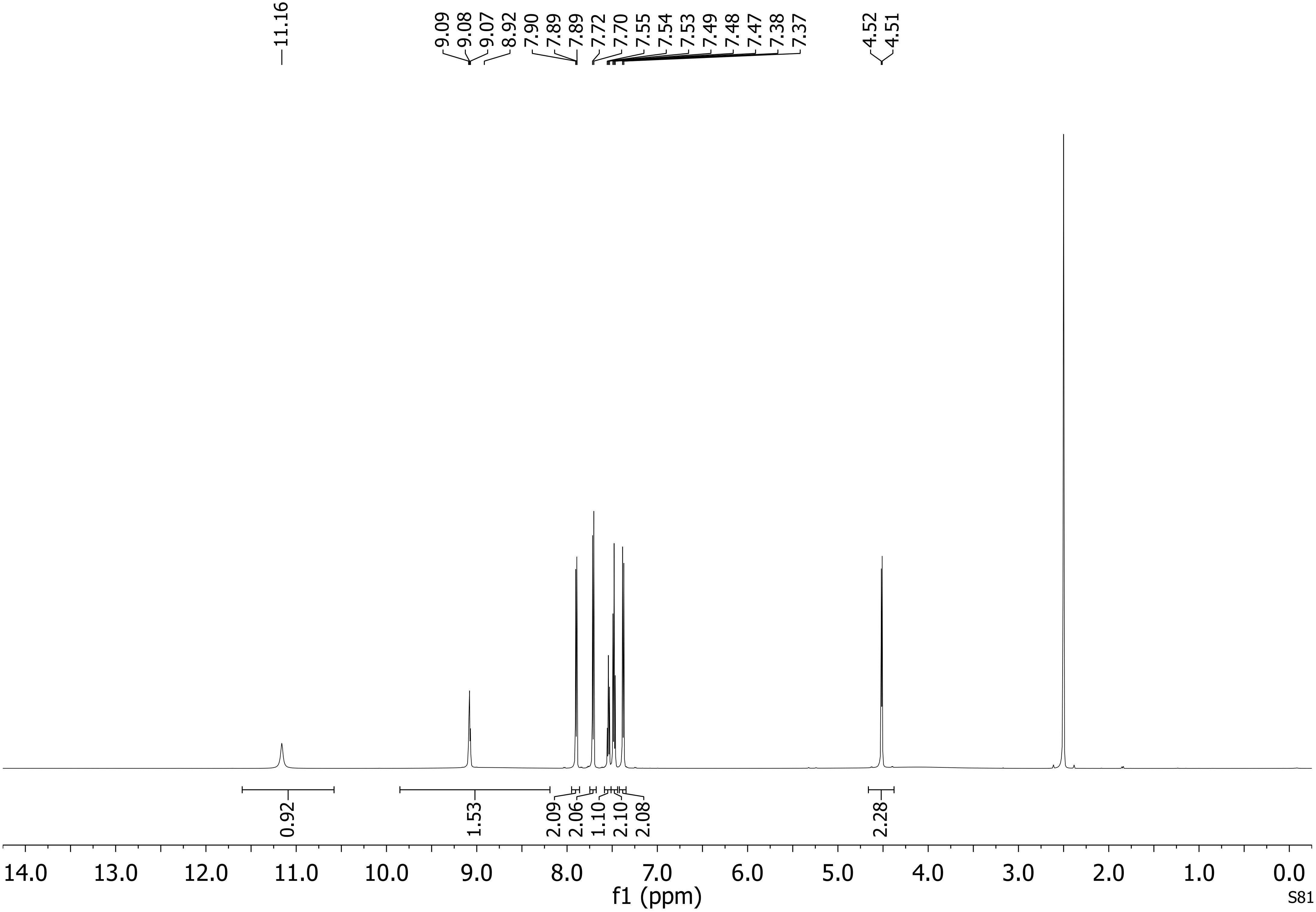

^{13}C NMR (151 MHz, DMSO-d_6) for compound **56**

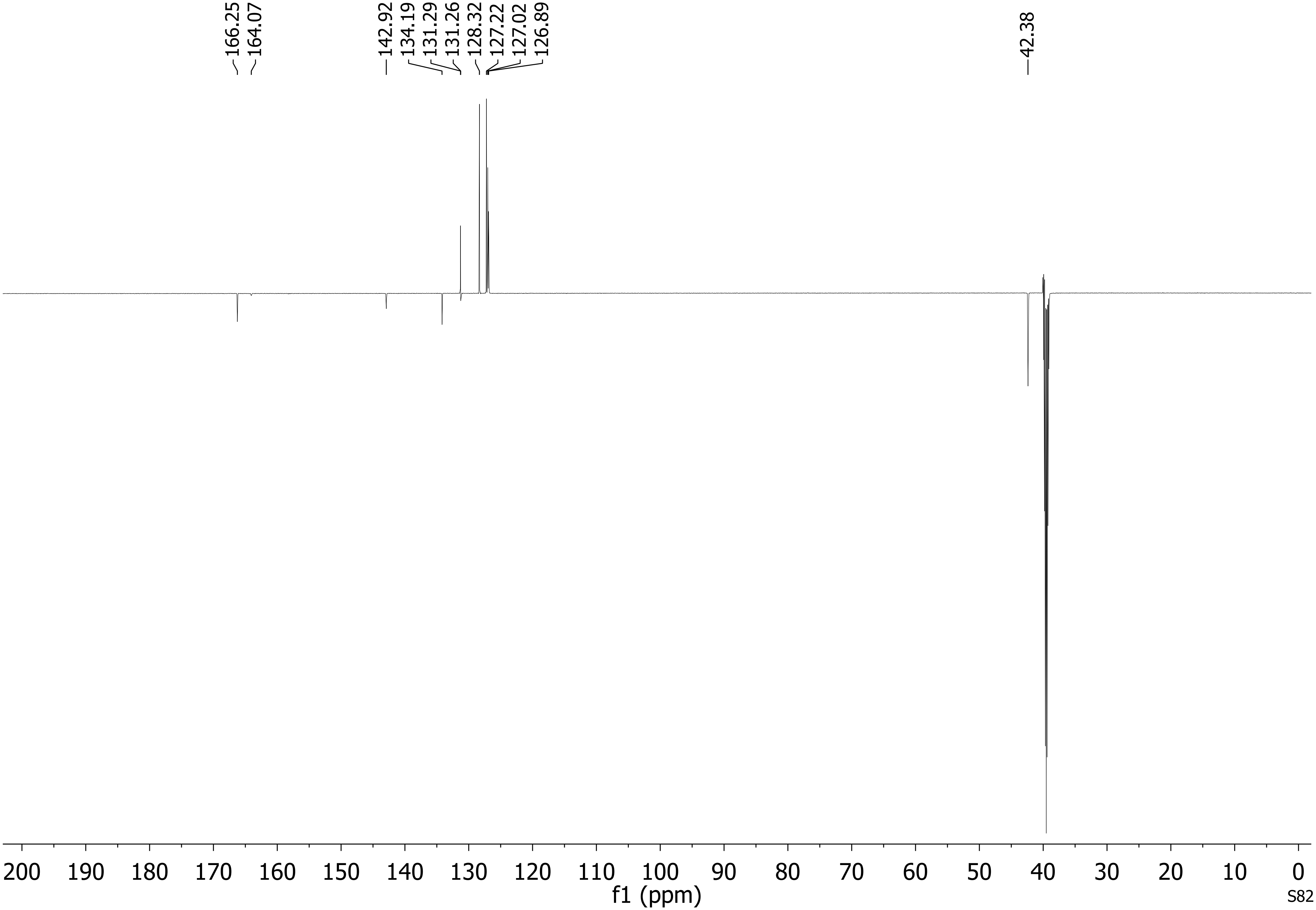

^{1}H NMR (400 MHz, DMSO-d_6) for compound **57**

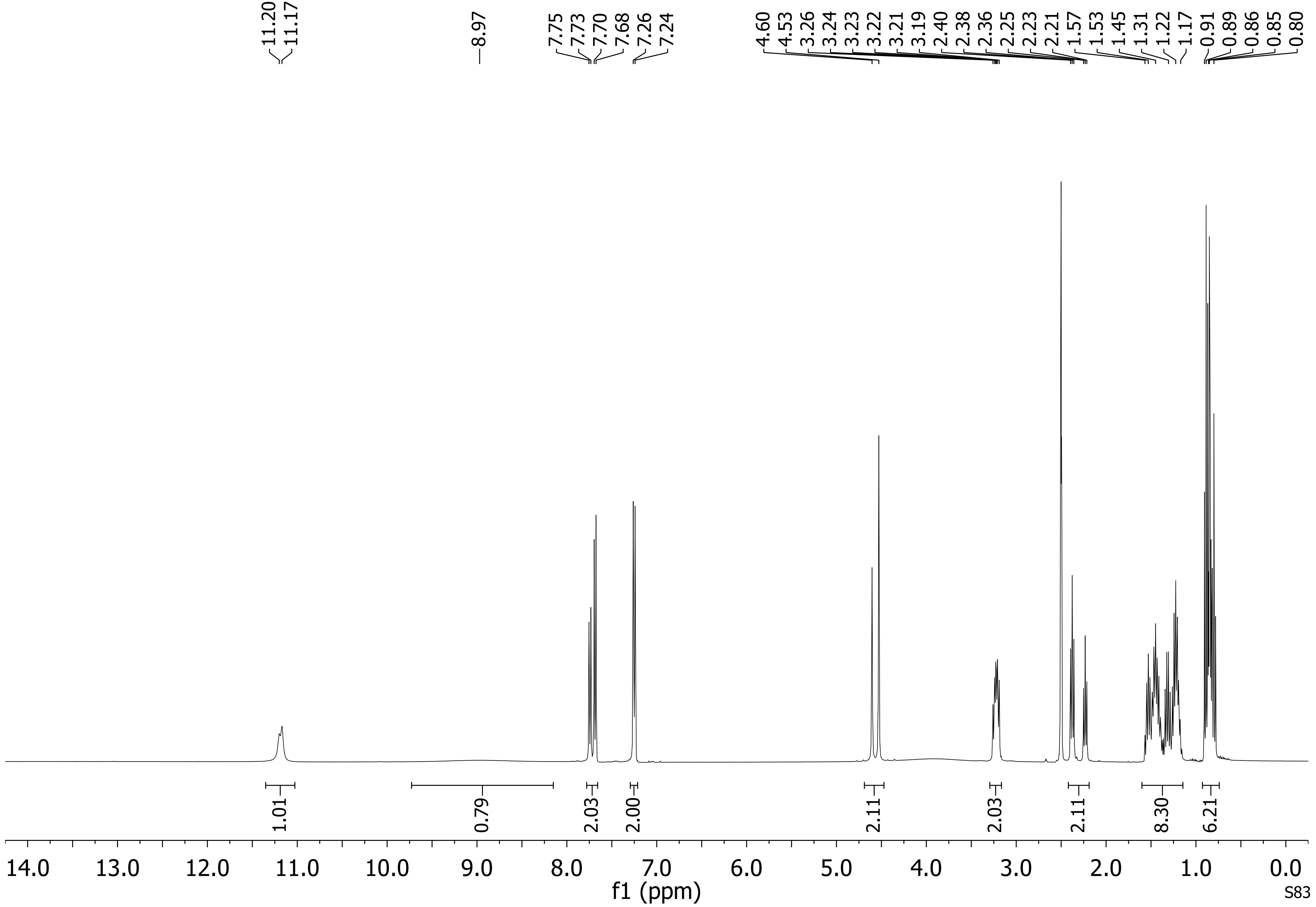

^{13}C NMR (101 MHz, DMSO-d_6) for compound **57** (full view)

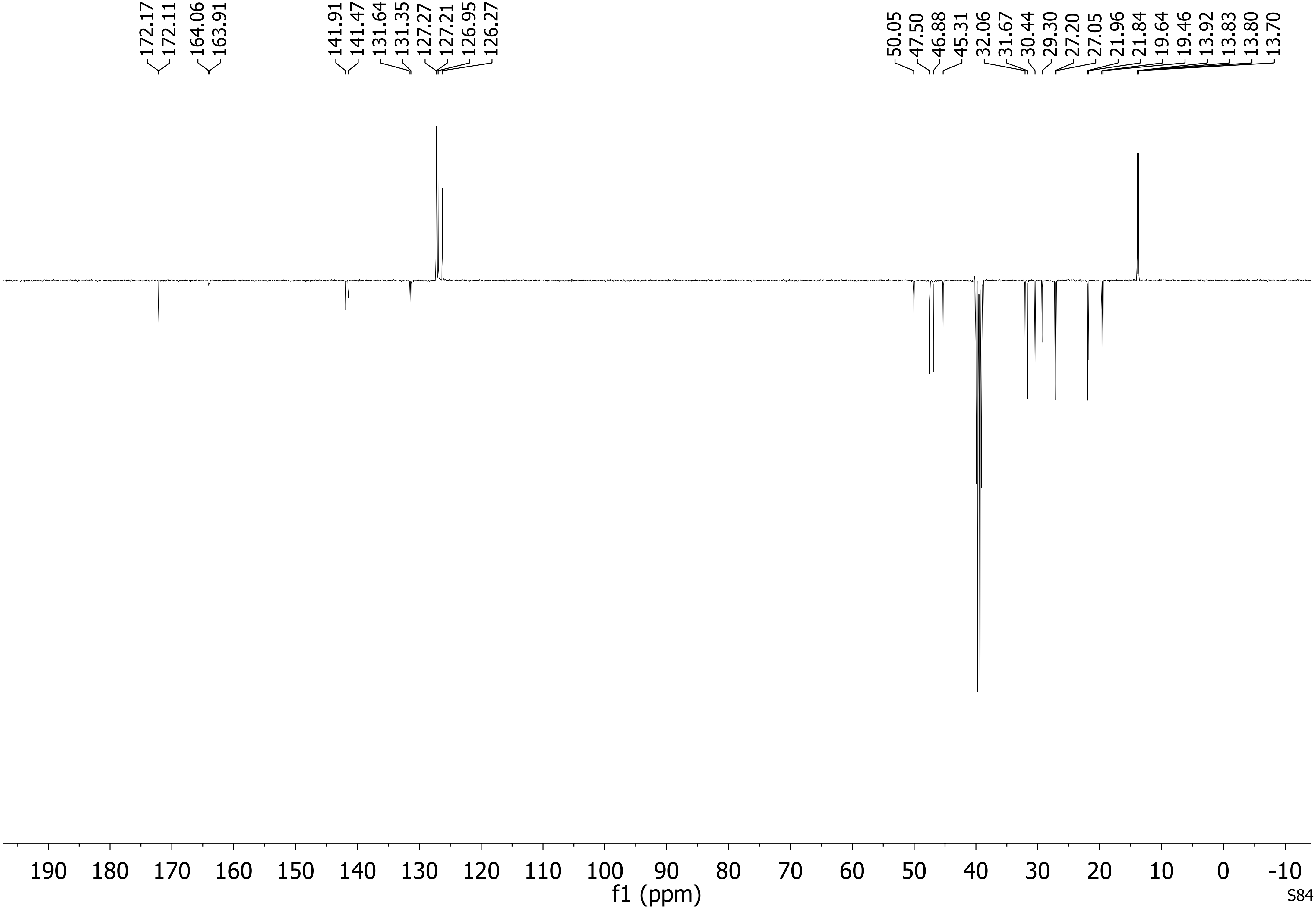

^{13}C NMR (101 MHz, DMSO-d_6) for compound **57** (zoomed-in view 1)

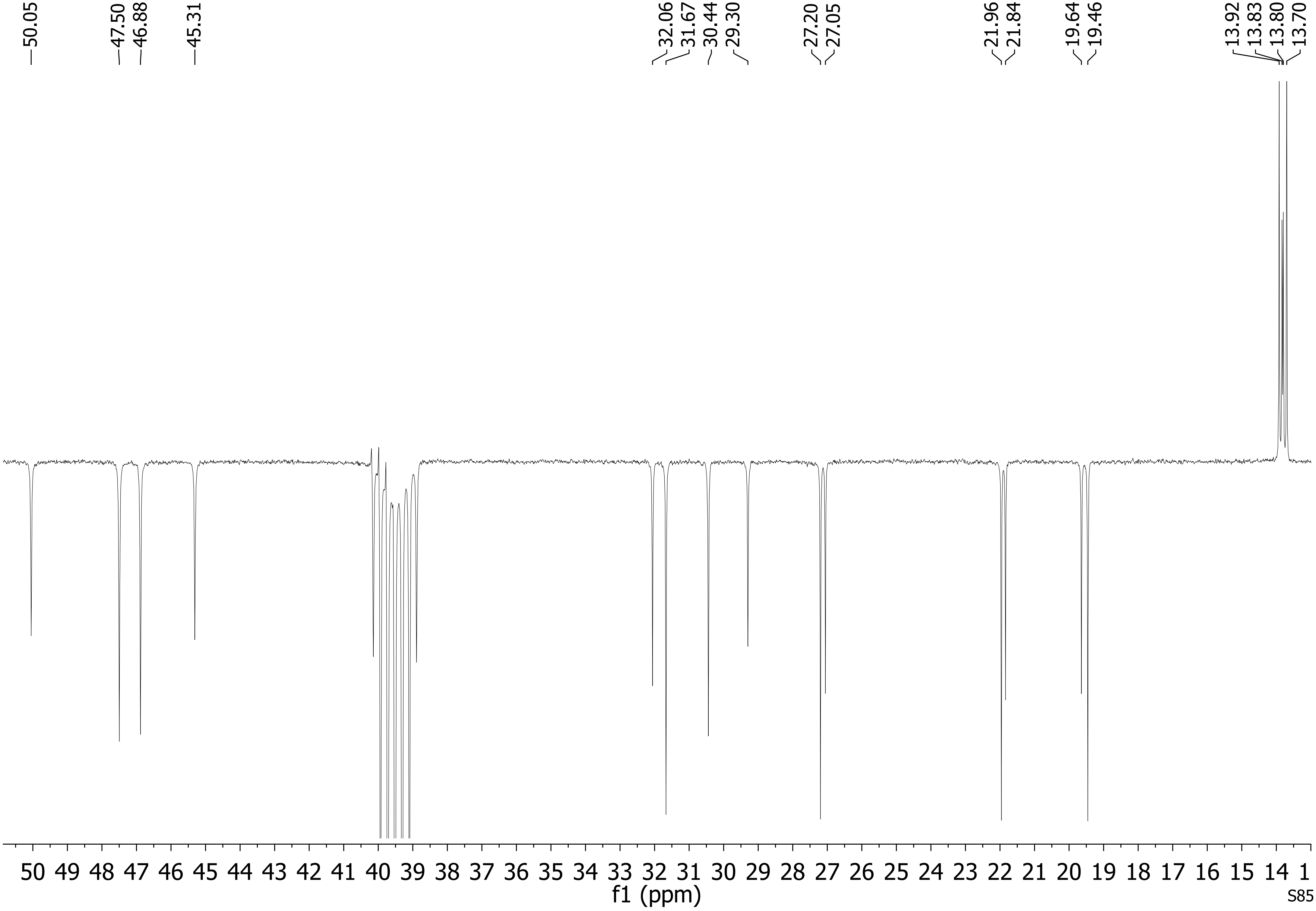

^{13}C NMR (101 MHz, DMSO-d_6) for compound **57** (zoomed-in view 2)

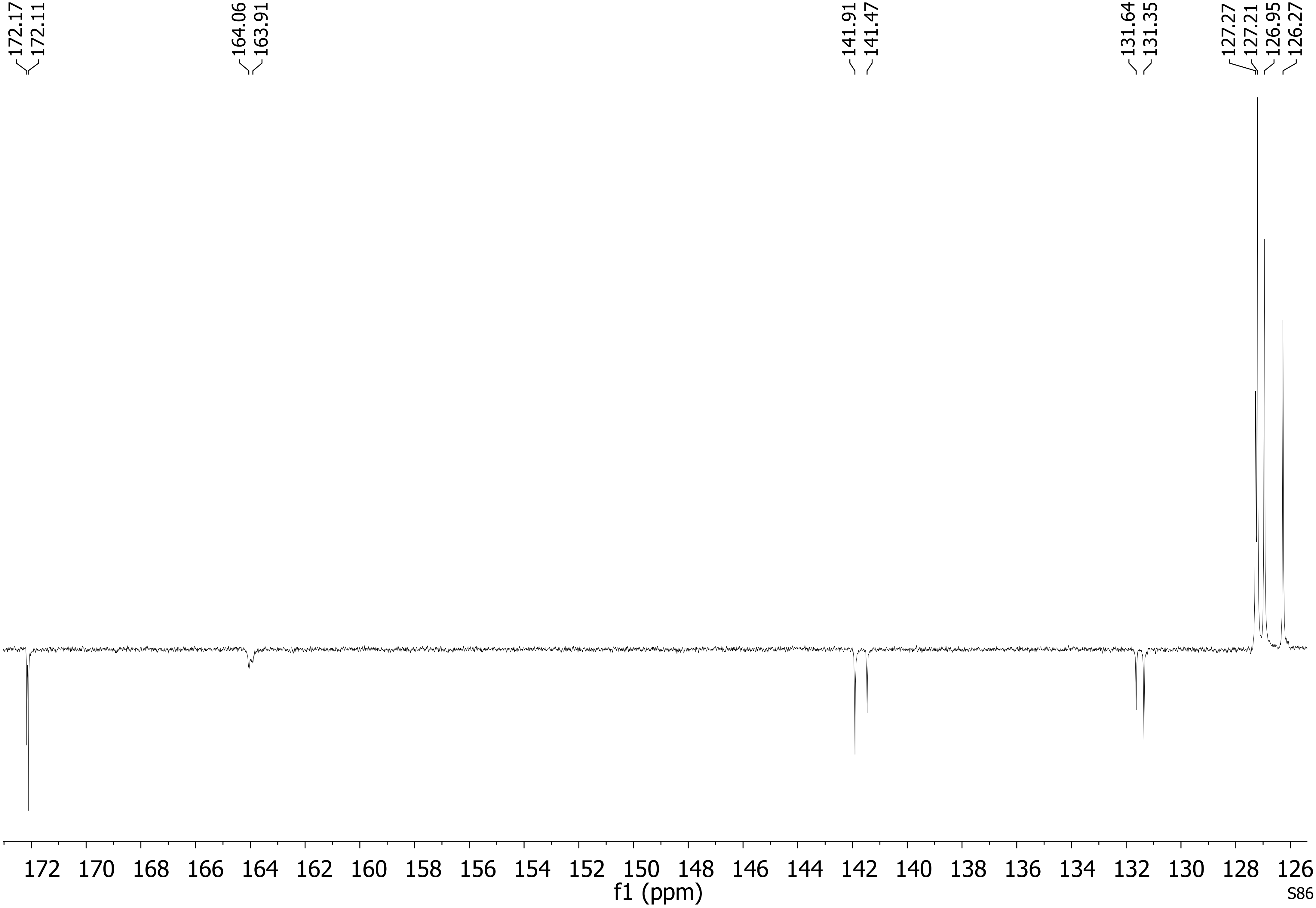

^1H NMR (400 MHz, DMSO-d_6) for compound **58**

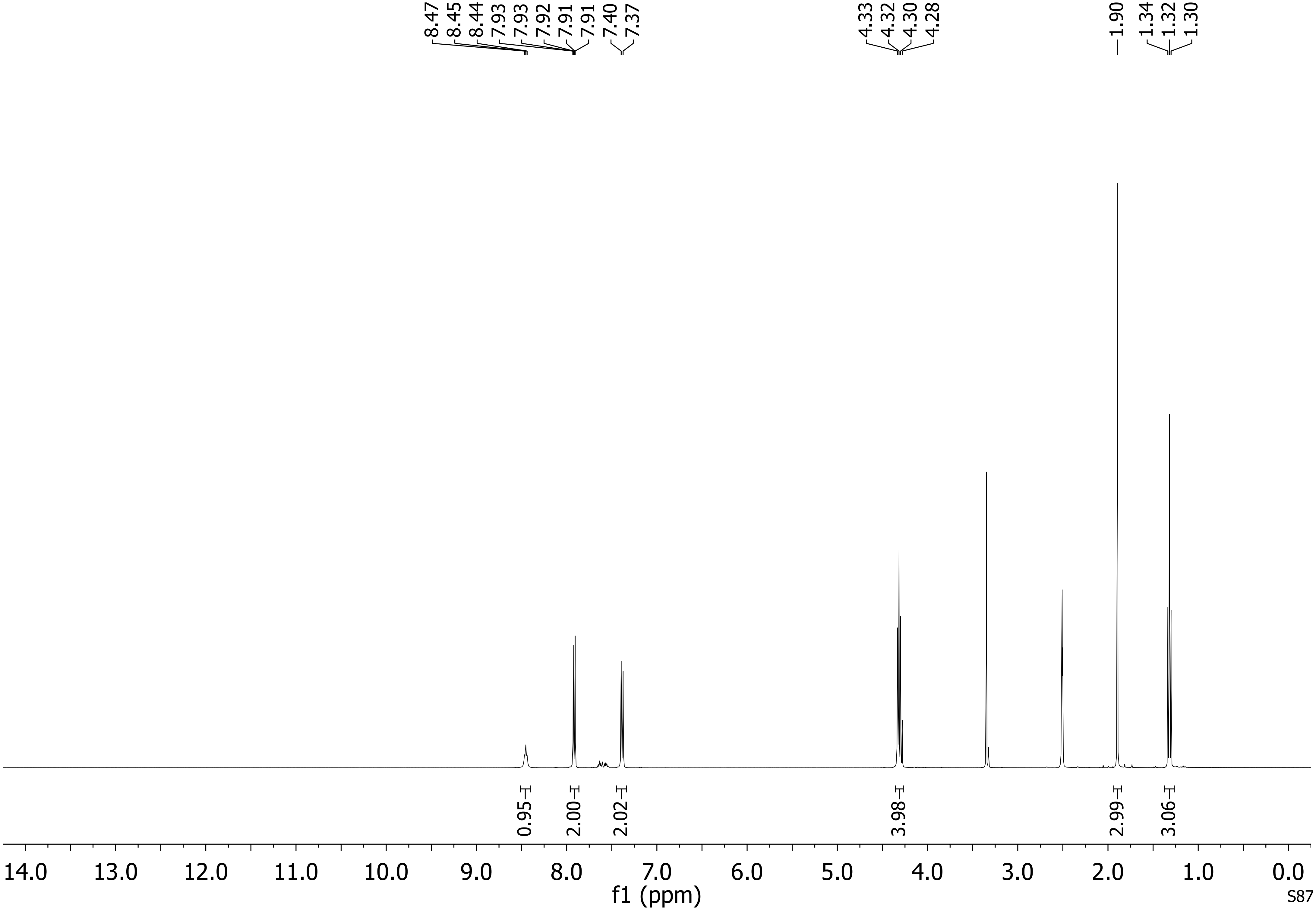

^{13}C NMR (101 MHz, DMSO-d_6) for compound **58**

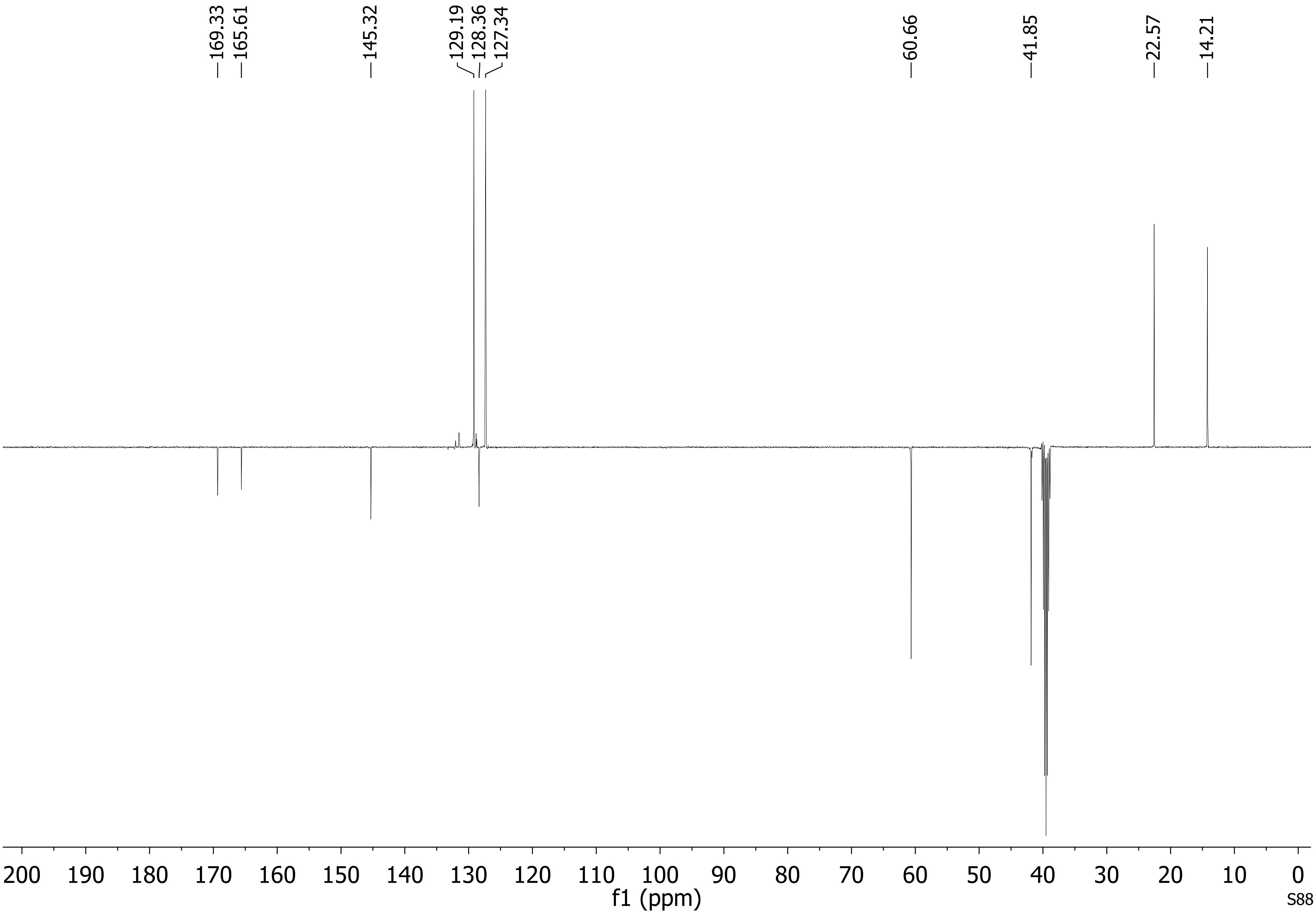

^{1}H NMR (400 MHz, DMSO-d_6) for compound **59**

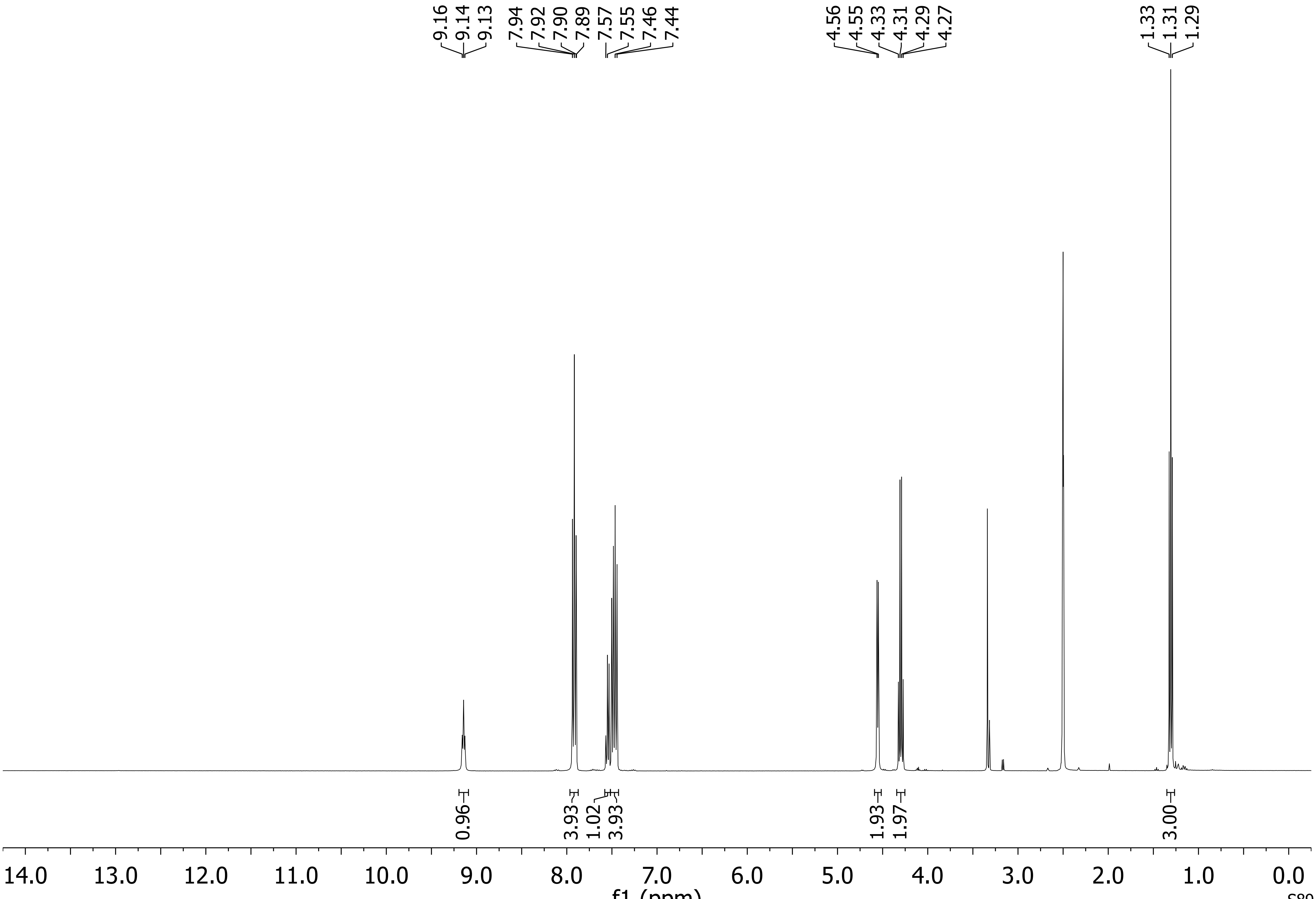

^{13}C NMR (101 MHz, DMSO-d_6) for compound **59**

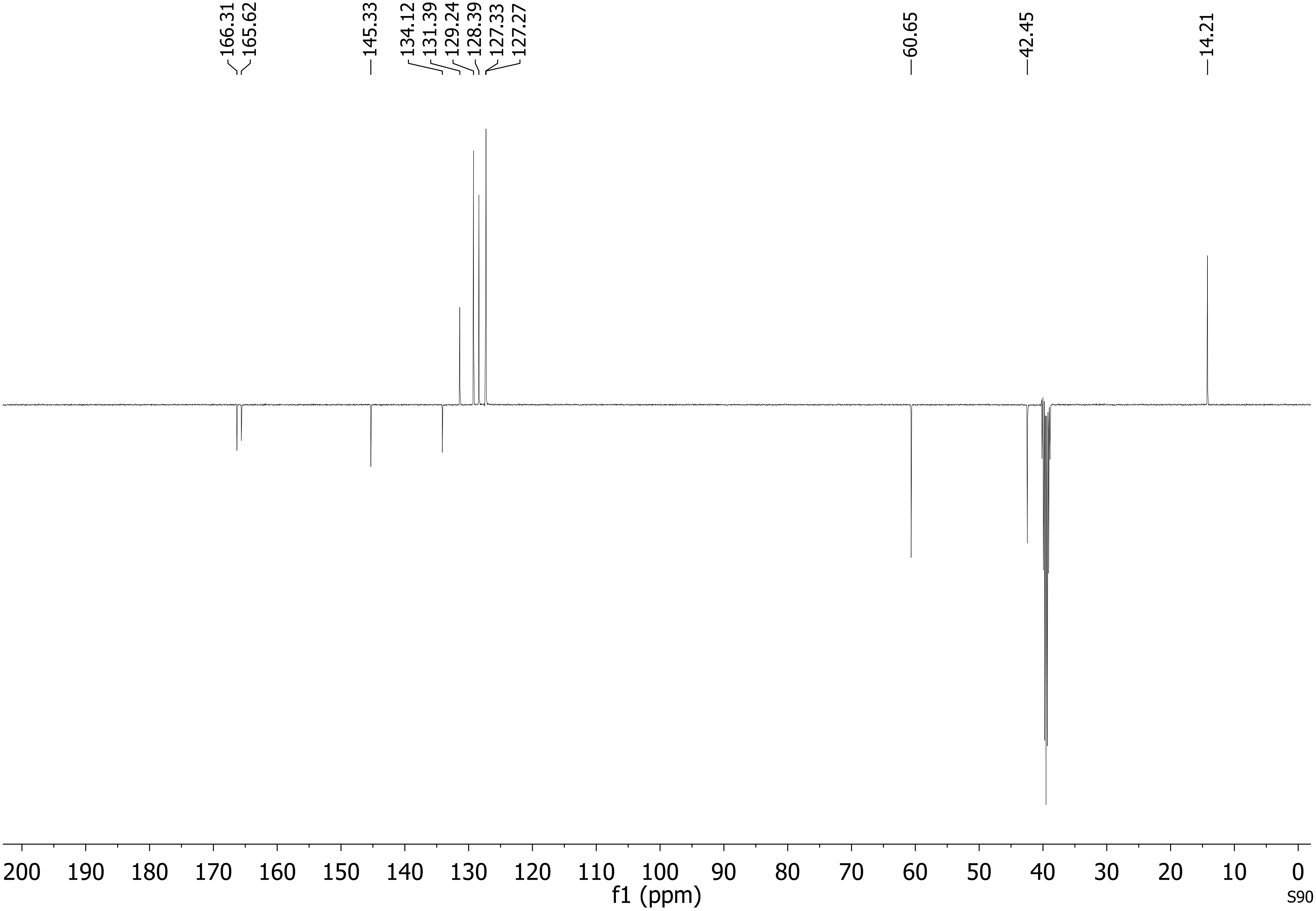

^{1}H NMR (600 MHz, DMSO-d_6) for compound **60**

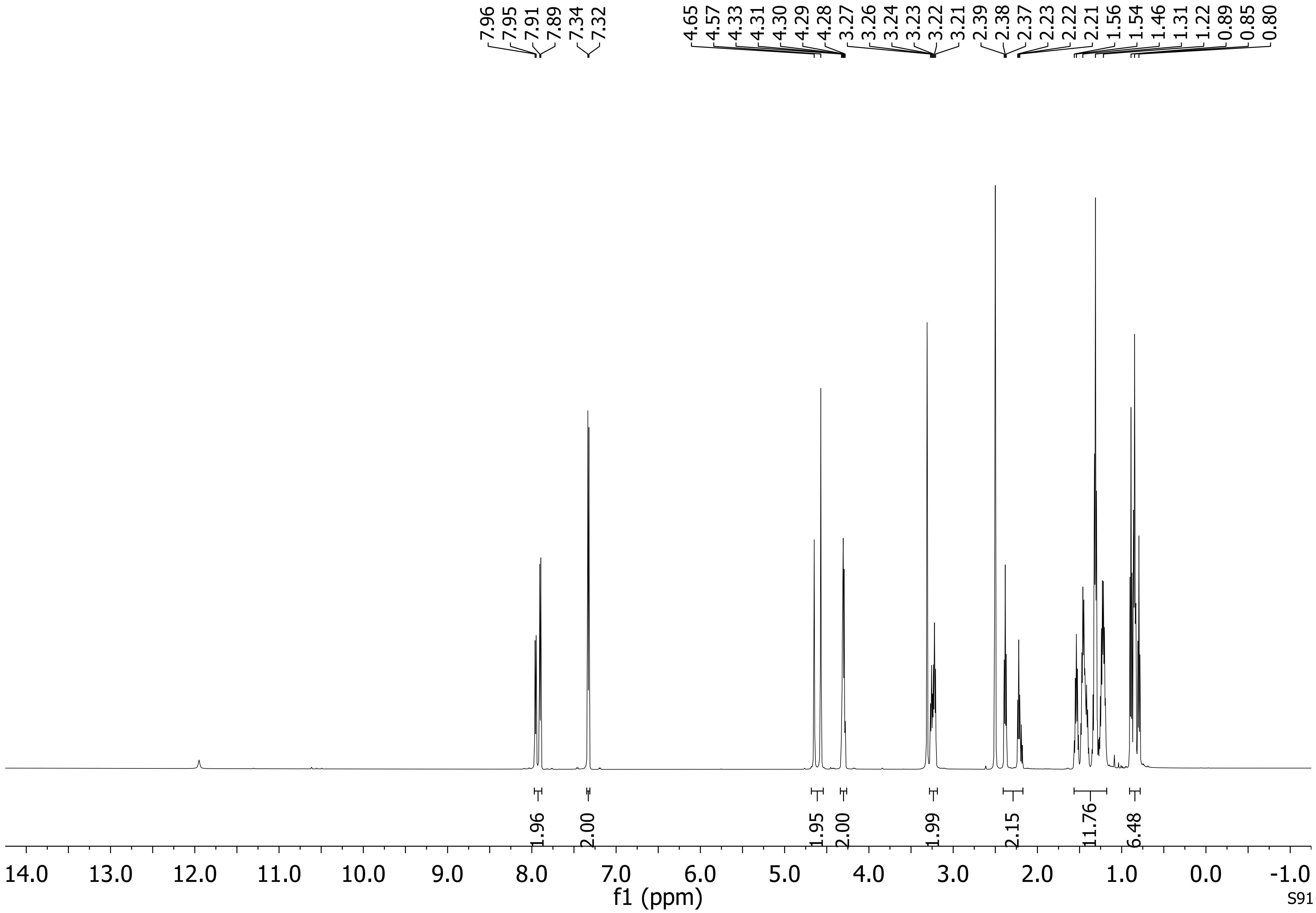

^{13}C NMR (151 MHz, DMSO-d_6) for compound **60** (full view)

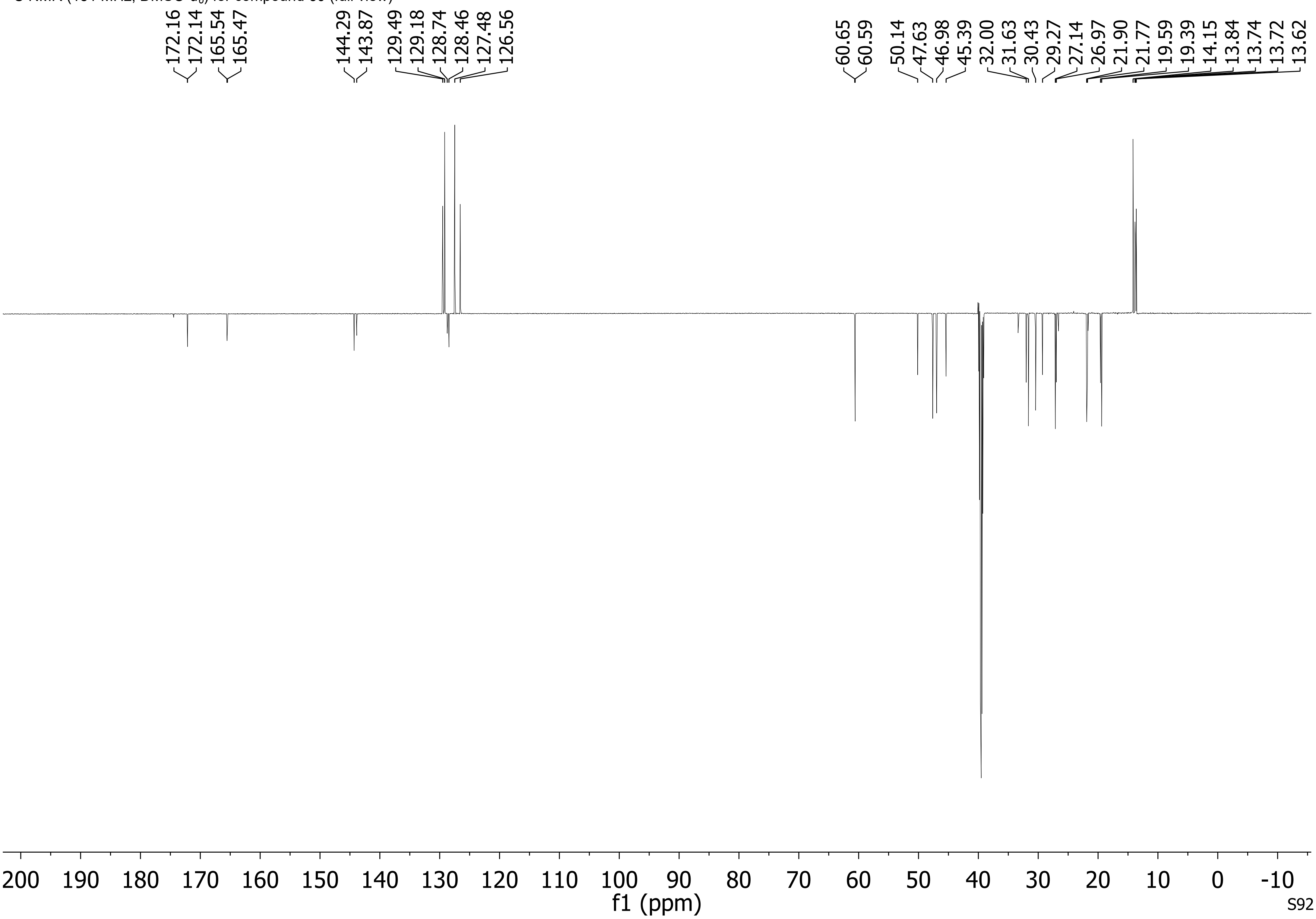

^{13}C NMR (151 MHz, DMSO-d_6) for compound **60** (zoomed-in view 1)

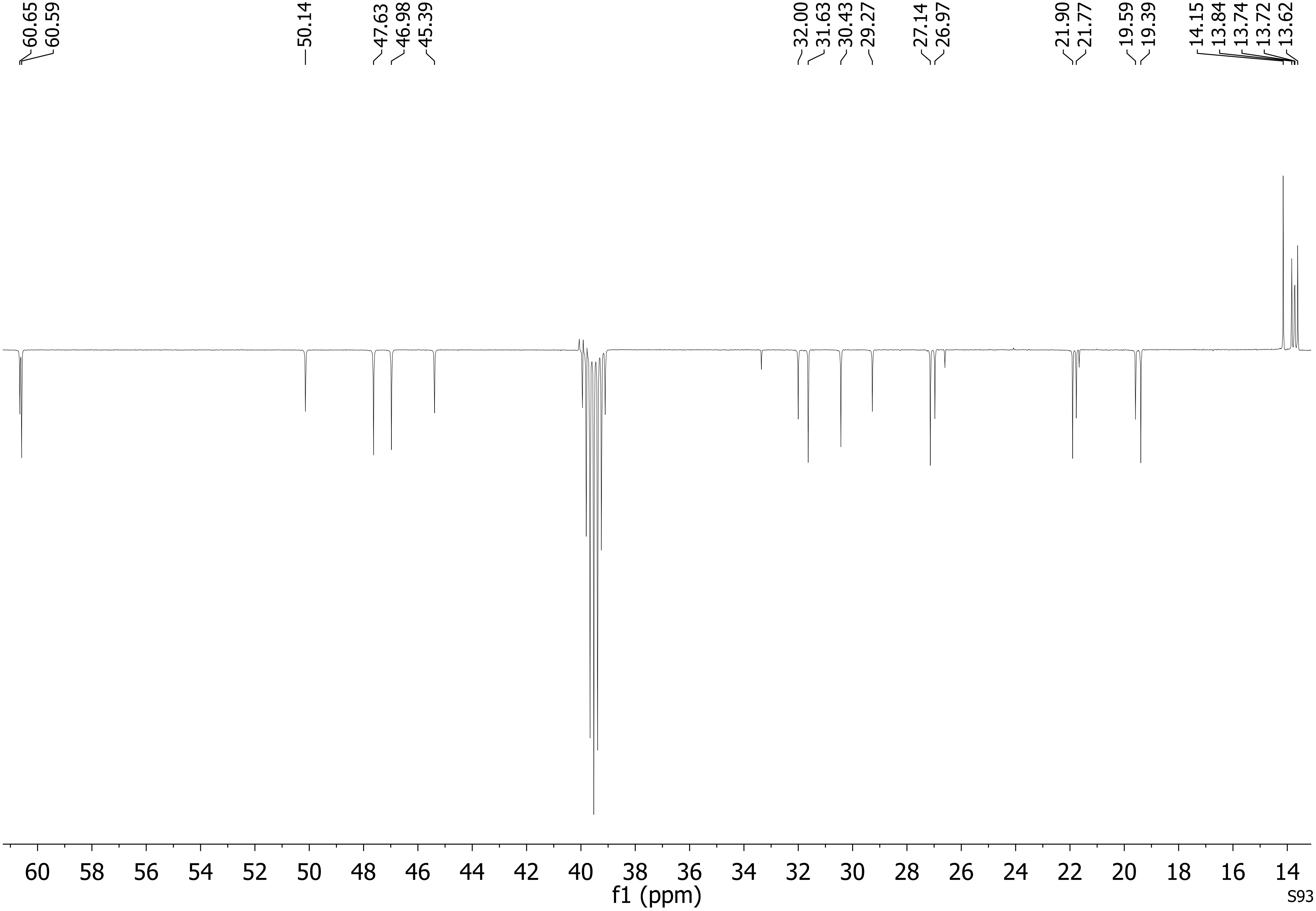

^{13}C NMR (151 MHz, DMSO-d_6) for compound **60** (zoomed-in view 2)

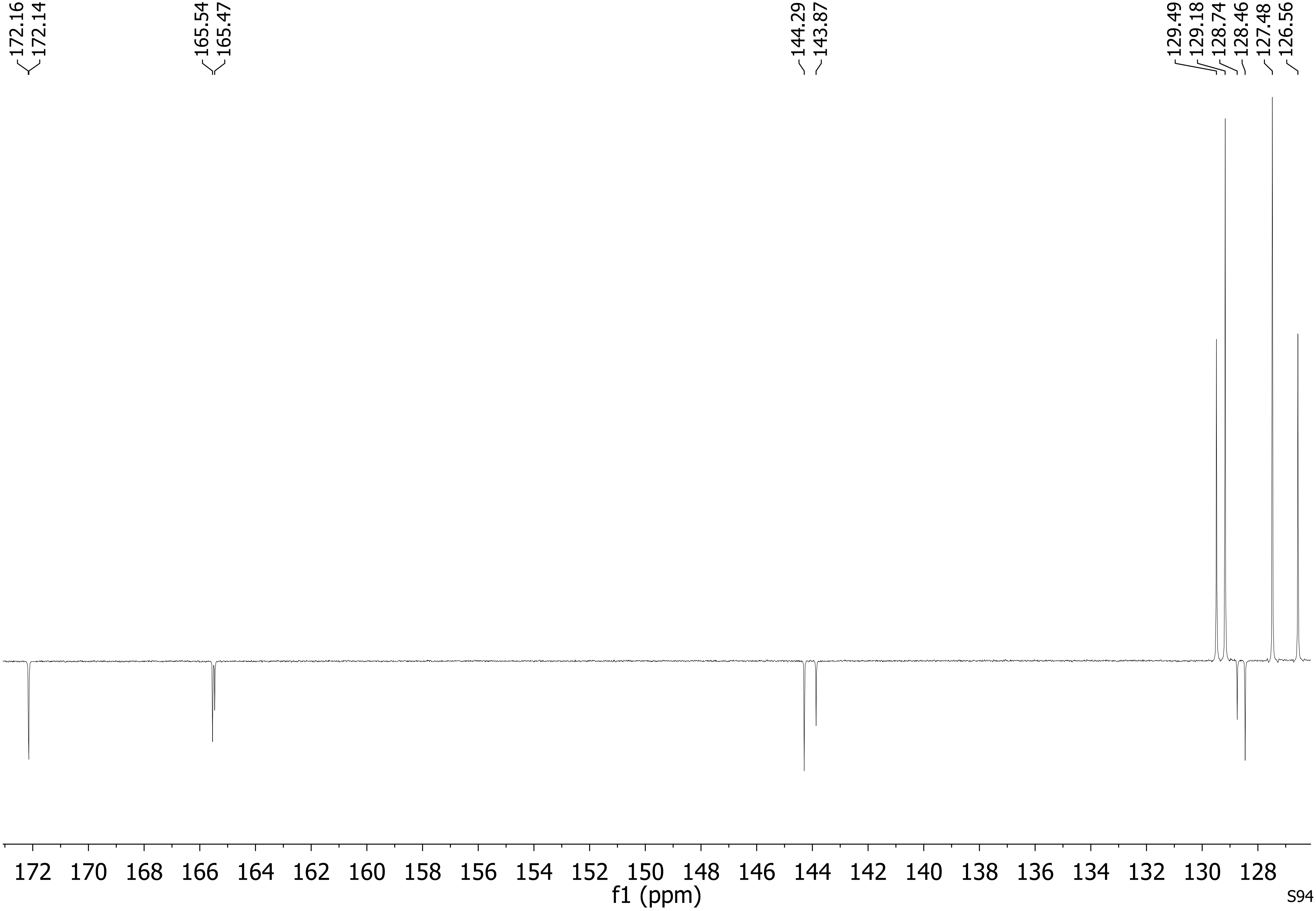

Exemplary HPLC Chromatograms

HPLC chromatogram of **21**:

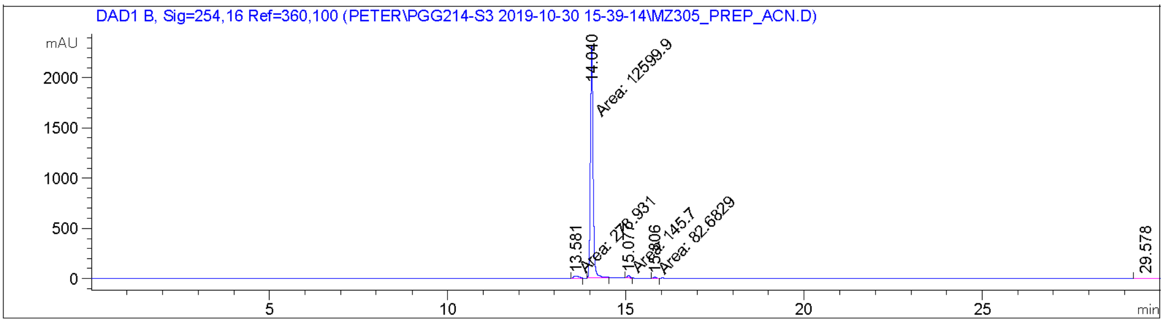


```
=====================================================================
                          Area Percent Report
=====================================================================

Sorted By             :      Signal
Multiplier            :      1.0000
Dilution              :      1.0000
Use Multiplier & Dilution Factor with ISTDs


Signal 2: DAD1 B, Sig=254,16 Ref=360,100

Peak RetTime  Type   Width     Area      Area    Name
  #   [min]          [min]   [mAU*s]      %
----|-------|------|-------|----------|--------|---------------------
   1  13.581 MM     0.1920  278.93054   2.1242 ?
   2  14.040 MM     0.0902 1.25999e4   95.9554 ?
   3  15.077 MM     0.0990  145.69951   1.1096 ?
   4  15.806 MM     0.1252   82.68288   0.6297 Ass
   5  29.578 BBA    0.2791   23.77905   0.1811 ?
                                                 ?

Totals :                   1.31310e4
```

HPLC chromatogram of **22**:

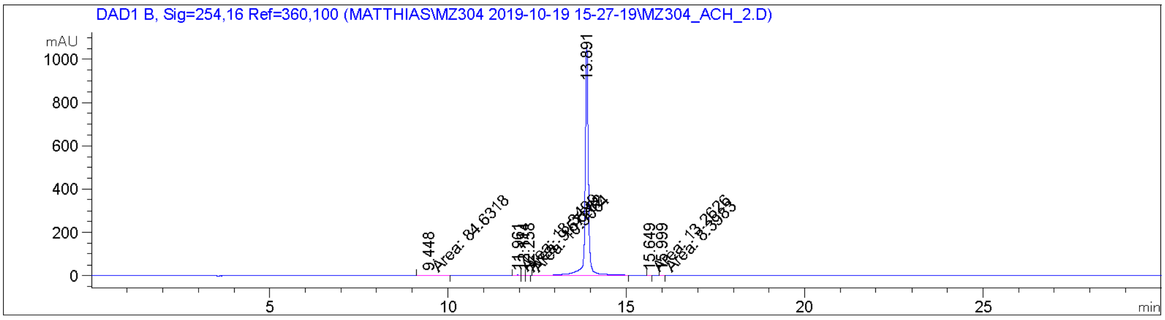


```
=====================================================================
                         Area Percent Report
=====================================================================

Sorted By             :      Signal
Multiplier            :      1.0000
Dilution              :      1.0000
Use Multiplier & Dilution Factor with ISTDs


Signal 2: DAD1 B, Sig=254,16 Ref=360,100

Peak RetTime  Type   Width     Area      Area    Name
  #   [min]          [min]   [mAU*s]      %
----|-------|------|-------|----------|--------|--------------------
   1   9.448 MM      0.7499   84.63179   1.1922 ?
   2  11.961 MF      0.1065   18.34994   0.2585 ?
   3  12.114 FM      0.1009    9.57282   0.1349 ?
   4  12.258 FM      0.1181   10.90641   0.1536 ?
   5  13.891 VB      0.0956 6953.65918  97.9557 ?
   6  15.649 MM      0.1124   13.26255   0.1868 Ass
   7  15.999 MM      0.1318    8.39830   0.1183 ?
                                                ?

Totals :                     7098.78100
```

HPLC chromatogram of **31**:

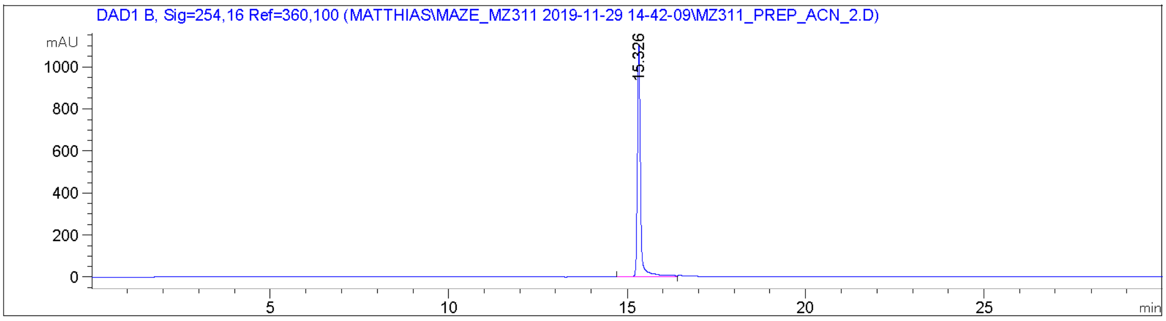

===

Area Percent Report

===

Sorted By : Signal
Multiplier : 1.0000
Dilution : 1.0000
Use Multiplier & Dilution Factor with ISTDs

Signal 2: DAD1 B, Sig=254,16 Ref=360,100

Peak #	RetTime [min]	Type	Width [min]	Area [mAU*s]	Height [mAU]	Area %
1	15.326	BV	0.0909	6727.89404	1106.98889	100.0000

Totals : 6727.89404 1106.98889

HPLC chromatogram of **32**:

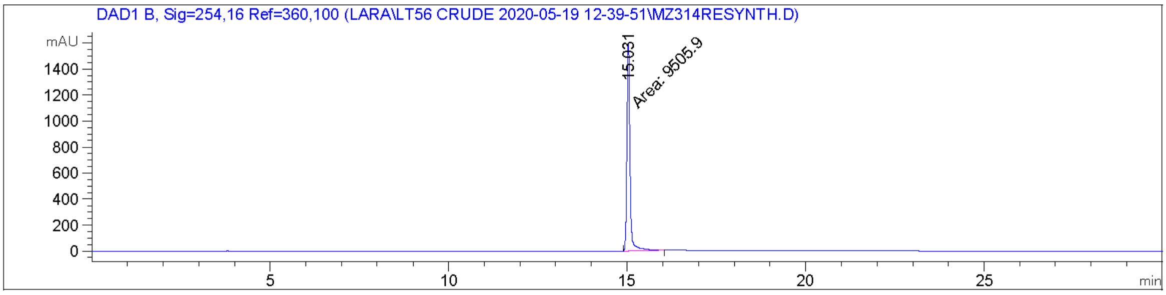


```
=====================================================================
                          Area Percent Report
=====================================================================

Sorted By             :      Signal
Multiplier            :      1.0000
Dilution              :      1.0000
Use Multiplier & Dilution Factor with ISTDs

Signal 2: DAD1 B, Sig=254,16 Ref=360,100

Peak RetTime Type  Width     Area      Height     Area
  #   [min]        [min]   [mAU*s]     [mAU]        %
----|-------|----|-------|----------|----------|--------|
   1  15.031 MM    0.0987 9505.90430 1605.17310 100.0000

Totals :                  9505.90430 1605.17310
```

HPLC chromatogram of **33**:

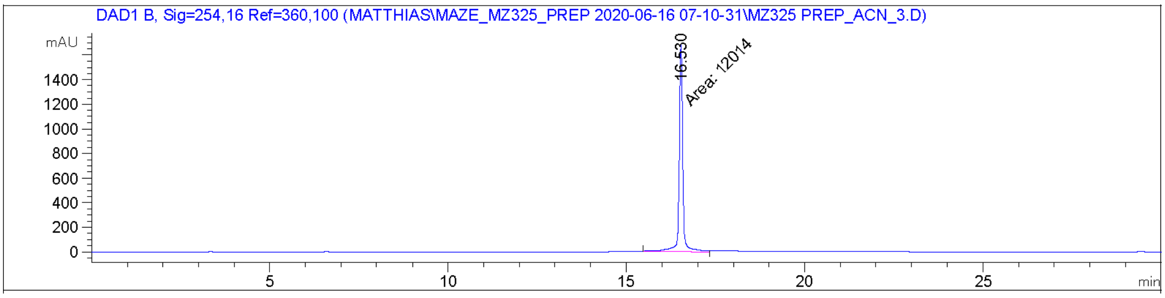


```
=====================================================================
                         Area Percent Report
=====================================================================

Sorted By             :      Signal
Multiplier            :      1.0000
Dilution              :      1.0000
Use Multiplier & Dilution Factor with ISTDs

Signal 2: DAD1 B, Sig=254,16 Ref=360,100

Peak RetTime Type  Width     Area      Height     Area
  #   [min]        [min]   [mAU*s]     [mAU]        %
----|-------|----|-------|----------|----------|--------|
   1  16.530 MM    0.1180 1.20140e4  1696.61670 100.0000

Totals :                  1.20140e4  1696.61670
```

HPLC chromatogram of **44**:

3 LS-97-Prep 2+3

MN Nucleodur 100-5 C18 ec

Sample Name:	**LS-97-Prep 2+3**	*Injection Volume:*	**10,0**
Vial Number:	**RE1**	*Channel:*	**UV_VIS_1**
Sample Type:	**unknown**	*Wavelength:*	**254.0**
Control Program:	**LS_5-95_15min_254nm**	*Bandwidth:*	**2**
Quantif. Method:	**Burda**	*Dilution Factor:*	**1,0000**
Recording Time:	**17.01.2020 16:36**	*Sample Weight:*	**1,0000**
Run Time (min):	**32,00**	*Sample Amount:*	**1,0000**

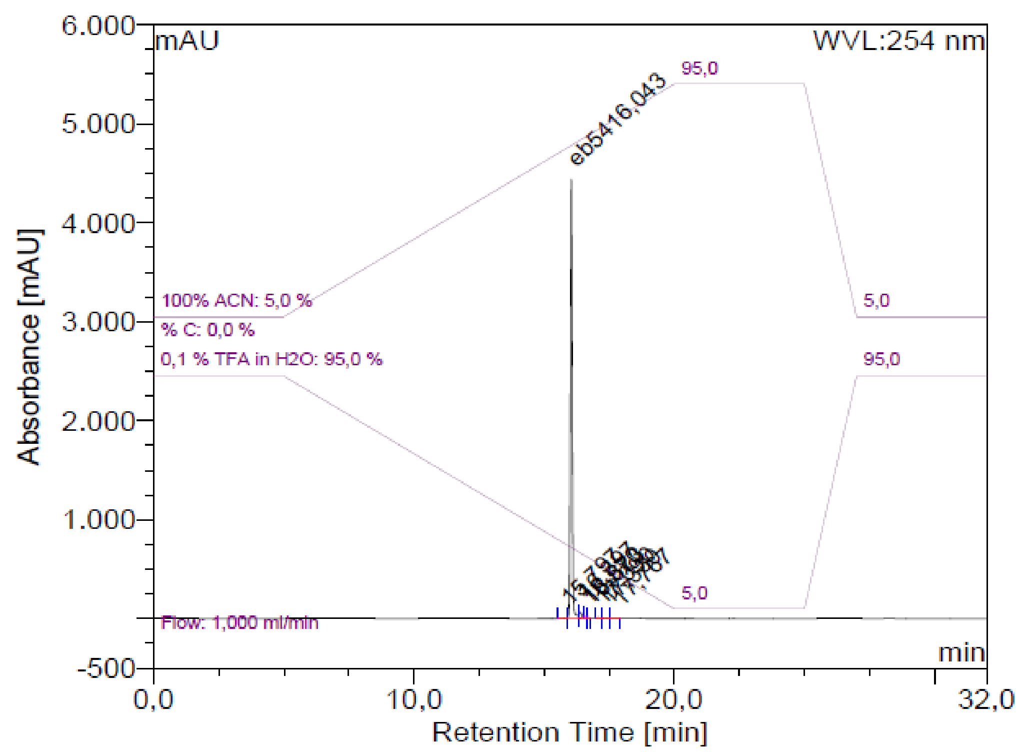

No.	Ret.Time min	Peak Name	Height mAU	Area mAU*min	Rel.Area %	Amount	Type
1	15,80	n.a.	5,219	0,680	0,17	n.a.	BM
2	16,04	eb54	#######	397,616	97,39	n.a.	M
3	16,40	n.a.	61,559	8,395	2,06	n.a.	M
4	16,57	n.a.	1,828	0,103	0,03	n.a.	Rd
5	16,67	n.a.	5,991	0,514	0,13	n.a.	MB
6	17,09	n.a.	4,872	0,411	0,10	n.a.	BMb
7	17,33	n.a.	3,955	0,410	0,10	n.a.	bMB
8	17,77	n.a.	0,886	0,142	0,03	n.a.	BMB
**Total:**			#######	408,271	100,00	0,000	

HPLC chromatogram of **45**:

3 LS-101 Prep2

MN Nucleodur 100-5 C18 ec

Sample Name:	LS-101 Prep2	*Injection Volume:*	3,0
Vial Number:	GD1	*Channel:*	UV_VIS_1
Sample Type:	unknown	*Wavelength:*	254.0
Control Program:	LS_5-95_15min_254nm	*Bandwidth:*	2
Quantif. Method:	Burda	*Dilution Factor:*	1,0000
Recording Time:	22.01.2020 17:09	*Sample Weight:*	1,0000
Run Time (min):	32,00	*Sample Amount:*	1,0000

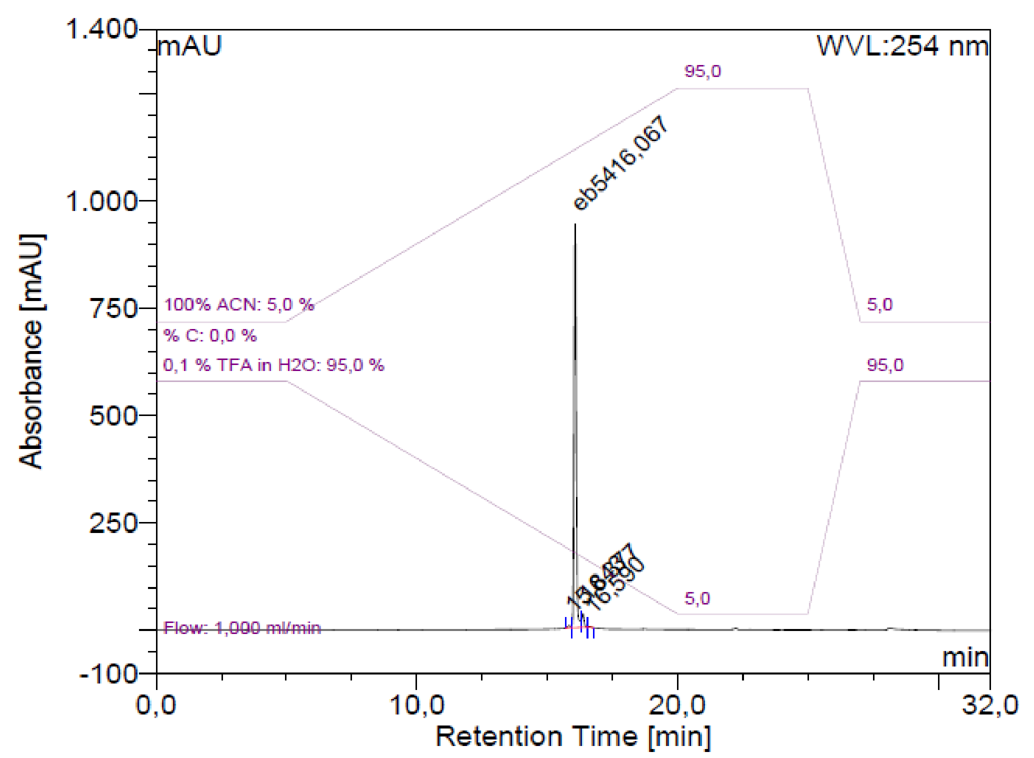

No.	Ret.Time min	Peak Name	Height mAU	Area mAU*min	Rel.Area %	Amount	Type
1	15,84	n.a.	5,407	0,660	0,73	n.a.	BM *
2	16,07	eb54	941,720	86,465	95,03	n.a.	M *
3	16,38	n.a.	31,528	3,632	3,99	n.a.	Mb*
4	16,59	n.a.	1,633	0,227	0,25	n.a.	bMB*
**Total:**			980,287	90,984	100,00	0,000	

HPLC chromatogram of **46**:

2 LS-130 Prep 1 H

MN Nucleodur 100-5 C18 ec

Sample Name:	**LS-130 Prep 1 H**	*Injection Volume:*	**10,0**
Vial Number:	**BE7**	*Channel:*	**UV_VIS_1**
Sample Type:	**unknown**	*Wavelength:*	**254.0**
Control Program:	**LS_5-95_15min_254nm**	*Bandwidth:*	**2**
Quantif. Method:	**Burda**	*Dilution Factor:*	**1,0000**
Recording Time:	**23.06.2020 12:56**	*Sample Weight:*	**1,0000**
Run Time (min):	**32,00**	*Sample Amount:*	**1,0000**

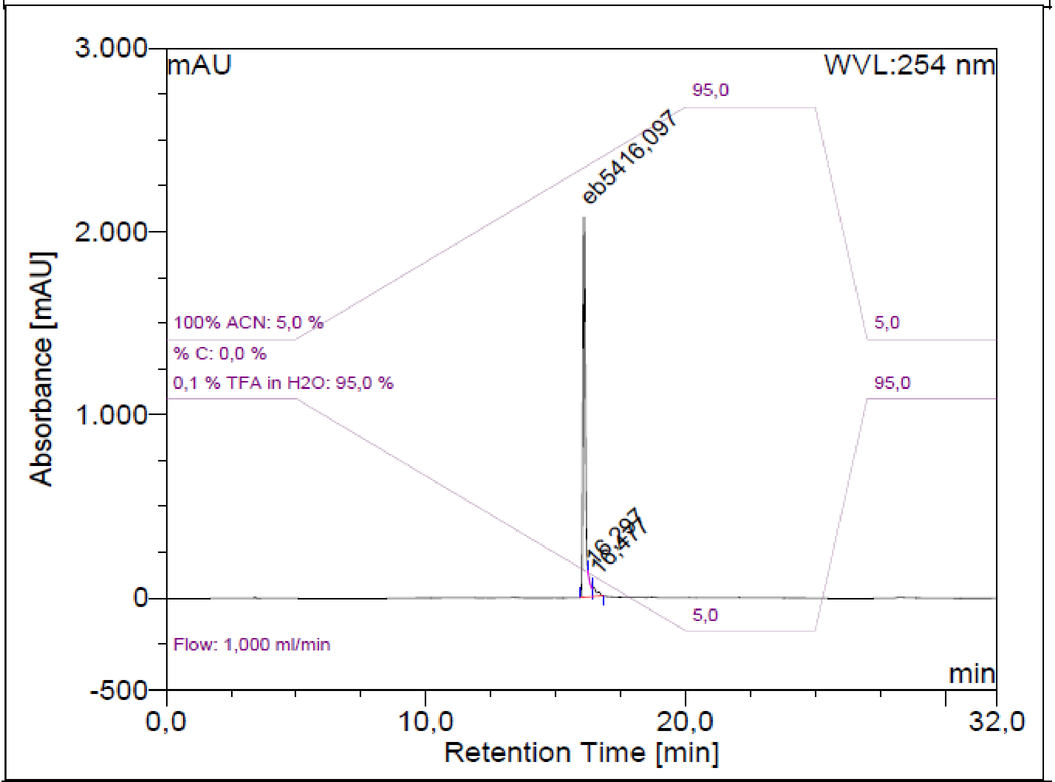

No.	Ret.Time min	Peak Name	Height mAU	Area mAU*min	Rel.Area %	Amount	Type
1	16,10	eb54	#######	221,954	95,26	n.a.	BM *
2	16,30	n.a.	14,262	1,164	0,50	n.a.	Rd*
3	16,48	n.a.	50,041	9,882	4,24	n.a.	MB*
**Total:**			#######	232,999	100,00	0,000	

HPLC chromatogram of **55**:

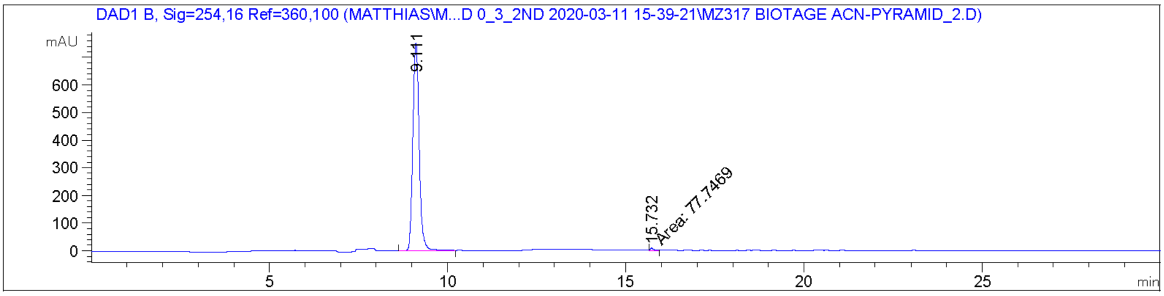


```
=====================================================================
                          Area Percent Report
=====================================================================

Sorted By             :      Signal
Multiplier            :      1.0000
Dilution              :      1.0000
Use Multiplier & Dilution Factor with ISTDs

Signal 2: DAD1 B, Sig=254,16 Ref=360,100

Peak RetTime Type  Width     Area      Height     Area
  #   [min]        [min]   [mAU*s]     [mAU]        %
----|-------|----|-------|----------|----------|--------|
   1   9.111 BB     0.1910 9205.39063  751.04425 99.1625
   2  15.732 MM     0.1346   77.74689    9.62935  0.8375

Totals :                   9283.13751  760.67360
```

HPLC chromatogram of **56**:

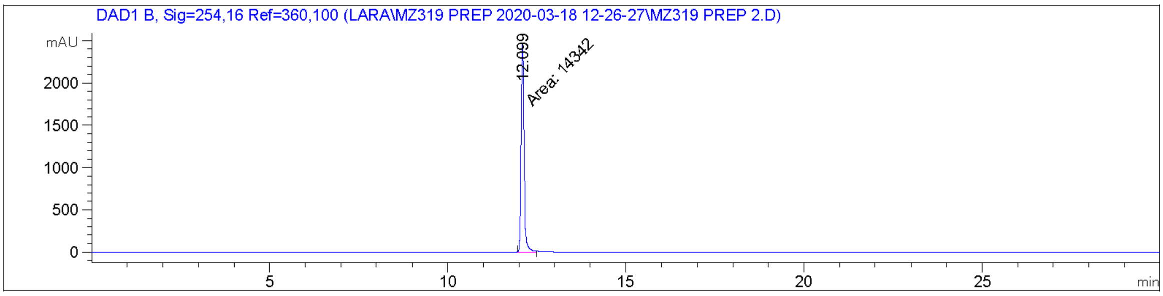


```
=====================================================================
                         Area Percent Report
=====================================================================

Sorted By             :      Signal
Multiplier            :      1.0000
Dilution              :      1.0000
Use Multiplier & Dilution Factor with ISTDs


Signal 2: DAD1 B, Sig=254,16 Ref=360,100

Peak RetTime Type  Width     Area      Height     Area
  #   [min]        [min]   [mAU*s]     [mAU]        %
----|-------|----|-------|----------|----------|--------|
   1  12.099 MM    0.0968 1.43420e4  2468.31201 100.0000

Totals :                  1.43420e4  2468.31201
```

HPLC chromatogram of **57**:

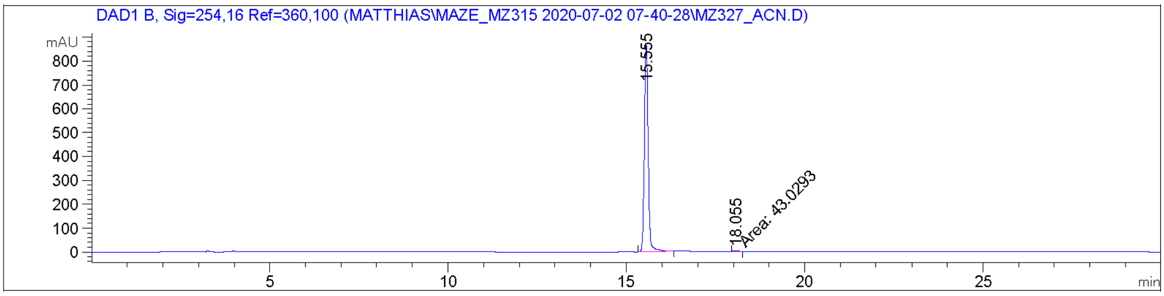


```
=====================================================================
                         Area Percent Report
=====================================================================

Sorted By             :      Signal
Multiplier            :      1.0000
Dilution              :      1.0000
Use Multiplier & Dilution Factor with ISTDs

Signal 2: DAD1 B, Sig=254,16 Ref=360,100

Peak RetTime Type  Width     Area      Height     Area
  #   [min]        [min]   [mAU*s]     [mAU]        %
----|-------|----|-------|----------|----------|--------|
   1  15.555 BB    0.1120 6289.54004  873.73260  99.3205
   2  18.055 MM    0.1449   43.02932    4.94885   0.6795

Totals :                  6332.56936  878.68146
```

Supplementary References

1. Zessin, M.; Meleshin, M.; Hilscher, S.; Schiene-Fischer, C.; Barinka, C.; Jung, M.; Schutkowski, M., Continuous fluorescent sirtuin activity assay based on fatty acylated lysines. *Int. J. Mol. Sci.* **2023,** *24*, 7416.
2. Schiedel, M.; Rumpf, T.; Karaman, B.; Lehotzky, A.; Gerhardt, S.; Ovadi, J.; Sippl, W.; Einsle, O.; Jung, M., Structure-based development of an affinity probe for sirtuin 2. *Angew. Chem. Int. Ed.* **2016,** *55*, 2252-2256.
3. Vogelmann, A.; Schiedel, M.; Wössner, N.; Merz, A.; Herp, D.; Hammelmann, S.; Colcerasa, A.; Komaniecki, G.; Hong, J. Y.; Sum, M., et al., Development of a NanoBRET assay to validate inhibitors of Sirt2-mediated lysine deacetylation and defatty-acylation that block prostate cancer cell migration. *RSC Chem. Biol.* **2022,** *3*, 468-485.
4. Swyter, S.; Schiedel, M.; Monaldi, D.; Szunyogh, S.; Lehotzky, A.; Rumpf, T.; Ovadi, J.; Sippl, W.; Jung, M., New chemical tools for probing activity and inhibition of the NAD(+)-dependent lysine deacylase sirtuin 2. *Philos. Trans. R. Soc. B* **2018,** *373*, 20170083.
5. Sinatra, L.; Yang, J.; Schliehe-Diecks, J.; Dienstbier, N.; Vogt, M.; Gebing, P.; Bachmann, L. M.; Sonnichsen, M.; Lenz, T.; Stuhler, K., et al., Solid-phase synthesis of cereblon-recruiting selective histone deacetylase 6 degraders (HDAC6 PROTACs) with antileukemic activity. *J. Med. Chem.* **2022,** *65*, 16860-16878.
6. Schäker-Hübner, L.; Haschemi, R.; Buch, T.; Kraft, F. B.; Brumme, B.; Scholer, A.; Jenke, R.; Meiler, J.; Aigner, A.; Bendas, G., et al., Balancing histone deacetylase (HDAC) inhibition and drug-likeness: Biological and physicochemical evaluation of class I selective HDAC inhibitors. *ChemMedChem* **2022,** *17*, e202100755.
7. De los Santos, M.; Martinez-Iglesias, O.; Aranda, A., Anti-estrogenic actions of histone deacetylase inhibitors in MCF-7 breast cancer cells. *Endocr. Relat. Cancer* **2007,** *14*, 1021-1028.
8. Jawaid, K.; Crane, S. R.; Nowers, J. L.; Lacey, M.; Whitehead, S. A., Long-term genistein treatment of MCF-7 cells decreases acetylated histone 3 expression and alters growth responses to mitogens and histone deacetylase inhibitors. *J. Steroid Biochem. Mol. Biol.* **2010,** *120*, 164-171.
9. Kalbas, D.; Liebscher, S.; Nowak, T.; Meleshin, M.; Pannek, M.; Popp, C.; Alhalabi, Z.; Bordusa, F.; Sippl, W.; Steegborn, C., et al., Potent and selective inhibitors of human sirtuin 5. *J. Med. Chem.* **2018,** *61*, 2460-2471.
10. Zessin, M.; Kutil, Z.; Meleshin, M.; Novakova, Z.; Ghazy, E.; Kalbas, D.; Marek, M.; Romier, C.; Sippl, W.; Barinka, C., et al., One-atom substitution enables direct and continuous monitoring of histone deacylase activity. *Biochemistry* **2019,** *58*, 4777-4789.
11. Brosowsky, J.; Lutterbeck, M.; Liebich, A.; Keller, M.; Herp, D.; Vogelmann, A.; Jung, M.; Breit, B., Syntheses of Thailandepsin B pseudo-natural products: Access to new highly potent HDAC inhibitors via late-stage modification. *Chem. - Eur. J.* **2020,** *26*, 16241-16245.
